# Chemical Deuteration of α-Amino Acids and Optical Resolution: Overview of Research Developments

**DOI:** 10.3390/bioengineering12090916

**Published:** 2025-08-26

**Authors:** Nageshwar R. Yepuri

**Affiliations:** Australian Nuclear Science and Technology Organisation (ANSTO), National Deuteration Facility, New Illawarra Road, Lucas Heights, NSW 2232, Australia; nageshwar.yepuri@ansto.gov.au

**Keywords:** amino acids, catalysis, specific deuteration and deuteration

## Abstract

Deuterium-labelled amino acids have found extensive applications in such research areas as pharmaceutical, bioanalytical, neutron diffraction, inelastic neutron scattering, in analysis of drug metabolism using mass spectrometry (MS), and, structuring of biomolecules by NMR. For these reasons, interest in new methodologies for the deuterium labelling of amino acids and the extent of their applications are equally rising. The ideal method will be able to label target compounds rapidly and cost-effectively by the direct exchange of a hydrogen atom by a deuterium atom. Most of these exchange reactions can often be carried out directly on the final target compound or a late intermediate in the synthesis, and often D_2_O can be used as the deuterium source. This review aims to provide a high-level overview of the chemical deuteration of amino acids in various groups (aromatic, heterocyclic, and non-aromatic α-amino acids). It primarily focuses on metal-catalyzed H/D exchange under hydrothermal conditions, with some attention given to studies on stereoselectivity and chemically synthesized perdeuteration and selective deuteration. In addition, we present different methods tested, manipulated, and developed for versatile new scalable protocols for preparation of selective and perdeuterated biologically important amino acids and their enzymatic and kinetic resolution to give pure enantiomers. Different methods for the synthesis of stereocontrolled selective and perdeuterated amino acids, including synthetic, and methods for preparing optically pure amino acids are presented.

## 1. Introduction

**Figure 1:**
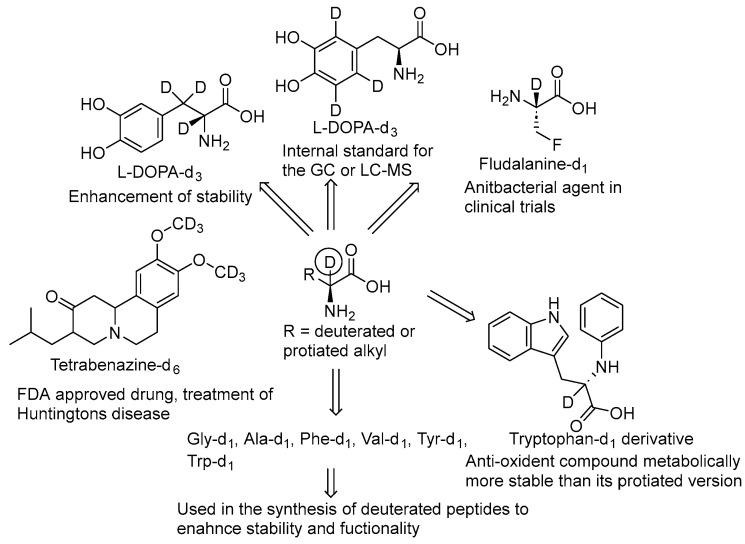
Some of the biological applications of deuterated amino acid.

Since the discovery of deuterium [1], there has been considerable interest in the preparation of deuterated compounds. The characteristic properties of deuterium compared with hydrogen generate a vast demand in numerous fields. Although deuterium (^2^H) is a stable isotope and not detectable by radioactive tracer techniques, it is readily monitored by other commonly available analytical methods, thus making deuterium a useful labelling agent. By analyzing the characteristic behavior of a deuterium-substituted compound using infrared spectroscopy [2,3], proton magnetic resonance (PMR) [4,5,6,7], mass spectrometry [8,9,10], and neutron scattering techniques [11,12], it is possible to obtain a broad range of information that is not easily accessible through conventional radioactive tracers. Some of the research has targeted the synthesis of deuterated compounds for biological investigations. The breakdown of drugs by Cytochrome P450 (CYP450) enzymes can be slowed by strategically replacing vulnerable carbon–hydrogen (C-H) bonds with more stable carbon–deuterium (C-D) bonds. This technique enhances a drug’s pharmacokinetics, improving how it functions in the body. The success of this method was validated in 2017 with the FDA approval of deutetrabenazine, a pioneering deuterated drug used to treat Huntington’s disease (Figure 1) [7]. Since amino acids are fundamental chemical building blocks for many biological components and serve as key components of peptide-based therapeutics, studies related to biomaterials might be facilitated using deuterated amino acids [5,7,10,13,14,15]. Deuterium-labelled amino acid-based compounds have found extensive applications in such research areas as pharmaceutical (Figure 1) [16,17,18,19], bioanalytical [19,20], biological, (Figure 1) [5,13,14,21] neutron diffraction [11,12,22], inelastic neutron scattering [12], Raman scattering [12], and in analysis of drug metabolism mechanisms [19,23] using mass spectrometry (MS) [8,18] and the structure of biomolecules by NMR [24,25]. Deuterium-labeled amino acids and their derivatives have proven to be invaluable tools for studying biosynthetic pathways, enzyme mechanisms, and the structures of peptides and proteins. Additionally, they play a significant role in improving absorption, distribution, metabolism, and excretion (ADME) profiling, as well as enhancing drug efficacy [26]. Furthermore, the pharmacokinetics and predicted metabolic sites of amino acid drug candidates deuterated at the α-position adjacent to nitrogen have been extensively examined (Figure 1). While the current review focuses on the deuteration of non-exchangeable protons, it is worth noting that deuteration of exchangeable protons has also been shown to affect protein–ligand binding affinities [27,28].

While improving metabolic stability is a well-known advantage of deuteration, its effect on protein–ligand binding affinity is a crucial and often overlooked aspect. This phenomenon can significantly influence the behavior of deuterated molecules in biological systems. Instead of synthesizing deuterated molecules, ligand binding to the histamine H_2_ receptor was studied by performing the experiments in heavy water (D_2_O) [28]. This method replaces all exchangeable hydrogen atoms in the protein with deuterium, allowing for the study of how hydrogen bonding affects the interaction. Using a range of computational techniques, the authors showed that deuteration increases histamine’s binding affinity for its H_2_ receptor. They concluded that this increased affinity is due to changes in hydrogen bonding, both at the receptor site and within the surrounding aqueous environment [27,28]. The results of the study highlight the importance of deuteration for the development of new drugs, as the selective replacement of exchangeable hydrogen atoms with deuterium can increase the duration of action due to their slower decomposition. This growing interest highlights the demand for these compounds and drives researchers to develop more efficient methods for their synthesis and direct deuteration methods for their production have appeared in the literature.

Neutron scattering offers a compelling complementary analytical approach. Its strength comes from its high sensitivity to deuterium. This unique property allows for researchers to exploit the differences in neutron scattering cross sections by substituting hydrogen atoms with deuterium in a sample [29]. The result is a significant reduction in background noise and enhanced contrast in specific areas [30]. Furthermore, the ability to perform site-selective deuteration provides an unparalleled means to obtain high-resolution information regarding conformational dynamics and structural alterations within targeted domains.

NMR spectroscopy is an invaluable technique for understanding protein structure and dynamics in solution, offering a robust method for determining 3D structures of proteins that resist crystallization [31]. However, its utility diminishes with increasing molecular mass due to spectral crowding and line broadening from fast transverse relaxation. While deuteration has long been used to simplify NMR spectra, achieving this requires stereospecific and regiospecific deuterated amino acids. Perdeuteration, while simplifying spectra, leads to a significant loss of crucial NOE information by removing all carbon-bound protons [31].

Deuteration of amino acids is achieved by either by direct deuteration or using deuterated precursors to synthesize final amino acids. Deuterium-labelled precursors are often prepared by direct exchange using transition metal-catalyzed hydrothermal or acid- or base-mediated reactions. These deuterated precursors are used in a multi-step reaction pathway to the final compound. The target compound is labelled rapidly and cost effectively by the direct exchange of a hydrogen atom by a deuterium atom [32]. Most of these exchange reactions can often be carried out directly on the final target compound or a late intermediate in the synthesis, and often D_2_O can be used as the deuterium source [33]. Direct deuteration on amino acids often resulted in partially deuterated compounds and the accompaniment of racemization. Direct deuteration is also sometimes used to achieve selective or site-specific H/D exchange. Direct deuteration, particularly using D_2_O as the deuterium source, offers several compelling advantages: D_2_O is generally the most affordable and readily available deuterium source. This significantly reduces the overall cost of deuteration, making it a highly economical option, especially for large-scale production. The exchange reaction can often be performed directly on the final target compound or a late-stage intermediate. This eliminates the need for lengthy, multi-step syntheses using expensive deuterated precursors, which can be time-consuming and resource-intensive. As a result, direct deuteration simplifies the experimental setup and execution, streamlining the entire process. However, its major limitations lie in achieving precise site-specificity and maintaining stereochemical integrity, which may necessitate alternative, more expensive, and complex methods for specific research or development goals. The commercial interest in these methods is evidenced from appearance of series of patents [34,35,36,37,38,39].

This review aims to provide a high-level overview of the chemical deuteration of amino acids in various groups (aromatic, heterocyclic, and non-aromatic α-amino acids). Methods for metal-catalyzed or chemically catalyzed selective deuteration and perdeuteration will be discussed. Most of the previous studies related to deuteration of amino acids are confined to specific deuteration [40,41,42,43,44]. In addition, methods related to deuteration through chemical synthesis and deuteration through stereoselective chemical synthesis also presented. Investigations related to different methods of transition metal- or chemical-catalyzed deuteration of biologically significant amino acids and their optical resolution methods from our laboratory will be discussed. A comprehensive summary of different selective and perdeuteration methods added at the end of this paper as a Table 1. It is our hope that this paper serve as useful guidance for scientists involved in the chemical deuteration of amino acids. Metal-catalyzed hydrothermal H/D exchange reactions in D_2_O are generally used to produce completely or partially deuterated organic compounds [45,46]. Direct deuteration of amino acids using metal-catalyzed hydrothermal H/D exchange reactions (typically >200 °C and >20 bar) would cause several types of undesirable side reactions, such as epimerization or the cleavage of amine or acid groups, and decomposition.

## 2. Methods

**Scheme 1:**
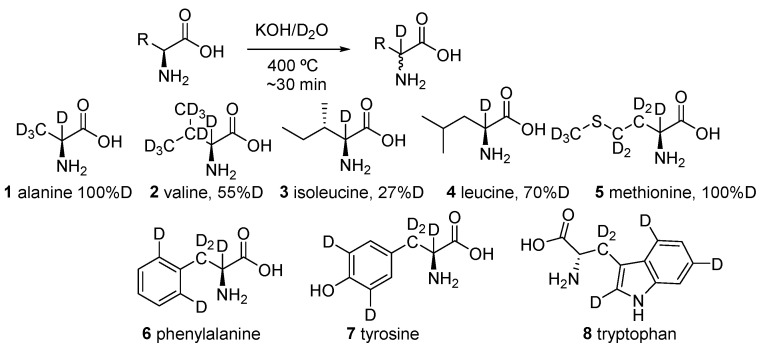
Deuteration of amino acids under basic conditions at supercritical temperatures.

A direct and non-destructive perdeuteration method would be a valuable method for the commonly available amino acids so that they may be obtained in high quantity, in a more routine-based manner, high in isotopic (above 90%D) content, without loss of chirality, and at low cost. The reactivity of the C-H bonds of amino acids in supercritical-temperature deuteroxide solutions was tested to examine the acid or base reactivity of C-H bonds. Deuteration of aromatic and non-aromatic compounds at the supercritical temperature in D_2_O is a known technique [47,48,49]. Ying Yang and Evilia attempted direct deuteration on commonly available amino acids at 400 °C in basic conditions (Scheme 1) with heating time varied between 5 to 10 min [50]. H/D exchange is found to be rapid at α carbon in all examples and lower deuteration also observed at alkyl chains and aromatic rings. Decomposition occurred in cases when having OH, SH, SS, or C=N groups on alkyl chains except for methionine **5** (Scheme 1). However, if any deuteration was observed at the chiral center, it was often accompanied by racemisation [50]. Extended heating significantly enhanced deuteration in various amino acids. For alanine, complete deuteration at both the α-position and methyl groups was achieved within 40 min. Methionine’s S-CH_3_ group also reached full deuteration with a shorter reaction time of 10 min. However, for isoleucine, leucine, and valine, longer reaction times (30 to 40 min) resulted in only partial deuteration: 27% for isoleucine, 70% for leucine, and 55% for valine’s methyl groups. Rapid and complete deuteration was observed at methylene -CH_2_CO_2_H in glutamic acid within 5 min, even before α-deuteration. The mechanism for this exchange seems to go through an increase in C-H activation (acidity) under basic supercritical temperature conditions, leading to carbanion formation. The reason for the rapid exchange at the α-position is lower stability of the tertiary carbanion. Loss of chirality at chiral centers might have resulted due to the incoming deuterium attacking either side of the carbanion ion.

The group further explored direct deuteration of amino acids at 400 °C in strong acid [50], with α-carbon exchange observed in all cases [50]. Racemization occurred at the chiral centers, as was previously observed for the reaction under basic conditions.

**Scheme 2:**
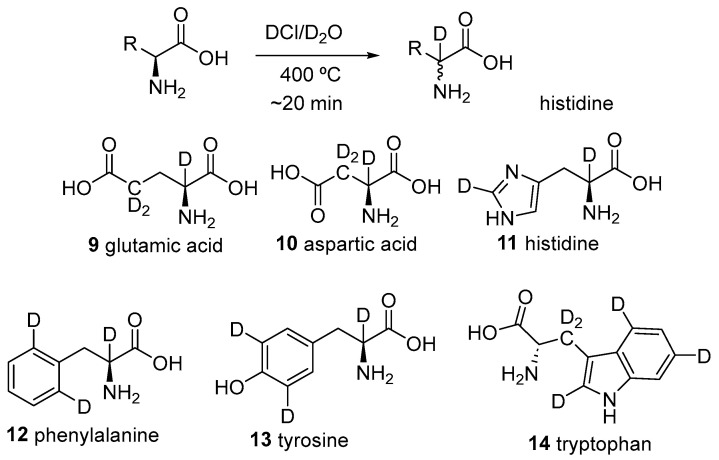
Deuteration of amino acids under acidic conditions at supercritical temperatures.

**Scheme 3:**
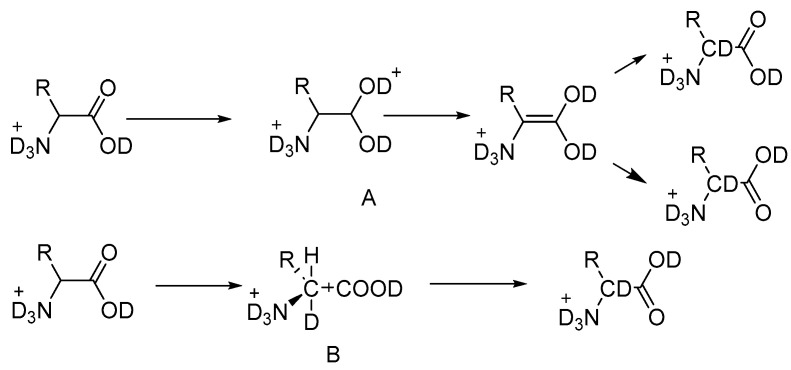
Mechanism of chirality loss and α-carbon deuteration under acidic conditions.

In case of glutamic acid **9** and histidine **11**, it seems supercritical temperature is not required for deuteration (Scheme 2) [50]. Phenylalanine **12** was found to be deuterated at α-carbon, -CH_2_-, and ring deuteration was observed at *ortho* to -CH_2_- group at 400 °C; similarly, ring deuteration on tyrosine **13** was observed at *ortho* to OH at the same temperature. Deuteration pattern in tryptophan **14** was completely deuterated at α-β-carbon, 2, 4, and 6 positions on hetero cyclic ring and partial deuteration occurred at 5 and 7 positions. Regarding deuteration under supercritical strong acidic (8% DCl) conditions, all the amino acids studies were found to be deuterated at α-carbon (Scheme 2) with loss of chirality except for cysteine, methionine, serine, and threonine, which decomposed rapidly [50]. Acidic conditions are less suitable for production of deuterated amino acids because of the greater degree of decomposition and racemization than basic conditions. All others that survived under these conditions with the deuteration sites are shown in Scheme 2. The mechanism seems to be slightly different compared to basic conditions. There are two possible mechanisms proposed in the paper [50] (Scheme 3A,B), the first is the loss of chirality and deuteration at α-carbon center through the formation of super acid, and the second is through formation of carbocation, as shown in Scheme 3A,B [50].

### 2.1. Specific Deuteration

Selectively deuterium-labelled amino acids are incorporated biosynthetically into fully assembled biological units and subsequently use the label to monitor the site of interest on the macromolecule [51]. Selective deuteration of amino acids is often useful for the study of biological macromolecules by NMR and other biophysical techniques. Proton NMR investigations of proteins have provided a great deal of information about conformation, dynamics, and microscopic states of amino acid residues. For example, the C2-proton of histidine, which resonates downfield from the rest of the aromatic resonance envelope, has been studied extensively in a number of proteins [51]. Regio- and stereoselective deuterium-labelled amino acids also have an important application as probes for the elucidation of biosynthetic pathways or reaction mechanisms by NMR and MS either as a substrate or added into peptides or proteins. One hurdle for such studies has been the limited availability and/or high cost of selectively deuterated amino acids. α-Deuterated amino acids play an indispensable role in the emerging area of clinical functional metabolomics, providing various essential data on in vivo amino acid metabolism and protein turnover [32]. The goal of clinical metabolomics is to identify metabolic signatures in bodily fluids. These signatures can then be used to assess a person’s current health and predict their risk of developing diseases [52]. α-Deuterated L-valine is incorporated biosynthetically into *L. casei* dihydrofolate reductase (DHFR); this allows comparison of the α-CH-NH fingerprint regions of COSY spectra of deuterated and normal DHFR (Dihydrofolate Reductase) complexes to identify cross-peaks from 15 of the 16 valine residues [5]. Over recent years, the asymmetric synthesis of α-deuterated α-amino acids has received due attention; however, the issues of selectivity, level of deuteration, and enantiomeric purity of the target products still leave room for improvement [32]. Therefore, the development of new methods for the synthesis and resolution of α-deuterated amino acids is highly desirable.

**Scheme 4:**
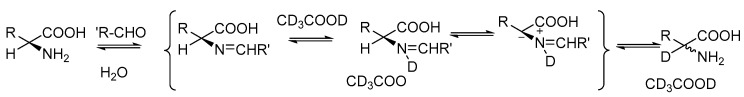
Mechanism of α-deuteration of amino acids via a Schiff base intermediate.

Through a simple method, racemic α-deuterated amino acids were prepared by heating corresponding amino acids with benzaldehyde in acetic acid-d_4_ [18,44]. The isotopic purity of the products was more than 99.5% and α-deuterated amino acids were racemized. Authors used the racemization method (via Schiff base intermediate) to exchange the deuterium at α-position of amino acids in deuterated acid (Scheme 4) [44]. These DL-α-deuterated amino acids were converted to methyl esters subsequently resolved by the enzyme alcalase via selective ester hydrolysis to give highly enantiomerically pure amino acids [53].

**Scheme 5:**
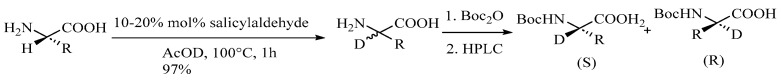
Catalytic α-deuteration using phenol-crosslinked salicylaldehyde-formaldehyde polymer and subsequent isomer separation.

A general rapid, inexpensive, and generally applicable method for preparation of α-deuterated α-amino acids starting from commercially available amino acids was described by Tikhonov et al. [42]. DL-α-Amino acids were deuterated at the α-position using D_2_O and phenol-crosslinked salicylaldehyde–formaldehyde polymer catalyst (Scheme 5). This process involves replacing hydrogen atoms with deuterium atoms at the α-carbon of the amino acid. The use of a polymer catalyst suggests a heterogeneous reaction, where the catalyst is a solid material, and the reaction takes place on its surface. The deuteration is likely achieved through an exchange reaction facilitated by the catalyst. The preparation of the catalyst is also described in the paper [42]. The method of separation of two isomers seems achievable on *N*-boc-protected amino acids using a chiral HPLC technique [42].

**Scheme 6:**
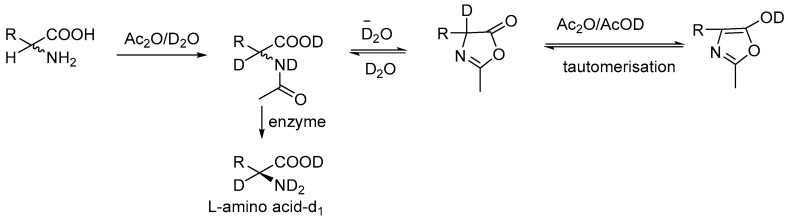
α-Deuteration likely proceeds via an oxazolidinone intermediate.

Selective α-deuteration also occurred through the racemization of amino acids, which employs refluxing acetic acid-d_1_ and acetic anhydride to give α-deuterated racemic *N*-acetylated amino acids [41]. A mixture of large excess acetic anhydride with D_2_O is used to give a solution of Ac_2_O in AcOD. Treatment of amino acids with this solution at reflux temperature for a few minutes leads to acylation, racemization, and H/D exchange at the α-carbon. One possible mechanism for the reaction is through the formation of the oxazolidinone intermediate as shown in Scheme 6 [41]. Several other amino acids were also deuterated at the α-position; the first cycle gave 70–80% exchange and a second cycle gave 90–100% exchange. This method can also be used for the exchange of tritium onto the α-position of amino acids [41]. This process directly yields acetylated products, which are the starting material for enzymatic resolution. This method also proved to preserve stereocenters other than at the α-carbon. Mitulovi et al. also reported that the above racemic α-deuterated *N*-acetylated amino acids separated into (S) and (R) by HPLC liquid chromatography on preparative scale using a chiral stationary phase based on quinine carbamate [54]. Enantiomerically pure (R) and (S) amino acids can be obtained in good yields by hydrolyzing the *N*-protecting group. Usually, an enzyme is required for the preparation of stereoselectively labelled amino acids. This method is notable for the absence of any enzyme involvement. The authors also demonstrated that this can be extended to secondary amino acids like proline [54].

**Scheme 7:**
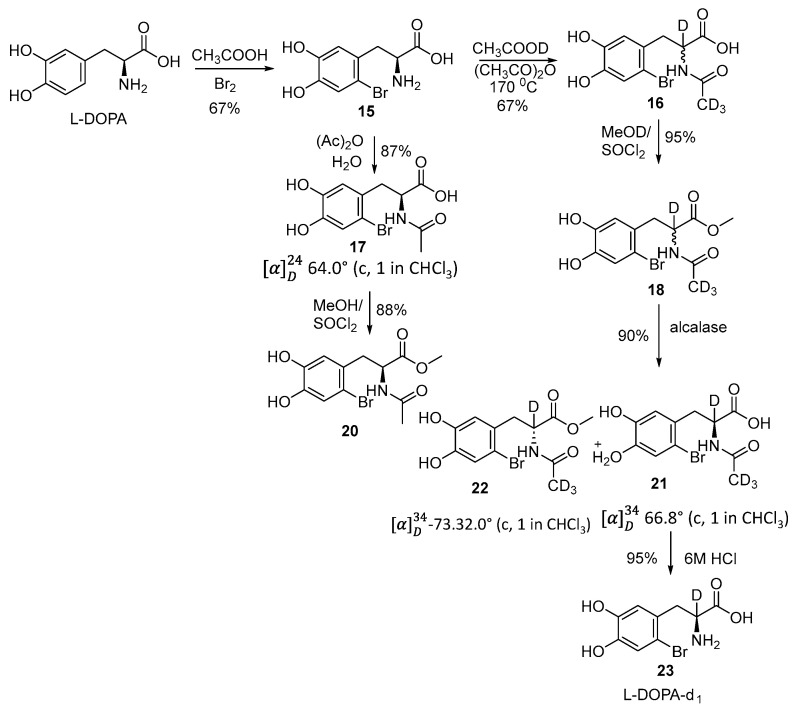
Synthesis and resolution of α-deuterated Br-L-DOPA derivatives from our laboratory.

Novel radiosyntheses of [^18^F]fluorinated aromatic amino acids and their α-deuterated forms were developedand utilized compounds in preclinical positron emission tomography (PET) studies in order to test the hypothesis that α-deuteration should afford superior tracers for PET studies of dopamine and serotonin synthesis in living brain. Part of the synthesis of [^18^F]DOPA image tracer and its α-deuterated isotopologue can be achieved through synthesis of the corresponding bromo analogues followed by nucleophilic [^18^F]-fluorine labelling. The commercially available L-DOPA (Scheme 7) was brominated at the 6 position by using molecular bromine in acetic acid to give bromo compound **15** in good yield [55]. Pre-exchanged compound **15** was heated in acetic anhydride and monodeuterated acetic acid to give a racemic mixture of α-deuterated *N*-acetylated **16** with 92%D level [56]. The racemic mixture **16** was esterified and followed by an efficient alcalase enzymatic selective hydrolysis of the *L*-isomer only to give **21** and **22** unhydrolyzed as a Br-D-DOPA-d_1_ acetyl ester (Scheme 7) [57]. The enantiomeric purity of **21** is confirmed by comparing optical rotation of the protonated version of **17** [57], which was synthesized according to a procedure from the literature (Supplementary Information) [58]. N-acetyl-L-DOPA-d_1_ was hydrolyzed by refluxing in 6 M DCL for 5 h to give a white solid 6-Br-L-DOPA-d_1_ **23** with 92% deuteration level. This compound can be used for ^18^F labelling though a nucleophilic substitution. The method can be extended to all other aliphatic and aromatic amino acids to give α-deuterated L-amino acids.

**Scheme 8:**
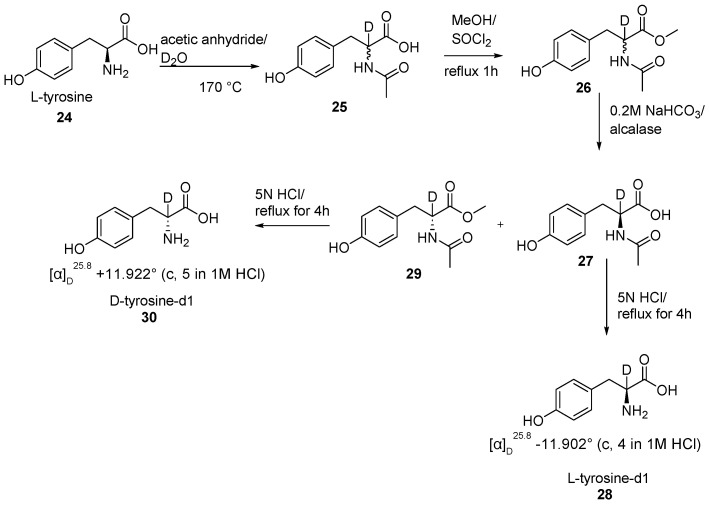
Synthesis and resolution of α-deuterated D- and L-tyrosine isomers from our laboratory.

The selectively α-deuterated L- and D-tyrosine were used as analytes to test against a chiral metal-organic framework (CMOF) chemical sensor. The chiral analyte coordinates with the open metal sites of the CMOF [59]. The dynamics of this guest are taken up into the host and can be measured using ^2^H Solid-State NMR. ^2^H Solid-State NMR has been demonstrated as an effective tool for determining the molecular dynamics of guests within short-range ordered MOFs [60]. The aim of the ^2^H solid-state NMR experiment is to observe molecular motional differences between enantiomers of deuterated tyrosine within a CMOF. The above α-deuteration through acetylation method extends to D- and L-α-deuterated tyrosine derivatives (Scheme 8). The commercially available L-tyrosine (Scheme 8)-exchangeable protons exchanged with D_2_O a couple of times by evaporation. Pre-exchanged compound **24** was heated in large excess of acetic anhydride and a small amount of monodeuterated acetic acid to give a racemic mixture of α-deuterated *N*-acetylated **25** [56]. The racemic mixture was esterified to give **26**, followed by an efficient alcalase enzymatic-selective hydrolysis to ester-hydrolyzed N-acetylated *L*-isomer **27** and unhydrolyzed D-isomer acetyl ester **29** (Scheme 8) (Supplementary Information) [57]. Acid hydrolysis of **27** gave α-deuterated L-tyrosine-d_1_ as a white solid. Unhydrolyzed D-isomer acetyl ester **29** recrystallized in ethyl acetate followed by acid hydrolysis gave α-deuterated D-tyrosine-d_1_ **30**. The enantiomeric purity of **28** and **30** is confirmed by comparing optical rotation of the protonated version of **28** and **30**, which was reported in the literature (Supplementary Information) [61].

**Scheme 9:**
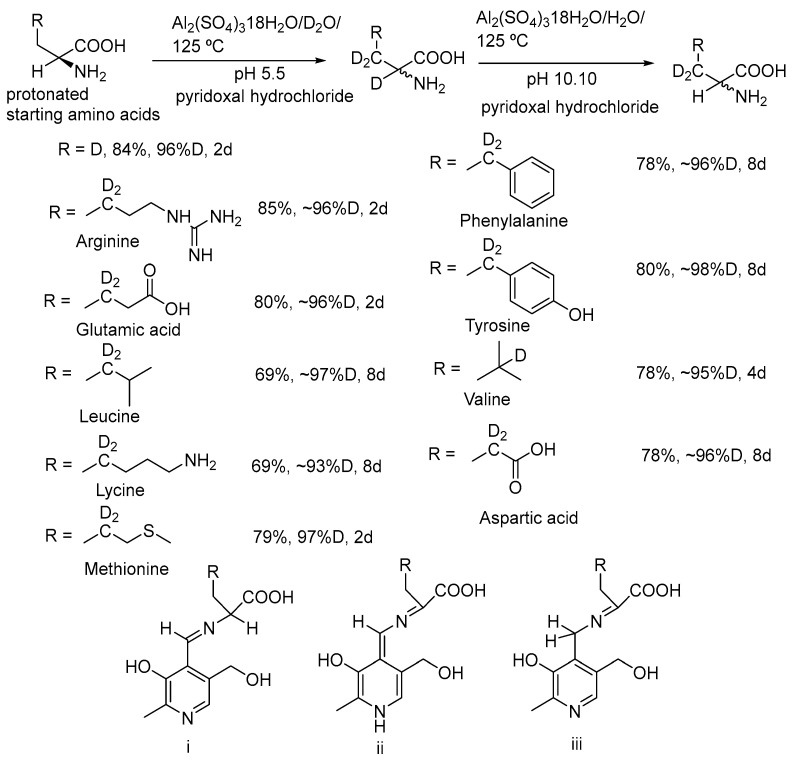
α- and β-Protons of common amino acids undergo deuteration catalyzed by aluminum(III) and pyridoxal hydrochloride through Schiff base tautomerization (**i**–**iii**).

A general procedure for the preparation of α,β-deuterium-labelled amino acids is reported by LeMaster and team [43,62]. Exchange of α,β-protons is catalyzed by AlSO_4_ and pyridoxal hydrochloride via Schiff base tautomerization (Scheme 9i–iii). This method is applied to ten commonly available amino acids successfully with high deuteration levels (Scheme 9) [42]. The amino acids are known to be reversibly transaminated and racemized by pyridoxal and Al ions. Details about the preparation of amino acids α- or β-deuterated selectevly or combined using pyridoxal and Al(III) ions and without using enzymes have been reported [42]. They clearly illustrated that selectivity between α or α,β combined is pH-dependent; therefore, the method represents a convenient and inexpensive technique for preparing selectively deuterated amino acids (Scheme 9). Further, selective β-deuteration was achieved via back-exchange of the α-position from α,β-deuterated amino acid reflux in water the above same reaction at pH 10. A couple of other papers also reported preparation of α-deuterated valine, isoleucine, and phenylalanine by treating with pyridoxal hydrochloride and NaOD [5,63]. This procedure was unsuccessful for the deuteration of cysteine, serine, threonine, histidine, and tryptophan. Functional groups interfere with Schiff’s base formation, which is essential for α,β-deuteration. A comprehensive summary of different selective and perdeuteration methods added at the end of this paper as a Table 1.

**Scheme 10:**
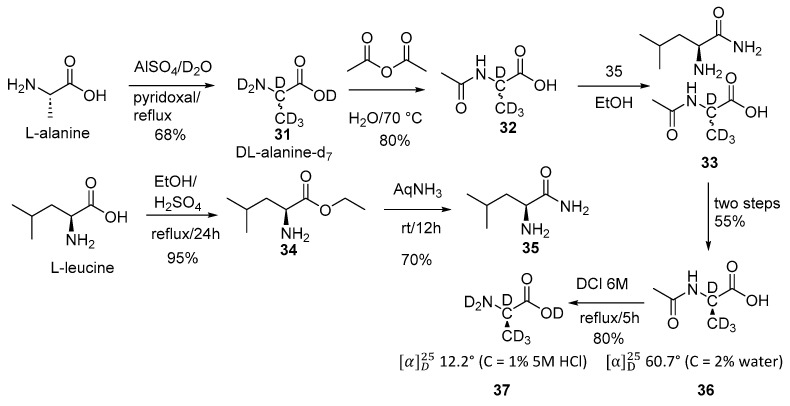
Deuteration and kinetic resolution of DL-alanine-d_7_ from our laboratory.

For our in-house deuterated protein biosynthesis efforts using *Pichia pastoris*, L-alanine-d_7_ was required as a carbon source. We successfully prepared perdeuterated DL-alanine-d_7_ **31** on a 100 g scale by treating commercially available L-alanine with AlSO_4_ and pyridoxal hydrochloride in D_2_O at reflux temperature for 24 h. After a single cycle, the overall deuteration was found to be 90% ± 1, with 97% deuteration at the α-position and 86% at the β-position (Scheme 10). Gram-scale kinetic resolution of **31** was achieved using the N-acetyl-alanine-d_4_ salt with L-leucinamide as a simple base. L-leucinamide **35** was prepared by treating L-ethyl leucinate **34** with an excess of aqueous NH_3_ (Supplementary Information) (Scheme 10). DL-Alanine-d_7_ **31** was acetylated with excess acetic anhydride in D_2_O at 70 °C. Mole equivalents of N-Acetyl-DL-alanine-d_4_ (**32**) and L-leucinamide were dissolved in ethanol at 50 °C. After being kept overnight at room temperature, separated needle crystals were filtered, washed with a small amount of ethanol and dried in vacuo to give salt of L-leucinamide and *N*-acetyl-L-alanine-d_4_ **33** in good yield [64]. The salt was showing an optical rotation of $[{\alpha ]}_{D}^{25}$ −8.96° (C = 2, water) reference $[{\alpha ]}_{D}^{25}$ −10.5° (C = 2, water). *N*-acetyl-L-alanine-d_4_ was separated from its salt by passing through an Amberlite-IR−120 column to give a white solid; N-acetyl-L-alanine-d_4_ showed an optical rotation of $[{\alpha ]}_{D}^{25}$ 60.7° (C = 2, water) reference $[{\alpha ]}_{D}^{25}$ 63.6° (C = 2, water) (Scheme 10). N-acetyl-L-alanine-d_4_ was hydrolyzed in 6 M DCl (deuterium chloric acid) reflux for 5 h to give a white solid **37**. Optical rotation for the resolved L-alanine-d_7_ **37** $[{\alpha ]}_{D}^{25}$ 12.2° (C = 1, 5 M HCl) reference $[{\alpha ]}_{D}^{25}$ 14.2° (C = 1, 5 M HCl) (Scheme 10).

**Scheme 11:**
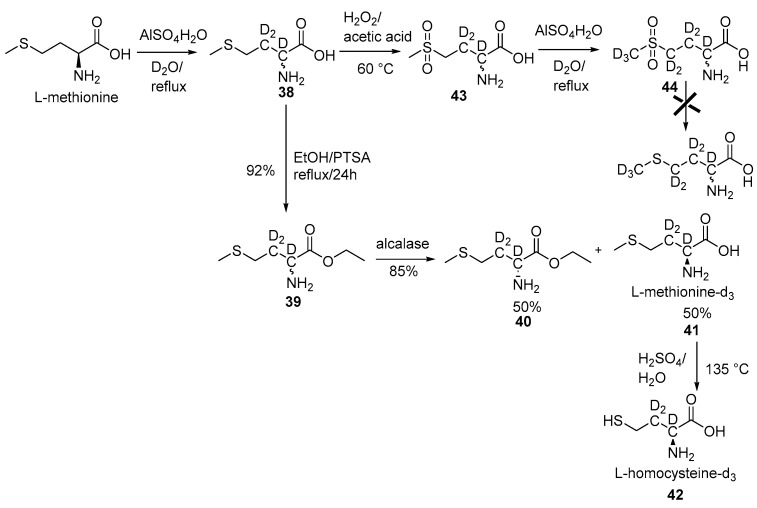
Deuteration and resolution of DL-methionine and synthesis of L-homocystein-d_3_ (**42**): results from our laboratory.

As part of an in-house project to biosynthesize and characterize mutant GPCR (G-protein-coupled receptor) suitable for X-ray crystallography and NMR studies, deuterated L-methionine and L-homocysteine were required. Initially, exchange of α,β-protons of L-methionine was achieved with a 91% D level using AlSO_4_ and pyridoxal HCl, yielding racemized DL-methionine-d_3_ **38**. To achieve perdeuteration across the entire methionine molecule, the sulfur in compound **38** was oxidized with hydrogen peroxide in acetic acid to give dioxidized sulfone **43**. This sulfone moiety activated the adjacent methyl and methylene groups, facilitating perdeuteration to yield product **44** with a 90% D level (Scheme 11) (Supplementary Information). Unfortunately, attempts to reduce the sulfone back to give perdeuterated DL-methionine-d_8_ were unsuccessful. Therefore, compound **38** was used to proceed further to obtain L-methionine-d_3_ and L-homocysteine-d_3_. The racemic mixture **38** was esterified and subsequently subjected to an efficient alcalase enzymatic selective hydrolysis of only the L-isomer, which gave **41**, leaving **40** unhydrolyzed as an ester (Scheme 11). The optical rotation for the resolved L-methionine-d_3_ **41** was measured as 17.09° (C = 1, 1 M HCl), compared to a reference of 23.1° (C = 1, 1 M HCl). L-methionine-d_3_ **41** was then demethylated with sulfuric acid to yield L-homocysteine-d_3_ **42** (Scheme 11) without loss of its stereochemistry. The optical rotation for the resolved L-homocysteine-d3 **42** was $[{\alpha ]}_{D}^{25}$ 20.06° (C = 1, 1 M HCl) reference $[{\alpha ]}_{D}^{25}$ 25.0° (C = 1, 1 M HCl) (Supplementary Information).

### 2.2. Selective and Perdeuteration of Aromatic or Heterocyclic Amino Acids

**Scheme 12:**
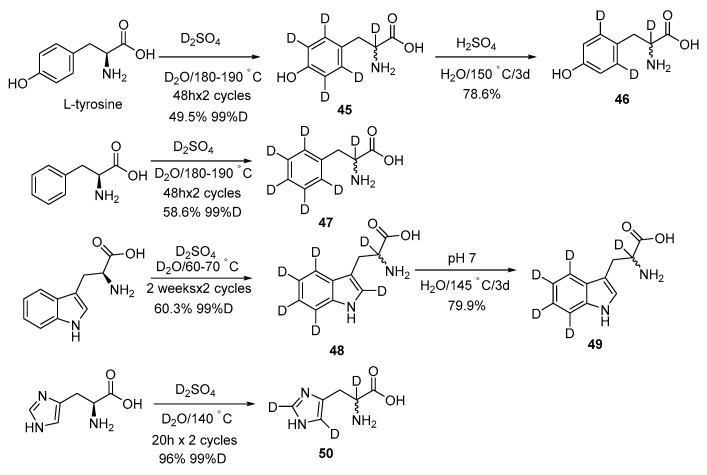
Selective deuteration of tyrosine, phenylalanine, tryptophan, and histidine using D_2_SO_4_.

A straightforward, inexpensive, and large-scale procedure for the selective deuteration of phenylalanine, tyrosine, tryptophan, and histidine has been reported, making them suitable for incorporation into bacterial proteins (Scheme 12) [21]. This method greatly facilitates the use of selective deuteration in detailed ^1^H NMR studies for elucidating protein structure and function. In this procedure, amino acids were treated with deuterated sulfuric acid to yield selectively deuterated racemic mixtures. To achieve high deuteration levels, the exchangeable N-H and O-H protons in the four amino acids were first exchanged with deuterium oxide prior to the main deuteration. Tyrosine was heated at 180–190 °C in 50% D_2_SO_4_ in D_2_O for 48 h. Two cycles of this treatment yielded α,2,3,5,6-pentadeuterated tyrosine **45** with 99% deuterium levels and a 50% yield. Tri-α,2,6-deuterated tyrosine **46** was subsequently prepared from α,2,3,5,6-pentadeuterated tyrosine **45** by heating at 150 °C for 3 days in water with sufficient sulfuric acid to dissolve the tyrosine (Scheme 12). It was observed that tyrosine tended to decompose at higher temperatures, with 190 °C being determined as the optimum temperature for the exchange. When phenylalanine was heated under the same conditions for two cycles, the reaction yielded α,2,3,4,5,6-hexadeuterated phenylalanine **47** with 99% deuterium levels and yields around 60% (Scheme 12) [21]. The use of D_2_SO_4_, due to its toxicity and corrosive nature, must be carried out under strict safety precautions.

In tryptophan, a complete exchange of all aromatic protons for deuterium was achieved by maintaining a solution of tryptophan in approximately 9% D_2_SO_4_ in D_2_O at 60–70 °C for two weeks. Conversely, α,4,5,6,7-pentadeuterated tryptophan was prepared via back exchange at the C2 indole position from α,2,4,5,6,7-hexadeuterated tryptophan **49** by heating in water at pH 7 and 145 °C for 3 days. The acid labile nature of tryptophan limits the concentrations of D_2_SO_4_ that can be employed. For histidine, the trideutero compound **50** can be obtained by heating at 140 °C in D_2_O with sufficient D_2_SO_4_ to dissolve the histidine for 20 h. After two cycles, this process yielded racemic histidine with 99%D levels and a 96% yield (Scheme 12).

**Scheme 13:**
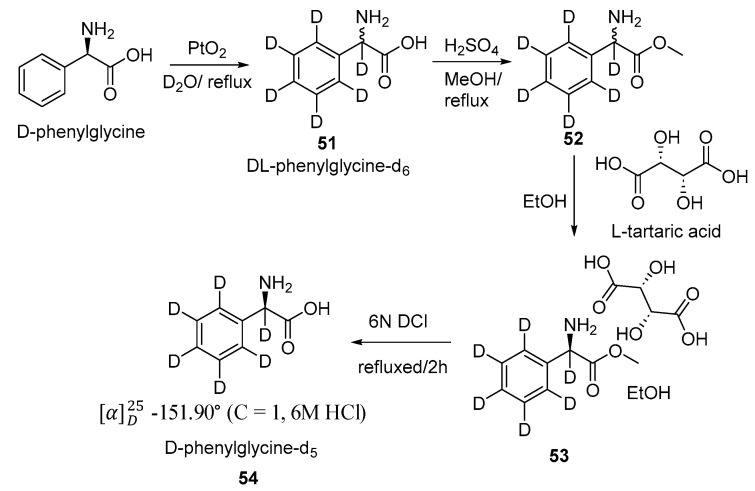
Deuteration and kinetic resolution of phenylglycine-d_6_.

As part of an internal project to synthesize ampicillin with a selectively deuterated phenylglycine moiety from 6-aminopenicillanic acid (6-APA), we needed perdeuterated D-phenylglycine-d_6_. We started with commercially available D-phenylglycine, which was heated to reflux in D_2_O with reduced PtO_2_ (Adam’s catalyst) (pre-treated with NaBH_4_). Reduced PtO_2_ refers to the process of converting platinum dioxide (PtO_2_) to metallic platinum (Pt). This transformation is crucial because PtO_2_ itself is not catalytically active, but it readily reduces to highly dispersed, catalytically active platinum metal under conditions like exposure to hydrogen or certain reducing agents (caution must be exercised when using PtO_2_, as it is a strong oxidizing agent). This yielded a racemic mixture of DL-phenylglycine-d_6_ **51** in high yield with 96% deuteration (Scheme 13) (Supplementary Information). The racemic mixture **51** was then esterified in acidic ethanol and subjected to an efficient kinetic resolution with (+)-tartaric acid, yielding the tartrate salt of D-ester-d_6_ **53**. Specifically, the racemic ethyl ester **52** was resolved with 1 molar equivalent of (+)-tartaric acid in aqueous alcohol, providing the D-ester hydrogen (+)-tartrate salts **53** in 41% yield (or 82% based on the D-component) (Scheme 13) (Supplementary Information). Finally, enantiomerically pure D-phenylglycine-d_6_ **54** was obtained upon DCl hydrolysis of **53**. Its optical rotation was confirmed by comparing it to commercially available D-phenylglycine ($[{\alpha ]}_{D}^{25}$ −151.90° (C = 1, 6 M HCl) reference $[{\alpha ]}_{D}^{20}$ −155.0° (C = 1, 1 M HCl) Sigma Aldrich Australia).

**Scheme 14:**
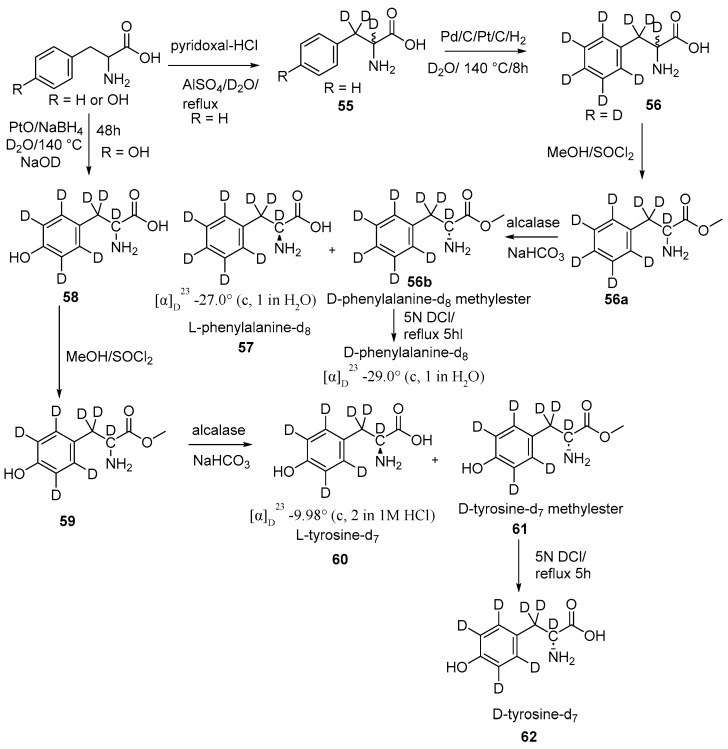
Deuteration and resolution of DL-Phenylalanine-d_8_ and DL-tyrosine-d_7_.

**Scheme 15:**
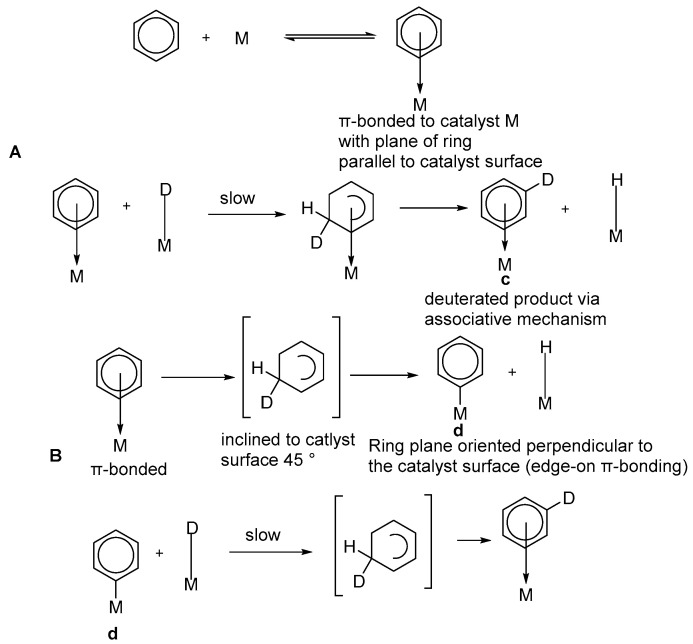
Associative (**A**) and dissociative mechanism (**B**) of the heterogeneous H/D exchange in aromatic substrates.

Perdeuterated ^10^B-boronophenylalanine-d_7_ (^10^BPA) is being developed for Neutron Capture Enhanced Particle Therapy (NCEPT), with the expectation that its perdeuteration could significantly enhance this treatment. This is due to the vastly different neutron scattering properties of hydrogen (^1^H) and deuterium (^2^H or D), allowing for substantial enhancement of a material’s neutron contrast through molecular deuteration. This can increase neutron contrast in tissues, which is helpful in therapies and imaging involving neutron capture. If hydrogens replace in a molecule with deuteriums, the way neutrons interact with that molecule changes a lot. As part of this effort, perdeuterated tyrosine-d_7_ and phenylalanine-d_8_ are crucial requirements. To prepare these, commercially available L-tyrosine and L-phenylalanine were separately refluxed in D_2_O with reduced PtO_2_ (Adam catalyst) (caution must be exercised when using PtO_2_, as it is a strong oxidizing agent) (pre-treated with NaBH_4_). This process yielded a racemic mixture of DL-tyrosine-d_7_ **58** in moderate yields (Scheme 14). However, phenylalanine degraded under these conditions, resulting in a very low yield of DL-phenylalanine-d_8_ **56** (approximately 10%). The racemic mixture **58** was then esterified in acidic methanol, followed by efficient enzymatic resolution with alcalase at pH 8, to successfully obtain L-tyrosine-d_7_ **60**. Acid hydrolysis of the unhydrolyzed D-tyrosine-d_7_ ester **61** yielded enantiomerically pure D-tyrosine-d_7_ **62**. Phenylalanine was perdeuterated using two different methods. Initially, the exchange of α,β-protons of L-phenylalanine was achieved using AlSO_4_ and pyridoxal HCl, which produced racemized DL-phenylalanine-d_3_ **55**. The aromatic ring was then deuterated with a high yield and 90%D level by refluxing in D_2_O with 10% *w*/*w* Pd/C/Pt/C (1:1) (Scheme 14). After bubbling with H_2_ gas, the heterogeneous mixture was refluxed at 140 °C for 8 h. The resulting racemic mixture DL-phenylalanine-d_8_ **56** was also esterified in acidic methanol, followed by enzymatic resolution with alcalase at pH 8, to give L-phenylalanine-d_8_ **57** (Scheme 14), (Supplementary Information). Prior to acid hydrolysis, both the unhydrolyzed D-phenylalanine methyl ester and D-tyrosine methyl ester were recrystallized from ethyl acetate (Supplementary Information). All the D and L enantiomers were confirmed by comparing their respective optical rotations with commercially available protonated versions. The mechanism of aromatic H/D exchange proceeds via heterogeneous catalysis. Garnett and colleagues proposed that a π-complex mechanism is involved in the heterogeneous catalysis of H/D exchange [65] (Scheme 15). Kinetic studies revealed that, alongside the associative mechanism A, a competing dissociative π-complex mechanism B also played a role. The key distinction between these two reaction pathways lies in the substitution process: in the associative mechanism A, a hydrogen atom is directly replaced by a deuterium atom coordinated to the metal center (Scheme 15). In contrast, the dissociative mechanism B involves substitution of a proton from the initially formed π-complex by the metal atom [18,65]. The intermediate phenyl radical **d** is subsequently formed. It is only in the second step that the metal atom is replaced by a deuterium atom, leading to the formation of the final product **c**. For platinum, the dissociative mechanism is believed to play a more prominent role.

### 2.3. Specific Deuteration with Retention of Stereochemistry

**Figure 2:**
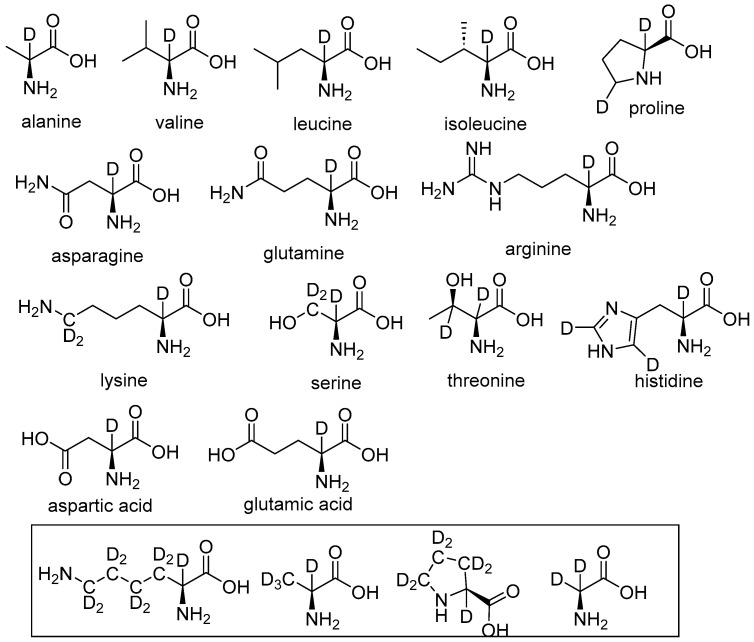
Enantiospecific α-deuteration of common amino acids was performed with RuNP@PVP nanoparticles and D_2_ gas at 55 °C, while perdeuteration (>95%) of lysine, alanine, proline, and glycine was obtained by heating in D_2_O with H_2_ using Ru/NPC-600 at 100 °C.

Many existing methods for α-, β-, or α,β-deuteration of amino acids often lead to a loss of optical activity at the chiral α-carbon [66]. To avoid this, deuteration typically needs to be performed either on the intact molecule or by synthesizing the molecule from deuterated precursors. Celine Taglang and colleagues, however, reported a method for the selective and enantiospecific α-deuteration of several common amino acids and their derivatives [67]. This was achieved through enantiospecific C-H activation using RuNP@PVP nanoparticles as a catalyst and D_2_ gas at 55 °C (Figure 2). Their mechanistic studies suggest that the selectivity for the α-position of the directing heteroatom stems from a four-membered dimetallacycle, which acts as a key intermediate. For serine and threonine, additional C-H activation occurred at the β-carbon, with both cases retaining stereoselectivity. Notably, in threonine, α,β-deuteration proceeded with full retention of configuration for both chiral centers (Figure 2). This method demonstrated high regioselectivity and full enantiospecificity across various solvents, including D_2_O, THF, and DMF. This enantiospecific C-H activation was also successfully extended to biologically active peptides, where similar deuterium incorporation was observed. More recently, a significant phytic acid-modulated Ru (Ru/NPC-600) catalyst was developed for the regioselective deuteration of amines, diamines, and amino acids [68]. While Ru/NPC-600 still achieves over 95% perdeuteration on alanine, proline, glycine, and lysine, the authors focused on the regioselective deuteration of these compounds and did not explicitly mention the stereoselectivity at the α-carbon [68].

**Scheme 16:**
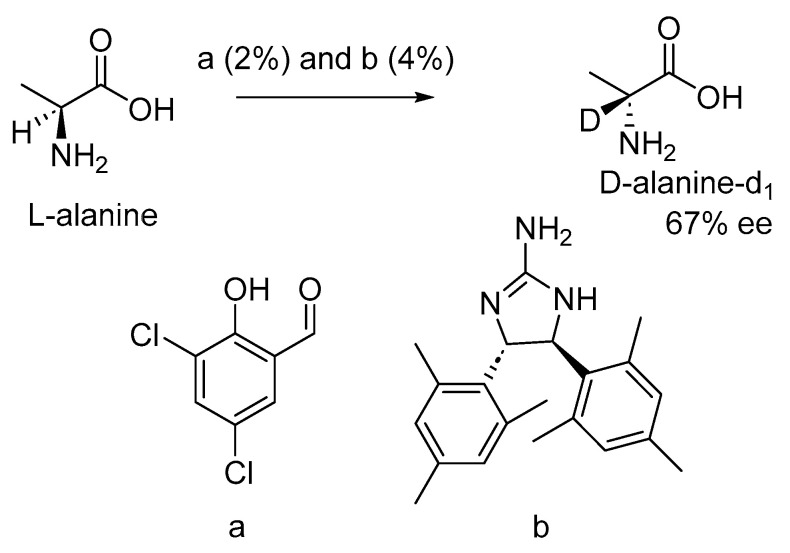
Catalytic selective deuteration of L-alanine to α-deuterated D-alanine using dichloropyridoxal (**a**) and a chiral base (**b**).

A new method has been reported for the stereoselective α-deuteration of L-alanine to yield deuterated D-alanine with an inversion of stereochemistry, all under milder reaction conditions (25 °C) [69]. This catalytic deuteration utilizes a combination of an achiral dichloropyridoxal analogue and a chiral base, notably without requiring the protection of amine and acid groups (Scheme 16). The versatility of this method also allows for the catalytic deuteration of D-alanine with retention of stereochemistry, resulting in deuterated D-alanine. Furthermore, the researchers demonstrated that a racemic mixture of alanine can be catalytically deuterated to produce an enantiomeric excess of deuterated D-alanine [69].

**Scheme 17:**
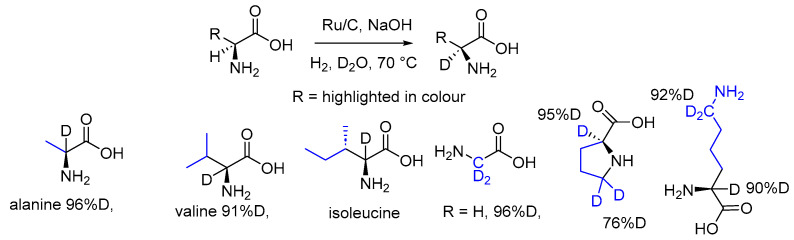
Ruthenium-catalyzed stereoselective α-deuteration of L-alanine and other amino acids.

Alessia Michelotti and colleagues have reported a scalable method for the stereoselective deuteration of amino acids using 5% Ru/C under basic conditions in D_2_O [70]. Under optimal conditions—10% catalyst loading, 3 mol equivalents of NaOH in D_2_O under an H_2_ atmosphere at 70 °C for 12 h—they achieved 99%D levels on L-alanine (Scheme 17). High α-deuteration was also achieved for other unfunctionalized amino acids such as glycine, leucine, and valine. For proline and lysine, deuteration was observed at all positions adjacent to the amine group. Notably, platinum and palladium on carbon catalysts were found to be ineffective for this H/D exchange on L-alanine, a finding consistent with other reported literature methods. The optimized reaction conditions involved using 1 mol of substrate with 3 mol of NaOH and 10% *w*/*w* Ru/C (5% Ru on carbon) in D_2_O, heated at 90 °C under 1 atm of H_2_. These conditions resulted in approximately 95% deuterium incorporation at the α-carbon without any loss of stereochemical configuration.

**Scheme 18:**
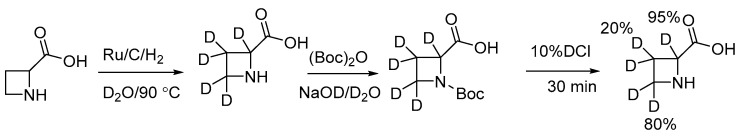
This work from our laboratory describes a ruthenium-catalyzed, stereoselective incorporation of deuterium into azetidine.

Azetidine-2-carboxylic acid (AZE) is a non-protein amino acid (NPAA) and a toxic metabolite found in several plant species, including members of the *Beta vulgaris* group like sugar beet and garden beet. As an analog of proline, AZE can be mistakenly incorporated into proteins during synthesis. Fodder beets present a potential pathway for AZE to enter the human food chain. To investigate whether the fodder beet cultivar group of *Beta vulgaris* produces AZE, we used deuterated AZE-d_5_ in a mass spectrometry experiment. In our lab, the four-membered cyclic AZE was deuterated under Ru/C/H_2_-mediated hydrothermal conditions. While this method yielded AZE-d_5_, the yield was low (50%), and the deuteration level was only 60%D after two cycles. For purification, the final product was converted into its *N*-Boc derivative, which then underwent acid hydrolysis to give pure AZE-d_5_ as an HCl salt (Scheme 18) (Supplementary Information). Site-specific deuteration levels were determined using a previously reported formula [71], and overall deuteration levels were calculated with DGet! Software [72].

**Scheme 19:**
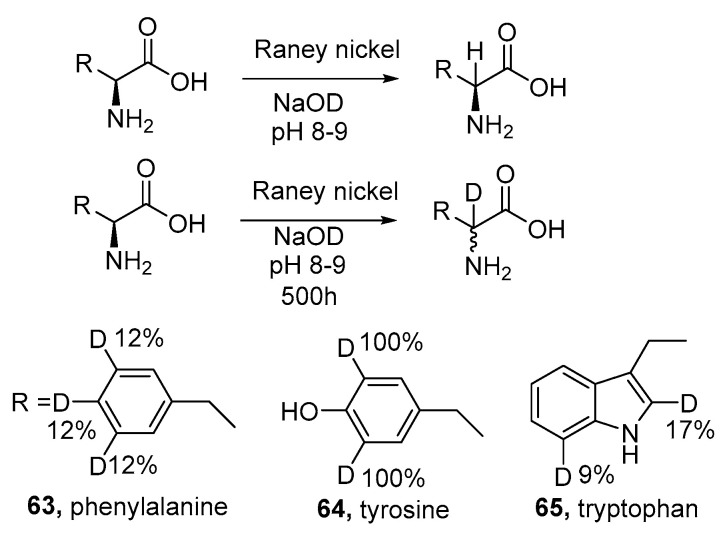
Raney nickel-catalyzed selective deuteration of phenylalanine, tyrosine, and tryptophan in basic media.

Robert C. Woodworth and colleagues developed a catalytic method for the selective deuteration of aromatic amino acids under mild conditions. Their approach utilized Raney nickel as a catalyst, employed under either acidic or basic conditions at room temperature [66]. They successfully used this catalyst to prepare selectively deuterated derivatives of phenylalanine **63**, tyrosine **64**, and tryptophan **65**, suggesting the potential to extend this method to other amino acids. Deuteration was observed not only on the aromatic rings but also on the aliphatic chains of all three amino acids. Interestingly, while other positions on the rings of phenylalanine and tyrosine showed significant deuteration, there was no substantial deuteration at the *ortho*-to-methylene positions. In the heterocyclic ring of tryptophan, only the 7′ and 2′ positions were significantly deuterated **65** (Scheme 19). The authors also noted that the overall optical activity remained high for all three amino acids. However, exchange at the α-proton was slow, requiring 500 h and accompanied by racemization.

**Scheme 20:**
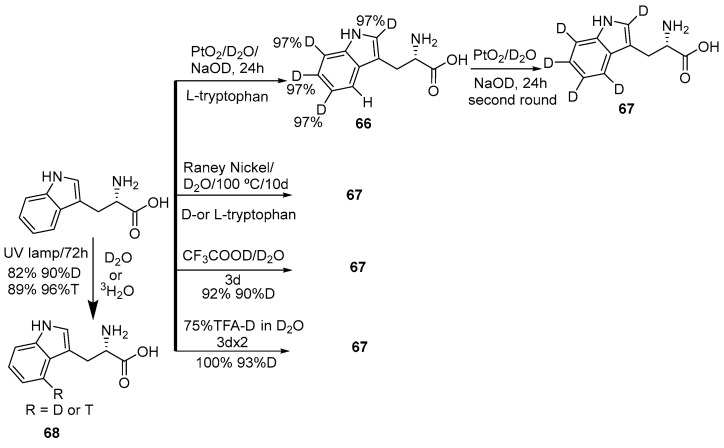
Deuteration of the tryptophan heterocyclic ring with retention of α-carbon stereochemistry.

The heterocyclic ring in tryptophan has been successfully deuterated with retention of stereochemistry at the α-carbon using four distinct sets of conditions (Scheme 19). One method involved using a homogenous activated Adam’s catalyst (PtO_2_) for hydrothermal reflux in D_2_O for 24 h, yielding tryptophan (2′,5′,6′,7′-d_4_) **66** [4]. Further deuteration to obtain tryptophan (2′,4′,5′,6′,7′-d_5_) **67** was achieved by subjecting **66** to a second 24 h exchange round (Scheme 20).

Both R- and S-tryptophan were also deuterated to give R- and S-tryptophan (2′,4′,5′,6′,7′-d_5_) by heating them in Raney/nickel/D_2_O at 100 °C for 10 days [34]. Winnicka and colleagues also reported an acidic condition for L-tryptophan indole ring deuteration, involving stirring at room temperature in CF_3_COOD/D_2_O for 3 days to produce S-tryptophan (2′,4′,5′,6′,7′-d_5_) **67** (Scheme 20) [51]. After three cycles of exchange, 93% deuterium incorporation was reported on the tryptophan ring. Randall and Mathew and their team also reported the deuteration of all protons of the heterocyclic ring of L-tryptophan **67** using trifluoroacetic acid in 25% D_2_O, crucially without losing optical activity at the α-carbon [51]. Two rounds of treatment, each lasting three days, resulted in L-tryptophan-d_5_ with 100% recovery and 93% deuteration. Selective deuteration at the 4′-position of L-tryptophan was achieved to give **68** by subjecting it to UV light for 72 h. When L-tryptophan was treated with a mixture of D_2_O and ^3^H_2_O under irradiation conditions, it resulted in doubly 4′-labeled deuterium and tritium L-tryptophan (Scheme 20) [73].

**Scheme 21:**
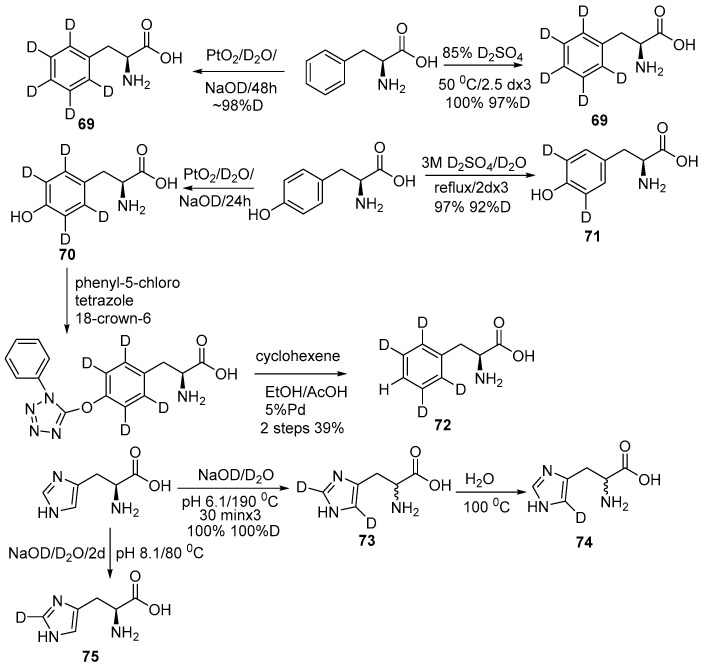
Phenylalanine and tyrosine undergo ring deuteration with α-carbon stereochemistry retained, while histidine allows for imidazole ring deuteration. At higher temperatures, α-carbon stereochemistry may be partially or fully lost during deuteration.

Activated Adam’s catalyst plays a role in preparing L-phenylalanine-d_5_ **69** and tyrosine-d_4_ **70** under basic conditions (Scheme 21) [4]. This L-tyrosine-d_4_ was then converted to selectively protonated L-phenylalanine-d_4_ **72** by treating it with 1-phenyl-5-chlorotetrazole, followed by heating with 5% Pd/C [4]. The aromatic ring of L-phenylalanine was deuterated to yield L-phenylalanine-d_5_ using 85% D_2_SO_4_ heated at 50 °C for 2.5 days [51]. This process achieved up to 90%D ring deuteration without any loss of stereochemistry. L-Tyrosine-d_2_ (3′,5′) **71** was also prepared from L-tyrosine by heating it in 20% D_2_SO_4_ in D_2_O for over 2 days (Scheme 21). For L-histidine, ring deuteration occurred at 190 °C in NaOD/D_2_O (pH 6), resulting in DL-histidine-d_2_ **73**. Similarly, when compound **73** was heated at 80 °C in NaOH/H_2_O (pH 8), it produced DL-histidine-d_1_ (2′) **74**. L-Histidine-d_1_ **75** can be prepared from L-histidine by heating it in basic D_2_O at 80 °C for 2 days. In all three amino acids, acid- or base-mediated deuteration is achieved through an equilibration mechanism. To reach the desired deuteration level without losing stereochemistry at the α-carbon, the reaction was repeated three times for each case [51].

**Scheme 22:**
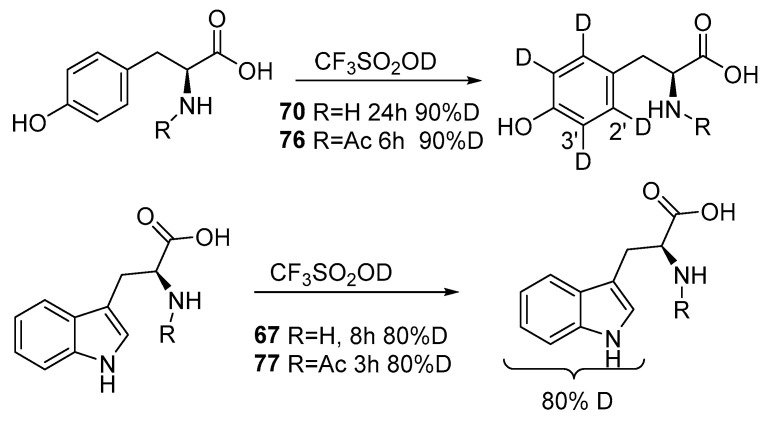
Lei Wang et al. reported ring deuteration of L-tyrosine and L-tryptophan with retention of α-carbon stereochemistry.

Lei Wang and colleagues reported a straightforward method for the aromatic ring deuteration of L-tyrosine and L-tryptophan without affecting the stereochemistry at the α-carbon [10]. The exchange was carried out at room temperature using deuterated triflic acid (Scheme 22). The researchers also attempted the exchange on L-tyrosine and N-acetylated L-tyrosine. They observed that a 90% H/D exchange occurred at the 2′ and 3′ positions of L-tyrosin**e 70** within 24 h at room temperature (Scheme 22). Crucially, the exchange rate significantly improved when using N-acetylated L-tyrosine. Over 90% exchange was achieved within 6 h at the 3- and 2-positions, respectively, for compound **76** (Scheme 22). Similar trends were observed for L-tryptophan derivatives **67** and **77** [10]. The H/D exchange for the aromatic protons of L-tryptophan reached up to 80% at room temperature. While unprotected compound **70** took over 8 h, N-Ac-L-tryptophan **77** completed the exchange in less than 3 h (Scheme 22) [10]. These results clearly indicate that the exchange rate for N-acetyl-protected aromatic α-amino acids is faster than that for unprotected compounds. Importantly, the exchange occurred without any racemization at the α-carbon.

**Scheme 23:**
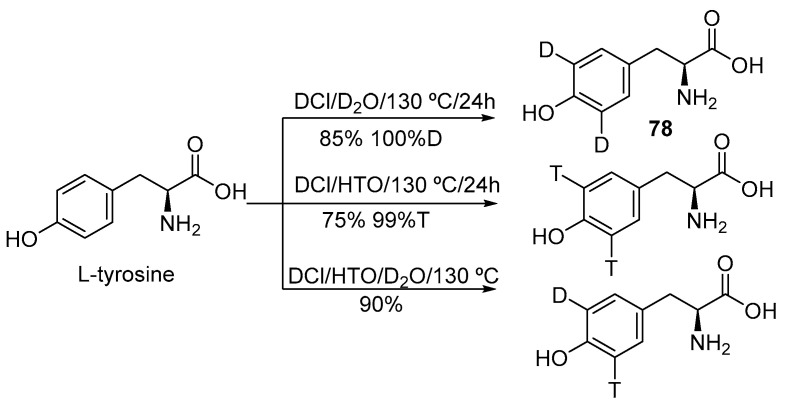
Selective ring deuteration at 3′,5′-positions of L-tyrosine.

L-Tyrosine, a precursor of L-DOPA, plays an essential role in a variety of metabolic processes. The enzyme tyrosinase, classified as an oxidoreductase, catalyzes two sequential oxidation reactions: first, the conversion of L-tyrosine to L-DOPA, and then the oxidation of L-DOPA to dopaquinone. Kanska et al. investigated the deuterium kinetic isotope effect (KIE) in the tyrosinase-catalyzed hydroxylation of L-tyrosine-d_2_ (3′,5′-positions) to produce L-DOPA. For these kinetic studies, selective ring deuteration of L-tyrosine at the 3′ and 5′ positions was successfully achieved with nearly 100% deuterium incorporation (Scheme 23) [74].

### 2.4. Metal Mediated Heterogenous Conditions Selective Direct Deuteration

**Scheme 24:**
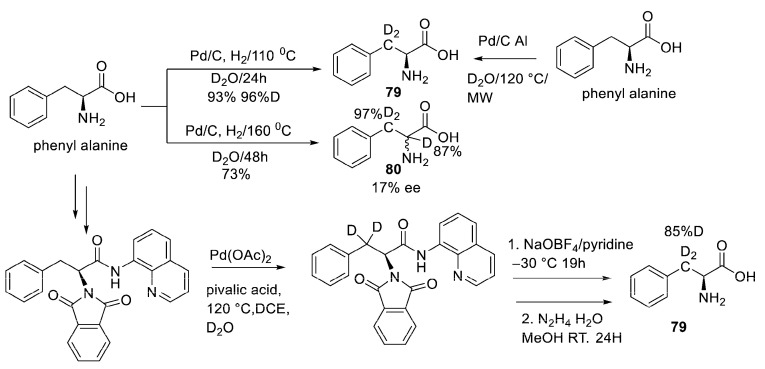
Selective β-deuteration of phenylalanine via metal-mediated heterogeneous catalysis and Pd(OAc)_2_-catalyzed β-deuteration of its N-protected phenylalanine aminoamide.

Kokel and colleagues have developed an environmentally benign method for the selective deuteration of amino acids and synthetic building blocks using a Pd/C-Al-D_2_O catalytic system. This approach is particularly valuable given the growing importance of isotope-labeled compounds in medicinal chemistry and structural biology, where they serve as enhanced drug candidates and crucial biological probes. The core of their method lies in selective H-D exchange reactions that utilize simple D_2_O as the deuterium source. D_2_ gas is generated in situ from the reaction of aluminum and D_2_O, while a commercially available palladium catalyst facilitates the H-D exchange. The procedure’s high selectivity and efficiency, coupled with its simplicity and safety, establish it as an environmentally friendly alternative to existing methods (Scheme 24) [33]. Sajiki and coworkers also reported an efficient and selective method for the α,β-deuteration of phenylalanine derivatives [75]. They observed that at 110 °C, Pd/C-mediated exchange in D_2_O exclusively occurred at the β-position with retention of stereochemistry, yielding compound **79** (Scheme 24). However, increasing the temperature to 160 °C extended the exchange to both α- and β-position, albeit with a loss of stereochemistry, resulting in racemic product **80** (Scheme 24). The authors also noted the formation of a small amount of ring-saturated product under these conditions.

Fei-Fei Sheng and colleagues have developed a highly efficient method for synthesizing β-deuterated amino acids. Their protocol utilizes palladium diacetate-catalyzed H/D exchange from N-protected aminoamides. A key advantage of this approach is its simplicity: the β-deuterated amino acid can be obtained simply by removing the protecting groups (Scheme 24) [76]. This versatile method is not limited to a narrow scope; it can be readily extended to produce a variety of other natural and unnatural α-amino amides. The authors proposed a mechanism in which palladium acetate first reacts with the amino amide substrate, replacing the acetate group to form intermediate A. This is followed by C–H bond activation, resulting in the formation of cyclopalladium complex B with the release of a second acetic acid molecule. The liberated acetic acid undergoes H/D exchange with heavy water, generating deuterated acetic acid. This deuterated acid then assists in the cleavage of the C–Pd bond, leading to the incorporation of a deuterium atom at the β-position of the amino amide. Finally, ligand exchange regenerates palladium acetate and produces the deuterated amino amide.

**Scheme 25:**
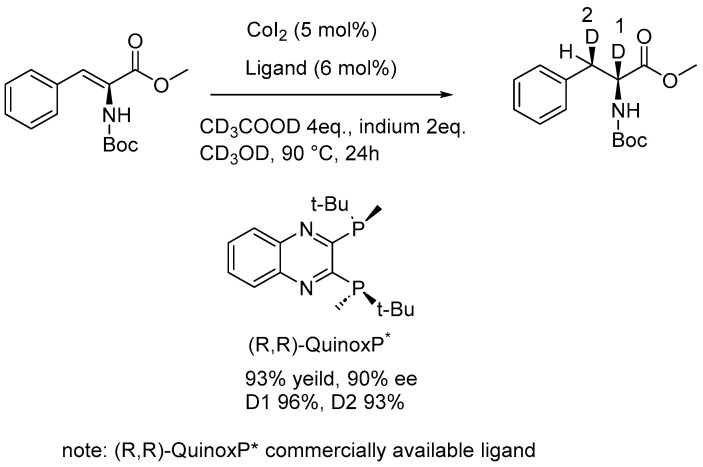
A cobalt-catalyzed enantioselective deuteration provides α,β-dideuterio-phenylalanine.

Aibo Li and his team have developed a highly efficient protocol for the enantioselective deuteration of amidoacrylates. This method, catalyzed by cobalt, yields α,β-dideuterio-α-amino esters with excellent enantiomeric ratios and almost complete deuteration (99%). A significant advantage of this approach is its use of deuterated methanol as a cost-effective deuterium source (Scheme 25) [77]. This new protocol has already been successfully applied to prepare dideuterated amino acid fragments found in various drugs. Furthermore, the stereoselective deuteration has been directly utilized in the synthesis of L-DOPA-d_2_.

**Scheme 26:**
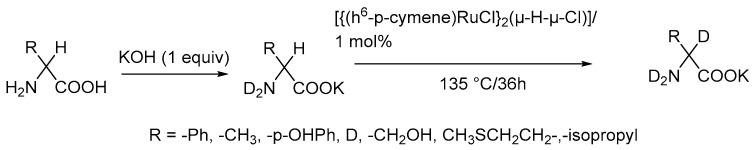
α-deuteration of amino acids using Ru complex catalyst and deuterium oxide as a deuterium source.

While efforts to deuterate L-tyrosine showed lower deuterium efficiency at the β-position, a minor amount of deuterium incorporation (17%D) was observed at the *ortho*-positions of the phenolic hydroxyl group. In related work, Basujit Chatterjee and colleagues developed an easy and stereoselective method for α-deuteration of amines and amino acids. Their technique utilizes a monohydrido-bridged dinuclear Ru complex as a catalyst and deuterium oxide (D_2_O) as the deuterium source (Scheme 26) [78].

**Scheme 27:**
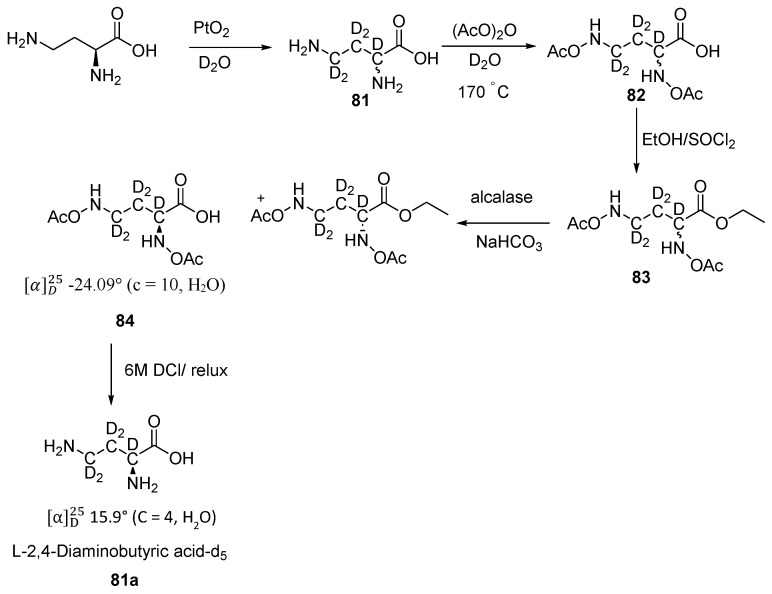
Metal mediated heterogenous deuteration of 2,4-diaminobutyric acid and resolution.

For an in-house project focused on preparing deuterated polymyxin analogues to study their structure–activity relationship on Gram-negative bacterial membranes, L-2,4-diaminobutyric acid was needed. Initially, attempts to deuterate commercially available L-2,4-diaminobutyric acid using Ru/C in D_2_O under reflux only achieved an overall deuteration level of 65%D. To improve this, L-2,4-diaminobutyric acid was then refluxed in D_2_O with reduced PtO_2_ (Adam’s catalyst) (which was pre-treated with NaBH_4_). This approach successfully yielded a racemic mixture of DL-2,4-diaminobutyric acid-d_5_ **81** with approximately 94%D level in quantitative yield (Scheme 27) (Supplementary Information). The racemic mixture **81** was subsequently diacetylated by heating it in acetic anhydride and D_2_O to produce **82**. This diacetylated product was then purified using flash column chromatography. The racemic diacetylated mixture **82** was esterified in acidic ethanol, followed by an efficient enzymatic resolution with alcalase at pH 8 to give the L-diacylated product. The unhydrolyzed product was extracted using ethyl acetate. Finally, enantiomerically pure L-2,4-diaminobutyric acid-d_5_ **81a** was obtained after hydrolysis of **82** with 6 M DCl (deuterium HCl).

## 3. Deuteration by Chemical Synthesis

**Scheme 28:**
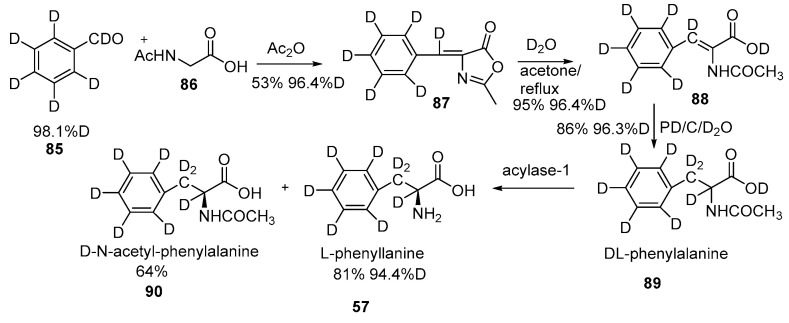
Synthesis of perdeuterated N-acetyl-DL-phenylalanine and L-phenylalanine **57**.

**Scheme 29:**
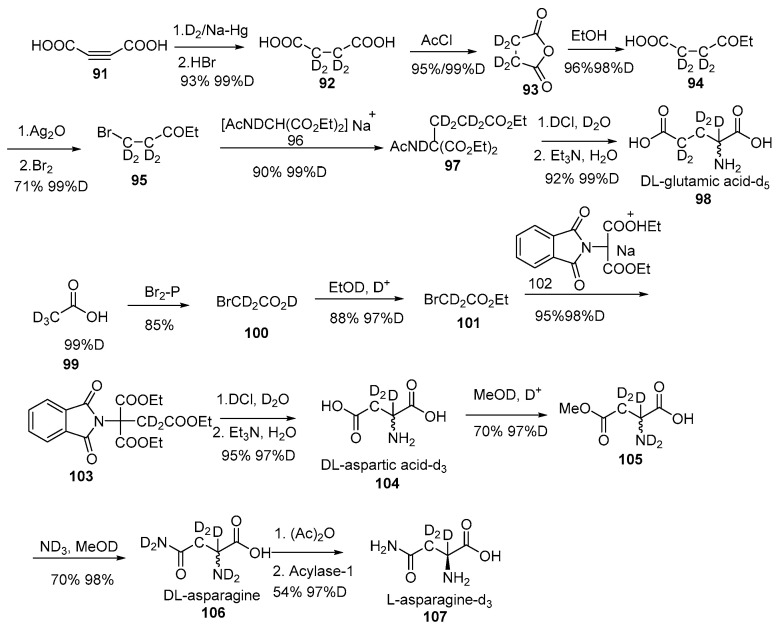
Synthesis of perdeuterated DL-glutamic acid, DL-aspartic acid, DL-asparagine, and L-asparagine.

In the early 1960s, researchers successfully synthesized fully deuterated L-phenylalanine **57**, glutamic acid, aspartic acid, and asparagine. These were initially obtained as their DL N-acetylated mixtures, with the L-amino acids then resolved using the acylase-1 enzyme (Scheme 28) [79,80]. Phenylalanine, for instance, was synthesized from benzaldehyde-d_6_ **85** and N-acetyl-glycine **86** (Scheme 28) [79]. This particular approach was not a convergent method, meaning each amino acid was synthesized individually. For example, glutamic acid-d_5_ was synthesized from acylamido malonic ester and acetylene dicarboxylic acid. Similarly, asparagine-d_3_ was prepared from phthalidomide and malonic ester by treating them with an appropriate alkyl halide (Scheme 29). In all these cases, the resulting DL-amino acids were resolved into their L-amino acid forms using the acylase-1 enzyme.

**Scheme 30:**
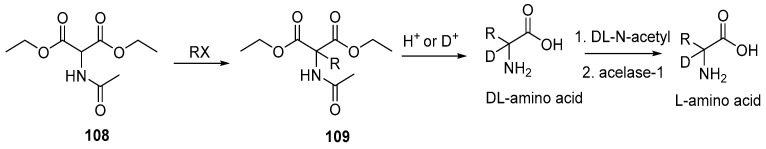
Laboratory preparation of perdeuterated DL-amino acids.

One of the most straightforward and convergent laboratory routes for synthesizing DL-amino acids involves starting from commercially available acylamino malonic ester **108** (Scheme 30) [40,80]. This method begins by generating a carbanion from the acylamino malonic ester in a basic medium, such as ethanolic sodium ethoxide [80]. This carbanion then acts as a nucleophile, displacing the halide from any given alkyl halide. The resulting intermediate, **109**, is typically hydrolyzed, deacylated, and decarboxylated in a single step by boiling it in either deuterated or protonated acid, which produces the desired DL-amino acid [40,80]. If the goal is specifically to create α-deuterated amino acids, the final step would involve boiling the intermediate in a deuterated acid environment. Furthermore, if RX (the alkyl halide) is a fully deuterated precursor, the resultant product will be a fully deuterated DL-amino acid.

### Deuteration by Stereoselective Chemical Synthesis

**Scheme 31:**
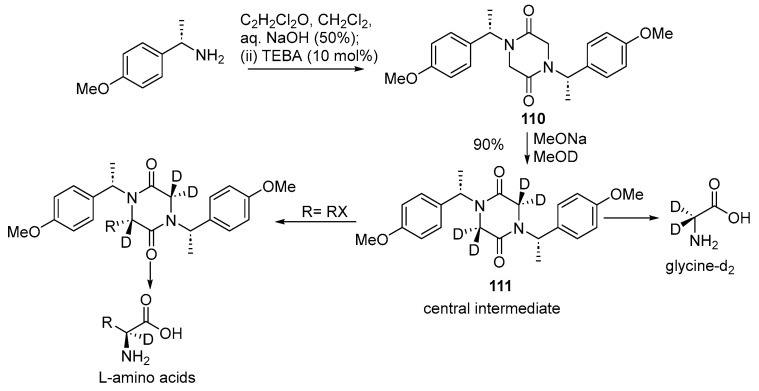
Stereoselective synthesis of α-deuterated amino acids using chiral auxiliary diketopiperazine **110**.

A novel approach for the stereoselective synthesis of α-deuterated amino acids starts with the chiral auxiliary diketopiperazine **110**. Diketopiperazine templates like **110** are effective building blocks for chiral amino acid synthesis because they are easily monofunctionalized and can be hydrolyzed to their corresponding amino acids (Scheme 31) [81]. Efficient mono-alkylation of this chiral template using the desired electrophile successfully yields the (S)-substituted adducts (Scheme 31). While initially reported for α-deuterated amino acids, this method can be extended to produce fully deuterated amino acids while maintaining stereoselectivity. Introducing two deuterium atoms on each side can be achieved by generating a carbanion at the active methylene group on the chiral auxiliary **110** (Scheme 31), which is then quenched with D_2_O to form the central intermediate **111** [82]. Subsequently, introducing an R alkyl group (RX), including different deuterated alkyl halides, yields the desired fully deuterated or selective α-L-deuterated amino acids. This method has been applied to produce L-phenylalanine, L-tyrosine, L-alanine, glycine, L-leucine, L-isoleucine, L-valine, and L-tryptophan [81]. The authors note that the nature of the electrophile plays a significant role in the observed high stereochemical excess. Specifically, the bulkiness of the incoming electrophile is crucial. For instance, no selectivity was observed when methyl iodide was used as the electrophile; however, the resulting 50:50 mixture of isomers was easily separated by column chromatography.

**Scheme 32:**
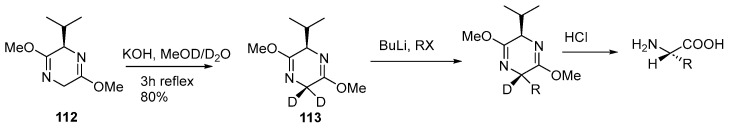
A stereoselective approach to synthesizing α-deuterated amino acids using a central scaffold.

Rose and colleagues developed another valuable chiral auxiliary: (3S)-isopropyl-2,5-dimethoxy-dihydropyrazine **112**. When subjected to base-catalyzed deuteration in MeOD/D_2_O, this compound yields **113** in excellent yield, crucially without altering the stereocenter at C3 (Scheme 32) [83,84]. This auxiliary allows for the selective alkylation at C3 via a butyllithium-generated carbanion, providing a diverse range of enantiomerically pure (S) and (R) amino acids (Scheme 32). Here, RX represents various protonated or deuterated alkyl halides. A significant advantage of (3S)-isopropyl-2,5-dimethoxy-dihydropyrazine is its commercial availability at a low cost, and it can also be readily synthesized from glycine and (2R)-valine. This method’s versatility extends to the preparation of tritiated L or D-amino acids. It is worth noting that longer straight-chain alkyl halides require extended reaction times compared to benzyl chloromethyl ether (Scheme 32). Finally, acid hydrolysis of the alkylated dihydropyrazine produces a mixture of the corresponding amino acid and valine methyl ester.

**Scheme 33:**
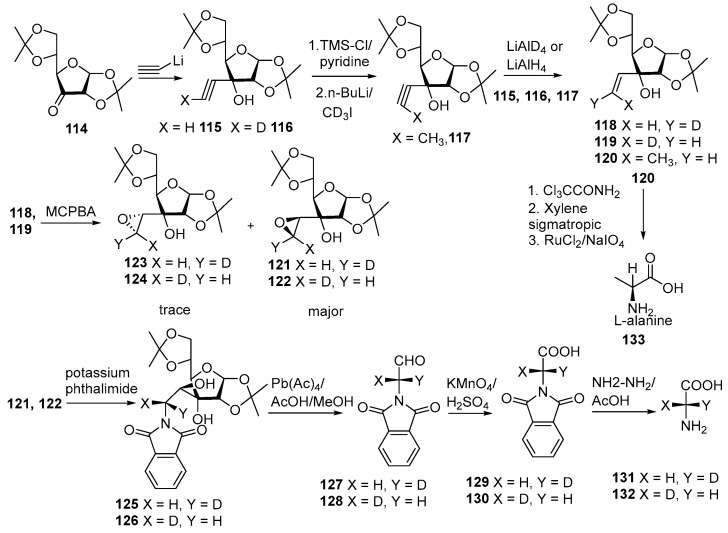
Synthesis of stereoselective glycine and alanine from the template **114**.

The chiral template diacetone-D-glucos-3-ulose **114** has proven highly effective in the stereoselective synthesis of a variety of chiral molecules, including chiral acetic acid, chiral glycine, D- or L-alanine, 3-alkylmalic acids, and chirally monodeuterated glycerol [85]. A key advantage of this carbohydrate template is its inherent double steric constraint, which directs the stereochemical outcome of reactions. The addition of acetylene to **114** was achieved using either lithium acetylide or an ethynyl magnesium bromide solution, yielding compounds **115** or **116**. Compound **117**, a starting material for L-alanine, was prepared in a three-step sequence involving silylation of the hydroxyl group, methylation of the acetylene, and desilylation, with an overall yield of 70% [86]. Reduction of **115**, **116**, or **117** using reagents like LiAlH_4_, LiAlD4, or Pd/C/H_2_CaCO_3_ provided stereospecifically Z or E olefin isomers **118**, **119**, and **120** (Scheme 33) [17,86]. Compound **120** was subsequently transformed into D- or L-alanine through a three-step process: protecting the hydroxyl group with trichloroacetamide, followed by a sigmatropic rearrangement in heated xylene, and finally periodate cleavage, as depicted in Scheme 33.

Due to the bulky α-oriented isopropylidene group at C4, only the β-face of the olefinic bond in intermediates **118** and **119** is accessible to incoming reactants. This steric hindrance ensures that electrophiles preferentially attack the less hindered side, leading to highly predictable stereochemistry. Indeed, epoxidation of **118** and **119** with *m*-chloroperbenzoic acid proceeded stereoselectively, yielding a diastereoisomeric mixture of **121**, **122**, and **122**, **123**, respectively, in an 87% yield and a 5:1 ratio [17]. This mixture was then separated using medium pressure column chromatography. Nitrogen functionality was introduced stereospecifically at the C-2′ position with an inversion of configuration by treating **121** and **122** with potassium phthalimide, which gave **125** and **126** [17]. In parallel, the methyl derivative **120** was transformed into **L-alanine-d_3_ 133** through a sequence involving ring opening with an amine and oxidative periodate cleavage. Periodate cleavage of **125** and **126** successfully yielded phthalimide glycinals **127** and **128** in good yields. Further oxidation of **127** and **128** with KMnO4 and H_2_SO_4_ provided the R and S phthaloyl glycines **129** and **130**. Finally, hydrolysis of **129** and **130** with Pb(OAc)_4_ in acetic acid and methanol effectively cleaved the phthaloyl group, affording the R and S glycines **131** and **132** (Scheme 33) [17].

**Scheme 34:**
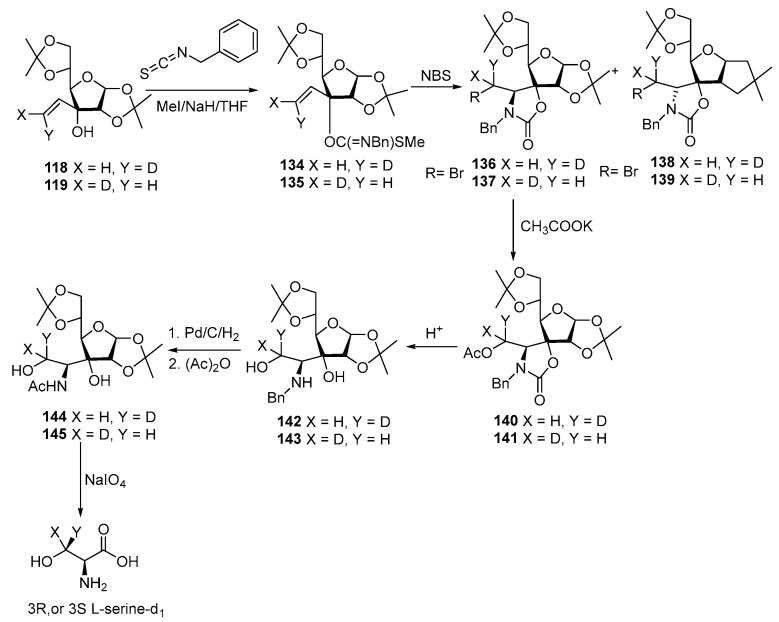
Stereoselective synthesis of β-deuterated 3R and 3S L-serines from the template **114**.

Maeda and colleagues developed a highly enantioselective method for synthesizing chirally pure 3R and 3S L-serines, utilizing intermediates **118** and **119** [85]. This approach relies on a sugar template, which ensures predictable stereochemistry in subsequent reactions. Intermolecular halogen addition to compounds **134** and **135**, both bearing an N-benzyl-methylthioformimidoyl group on the C-3α-hydroxyl group, proceeded diastereoselectively. This yielded **136** and **137** as the major products, rather than **138** and **139**. Consequently, the resulting stereochemistry at C-1′ and C2 could be precisely predicted to give L-amino acids. The halomethyl groups of **136** and **137** are versatile and can be transformed into various substituents and functional groups. This characteristic allows for the expansion of this methodology to prepare a range of other chirally pure β-labelled L-amino acids (Scheme 34).

**Scheme 35:**
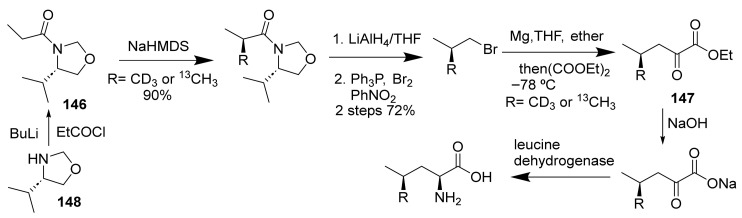
Enantioselective synthesis of isotopically labelled stable isotopes of L-leucine or isoleucine.

A general method has been developed for the enantioselective synthesis of isotopically labeled L-leucine or isoleucine. This approach involves preparing 2-oxo-4-methylpentanoic acid **146** from Evans chiral auxiliary **148**, which is selectively labelled with either 13C or deuterium in the R or S configured methyl group. The process then continues with a reductive amination of ketone **147**, catalyzed by leucine dehydrogenase (Scheme 35) [87]. This strategy has been successfully applied to the total synthesis of (2S,4R)-leucine-d_3_ using CD_3_I as the deuterium source. If the R group is ethylbromide-d_5_, the resulting compound will be (2S,4R)-isoleucine-d_5_.

Christopher J. Easton and his team have reported a novel method for the stereoselective synthesis of α-deuterated and (R), (S) α,β-dideuterated phenylalanine derivatives from (S)-phenylalanine. Their work also extends to preparing α-deuterated (R) alanine, (R) valine, and (R) leucine from their respective S-isomers. This new synthetic approach for chiral (R), (S) α-deuterated and (2S,3R) and (2R,3S) α,β-dideuterated phenylalanine derivatives is particularly significant because it bypasses the need for enzyme-catalyzed resolution or any other enantiomer separation techniques. This simplification streamlines the production of these valuable labelled compounds [88].

**Scheme 36:**
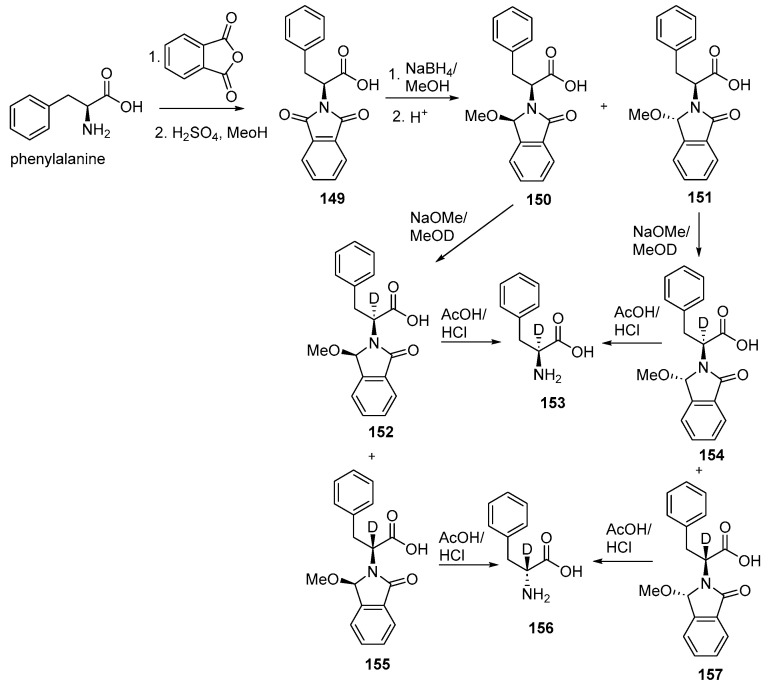
Stereoselective conversion of S-phenylalanine to α-deuterated (R) and (S)-phenylalanine isomers.

As previously discussed (referencing Scheme 7 and Scheme 8), racemic α-deuterated amino acids can be prepared using acetic anhydride and D_2_O, followed by enzymatic resolution of the esterified α-deuterated amino acids to yield optically pure L-α-deuterated amino acids. However, achieving high enantiomeric purity for the (D) R-isomer can sometimes be challenging. To address this, the authors developed a method for preparing both the (L) S- and (D) R-α-deuterated phenylalanines, **L-153** and **D-156**, directly from L-phenylalanine. This approach leverages the concept of self-regeneration of chirality through manipulation of an N-protecting group (Scheme 36) [88]. The diastereoisomers shown in Scheme 36 were successfully separated by silica gel chromatography. Reduced amides **150** and **151** were then treated with NaOMe in MeOD at reflux for 4 h, resulting in mixtures of the monodeuterides **152** and **155**, and **154** and **157**, respectively. Individual compounds were separated from these mixtures via silica chromatography, and subsequent acid hydrolysis yielded **153** and **156** from **152** and **154**, and **155** and **157**, respectively. Ultimately, the monodeuterated phenylalanines **153** and **156** were obtained as highly enantiomerically pure compounds (greater than 95% ee).

**Scheme 37:**
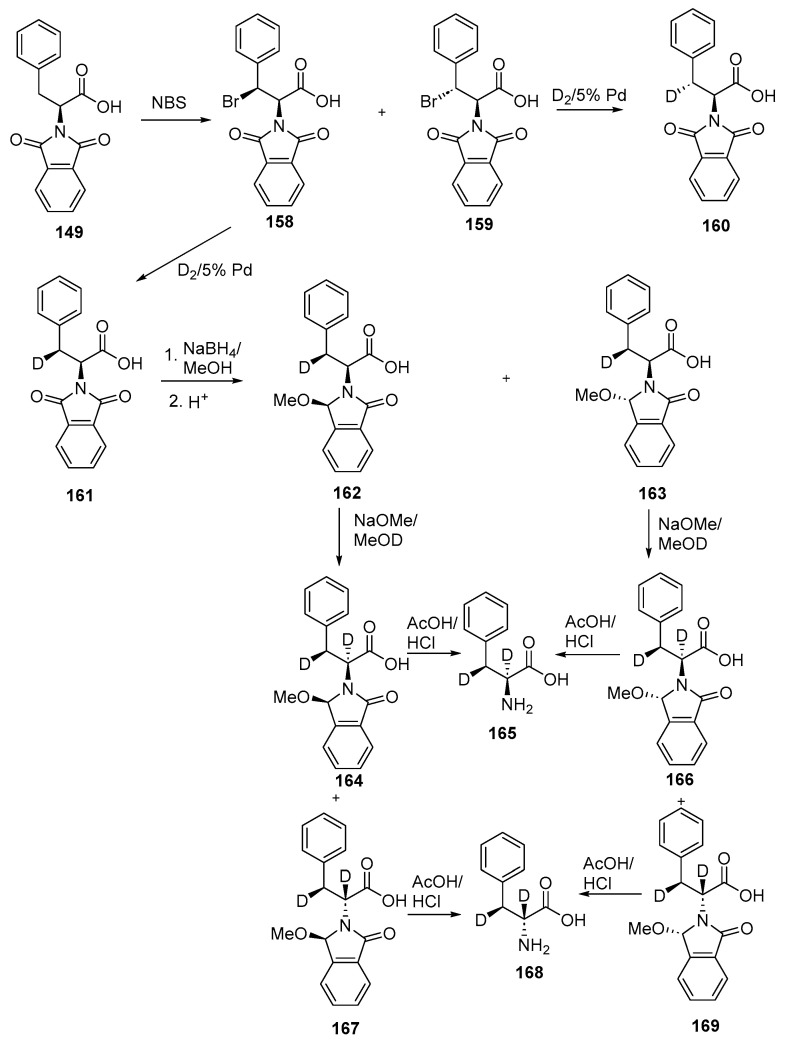
Stereoselective conversion of S-phenylalanine to α,β-deuterated (R,S) and (R,R)-phenylalanine isomers.

**Scheme 38:**
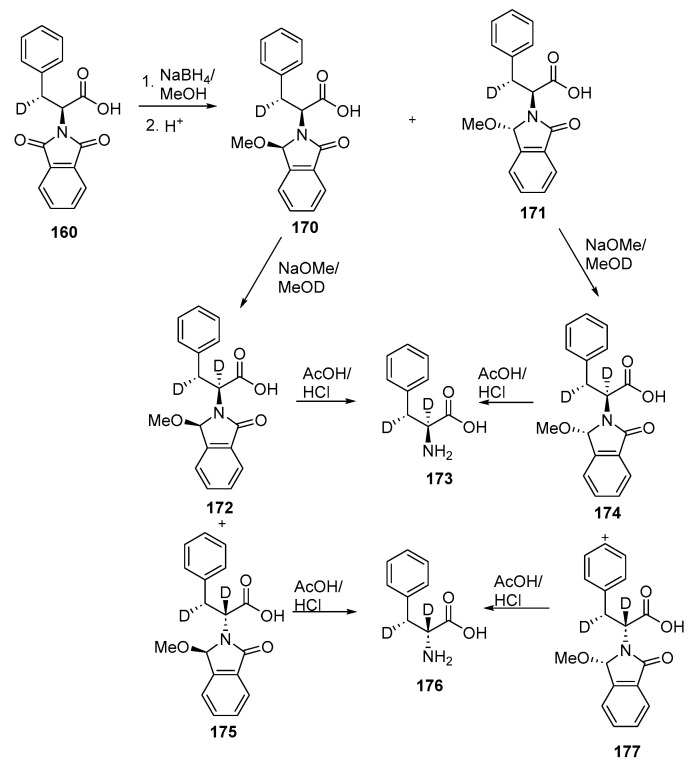
Stereoselective conversion of (L) S-phenylalanine to α,β-deuterated (S,S) and (S,R)-phenylalanine isomers.

A similar methodology has been successfully applied to prepare four distinct α,β-deuterated phenylalanine isomers (**165**, **168**, **173**, and **176**) in a stereocontrolled manner. This synthesis begins with the phthaloyl derivative of phenylalanine **149**. Halogenation of **149** with N-bromosuccinimide (NBS) yielded bromides **158** and **159**, crucially without any loss of stereochemistry at the α-carbon. These bromides then underwent β-deuteration with D_2_/Pd/C to give **160** and **161**, respectively. As a result, all four α,β-deuterated isomers **165**, **168**, **173**, and **176** were obtained as single enantiomers, as detailed in Scheme 37 and Scheme 38 [88]. All the diastereomers produced were pure and thoroughly characterized using NMR spectroscopy. Their mass spectra confirmed a high deuteration level, indicating they were 95% deuterated.

**Scheme 39:**
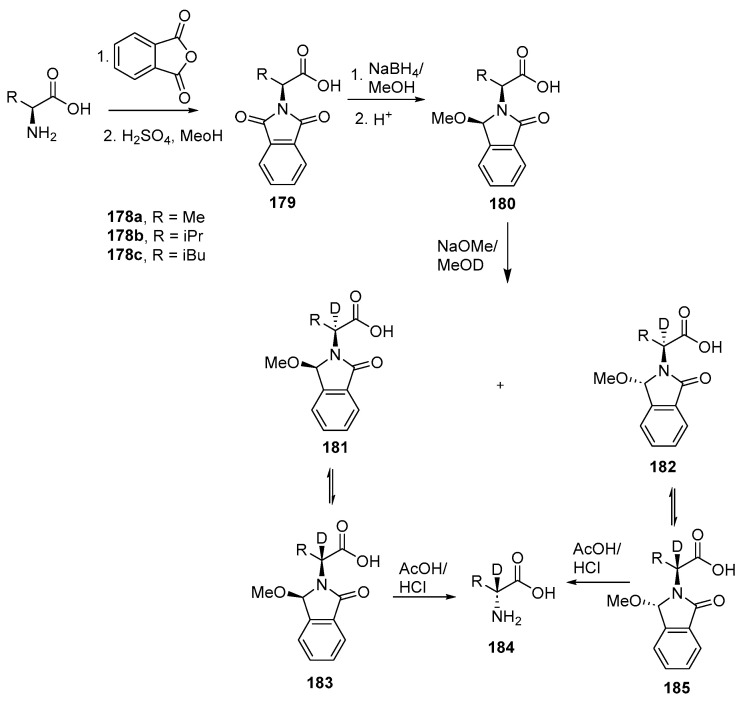
Conversion of (L) S-isomers of alanine **178a**, valine **178b**, leucine **178c** to the corresponding α-deuterated R-isomers **184a**, **184b**, and **184c**.

The same methodology extended to the conversion of (L) S-isomers of alanine **178a**, valine **178b**, and leucine **178c** to the corresponding α-deuterated R-isomers **184a**, **184b**, and **184c** as shown in Scheme 39.

**Scheme 40:**
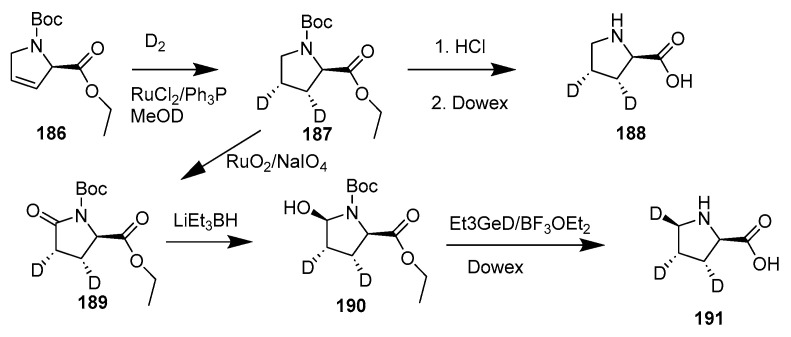
Synthesis of stereoselective specific deuteration of L-proline was reported by group Makoto Oba et al.

Chirally deuterium-labelled proline is highly valuable for probing peptide residue conformations and conducting detailed stereochemical investigations [89]. Makoto Oba’s group reported a method for the stereoselective deuteration of L-proline [89]. They achieved this by first performing a catalytic deuteration of protected 3,4-dehydro-L-proline **186** using RuCl_2_(PPh_3_)_3_. This was followed by RuO_4_-oxidation, which yielded a 3,4-dideuterated L-pyroglutamic acid **189** derivative (Scheme 40). This derivative is a promising precursor for various other deuterated amino acids. Furthermore, a stereoselective reduction of the amide carbonyl group led to L-proline-3,4,5-d_3_ **191**, where all the ring methylenes are stereoselectively labelled with deuterium. Additionally, Proline-d_2_ **188** was prepared through acid hydrolysis of **187**.

**Scheme 41:**
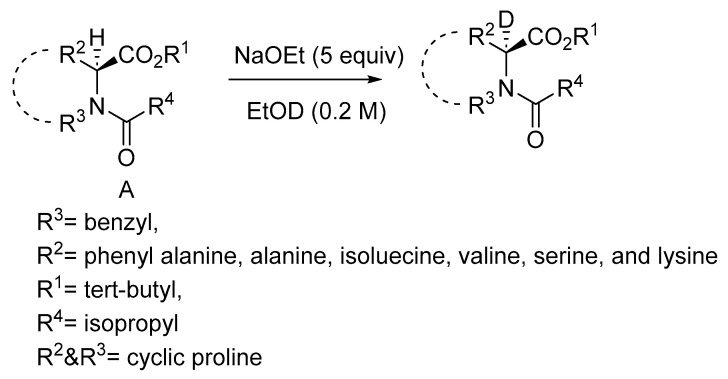
α-Deuteration of common amino acids with retention of enantiomeric configuration.

More recently, a new enantioselective deuteration method at the α-position of amino acids like proline, serine, cystine, phenylalanine, and lysine has been reported, notably without the need for external chiral sources (Scheme 41) [90]. When derivative A was treated with NaOEt in EtOD at room temperature, the α-deuterated product formed with retention of the C-α configuration. Interestingly, using dimethyl formamide significantly reduced the reaction’s efficiency and selectivity, and adding a nonpolar solvent also led to a considerable decrease in both yield and enantioselectivity. A supporting mechanism for enantioselective deuteration has also been proposed, in which deuteration likely occurs via direct transfer from the EtOD solvent to the chiral enolate.

**Scheme 42:**
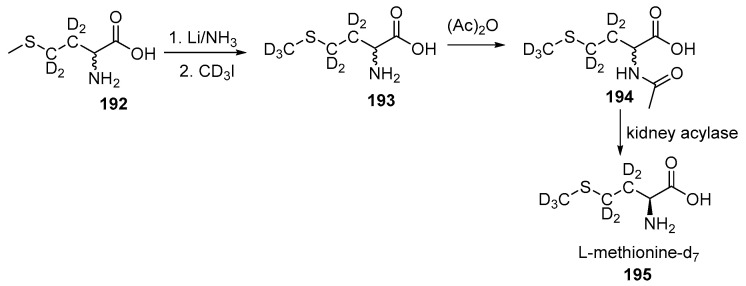
Methionine and its derivatives selectively deuterated at S-alkyl group.

Deuterated L-methionine tracers, like L-methionine-d_7_, are important tools for studying methionine metabolism, particularly in investigations such as the rat methionine loading test. Hiroshi and colleagues developed a straightforward method for synthesizing DL-methionine-d_7_ from DL-methionine-d_4_ (Scheme 42) [91]. This involved converting the S-CH_3_ group to S-CD_3_ using Birch reduction conditions. The resulting racemic methionine-d_7_ **194** was then resolved to give the desired **195** through selective hydrolysis of its N-acylated derivative using kidney acylase [91].

**Scheme 43:**
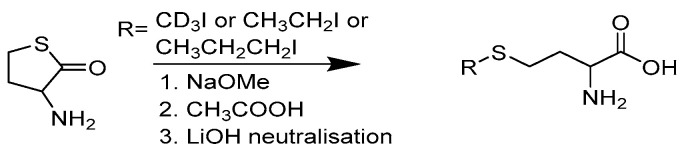
Protonated and deuterated DL-methionine derivatives were prepared from DL-homocysteine thiolactone hydrochloride.

Both protonated and deuterated DL-methionine derivatives can be prepared from DL-homocysteine thiolactone hydrochloride (HCTL) (Scheme 43) [92]. In this process, DL-homocysteine thiolactone hydrochloride is dissolved in NaOMe in methanol. After 30 min, an alkyl halide is added to yield the DL-S-alkyl homocysteine ester. This ester is subsequently converted to its free acid form via saponification with either LiOH or NaOH.

**Scheme 44:**
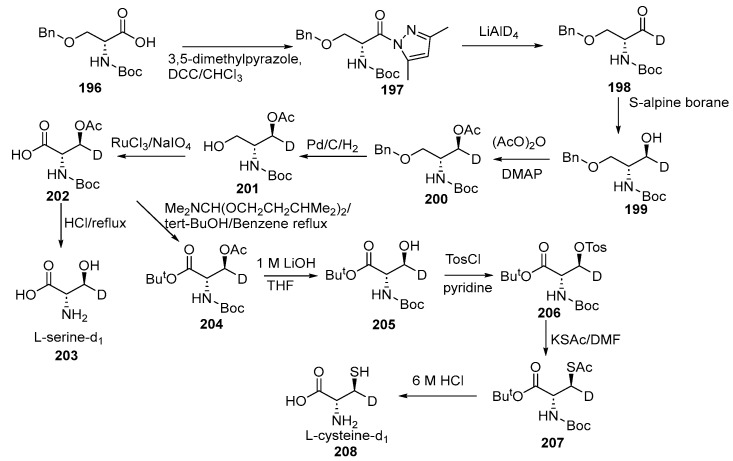
Synthesis of stereo selectively β-deuterated serine and cysteine from deutero aldehyde **198**.

Stereoselective deuteration of amino acids is crucial for determining the 3D solution structure of peptides and proteins using NMR spectroscopy. Makoto Oba and colleagues developed a method for the stereoselective β-deuteration of serine and cysteine, synthesizing them from deuterated aldehyde **198** without the need for biotransformations [25]. As shown in Scheme 44, protected serine **196** was first reacted with dimethyl pyrazole to afford compound **197**, which was subsequently reduced to yield the aldehyde intermediate **198**. Asymmetric reduction of **198** using S-alpine borane resulted in the chirally labelled alcohol **199**. Compound **199** underwent acetylation and debenzylation, followed by RuO_4_ oxidation of its hydroxymethyl group, to give protected serine **202**. Acid hydrolysis of **202** then successfully yielded the target compound, L-serine-d_1_ **203**, in good yield. L-cysteine-d_1_ **208** was also synthesized from protected serine **202** in four steps (Scheme 44). This involved removing the acetate group, introducing a tosyl leaving group, and then treating the intermediate with KSAc to obtain protected L-cysteine-d_1_-thioacetate **207**. Finally, acid hydrolysis of **207** provided L-cysteine-d_1_ **208**.

**Table 1:** **Comparative Analysis of Deuteration Methods**.

Method	Cost	Scalability	Stereochemistry/Site Specific	Applicability
Direct Deuteration (Acid/Base Catalyzed)	Low	High (with D_2_O)	Typically loses stereochemistry; challenges in site-selectivity without directing groups.	Broad applicability for many functional groups; effective for introducing multiple deuterium atoms.
Specific Deuteration (Acetic Anhydride/Acid/Aldehyde)	Low	High (with D_2_O)	Loss of stereochemistry but high regiospecificity.	Broad applicability for many functional groups.
Specific Deuteration (Pyridoxal/AlSO_4_)	Low	High (with D_2_O)	Loss of stereochemistry but high regiospecificity.	Not applicable to cysteine, serine, threonine, histidine, and tryptophan.
Metal-Mediated Hydrothermal Deuteration (Pt/C, Pd/C, PtO_2_)	Low	High (with D_2_O)	Loss of stereochemistry and less regiospecific.	Applicable to a limited number of amino acids like phenylalanine, tyrosine, and phenylglycine.
Ruthenium-Catalyzed Deuteration	High	Low to Medium	May or may not be stereoselective; high regiospecificity.	Broad applicability for many amino acids; not suitable for perdeuteration.
Metal or nonmetal ligand-Based Deuteration	High	Low	Highly stereoselective and highly regiospecific.	Broad applicability for many amino acids; not suitable for perdeuteration.
Chemical Synthesis	High	Low	May be stereoselective.	Broad applicability for many functional groups; good for introducing multiple deuterium atoms.
Stereoselective Chemical Synthesis (Chiral Auxiliary)	High	Low	Highly stereoselective and highly regiospecific.	Broad applicability for many amino acids, enabling both perdeuteration and site-specific labelling.

## 4. Conclusions

Chemical deuteration of α-amino acids and their optical resolution is a rapidly evolving field with significant implications for various research areas. The presented manuscript provides overview of the different methods of metal-catalyzed and chemical-mediated selective perdeuteration and their optical resolution of amino acids. Papers related to deuteration by chemical synthesis using deuterated precursors followed enzymatic resolution to give enantio-pure amino acids, and deuteration by stereoselective chemical synthesis using chiral auxiliary regents were also covered in this paper. Papers related to synthesis of both (S or L)- and (R or D)-α-deuterated amino acids from L-amino acids through manipulation of an N-protecting group, based on the concept of self-regeneration of chirality, were also presented. Investigations from our labs provided different methods of metal-catalyzed or chemical-mediated selective perdeuteration, and their enzymatic and kinetic resolution of biologically important amino acids such as L-DOPA, L-alanine, D-phenylglycine, L-methionine, L-cysteine, L-phenylalanine, L-tyrosine, and diaminobutyric acid were presented. These days, deuterated biologically important amino acids play crucial role in neutron scattering, neutron reflectometry, and NMR, and as internal standard in quantitative analysis by the LC-MS technique. Some perdeuterated or specific deutersted amino acids are commercially available at exuberant cost. Although deuteration has become a well-established methodology in chemistry, this field still offers interesting potential for innovation (e.g., with respect to heterogenous and homogenous catalysts). Most heterogeneous catalysis that support Pd, Pt, and Ru materials are commonly used to catalyze multi-H/D exchange of the arene part of amino acids. Unfortunately, the tolerance of these catalysts toward easily susceptible functional groups in amino acids as well as the selectivity is still challenging. Therefore, a strong need for the development of new methods for the selective and perdeuteration of amino acids and their optical resolution.

## Data Availability

This article presents both the author’s original contributions and a thorough review of the existing literature, all completed by the sole author. Further inquiries can be directed to the corresponding author.

## Supplementary Materials

The following supporting information can be downloaded at: https://www.mdpi.com/article/10.3390/bioengineering12090916/s1, General, Method and data-analysis, Synthesis, characterization, and enzymatic resolution of deuterated Br-DOPA-d_1_ 23, Synthesis, characterization and enzymatic resolution of selectively α-deuterated L and D-tyrosine and their resolution, Synthesis, characterization and kinetic resolution of L-alanine-d_7_ 37, Synthesis, deuteration, characterization and enzymatic resolution of L-methionine-d_3_ and L-homocysteine-d_3_, Synthesis, deuteration, characterization and kinetic resolution of D-phenyl glycine-d_6_, Synthesis, perdeuteration, and characterization of L-phenylalanine-d_8_ and L-tyrosine-d_7_, Synthesis, deuteration and characterization of L-2,4-diaminobuteryc acid-d_5_ DAB-d_5_, and Deuteration and characterization of azetidine-2carboxylic acid-d_5_. Figures S1–S103 NMR, Mass spectrum and resolution data.
