# Supplementary material for: Chemical Deuteration of α-Amino Acids and Optical Resolution: Overview of Research Developments
Source: Bioengineering (Basel). 2025 Aug 26;12(9):916. doi: 10.3390/bioengineering12090916 (PMC12467683; doi:10.3390/bioengineering12090916)
Supplement: Supplementary file 1 [file bioengineering-12-00916-s001.zip › bioengineering-3771290-supplementary.pdf]

bioengineering

MDPI

SUPPORTING INFORMATION

Chemical Deuteration of α-Amino Acids and Optical Resolution: Overview of Research Developments

Nageshwar R. Yepuri

National Deuteration Facility, Australian Nuclear Science and Technology Organisation, Lucas Heights, Australia

General, method and data-analysis; synthesis, characterisation and enzymatic resolution of deuterated Br-DOPA-d_1 23; synthesis, characterisation and enzymatic resolution of selectively α-deuterated L and D-tyrosine and their resolution; synthesis, characterisation and kinetic resolution of L-alanine-d_7 37; synthesis, deuteration, characterisation and enzymatic resolution of L-methionine-d_3 and L-homocysteine-d_3; synthesis, deuteration, characterisation and kinetic resolution of D-phenyl glycine-d_6; synthesis, perdeuteration and characterisation of L-phenylalanine-d_8 and L-tyrosine-d_7; synthesis, deuteration and characterisation of L-2,4-diaminobuteryc acid-d_5 DAB-d_5; and deuteration and characterisation of azetidine-2carboxylic acid-d_5.

Figures S1 to S102 NMR, mass spectrum and resolution data

Experimental Section

General

All reactions were performed under an atmosphere of nitrogen unless otherwise specified. Chemicals and reagents of the highest grade were purchased from Sigma-Aldrich (Sydney, Australia) and were used without further purification. Solvents were purchased from Sigma-Aldrich and Merck. NMR solvents were purchased from Cambridge Isotope Laboratories Inc. (MA, USA) and Sigma-Aldrich and were used without further purification. D_2O (99.8%) was supplied by AECL, Canada. Anhydrous dichloromethane, tetrahydrofuran and diethyl ether were obtained from a LC Technology Solutions Inc. SP-1 Stand Alone Solvent Purification System. Analytical thin-layer chromatography (TLC) was performed using Merck aluminium backed silica gel 60 F_{254} (0.2 mm) plates, which were visualised with shortwave (254 nm) ultraviolet light or with potassium permanganate, vanillin, or Hanessian's or bromocresol green stains. Flash column chromatography was performed using Merck Kieselgel 60 (230-400 mesh) silica gel, with the eluent mixture reported as the volume/volume ratio.

Methods and Data Analysis

Electrospray ionisation mass spectra (ESI-MS) were recorded on a 4000 QTrap AB SCIEX Mass Spectrometer. The overall percent deuteration of the molecules was calculated by ER-MS (enhanced resolution–MS) using the isotope distribution analysis of the different isotopologues by analysing the area under each MS peak, which corresponds to a defined number of deuterium atoms. The contribution of the carbon-13 (natural abundance) to the value of the area under each X+1 MS signal is subtracted based on the relative amount found in the protonated version. So, in a typical analysis we measure the C-13 natural abundance contribution by running ER-MS of the protonated version (or estimate it by Chem Draw software) and use this value in our calculation using an in-house

developed spread sheet which subtracts this contribution from each MS signal constituting the isotope distribution. Deuteration levels are also calculated using DGet! Software. ^{1}H NMR (400 MHz), ^{13}C NMR (100.6 MHz) and ^{2}H NMR (61.4 MHz) spectra were recorded on a Bruker 400 MHz spectrometer at 298 K. Chemical shifts, in ppm, were referenced to the residual signal of the corresponding NMR solvent. Deuterium NMR was performed using the probe's lock channel for direct observation.

Synthesis of deuterated Br-DOPA-d1 23:

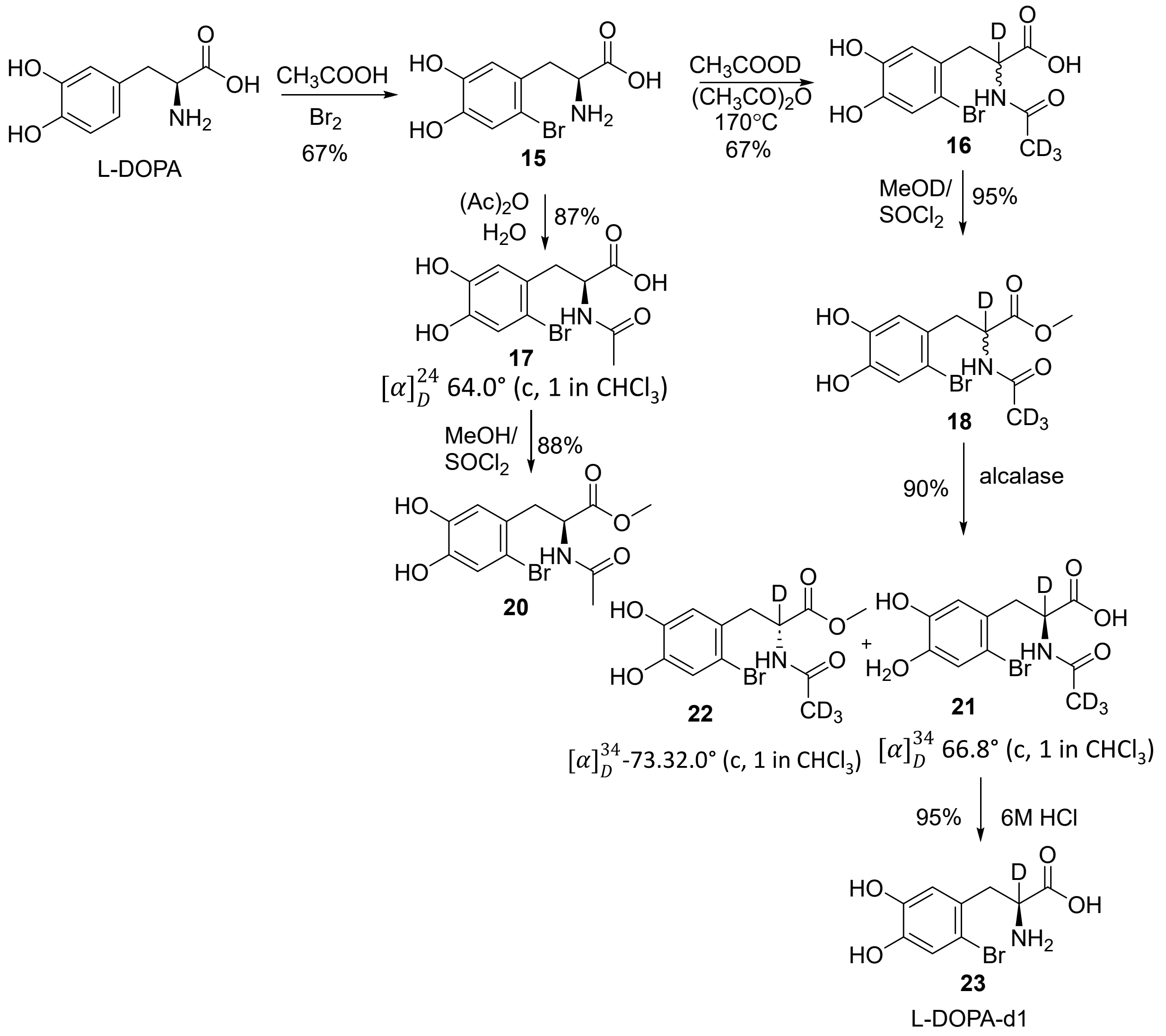

Synthesis of Br-L-DOPA 15:

To a stirred solution of L-DOPA (15 g, 0.076 moles) in glacial acetic acid (100 mL) add several drops of HCl until complete dissolution of dopa, then bromine was added dropwise (6.01 g, 0.076 moles, in glacial acetic acid 50 ml). The resulting solution was stirred at rt for 3 hrs, and then the solvent was removed under reduced pressure to produce a residue, which was redissolved in water (40 mL) to adjust pH with $NaCO_3$ to 3. Bromo-dopa was crystallised on cooling and filtered off, washed with cold water. Product was dried under vacuum to produce a white solid **15** (14.15 g, 67%).

^{1}H NMR (CD_3OD) δ 3.85 (d,d, J = 4.73 Hz, 1H), 3.40 (d,d, J = 4.73 Hz, 1H), 2.93 (d,d, J = 9.89 Hz, 1H), 6.83 (s, 1H) and 7.03 (s, 1H).

^{13}C NMR (CD_3OD) δ 36.6, 55.0, 112.4, 117.9, 119.1, 125.8, 145.1, 145.7 and 172.5.

ESI MS m/z : 300 [M+Na]$^+$.

Synthesis of Br-N-acetyl-DL-DOPA-d$_1$ 16:

A solution of Br-L-DOPA (2.00 g, 0.00725 mol) in D_2O (3.5 mL), after purging the solution with N_2, was added to acetic anhydride (Ac_2O) (20.0 mL, 194.20 mmol) dropwise over 1 h with an addition funnel. The reaction mixture was heated to 170 ^{0}C for 10 min and then allowed to cool. The solvents were removed in vacuo, and the residue was purified by flash column chromatography (40:60 MeOH/CH_2Cl_2), producing Br-N-acetyl-DL-DOPA-d$_1$ **16** as a sticky white solid. The product was then run through a second cycle with same conditions as above, producing a white solid (2.0 g, 86.9%) with 94%D based on internal proton reference.

^{1}H NMR (CD_3OD) δ 1.87 (m, 0.8H), 2.90 (d, J = 14 Hz, 1H), 3.21 (d, J = 14 Hz, 1H), 4.69 (m, 0.06H), 6.78 (s, 1H), 7.03 (s, 1H) and 7.52 (br, s, 1H).

^{13}C NMR (CD_3OD) δ 23.12 (m), 39.1, 54.6, 54.4 (m), 114.9, 120.1, 121.2, 129.7, 47.2, 147.6, 148.0, 172.8 and 174.8.

^{2}H NMR (CD_3OD) δ 4.75 (m).

ESI MS m/z: 320 [M-H]$^-$.

Synthesis of Br-N-acetyl-DL-DOPA-d$_1$ methyl ester 18:

To a cold solution (-10°C) of above Br-N-acetyl-DL-DOPA-d$_1$ (2.0 g, 0.0063 moles) in methanol-d$_1$ (30 ml) thionyl chloride was added (2 mL) dropwise. The resulting solution was then warmed to rt for 4 hrs. Then 20 mL of water and 15 mL solution of 0.8 g of $NaHCO_3$ were added. The pH of the solution was adjusted to 7 by addition of a dilute NaOH solution. The product was transferred to organic phase by extraction with oxygen-free ethyl acetate. The solvent evaporated to produce white amorphous powder, which is further recrystallised from diethyl ether to white powder **18** (1.8 g, 86.5%) and 98%D levels at α-position based on the internal standard.

^{1}H NMR ($(CD_3)_2CO$) δ 1.87 (m, 0.8H), 2.90 (d,d, J = 7.23 Hz, 1H), 3.13 (d,d, J = 6.23 Hz, 1H), 3.65 (s, 3H), 4.69 (m, 0.06H), 6.78 (s, 1H), 7.03 (s, 1H), 7.44 (br, s, 1H) and 8.25 (br,s, 2H, 2xOH).

^{13}C NMR ($(CD_3)_2CO$) δ 23.12 (m), 53.6, 54.4 (m), 114.9, 120.1, 121.2, 129.7, 147.2, 147.6, 171.6 and 174.2.

^{2}H NMR ($(CD_3)_2CO$) δ 4.69 (m).

ESI MS m/z: 334 [M-H]$^-$.

Synthesis of Br-N-acetyl-L-DOPA-d$_1$ 21:

To a suspension of racemic amino acid, 0.5g of ester in 60 ml mixed solvent of water (85% v/v) and ethanol was added to $NaHCO_3$ (0.5 g), while the pH of the solution was about 8. BL-Alcalase (0.25 g) was added to the mixture, and then the reaction mixture was gently stirred at 25 °C for 24 hrs under a nitrogen atmosphere. The solution was filtered through Celite bed to remove enzyme. The unhydrolyzed N-acetyl D-ester **22** was extracted by ethyl acetate (50 mL x 3). The combined organic layers were dried over $NaSO_4$ and evaporated to give **22** as a white solid, which is further recyclized from ethyl acetate (0.210 g, 45%). Spectral data of compound **22** is identical to that of compound **18**, whereas its optical rotation is $[\alpha]_D^{34}$ -73.32° (c, 1 in $CHCl_3$). To the aqueous phase 3N HCl was added until the pH was lowered to 2-3. Removal of water produced N-acetyl L-acid **21** crude, which was further purified by silica flash column chromatography using 40% methanol in chloroform to produce white solid **21** (0.205 g, 44%). Spectral data of compound **21** is identical to that of compound **16**. Compound **15** again converted to methyl ester **17** using same conditions as mentioned for compound **16.** The spectral data of **17** are

also comparable to compound **21,** whereas its optical rotation is $[\alpha]_D^{34}$ 66.8° (c, 1 in $CHCl_3$). The isomeric purity of **21** was further confirmed by comparing optical rotation of protonated **17**, which is synthesised according to literature procedure.

Synthesis of L-2-Br-DOPA-d₁ 23:

A solution of Br-N-acetyl-L-DOPA-d_1 **21** (0.180 g) in 3N HCl (10 mL) was heated to reflux for 12h. The solvent was evaporated to produce dark-coloured residue, which was further treated with $AqNH_3$ (28% solution) 2mL. After final evaporation, the residue was purified by flash column chromatography on silica using 40% methanol in chloroform to produce coloured compound **23** (60 mg, 40%), with 99%D levels according to the mass spectrum. Compound colour was removed by charcoal treatment with hot degassed methanol.

^{1}H NMR (CD_3OD) δ 2.90 (d, J = 14.4 Hz, 1H), 3.37 (d, J = 14.5 Hz, 1H), 3.82 (m, 0.09H), 6.83 (s, 1H) and 7.03 (s, 1H).

^{13}C NMR (CD_3OD) δ 38.7, 57.0, 114.7, 114.7, 119.9, 121.4, 128.1, 147.4, 147.9 and 174.5.

ESI MS m/z: 300 $[M+Na]^+$.

Synthesis of selectively α-deuterated L and D-tyrosine and their resolution:

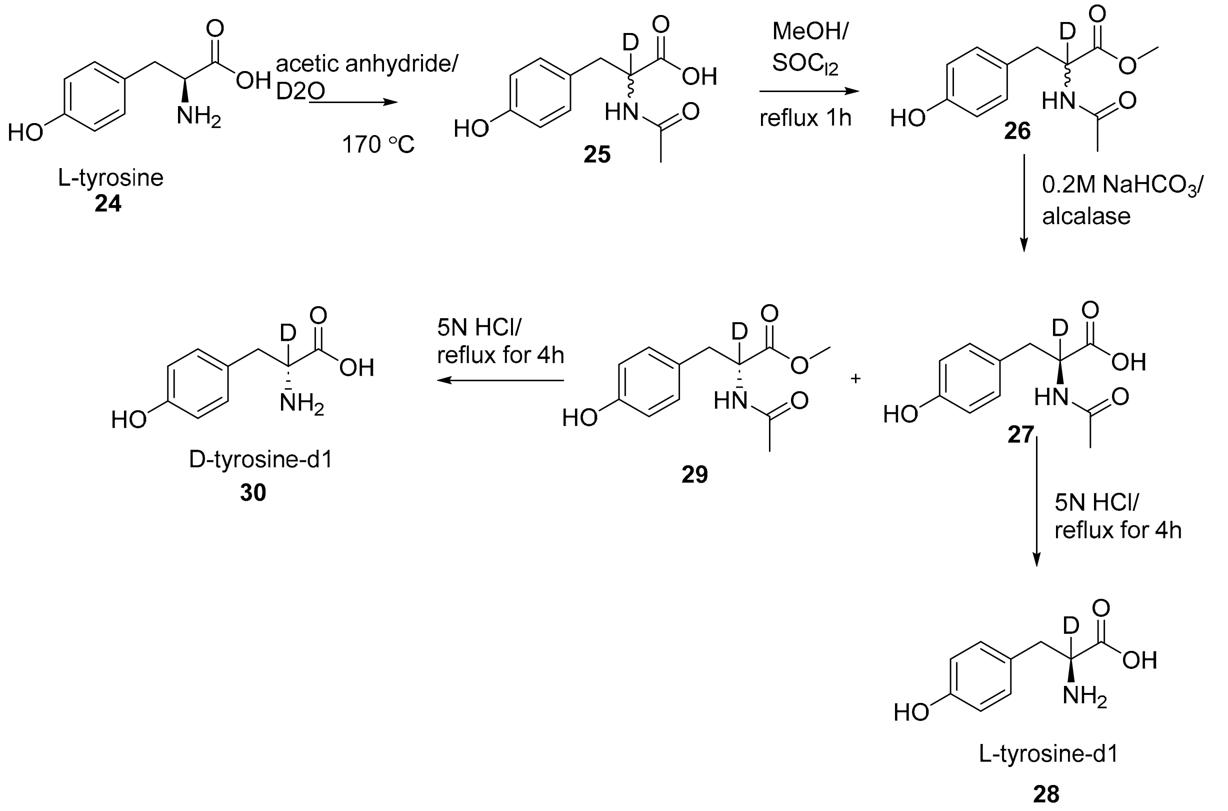

Synthesis of DL-acetyl tyrosine-d₁ (25):

A solution of L-tyrosine (amine and acid pre-exchanged with D_2O, added 50 ml D_2O and evaporated) (6.00 g, 0.0331 mol) in D_2O (6.5 mL) after purging the solution with N_2 was added to acetic anhydride (Ac_2O) (60.0 mL, 0.582 mol) dropwise over 1 h with an addition funnel. The reaction mixture was heated to 170 °C for 10 min and then allowed to cool. The solvents were removed in vacuo, and the residue and deuteration levels were based on the internal β-proton α-deuteration ~90%D. The product was then run through

a second cycle with same above conditions, and after second cycle, the deuteration level remained the same as 90%D. The residue was purified by flash column chromatography (40:60 MeOH/CH_2Cl_2), producing N-acetyl-tyrosine-d_1 as a sticky white solid **25** (4.5g, 60%).

Synthesis of DL-acetyl tyrosine-d_1-ester (26):

To a cold solution of N-acetyl tyrosine-d_1 (4.0g, 0.0177 mol) in MeOH (50 mL), 3 ml of H_2SO_4 was added slowly then refluxed for 4 hrs. After cooling, solvent evaporated to produce pale-yellow residue. Then 50 ml of water was added, and pH was adjusted to 7 and then extracted with EtOAc (3 x 75ml). Combined organic layers were dried over MgSO4 and evaporated to crude product. The crude was purified by flash column chromatography using 70% EtOAc in hexane to produce a white solid (4g, 95%) with 90%D.

^{1}H NMR ($CDCl_3$) δ 1.93 (m, acetyl), 2.93 (AB quartet, J = 14.7Hz, 1H), 3.04 (AB quartet, J = 14.7Hz, 1H), 3.70 (s, 3H), 4.85 (m, 0.13H), 6.25 (s, NH), 6.70 (d, J=8.02Hz, 2H) and 6.91 (d, J=8.02Hz, 2H).

^{2}H NMR ($CDCl_3$) δ 1.93 (m, acetyl) and 4.82 (m).

^{13}C NMR ($CDCl_3$) δ 22.3 (m), 52.7, 53.4 (m), 115.7, 126.7, 130.3, 155.7, 170.7 and 172.4.

ESMS m/z 226 [M^{+1}].

Enzymatic resolution of L-tyrosine-d_1 and D-tyrosine-d_1 (28 and 30):

Racemic DL-N-acetyl tyrosine-d_1 methyl ester (4.0 g, 0.0166 mol) was dissolved in 200 mL of 0.2 M $NaHCO_3$ solution. BL-Alcalase (200 mg PPL) was added to the mixture. The reaction was gently stirred at 38 °C for 25 minutes under nitrogen atmosphere. The completion was confirmed by mass spectral analysis showing 50:50 mixtures of N-acetyl acid and N-acetyl ester. The reaction mixture was passed through sintered funnel to remove enzyme. The filtrate was extracted with ethyl acetate (5 x 50 mL) and left-over aqueous part neutralised to from pH 7 to 6. The solution volume was reduced to 30ml and left overnight at 4 °C. Formed precipitate was filtered and dried to produce white solid, which was dissolved in 1N HCl 20 mL and heated to reflux for 4 h. After cooling it was filtered through sintered flask, and filtrate was evaporated to produce brownish solid. The solid was suspended in 50ml of DCM and we added 1.3 equivalents of Et_3N. After stirring for 10 min, 10 mL of cold water was added, stirring continued for another 10 min and the DCM layer separated. Precipitate in the water layer was filtered, washed with small amount cold water and dried to produce white solid L-tyrosine-d_1 **28** (0.6g, 40%). Specific rotation: $[\alpha]_D^{26.3}$ -11.901° (c, 4 in 1N HCl) (ref Sigma Aldrich $[\alpha]_D^{27}$ -11.5° (c, 4 in 1N HCl)).

^{1}H NMR (D_2O) δ 2.05 (AB quartet, J = 14.7Hz, 1H), 3.18 (AB quartet, J = 14.7Hz, 1H), 4.18 (m, 0.10H, residual α-proton), 6.80 (d, J = 8.02Hz, 2H) and 7.11 (d, J = 8.02Hz, 2H).

^{2}H NMR (D_2O) δ 3.72 (m) and 7.32 (m, arises from NH exchange with D_2O).

^{13}C NMR (D_2O) δ 34.7, 54.2 (m), 115.9, 125.7, 131.0, 155.2 and 171.7.

ESMS m/z 181 [M^{-1}]. Deuteration at α-carbon 88%D.

D-Tyrosine-d_1 (30):

Above combined EtOAc layers were dried over MgSO4 and filtered, and volume was reduced to 20 ml. The concentrated EtOAc was left overnight in the fridge at 4 °C to recrystalise the ester. The solid was filtered and dried to produce white crystalline solid (1.8g), which was dissolved in 30 mL of 1N HCl and was heated to reflux for 4 hr. After cooling, the clear solution was filtered through sintered funnel and evaporated to produce brownish solid. The solid was suspended in 50 ml of DCM, and while stirring 1.3

equivalents of Et_3N were added. After stirring for 10 minutes, 10 ml of water was added, and stirring continued for another 10 minutes. Precipitate in the aqueous layer was filtered, washed with small amount of water and dried to produce a white solid **30** (0.58g, 39%).

D-tyrosine-d_1-specific rotation $[\alpha]_D^{26.3}$ +11.901° (c, 4 in 1N HCl) (ref Sigma Aldrich $[\alpha]_D^{27}$ +11.5° (c, 4 in 1N HCl)).

^{1}H NMR (D_2O) δ 2.05 (AB quartet, J=14.7Hz, 1H), 3.18 (AB quartet, J=14.7Hz, 1H), 4.18 (m, 0.10H, residual α-proton), 6.80 (d, J=8.02Hz, 2H) and 7.11 (d, J=8.02Hz, 2H).

^{2}H NMR (D_2O) δ 3.72 (m) and 7.32 (m, arises from NH exchange with D_2O).

^{13}C NMR (D_2O) δ 34.7, 54.2 (m), 115.9, 125.7, 131.0, 155.2 and 171.7.

ESMS m/z 181 [M^{-1}]. Deuteration at α-carbon 89%D.

Synthesis of deuterated L-alanine-d_7 37:

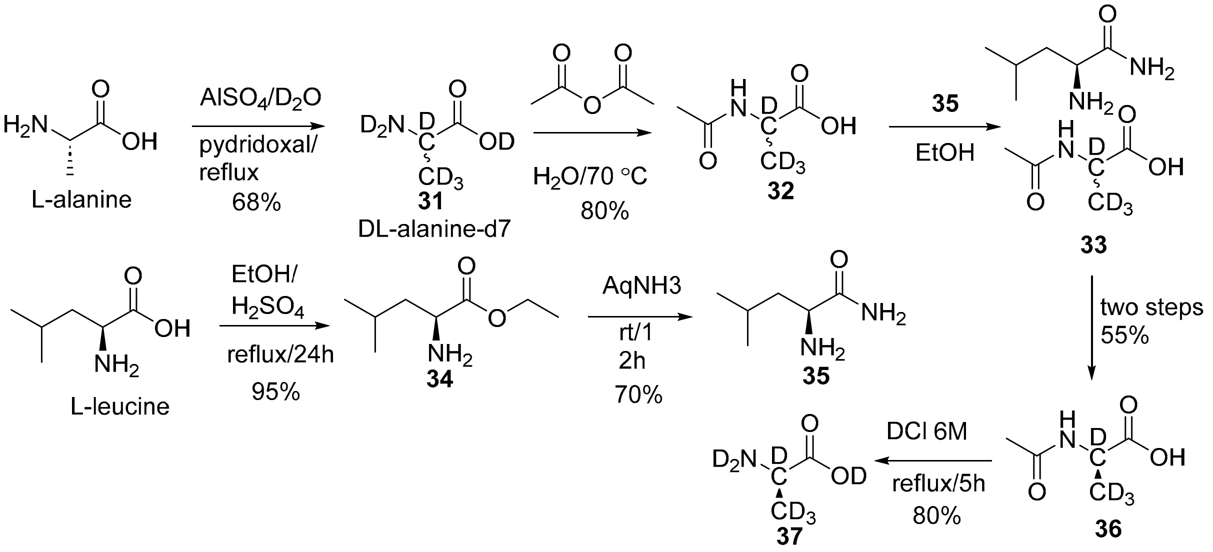

Preparation of DL-alanine-d_7 31:

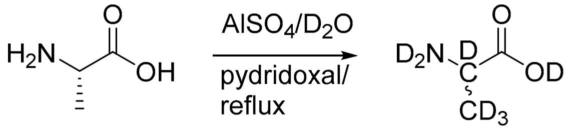

alanine-d7

A 1000 ml solution (D_2O as solvent) containing 100.0 g of DL-alanine, 20 g of pyridoxal hydrochloride and 90 ml of Al(III) D_2O solution ($Al(SO_4)_3$ $18H_2O$, 9.4 g, dried at 70 °C under vacuum and dissolved in 100 mL of D_2O) was adjusted to pH 5.5 using NaOD. The solution was refluxed for 24 h. The product was isolated at 0 ° C by raising the pH to 10 and then cooling and adding ethanol. The product was filtered, dried, dissolved in D_2O (200 mL) and then lyophilised to produce white solid **31** 68 g (68%). Overall deuteration was found to be 90% ±1, deuteration α position 97% and β 86%.

^{1}H NMR (D_2O) δ 1.35 (m) and 3.64 (m).

^{2}H NMR ($CDCl_3$) δ 1.26 (s, 1D) and 3.57 (s, 1.57D).

^{13}C NMR ($CDCl_3$) δ 15.7 (m), 50.1 (m) and 175.9 (s).

ESI-MS: [M^{+1}] 97. Isotopic distribution 55.0% d_7; 22.5% d_6; 15.6 %, d_5; and 6.8 % d_4.

Synthesis of N-acetyl alanine-d_7 32:

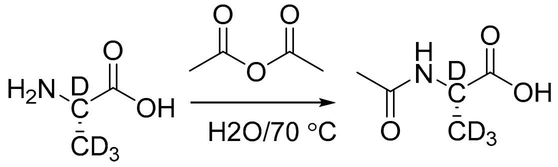

n-acetyl alanine

To a mixture of acetic anhydride (60 mL) and water (11 mL) DL-alanine-d_7 (8.0 g) was added at room temperature. The suspension was heated to 70 °C while stirring for 5 h. After cooling the reaction mixture, water (20 mL) was added, and we left the mixture for 24 h for crystallisation. We filtered and dried the crystallised product to produce white solid (9.28g), 80% yield.

^{1}H NMR (D_2O) δ 1.39 (m, 0.64), 1.98 (s, 3H) and 4.39 (m, 0.08).

^{2}H NMR ($CDCl_3$) δ 1.32 (m) and 4.33 (m).

^{13}C NMR ($CDCl_3$) δ 16.7 (m), 20.9, 51.6 (m), 171.6 and 174.3.

L-Leucinate preparation 34:

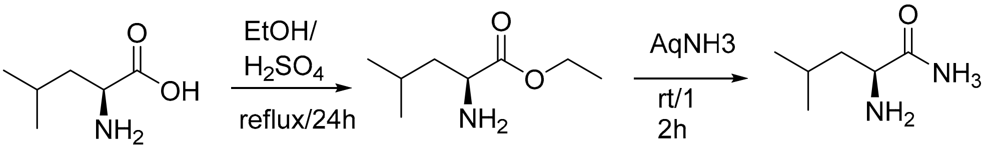

L-leucine

To a clear solution of L-leucine (80.0 g, 0.6103 mol) in ethanol (550 mL) H_2SO_4 (170 mL) was added dropwise. The reaction refluxed for 24 h. After cooling, concentrated aqueous ammonia was added dropwise to the reaction solution, until pH of about 8, while large amount of white ammonium sulphate was precipitated in the reaction solution. Water (70 mL) was added to make the solution clear. The resultant diethyl ether solution was extracted (7 x 200 mL). The combined organic layers were dried over MgSO4 and filtered. The solvent was evaporated to produce colourless liquid **34,** 92.2 g and 95%.

^{1}H NMR (D_2O) δ 0.95 (t, J = 6.8 Hz, 6H), 1.26 (m, 3H), 1.36-144 (m, 1H), 1.51-158 (m, 3H), 1.76 (m, 1H), 3.43 (m, 1H) and 4.12 (q, J = 7.42Hz, 2H).

^{13}C NMR (D_2O) δ 14.4, 22.0, 23.1, 25.0, 44.2, 53.1, 60.7 and 176.9.

L-Leucinamide 35:

A mixture of ethyl L-leucinate **34** (90.0 g, 0.56557 mol) and 28% aqueous ammonia (500 mL) was stirred for 12h. Excess ammonia was removed under reduced pressure, and hydrolysed L-leucine settled in the bottom and was removed by decanting the solution. The solution was adsorbed onto Diaion SK (ammonium form). The column was eluted with 2N aq. NH3 (500 mL). The elute was evaporated to produce white solid, which was further purified by recrystallization from boiling benzene, and L-leucinamide was obtained as colourless plate crystals, 51.5 g (70%).

^{1}H NMR (CD_3OD, $CDCl_3$) δ 0.86 (m, 6H), 1.33 (m, 1H), 1.52 (m, 1H), 1.62 (m, 1H) and 3.33 (m, 1H).

^{13}C NMR (CD_3OD, $CDCl_3$) δ 21.6, 23.2, 24.8, 43.9, 53.0 and 179.2.

Resolution of N-Acetyl-DL alanine to N-acetyl-L-alanine-d_4 36:

N-Acetyl-DL-alanine-d_4 (65.0 g, 0.500 mol) and L-leucinamide (71.5 g, 0.550 mol) were dissolved in 1L of ethanol at 50 °C. We left the reaction mixture overnight at room temperature; crystallised product was filtered, washed with a little amount of ethanol and dried to produce white crystalline product, 40.0 g and 61.5% yield (based on L-form). The salt was showing an optical rotation of $[\alpha]_D^{25}$ -8.96° (c, 2 in water) and reference $[\alpha]_D^{25}$ -

10.5° (c, 2 in water). The above salt was dissolved 300 mL of water loaded onto cation resin (200 g) Amberlite-IR-120 (previously washed with methanol until colourless). The resin column was eluted with each (7x 200 ml) water fraction. The combined elutes were evaporated to produce a white solid, 18 g, and N-acetyl-L-alanine-d_4 was showing an optical rotation of $[\alpha]_D^{25}$ 60.7° (c, 2 in water) reference $[\alpha]_D^{25}$ 63.6° (c, 2 in water).

Hydrolysis of N-acetyl-L-alanine-d_4 to L-alanine-d_7 37:

A solution of N-acetyl-L-alanine-d_4 (18.0 g, 0.13325 mol) in DCl 6M (50 mL) was heated to reflux for 5 h. After cooling to room temperature, contents were dried to produce white solid; then solid was redissolved in 50 ml D_2O, and acetone was added until precipitate was observed. The solid was filtered and dried to produce white solid, 10.0 g and 80% yield. Optical rotation for the resolved L-alanine-d4 $[\alpha]_D^{25}$ 12.2° (c, 1 in 5M HCl) reference $[\alpha]_D^{25}$ 14.2° (c, 1 in 5M HCl). NMR spectral data is identical with that of DL-alanine-d7.

Synthesis and deuteration of L-methionine-d_3 and L-homocysteine-d_3:

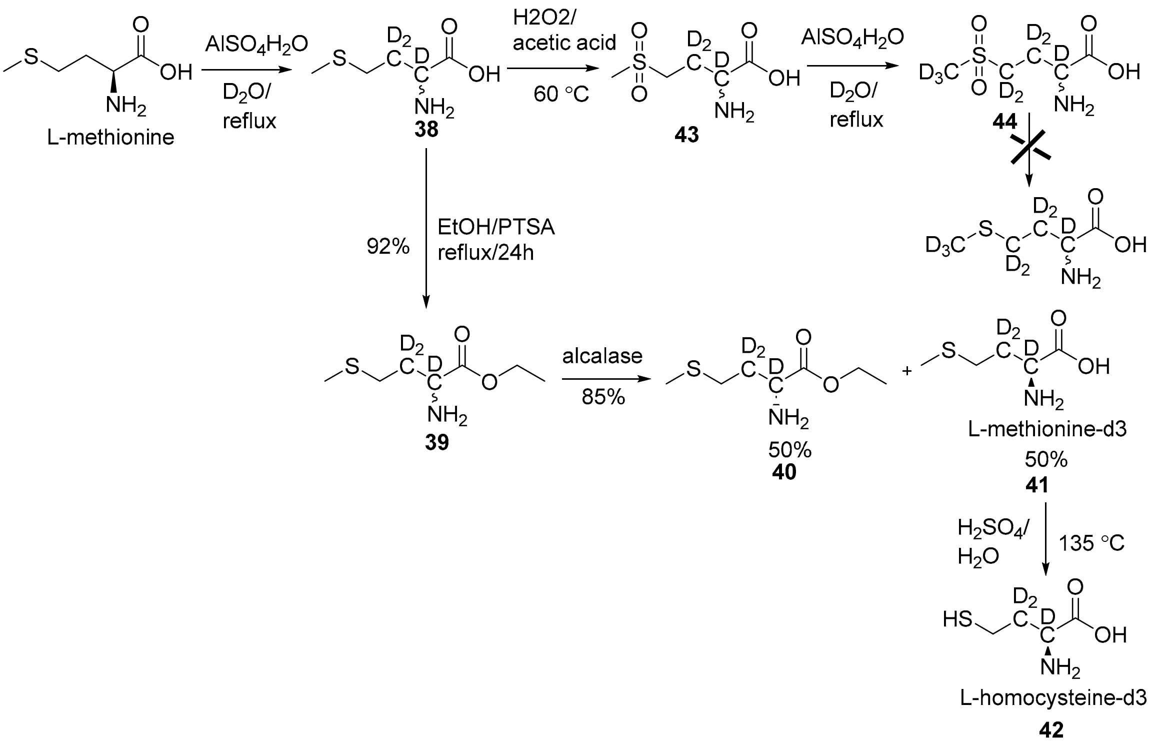

Preparation of Methionine-d_3 (38):

A 150 ml solution (D_2O as solvent) containing 10.0 g of L-methionine, 2 g of pyridoxal hydrochloride and 10 ml Al(III) of D_2O solution (($Al(SO_4)_3$ $18H_2O$, 9.4 g, dried at 70 °C under vacuum and dissolved in 100 mL of D_2O) was adjusted to pH 5.5 using NaOD. The solution was refluxed for 24 h. The product was isolated at 0 ° C by raising the pH to 10 and then cooling and adding acetone. The product was filtered, dried, dissolved in D_2O (40 mL) and then lyophilised to produce a white solid **38** (6.8 g, 68%). Overall deuteration was found to be 91% ±1, with deuteration α position of 97% and β 88%.

^{1}H NMR (D_2O) δ 1.80 (s, 3H), 1.89 (m, 0.13H), 1.98 (m, 0.14H) and 2.37 (s, 2H).
^{2}H NMR (D_2O) δ 1.94 (m, 2D) and 3.96 (m, 1D).
^{13}C NMR (D_2O) δ 13.9 (s), 28.1 (s), 28.6 (m), 51.3 (m) and 171.3 (s).
^{13}C NMR (D_2O) {^{1}H} and {^{2}H} decoupled spectra δ 13.9, 27.9, 28.6, 51.3 and 171.8.
ESI-MS: m/z 151 [M^{-1}].

DL-Methionine-d$_3$ ethyl ester (39):

To a solution of DL-methionine-d$_3$ (6.8 g, 0.04472 mol) in ethanol (100% 100 mL), *p*-toluene sulfonic acid (8.47 g, 0.0492 mol) was added, and the solution was refluxed for 24 h. The solvent was evaporated at reduced pressure, and the residue was triturated with cold diethyl ether. The product separates out as a white, crystalline solid. The crystals were filtered out, and the crystals were washed with diethyl ether and dried to yield (7.66 g, 95%) crystalline solid **39**.
^{1}H NMR (D_2O) δ 1.19 (t, 7.02Hz, 3H), 2.09 (s, 3H), 2.14 (m, 0.13H), 2.56 (m, 2H) and 4.20 (q, 7.02Hz, 2H).
^{2}H NMR (D_2O) δ 2.13 (m, 2D) and 4.17 (m, 1D).
ESI-MS: m/z 202.6 [M^{+Na}]

L-Methionine-d$_3$ (41):

Racemic DL-methionine ester (3.0 g, 0.0167 mmol) was suspended in 360 mL mixed solvent of water (85% v/v) and ethanol. To this, 3.0 g of NaHCO3 was dissolved in the reaction mixture, while the pH of the solution was about 8. BL-Alcalase (0.40 g PPL) was added to the mixture. The reaction was gently stirred at 38 °C for 72 h under nitrogen atmosphere. The completion was confirmed by mass spectral analysis, showing 50:50 mixtures of free acid and ester. The reaction mixture was passed through Celite bed to remove enzyme. The filtrate was extracted with ethyl acetate (5 x 50 mL). Left-over aqueous extract reduced the volume, and pH was adjusted to 7 using 1M HCl. The neutral solution was left in the fridge at 4 °C overnight to produce precipitate, which was filtered and dried to produce L-methionine-d$_3$ as white solid **41** (1.07 g, 85%) with 91%D. Optical rotation for resolved L-methionine-d$_3$ **41** $[\alpha]_D^{25}$ 17.09° (c, 1 in 1M HCl) reference $[\alpha]_D^{25}$ 23.1° (c, 1 in 1M HCl). L-methionine-d$_3$ **41** NMR spectral data was the same as DL-methionine-d$_3$ **38**.

L-Homocystine-d3 42:

To a solution of L-methionine-d$_3$ (1.0 g, 0.00657 mol) in concentrated sulfuric acid (6.0 g, 0.06124 mol), water (4 mL) was added slowly, and then the contents were heated under reflux for 5 hours at 135°C in a stream of nitrogen. The reaction solution was analysed by mass spectrometry. The reaction solution was neutralised with 10% sodium hydroxide aqueous solution while cooling, and the resulting precipitate was collected by filtration, washed with cold water and then dried to produce 0.41 g of L-homocystine-d3. The yield was 45%. Optical rotation for the resolved L-homocystine-d3 $[\alpha]_D^{25}$ was 20.06° (c, 1 in 1M HCl) reference $[\alpha]_D^{25}$ 25.0° (c, 1 in 1M HCl)). Overall, 90%D levels at α,β-positions were based on the internal methylene standard.
^{1}H NMR (D_2O) δ 2.23 (m, 0.30H), 2.67 (m, 2H) and 4.05 (m, 0.04H).
^{2}H NMR (D_2O) δ 2.19 (m, 2D) and 4.04 (m, 1D).
^{13}C NMR (D_2O) δ 28.7 (m), 31.9 (s), 51.0 (m) and 171.3 (s).
^{13}C NMR (D_2O) {^{1}H} and {^{2}H} decoupled spectra δ 28.4, 31.9, 51.1 and 171.3.
ESI-MS: m/z 137 [M^{-1}]

DL-Methioninedioxide-d$_3$ 43:

To a solution of DL-Methionine-d3 (10.0 g, 0.06610 mol) in acetic acid, 200 ml of H_2O_2 (15 mL, 30 % aqueous) was added and then solution left stirring for 24 h at 60 °C. The

solvents were evaporated to produce a solid. The product was further purified by recrystallization from water to produce white solid **43** (10.8 g, 90%).

^{1}H NMR (D_2O) δ 2.23 (m, 0.22H), 3.00 (s, 3H), 3.28 (q, j = 14.8Hz, 2H) and 3.75 (m, 0.05H).

^{2}H NMR ($CDCl_3$) δ 2.24 (m, 2D) and 3.79 (m, 1D).

^{13}C NMR ($CDCl_3$) δ 37.9, 49.8, 79.0 (m) and 172.8.

DL-Methioninedioxide-d8 ***44:***

A 70 ml solution (D_2O as solvent) containing DL-methionine-sulfoxide-d_3 (7.0 g, 0.03804 mol), 1 g of pyridoxal hydrochloride and 5 ml of Al(III) D_2O solution ($Al(SO_4)_3$ $18H_2O$, 9.4 g dried at 70 °C under vacuum and dissolved in D_2O) was adjusted to pH 5.5 using NaOD (40% solution). The solution was refluxed for 24 h. The product was isolated at 0 ° C by raising the pH to 10 and then cooling and adding ethanol. The product was filtered, dried, dissolved in D_2O (200 mL) and then lyophilised to produce white solid **44** (4.9 g, 68%). Overall deuteration was found to be 90%D.

^{1}H NMR (D_2O) δ 2.23 (br s), 2.99 (m), 3.28 (m) and 3.77(m).

^{2}H NMR ($CDCl_3$) δ 2.26 (m, 1.9D), 3.03 (br s,2D), 3.33 (m, 2D) and 3.81 (br s, 1D).

^{13}C NMR ($CDCl_3$) δ 23.2 (s), 39.2 (m), 49.4 (m), 52.6 (m) and 172.6 (s).

^{13}C NMR ($CDCl_3$) {^{1}H} and {^{2}H} decoupled δ 23.1 (s), 39.3 (m), 49.6 (m), 52.6 (m) and 172.6 (s).

ESI-MS: m/z 188 [M^{-1}].

Synthesis and deuteration of D-phenyl glycine-d_6:

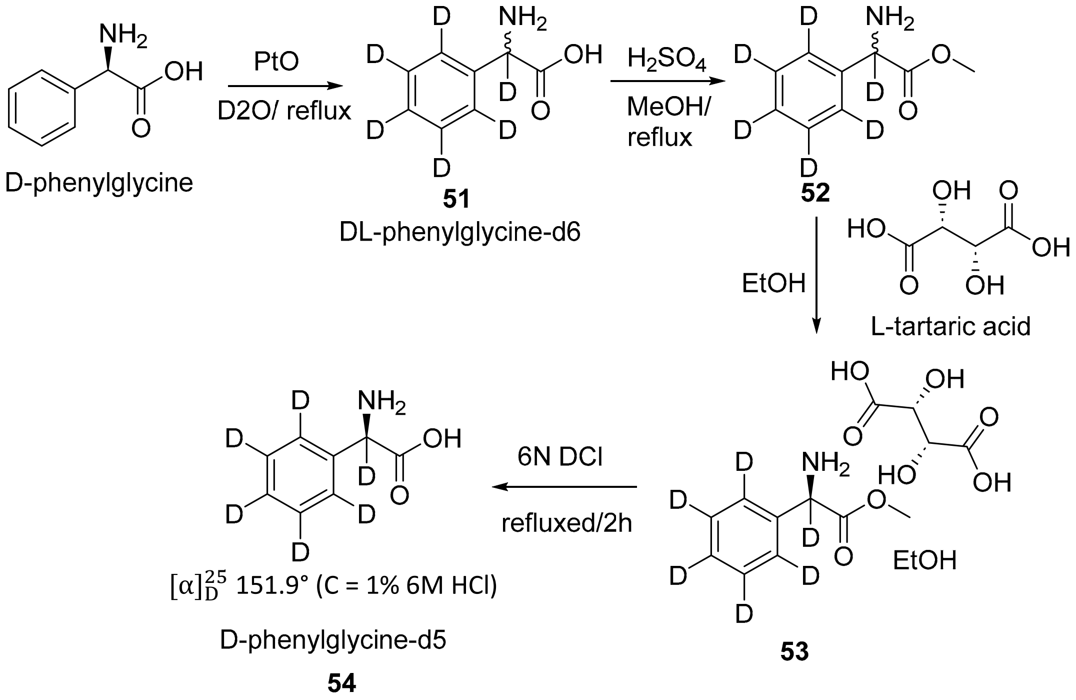

Deuteration of phenyl glycine-d_6 ***51:***

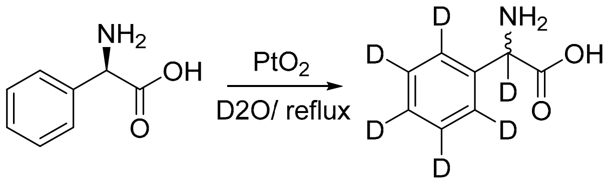

The solution of phenyl glycine (20.0 g, 0.1324 mol) and PtO_2 (previously treated with NaBH4, 0.8 g, 5 mol%) in D2O (150 mL) was heated to reflux for 24 h. After cooling the mixture filter through a Celite bed, the filtrate evaporated to produce a white solid, 18.8 g and 93% yield with 96%D.

^1H NMR (D_2O) δ 7.26 (m).

^2H NMR (D_2O) δ 7.26 (bs, 6) and 8.3 (bs).

^{13}C NMR (D_2O) δ 55.5 (m), 128.1 (m), 129.2 (m), 129.8 (m), 131.3 (s) and 170.0 (s).

^{13}C NMR (D_2O) {^1H} and {^2H} decoupled spectra δ 55.6, 127.4, 128.9, 129.66, 130.64 and 170.32.

ESI-MS: [M^{-1}] 156. 96% d level, Isotopic distribution 75.4% d_6; 18.5% d_5; and 6.2 % d_4.

Methyl phenyl glycenate-d_6 52:

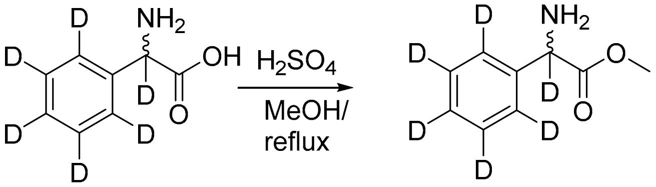

To a clear solution of DL-phenyl glycine-d_6 (18.0 g, 0.011464 mol) in methanol (150 mL) H_2SO_4 (5 mL) was added dropwise. The reaction refluxed for 24 h. After cooling, concentrated aqueous ammonia was added dropwise to the reaction solution until pH of about 8, while a large amount of white ammonium sulphate was precipitated in the reaction solution. Water (70 mL) was added to make the solution clear. Diethyl ether (5 x 50 mL) was extracted from the resultant solution. The combined organic layers were dried over MgSO4 and filtered. The solvent was evaporated to produce a colourless liquid (18.0 g, 98%).

^1H NMR (D_2O) δ 3.72 (s).

^2H NMR (D_2O) δ 7.46 (bs, D5) and 5.18 (m).

^{13}C NMR (D_2O) δ 54.2 (s), 56.1 (m), 127.6 (m), 129.2 (m), 130.0 (m), 130.7 (s) and 169.7 (s).

^{13}C NMR (D_2O) {^1H} and {^2H} decoupled spectra δ 54.2, 56.1, 127.6, 129.2, 130.0, 130.7 and 169.7.

Resolution of DL-phenylglycinate-d_6 53:

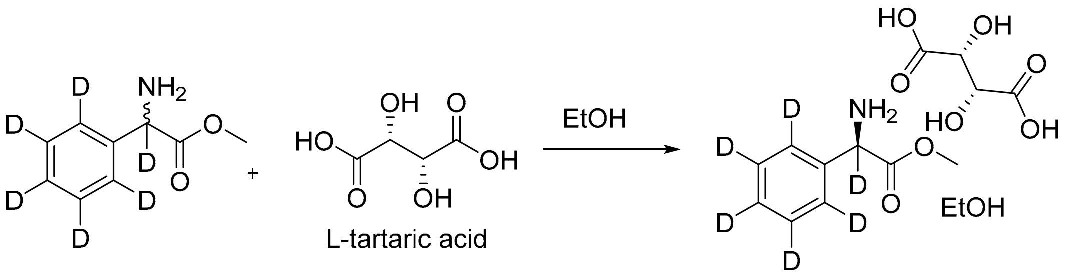

A solution of methyl DL-phenylglycinate-d_6 (10.0 g, 0.05844 mol) and (+)-tartaric acid (8.77 g, 0.05844 mol) in ethanol-H_2O (300 mL, 9 : 1 v/v) was heated at 50 °C. The solution was allowed to cool and then kept at 5 °C overnight. The crystallised product was filtered, and the product was washed with small amount of ethanol and dried at 20°C under reduced pressure to produce methyl D-phenylglycinate-d_6 hydrogen (+)-tartrate monoethanolate as white needles (9.0 g, 41%).

^{1}H NMR (D_2O) δ 1.06 (t, j = 7.85Hz, 3H, solvated ethanol), 3.53 (q, j = 7.85Hz, 2H, solvated ethanol), 3.70 (s, 3H), 4.41 (s, 2H) and 7.39 (m, 0.15H).

^{2}H NMR (D_2O) δ 7.39 (bs, D5) and 5.33 (m).

^{13}C NMR (D_2O) δ 16.8 (s), 54.2 (s), 56.1 (m), 57.5 (s), 72.7 (s), 127.6 (m), 129.2 (m), 130.0 (m), 130.7 (s), 169.7 (s) and 176.8 (s).

Hydrolysis of D-Phenylglycine-d_6 54:

A solution of methyl D-phenylglycinate-d_6 hydrogen (+)-tartrate (solvated monoethanolate) (9.0 g, 0.02452 mol) in 6N DCl (20 mL) was refluxed for 2 h; then contents were evaporated to minimum levels. The solution was diluted with water (30 mL) and neutralised to pH 7.0. The mixture was cooled on ice and filtered, and then the solid was washed with small amount of water to produce a white solid (3.56g, 86%). Optical rotation for the resolved D-phenyl glycine-d6 $[\alpha]_D^{25}$ was -151.9° (c, 1 in 6M HCl) reference $[\alpha]_D^{25}$ -154° (c, 1in 6M HCl). NMR spectral data is same as DL-phenyl glycine-d6.

Synthesis of perdeuterated L-phenylalanine-d_8 and L-tyrosine-d_7:

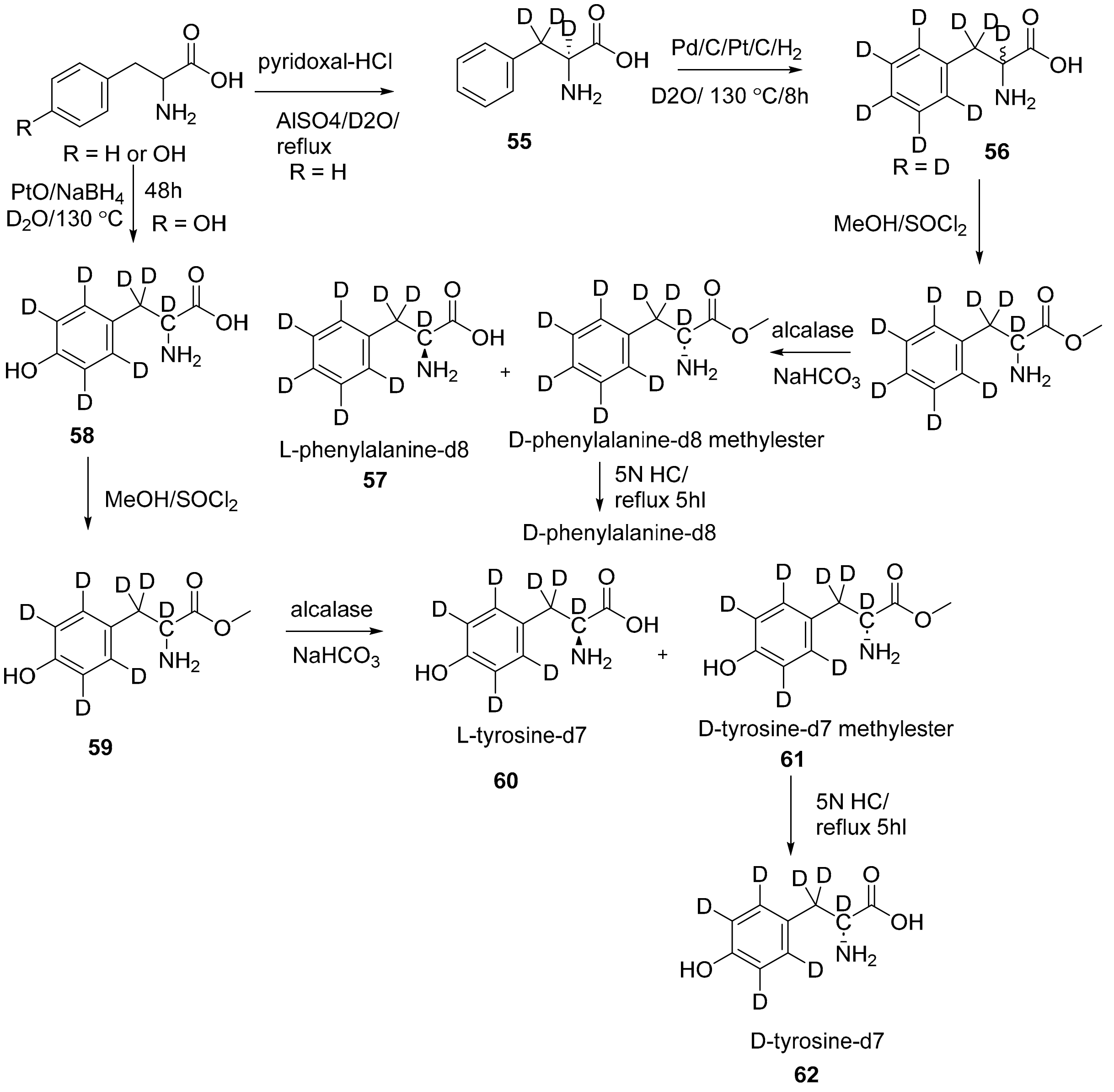

α,β-Deuteration of phenylalanine-d3 55:

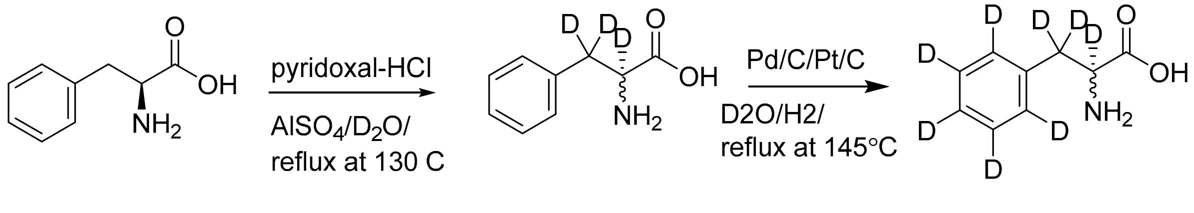

A 200 ml solution (D_2O as solvent) containing 20.0 g of L-phenylalanine, 4 g of pyridoxal hydrochloride and 90 ml of Al(III) D_2O solution ($Al(SO_4)_3$ $18H_2O$, 3.4 g, dried at 70 °C under vacuum and dissolved in 100mL D_2O) was adjusted to pH 5.5 using NaOD. The solution was refluxed at 130 °C for 24 h. The product was isolated at 0 ° C by raising the pH to 10 and then cooling and adding ethanol. The product was filtered, dried, dissolved in D_2O (200 mL) and then lyophilised to produce a white solid (14.5g, 72.5%) with 91%D.

^{1}H NMR (D_2O) δ 7.23-7.37 (m, 5H), 6.83 (s, 1H) and residual protons 3.11 (m, 0.01H).

^{2}H NMR (D_2O) δ 5.33 (m), 3.88 (m), 3.15 (m) and 299(m).

^{13}C NMR (D_2O) δ 171.12, 133.66, 129.32, 129.15, 127.15, 53.8 and 35.0.

ESI MS m/z : 191 $[M+Na]^+$ 91%D at α,β-positions.

Ring deuteration of phenylalanine-d$_8$ 56:

Phenylalanine-d$_3$ (7.0 g, 0.0416 mol), 10% Pt/C (1.0 g), 10% Pd/C (1.0 g) and D_2O (150 mL) were placed in a Schlenk round bottom flask and degassed by purging with N_2 gas followed by a balloon of H_2 gas. The reaction mixture was heated to reflux at 140 °C, with constant stirring for 8 h. After this time, the reaction was cooled to room temperature, and the reaction mixture was passed through a short plug of Celite® and washed with H_2O (100 mL) and then MeOH (100 mL). The mixture was evaporated to dryness to produce slightly coloured solid (4.8g, 68%), with overall isotopic purity of 89.4%D.

^{1}H NMR (D_2O) δ residual protons 7.52 (m), 3.91 (m), 3.19 (m) and 3.02 (m).

^{2}H NMR (D_2O) δ 7.38 (m), 3.88 (m), 3.88 (m), 3.16(m) and 2.99 (m).

^{13}C NMR (D_2O) δ 174.1, 135.0 (m), 129.25, 128.74 (m), 127.20, 55.8 (m) and 35.9 (m).

ESI MS m/z: 174 $[M+1]^+$ overall deuteration 89.3%D.

DL- phenylalanine-d$_8$ methyl ester 56a:

To a stirred solution of DL-phenylalanine-d8 (2.0 g, 0.0116 mol) in dry methanol (20 mL) at 0 °C, thionyl chloride (1.7 mL, 22 mmol, 2 mole equivalent) was added dropwise over 10 minutes. After complete addition, the reaction was allowed to adjust to ambient temperature and was stirred overnight. The reaction mixture was evaporated to dryness, and the solid obtained was dissolved in saturated sodium hydrogen carbonate and extracted into 20% isopropanol in chloroform (5 x 50 mL, or until no more product was detected in extract by TLC). Combined organics were dried with magnesium sulphate, filtered and reduced to obtain the product as a white powder. Yield of DL-phenylanaine-d8 methyl ester **56a** (2.0g, 87%).

^{1}H NMR ($CDCl_3$) δ residual protons 7.25 (m, 0.19H), 3.74 (s, 3H) and 3.17 (m, 0.15H).

^{2}H NMR ($CDCl_3$) δ 7.33 (m), 3.88 (m) and 3.15 (m).

Enzymatic resolution of DL-phenylalanine-d$_8$ 57:

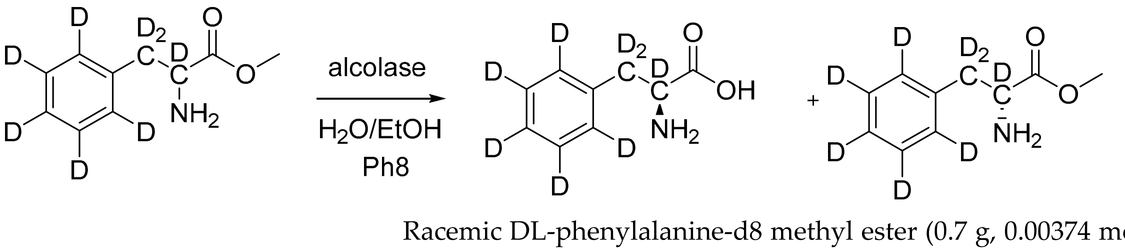

Racemic DL-phenylalanine-d8 methyl ester (0.7 g, 0.00374 mol) was dissolved in 40 mL of 0.2 mol $NaHCO_3$ solution. BL-Alcalase (20 mg PPL) was added to the mixture. The reaction was gently stirred at 38 °C for 25 minutes under nitrogen atmosphere. The completion was confirmed by mass spectral analysis, showing 50:50 mixtures of free acid and ester. The reaction mixture was passed through Celite bed to remove enzyme. The filtrate was extracted with ethyl acetate (5 x 50 mL) and left-over aqueous with a reduced volume, adjusted pH to 7. The neutral solution was left overnight at 4 °C to obtain white precipitate, which was filtered and dried to obtain L-phenylalanine-d8 as white solid **57** (0.25g, 77% based on the 50% isomer), with 89%D. L-phenylalanine-d8 showed an optical rotation, $[\alpha]_D^{23}$ -27.0° (c, 1 in H_2O) (ref $[\alpha]_D^{25}$ -30.0° (c, 1 in H_2O)). ^{1}H NMR and ^{13}C NMR data were identical to that of DL-phenylalanine-d8 **55**.

D-Phenylalanine-d_8: Combined organic extracts were dried over MgSO4 and evaporated to white solid, which was further recrystalised from ethyl acetate to give white crystalline solid (0.3g). The solid was hydrolysed in 5N DCl reflux conditions for 5 h. After cooling reaction, contents were evaporated to produce coloured dry residue. To this, 30 ml DCM and 200 µL Et_3N were added under stirring at rt; after stirring for 10 minutes, 4mL of water was added to form white precipitate. White solid was filtered and washed with 3 mL of cold water and dried under vacuum to obtain white solid (0.15g, 46% of 50% D-isomer) with 89%D. D-phenylalanine-d_8 showed an optical rotation, $[\alpha]_D^{23}$ +29.0° (c, 1 in H_2O) (ref $[\alpha]_D^{27}$ + 33.0° (c, 1 in H_2O) Sigma Aldrich catalogue). ^1H NMR and ^{13}C NMR data were identical to that of DL-phenylalanine-d_8 55.

Deuteration of DL-tyrosine-d_7 58:

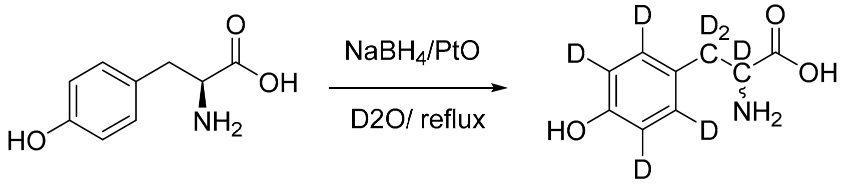

The Platinum Oxide (Adams catalyst) was activated according to Calf and Garnett's procedure. A suspension of Platinum Oxide (0.5 g) in Deuterium Oxide (100 mL) was extensively degassed by bubbling dry nitrogen whilst stirring. To the degassed solution Sodium Borohydride (2.0 g) was added incrementally while stirring. Once the reaction was complete, the flask was placed under positive nitrogen pressure to limit contact of solution with atmospheric oxygen. Next this flask was gently heated to 70°C for 15 minutes to hydrolyse any remaining Sodium Borohydride.

In a separate flask, a solution of L-tyrosine (2.0 g) was prepared in Deuterium Oxide (30 mL) and 40 wt% Sodium Deuteroxide (3 mL, 2 molar equivalents) and extensively degassed in a similar fashion to the catalyst suspension. This solution was added under inert atmosphere via needle to the prepared catalyst suspension and the complete reaction mixture was set to reflux under nitrogen flow for two days. The reaction mixture was filtered through a Celite plug to remove platinum catalyst and then allowed to cool to room temperature. The mixture was reduced to about 50 mL under vacuum distillation and transferred to a conical flask with a stirrer bar. The solution was neutralised by careful addition of 5M hydrochloric acid while stirring vigorously and set to cool in 4 °C fridge overnight. Resulting crystals were collected and washed with cold water, methanol and ether (in that order), yielding DL-phenylanaine-d8 **58** (1.5g, 75%) with 88%D.

^{1}H NMR (D_2O) δ residual protons 6.76 (m), 6.55 (m), 3.31 (m) and 2.83 (m).

^{2}H NMR (D_2O) δ 7.15 (m, ND2), 6.45 (m), 6.15 (m), 3.88 (m), 3.52 (m) and 2.42 (m).

^{13}C NMR (D_2O) ({^{1}H}&{^{2}H} decopouled spectra) δ 170.1, 154.3,129.9, 124.7, 114.8, 53.3 and 33.3.

ESI MS m/z: 189 $[M+1]^+$ overall deuteration 88.4%D.

DL- tyrosine-d_7 methyl ester 59:

To a stirred solution of DL-tyrosine-d_7 (3.0 g, 0.0159 mol) in dry methanol (20 mL) at 0 °C, thionyl chloride (1.7 mL, 22 mmol, 2 mole equivalent) was added dropwise over 10 minutes. After complete addition, the reaction was allowed to adjust to ambient temperature and was stirred overnight. The reaction mixture was evaporated to dryness, and the solid obtained was dissolved in saturated sodium hydrogen carbonate and extracted into

20% isopropanol in chloroform (5 x 50 mL, or until no more product was detected in extract by TLC). Combined organics were dried with magnesium sulphate, filtered and reduced to obtain the product as a white powder, yielding DL-tyrosine-d_7 methyl ester **59** (3.2g, 99%).

^{1}**H NMR** ($CDCl_3$) δ residual protons 6.99 (m, 0.07H), 6.55 (m, 0.24), 3.69 (s, 3H), 3.48(m, 0.03H) and 2.85 (m, 0.14H).

^{2}**H NMR** ($CDCl_3$) δ 7.03 (m), 6.74 (m) and 2.84 (m).

Enzymatic resolution of DL-tyrosine-d_7 60:

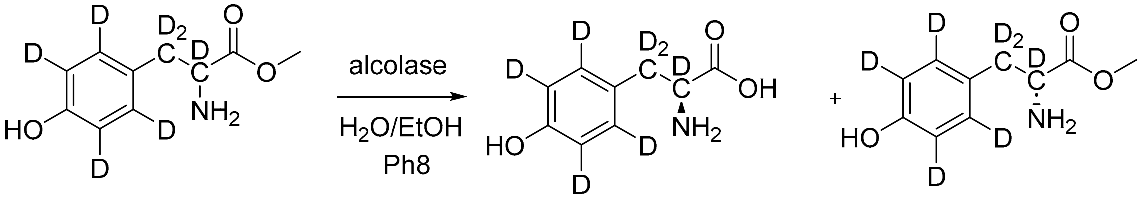

Racemic DL-tyrosine-d_7 methyl ester (2.0 g, 0.00099 mol) was dissolved in 50 mL of 0.2 mol $NaHCO_3$ solution. BL-Alcalase (60 mg PPL) was added to the mixture. The reaction was gently stirred at 30 °C for 25 minutes under nitrogen atmosphere. The completion was confirmed by mass spectral analysis, showing 50:50 mixtures of free acid and ester. The reaction mixture was passed through Celite bed to remove enzyme. The filtrate was extracted with ethyl acetate (5 x 50 mL), left-over aqueous reduced volume minimum and pH was adjusted to 7. The neutral solution was left in the fridge at 4 °C overnight to produce precipitate, which was filtered and washed with a little amount of cold water and dried to obtain L-tyrosine-d_7 as a white solid (1.0 g, 100% of 50% L-isomer) with 88%D: $[\alpha]_D^{23}$ -9.98° (c, 2 in 1M HCl) ($[\alpha]_D^{27}$-9.1° (c, 2 in 1M HCl)). ^{1}H NMR and ^{13}C NMR data were identical to that of DL-tyrosine-d7 **58**.

D-Tyrosine-d_7: Above combined organic extracts were dried over MgSO4 and evaporated to white solid, which was further recrystalised from ethyl acetate to obtain white crystalline solid (0.8g). The solid was hydrolysed in 5N DCl reflux conditions for 5 h. After cooling reaction, contents were evaporated to produce coloured dry residue. To this, 50 ml of DCM and 400 μL of Et_3N were added under stirring at rt, and after stirring for 10 minutes, 6mL of water was added to form white precipitate. White solid was filtered and washed with 5 mL of cold water and dried under vacuum to produce white solid **62** (0.5g, 50% of 50% D-isomer) with 88%D. $[\alpha]_D^{23}$ +9.0° (c, 2 in 1M HCl). ^{1}H NMR and ^{13}C NMR data were identical to that of DL-tyrosine-d7 **58**.

Synthesis of deuterated of L-2,4-diaminobuteryc acid-d5 DAB-d_5:

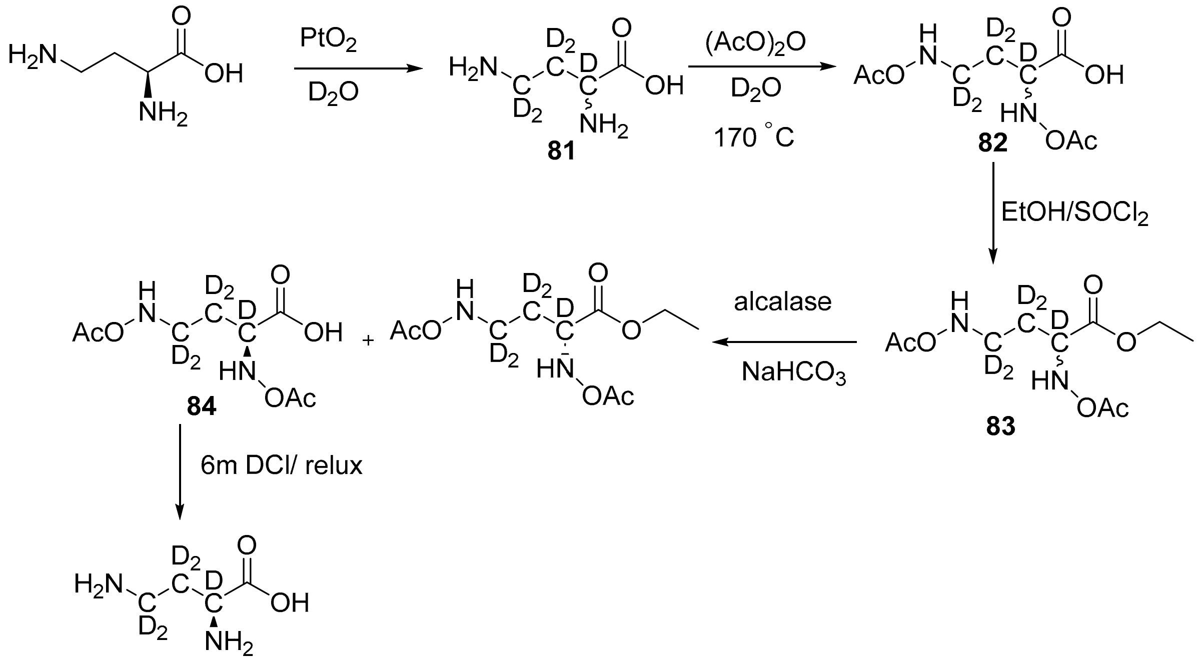

L-2,4-Diaminobutyric acid-d5

81a

Deuteration of DL-DAB-d_5 81:

The solution of 2,4-diaminobuteryc acid (2 x HCl salt) (3.0 g, 0.00254 mol) and PtO_2 (previously treated with NaBH4, 0.03 g, 10 mol%) in D_2O (150 mL) was heated to reflux for 24 h. After cooling, the mixture was filtered through a Celite bed, and the filtrate was evaporated to obtain a white solid, 3.0 g and 100% yield with 92%D.

^{1}H NMR (D_2O) residual protons δ 1.75 (m), 2.67 (s), 1.98 (m, 0.14H) and 3.8 (s).

^{2}H NMR ($CDCl_3$) δ 2.00 (m), 2.33 (m) and 3.20 (m).

^{13}C NMR ($CDCl_3$) δ 37.7 (m), 54.08 (m), 28.6 (m), 57.03 (s) and 163.7 (s).

ESI-MS: m/z 122 [M^{-1}]. Overall 92%D.

DL-2,4-bis(acetoxyamino)butanoic acid-d_5 (N,N′-diaceto-DAB-d5) 82:

To a solution of DAB-d5(2.0g, 0.00725 mol) in D_2O (24 mL, 1.11 mol), after purging the solution with N2, acetic anhydride (Ac_2O, 20 mL, 194 mol) was added dropwise over 1h with an addition funnel. The reaction mixture was heated to 170 ^{0}C for 10 min and allowed to cool. The solvents were removed in vacuo, and the residue was purified by flash column chromatography (40:60 MeOH/CH_2Cl_2). producing 2,4-bis(acetoxy-amino)butanoic acid as a sticky white solid (2.0 g, 86.9%).

^{1}H NMR (D_2O) residual protons δ 1.75 (m, 0.5H), 1.85 (s, 3H), 1.91 (s, 3H), 3.11 (m, 1.1H) and 4.02 (m, 0.4H).

^{2}H NMR ($CDCl_3$) δ 1.88 (m), 3.17 (m) and 4.14 (m).

^{13}C NMR ($CDCl_3$) δ 21.2 (s), 23.3 (s), 31.6 (m), 36.0 (s), 52.1 (s), 173.7 (s), 173.95 (s) and 181 (s).

ESI-MS: [M^{-1}]: 238

DL-2,4-bis(acetoxyamino)butanoic ethylester-d5 (N,N′-diaceto-DAB-d5 ethylester) 83:

To a cold solution (-10°C) of 2,4-bis(acetoxyamino)butanoic acid (2.0 g, 0.0097 moles) in ethanol-d_1 (30 ml), thionyl chloride (2 mL) was added dropwise. The resulting solution was then warmed to rt for 4 hrs. Then, 20 mL of water and 15 mL solution of 0.8 g of $NaHCO_3$ were added. The pH of the solution was adjusted to 7 by addition of a dilute

NaOH solution. The product was transferred to organic phase by extraction with ethyl acetate (4 x 50 mL). Combined extracts were dried over $MgSO_4$, filtered and evaporated to produce white solid, which was further recrystallised from diethyl ether to white powder **83** (1.8 g, 78.8%).

^{1}H NMR (D_2O) residual protons δ 1.14 (t, J = 7.36 Hz, 3H), 1.81 (m, 0.7H), 1.86 (s, 3H), 1.95 (s, 3H), 3.11 (m, 1.1H), 4.09 (m, 2H) and 4.26 (0.6H).

^{2}H NMR ($CDCl_3$) δ 1.88 (m) and 3.10 (m).

^{13}C NMR ($CDCl_3$) δ 13.15 (s), 21.48 (s), 21.78 (s), 35.46 (m), 50.58 (m), 62.27 (s), 173.7 (s), 174.00 (s) and 174.33 (s).

ESI-MS: [M^{+1}]: m/z 268.

L-2,4-bis(acetoxyamino)butanoic acid-d$_5$ 84:

To a suspension of racemic diacetyl amino acid ester, 1.0 g was in 60 ml mixed solvent of water (85% v/v), ethanol was added $NaHCO_3$ (0.5 g), while the pH of the solution was about 8. BL-Alcalase (0.25 g) was added to the mixture, and then the reaction mixture was gently stirred at 25 °C for 24 hrs under a nitrogen atmosphere. The reaction was filtered through sintered funnel, and filtrate was further basified with $NaHCO_3$ solution. Now the N-acetyl D-ester was extracted by ethyl acetate (50 mL x 3). Left-over aqueous portion was neutralised with 1M HCl until pH 6-7, then extracted with ethyl acetate (50 mL x 3), and combined organic layers were dried over NaSO4 and evaporated to produce L-2,4-bis(acetoxyamino) butanoic acid as pale-yellow solid (0.4g, 91%) with 92%D. $[\alpha]_D^{25}$ -24.09° (c, 10 in H2O).

Hydrolysis of L-2,4-bis(acetoxyamino)butanoic acid-d$_5$ to L-DAB-d5 81a:

A solution of L-2,4-bis(acetoxyamino)butanoic acid-d$_5$ (0.4 g, 0.00167 mol) in 6N DCl (20 mL) was refluxed for 3h; then contents were evaporated to obtain pale-yellow-coloured solid (0.23 g, 69%). Optical rotation for the resolved 2,4-diaminobutyric acid-d$_5$ as HCl salt with 92%D was $[\alpha]_D^{25}$ 15.9° (c, 4 in H_2O), reference $[\alpha]_D^{25}$ 14.5° (c ,3.7 in H_2O, Sigma Aldrich catalogue). NMR spectral data is same as **81**.

Deuteration of azetidine-2carboxylic acid-5 (AZE-d5):

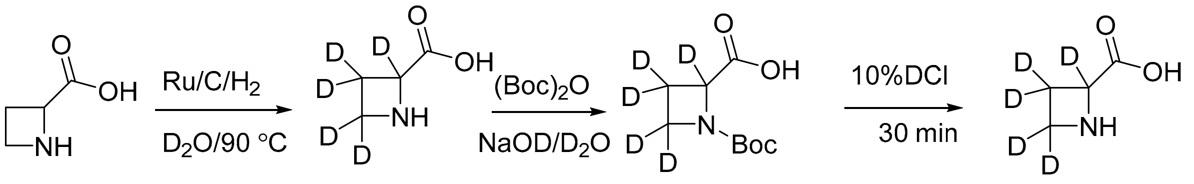

Azetidine-2-carboxylic acid (2.0 g, 0.0198 mol), 20% Ru/C (0.4 g) and D_2O (100 mL) were placed in a Schlenk round bottom flask and degassed by purging with N_2 gas followed by a balloon of H_2 gas. The reaction mixture was heated to reflux at 90 °C, with constant stirring for 3 days. After this time, the reaction was cooled to room temperature, and the reaction mixture was passed through a short plug of Celite® and washed with D_2O (20 mL). The mixture was evaporated to dryness to produce dark-coloured solid (1.4 g, 70%, 38%D). This solid was subjected to second cycle with above conditions with overall isotopic purity of 59.9%D and yield of 1.0g (50%). Pure compound was isolated through N-Boc derivative.

N-Boc-azetidine-2carboxylic acid-5

Crude dark-coloured AZE-d5 (0.7g, 0.0066 mol), di-tert-butyl dicarbonate (1.72 g, 0.0082 mol) and 0.5 mL of 40% NaOD were added to methanol-d (20 mL) and D2O (10

mL) at °0 C. The mixture was stirred overnight at ambient temperature. After evaporation of the methanol, D_2O (20 mL) was added then acidified with diluted HCl to a pH of 3 and extracted with ethyl acetate (50 mL × 3). The combined ethyl acetate was washed with water (30 mL) and saturated NaCl (30 mL) and dried over Na_2SO_4. Solvent was evaporated to obtain dark-coloured residue, which is further purified with column chromatography using 30% EtOAc in hexane to produce colourless liquid (0.7g, 51.4%)

^{1}H NMR ($CDCl_3$) δ 1.45 (s, 9H), 2.44 (m, 1.87H), 3.86 (m, 0.54H) and 4.74 (m, 0.093H).

^{2}H NMR ($CDCl_3$) δ 3.99 (m, 2D) and 4.82 (m, 1D).

^{13}C NMR ($CDCl_3$) δ 19.51 (m), 28.4 (s), 46.6 (m), 60.7 (m), 158.2 (m) and 172.6 (m).

ESI-MS: m/z 205 [M^{-1}] overall deuteration level 50%D, isotopologue distribution 5.5% *d*1, 46.2% *d*2, 40.2% *d*3, 6.0% *d*4 and 2.0% *d*5.

Azetidine-2-carboxylic acid-d5:

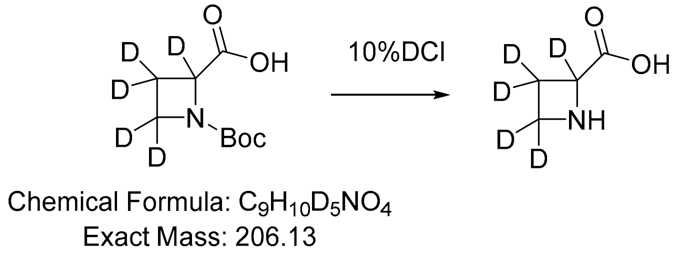

A solution of N-Boc-azetidine-2-carboxylic acid-d5 (0.7g, 0.0034 mol) in 10 ml of 10% DCl was stirred for 30 min. Solvent was removed under reduced pressure to obtain colourless gum (0.48 g, 99%) as HCl salt.

^{1}H NMR (D_2O) δ 2.06 (m, 1H), 2.32 (m, 0.97H), 3.16 (m, 0.55H) and 3.91 (m, 0.059H).

^{2}H NMR (D_2O) δ 1.75 (m), 2.14 (m), 2.98 (m) and 3.68 (m).

^{13}C NMR (D_2O) δ 22.40 (m), 43.5 (m), 57.4 (m) and 171.3 (s).

^{13}C NMR (D_2O) {^{1}H} and {^{2}H} decoupled spectra δ 25.50 (m), 42.2 (m), 58.9 (m) and 182.8 (s).

ESI-MS: m/z 107 [M^{-1}]. Overall deuteration level 59.49%, isotopologue distribution 3.39% *d*1, 33.4% *d*2, 33.01% *d*3, 22.76% *d*4 and 7.44% *d*5.

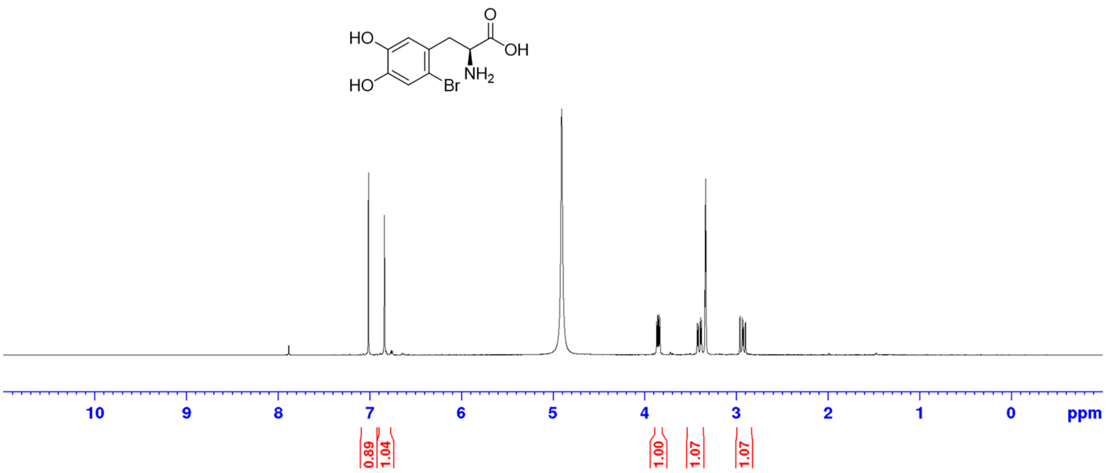

Figure S1. ^{1}H NMR spectrum of L-6-Br-DOPA **S15** in CD_3OD.

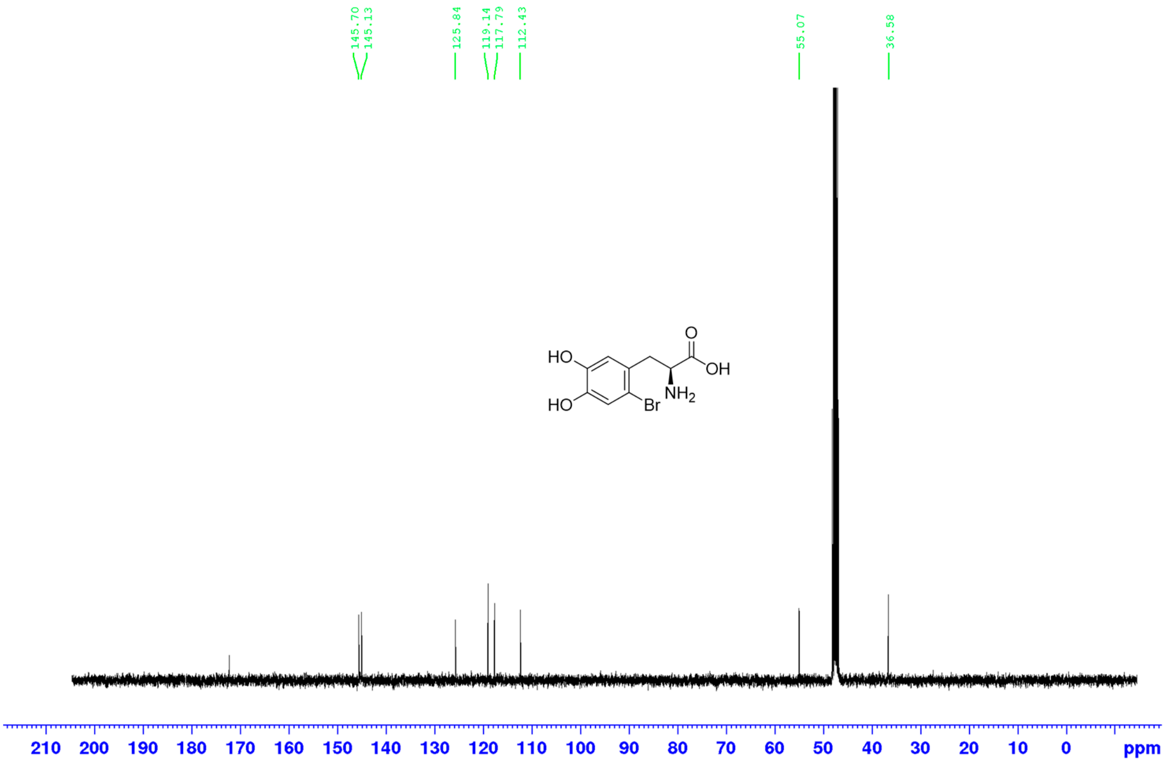

Figure S2. ^{13}C NMR spectrum of L-6-Br-DOPA **S15** in CD_3OD.

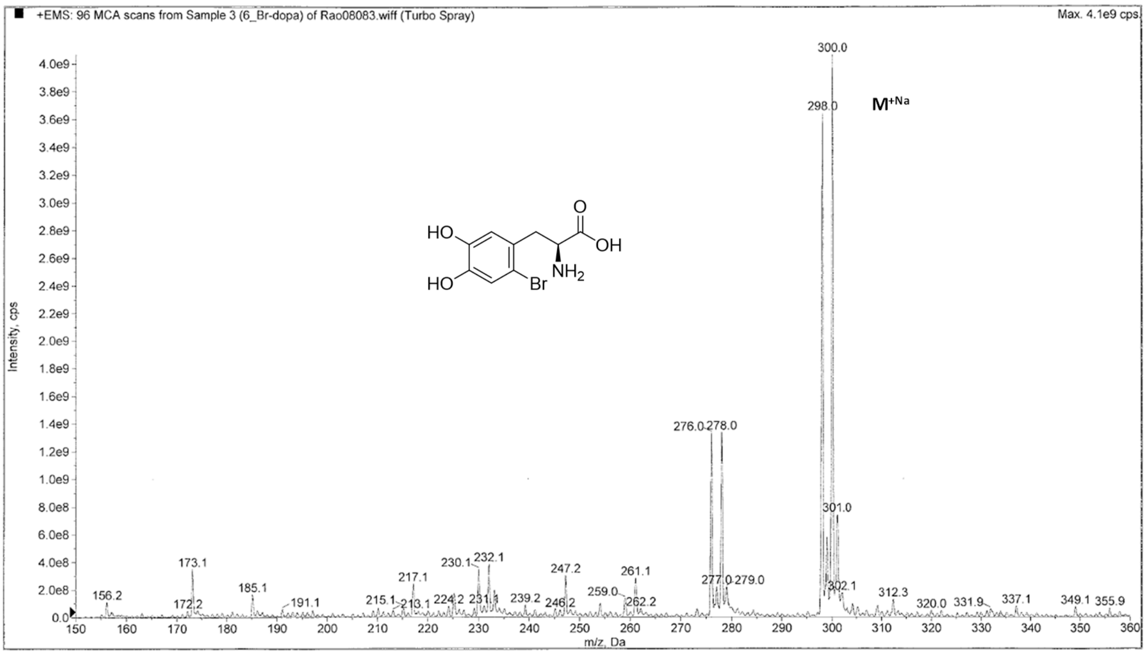

Figure S3. ESI-MS (Br^{79}) L-Br-DOPA **S15** m/z 300 M^{+Na}.

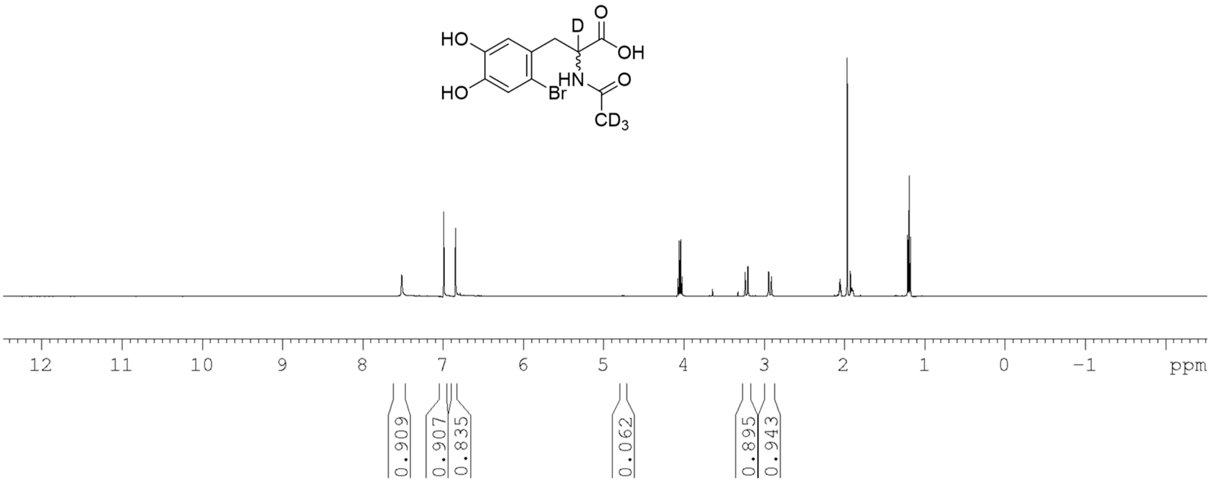

Figure S4. ^{1}H NMR spectrum of L-6-Br-DOPA-d_1 **S16** in CD_3OD.

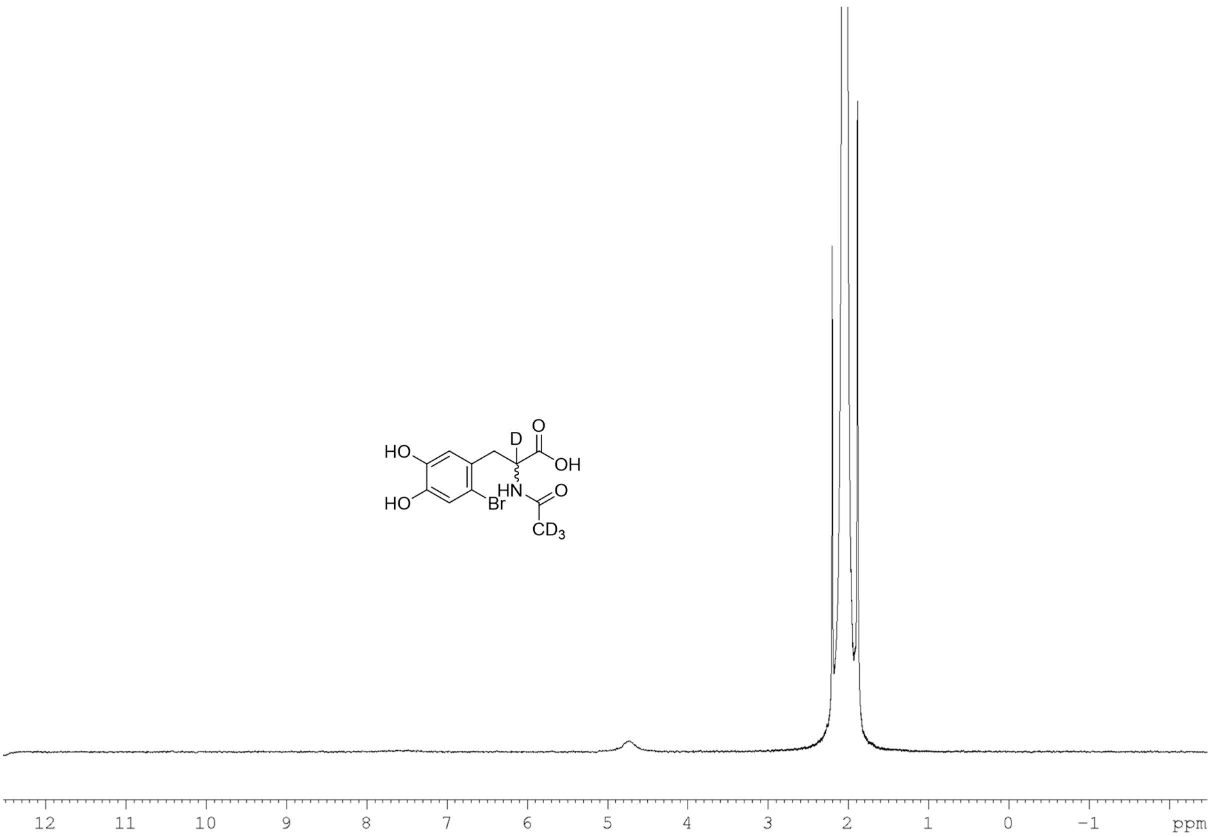

Figure S5. ^{2}H NMR spectrum of L-6-Br-DOPA-d$_1$ **S16** in CD_3OD.

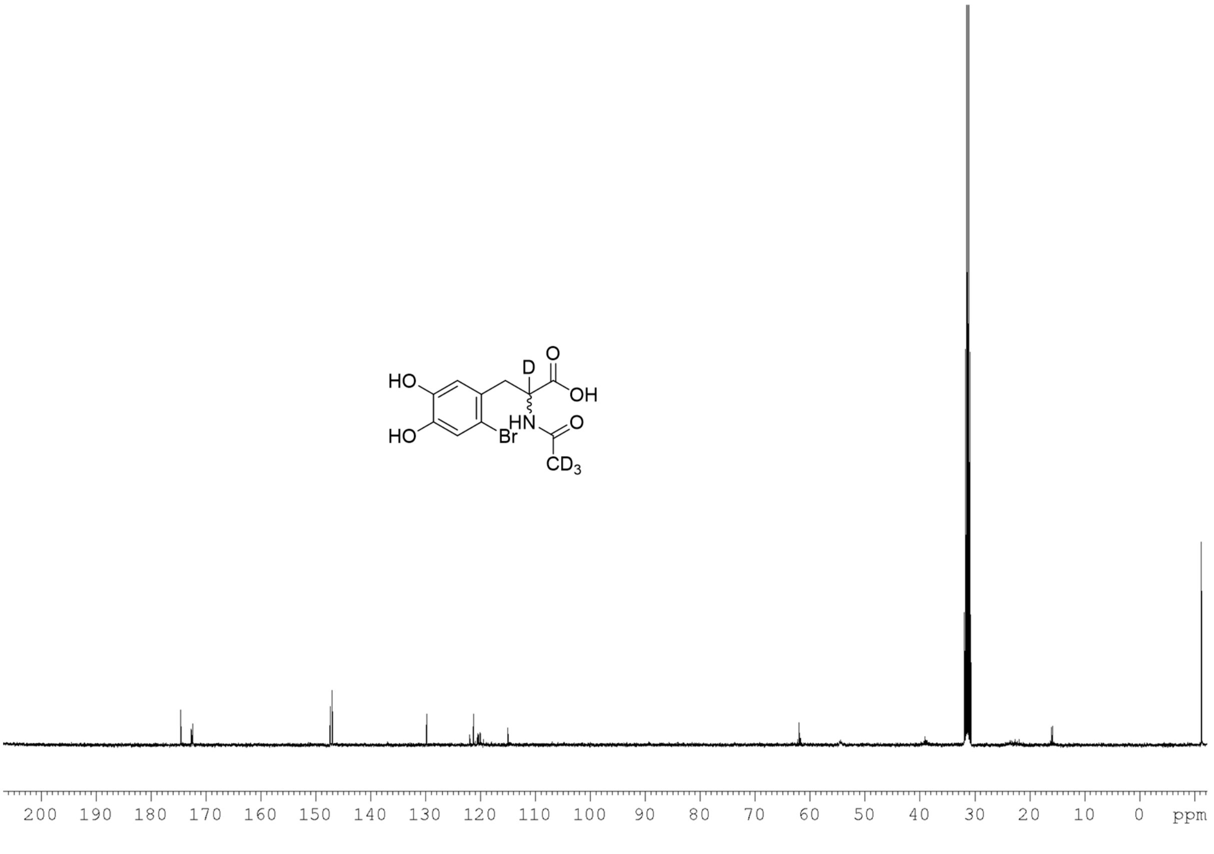

Figure S6. ^{13}C NMR spectrum of L-6-Br-DOPA-d$_1$ **S16** in CD_3OD.

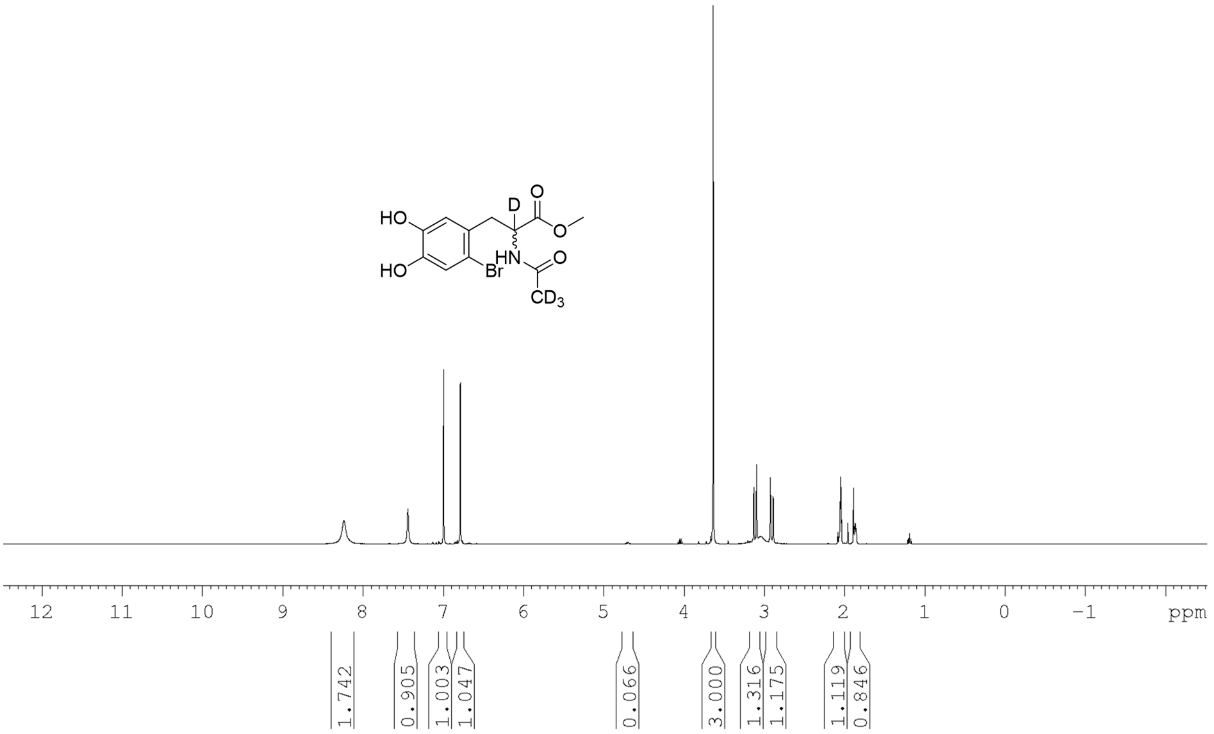

Figure S7. ^{1}H NMR spectrum of **S18** in $(CD_3)_2CO$.

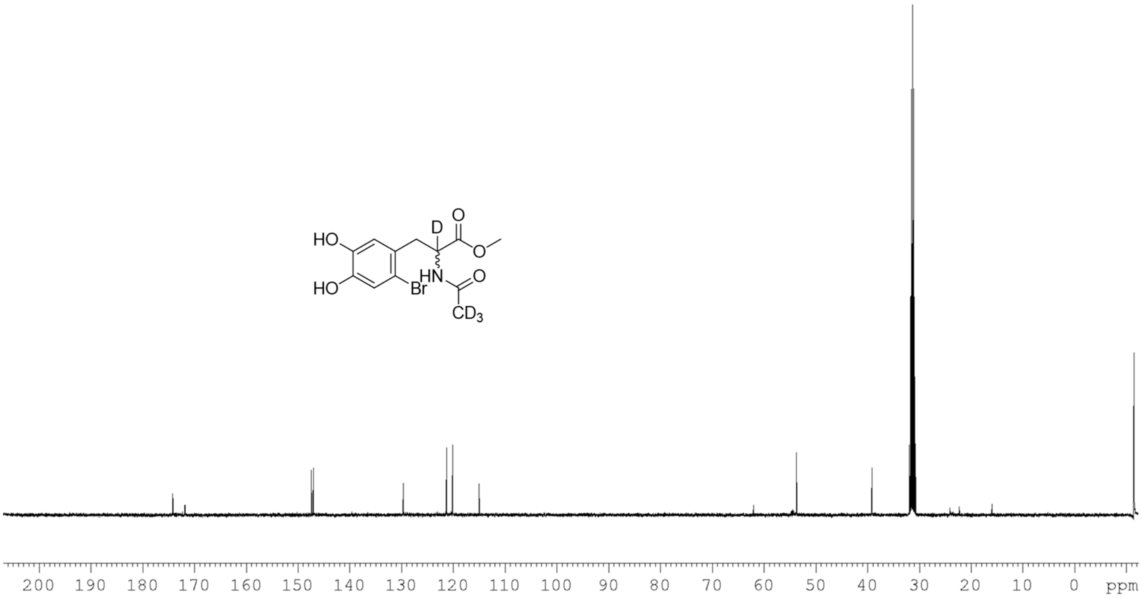

Figure S8. ^{13}C NMR spectrum of **S18** in $(CD_3)_2CO$.

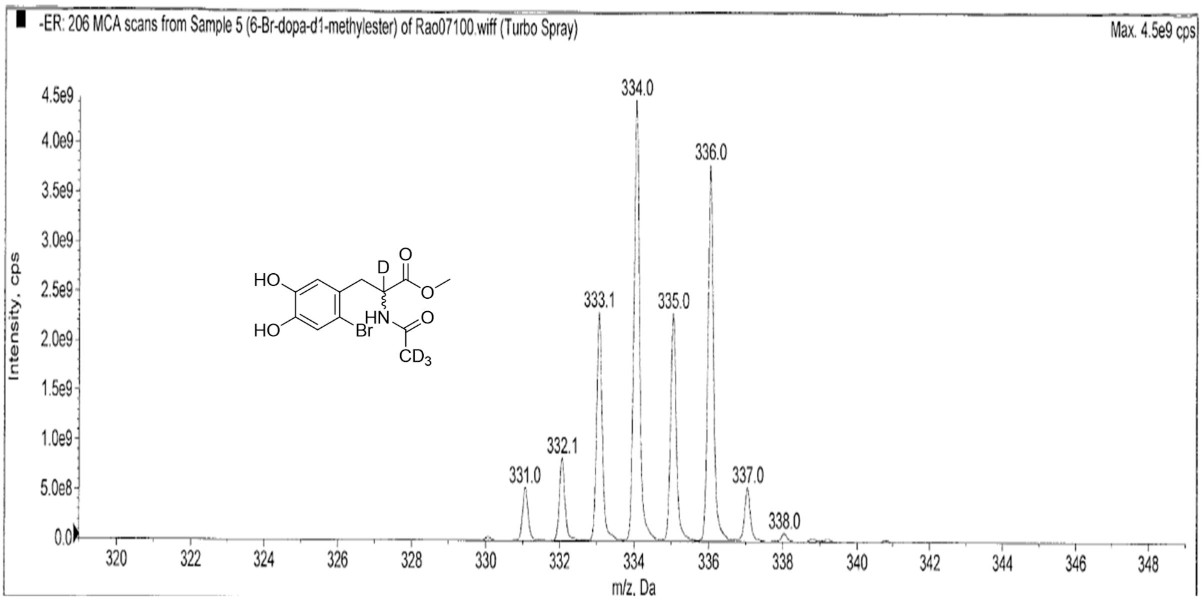

Figure S9. ESI-MS mass spectrum of **S18** m/z (Br^{79}) 334 M^{+Na}.

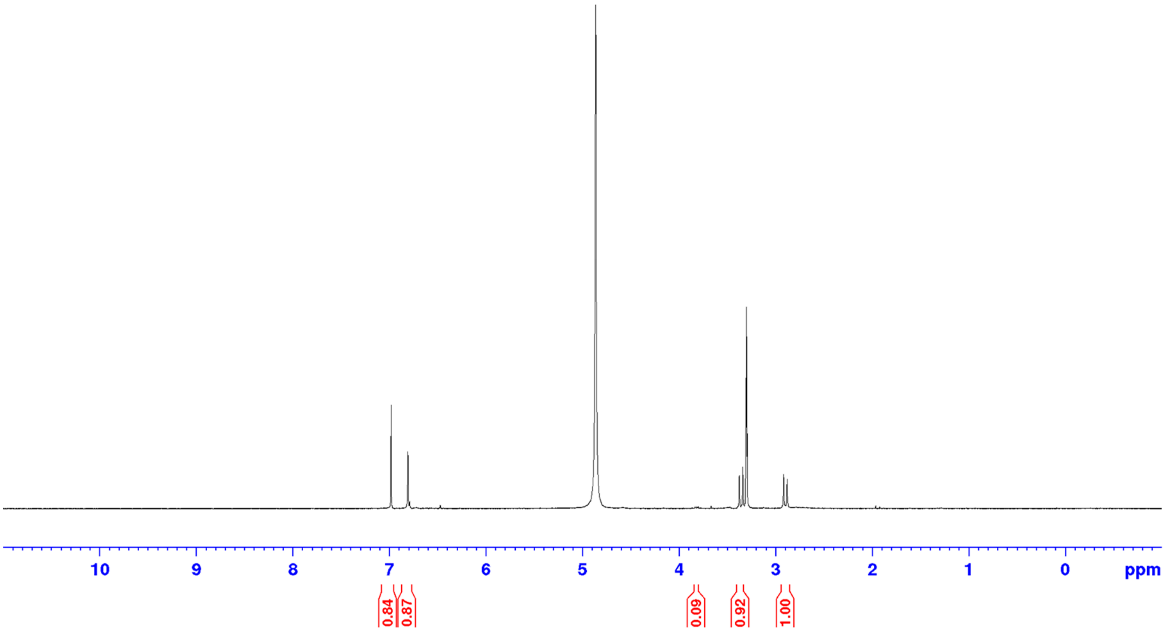

Figure S10. ^{1}H NMR spectrum of **S23** in CD_3OD.

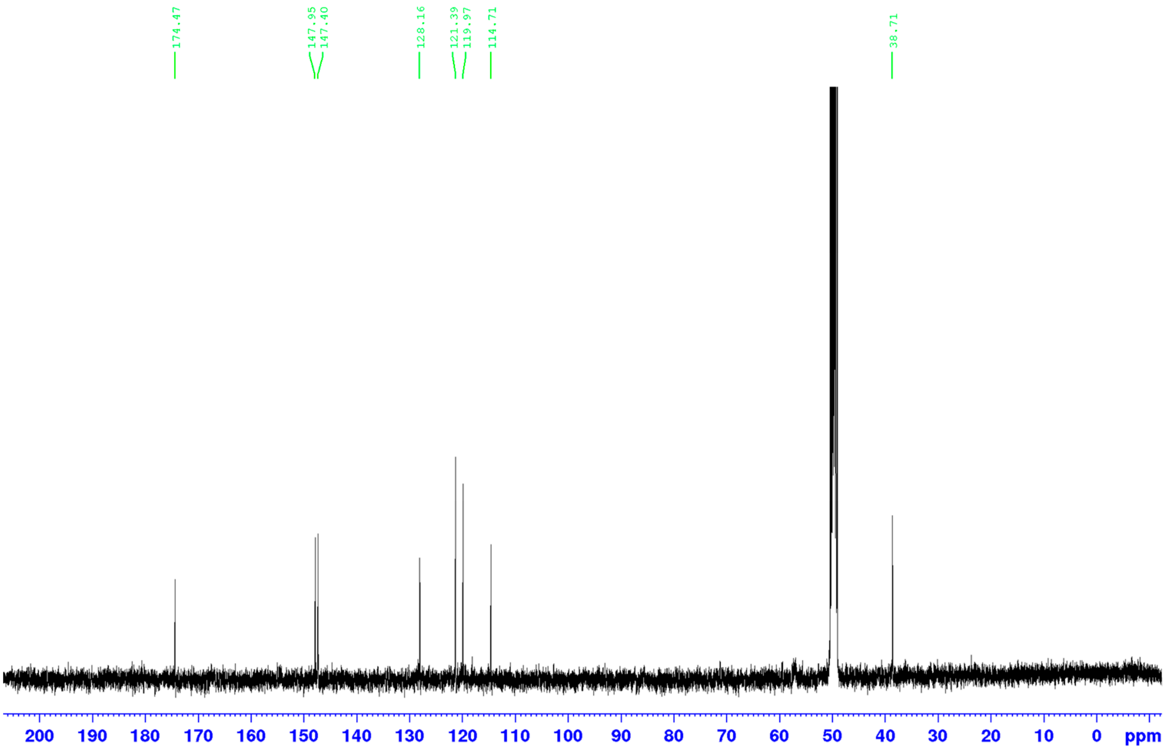

Figure S11. ^{13}C NMR spectrum of **S23** in CD_3OD.

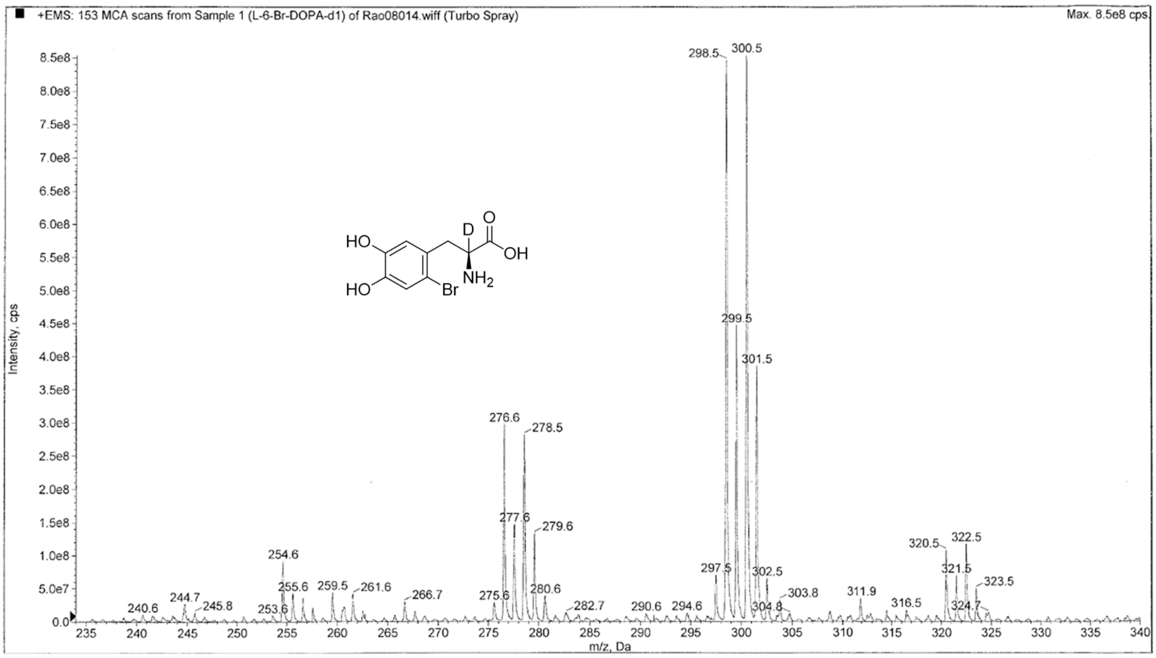

Figure S12. ESI-MS mass spectrum of (Br^{81}) **S23** m/z 300 M^{+Na}.

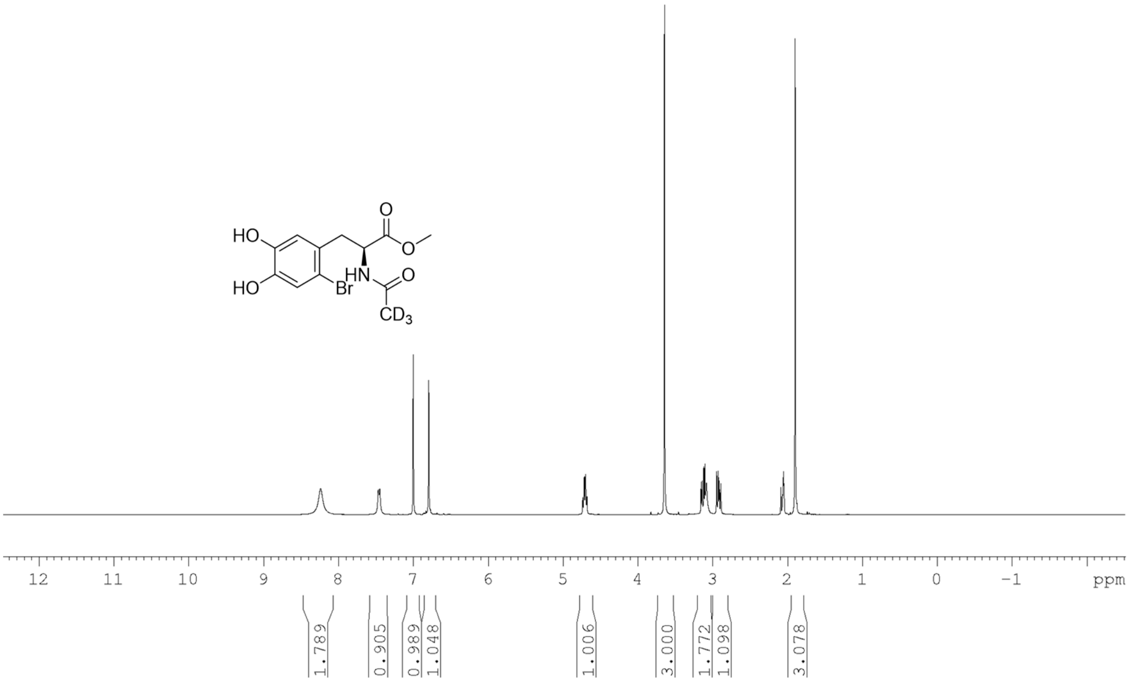

Figure S13. ^{1}H NMR spectrum of **S20** in $(CD_3)_2CO$.

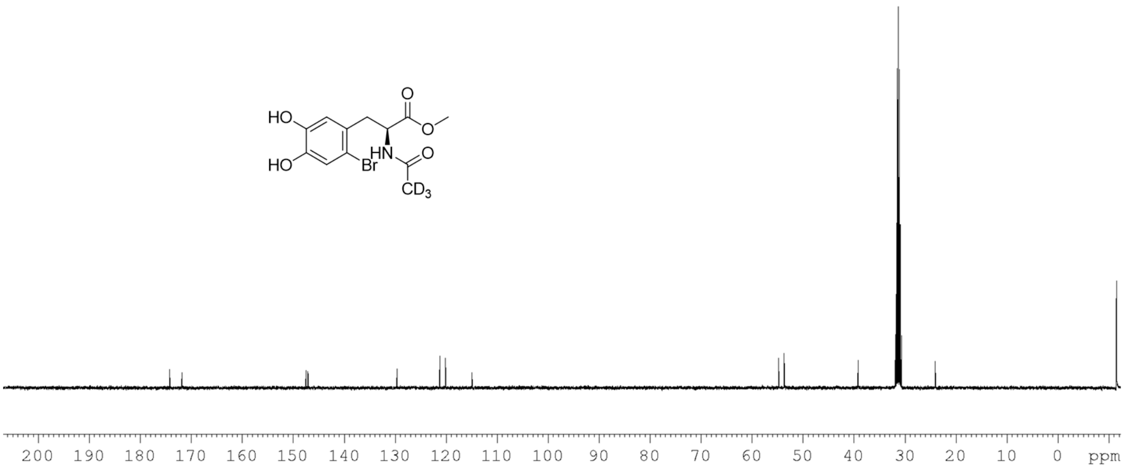

Figure S14. ^{13}C NMR spectrum of **S20** in $(CD_3)_2CO$.

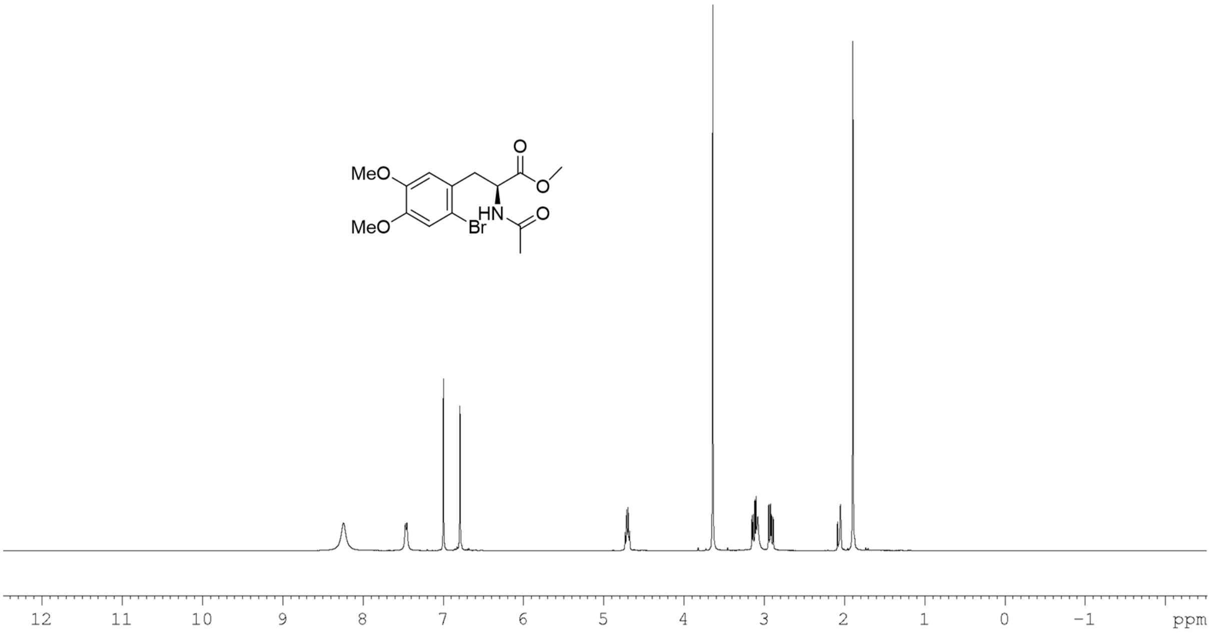

Figure S15. ^{1}H NMR spectrum of **S20** in $CDCl_3$.

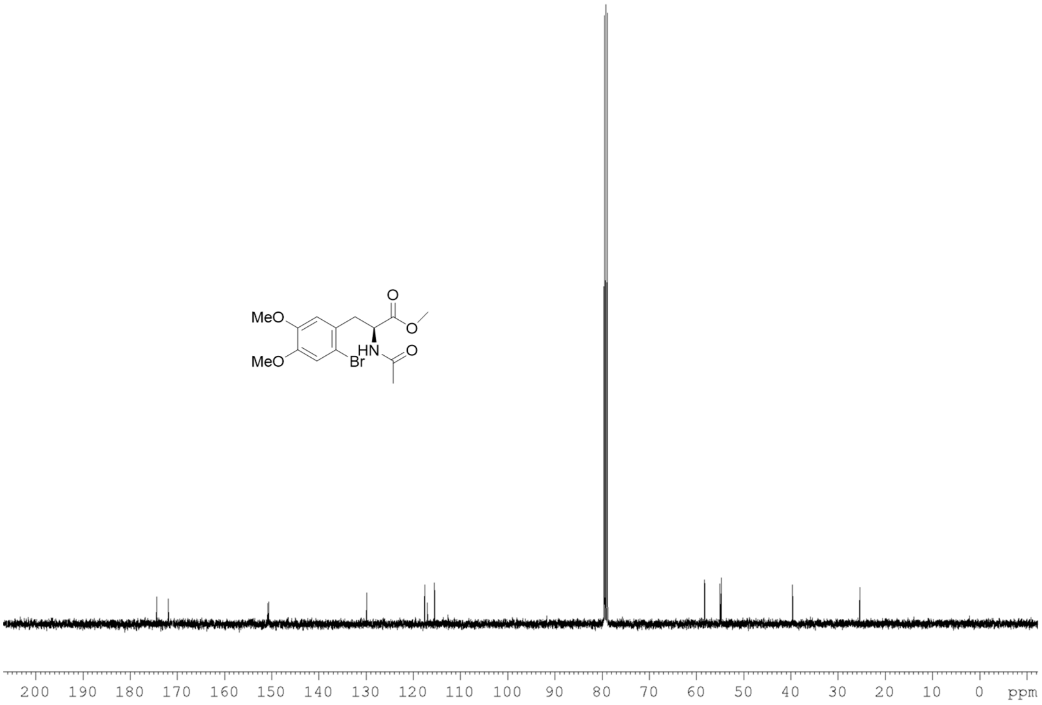

Figure S16. ^{13}C NMR spectrum of **S20** in $CDCl_3$.

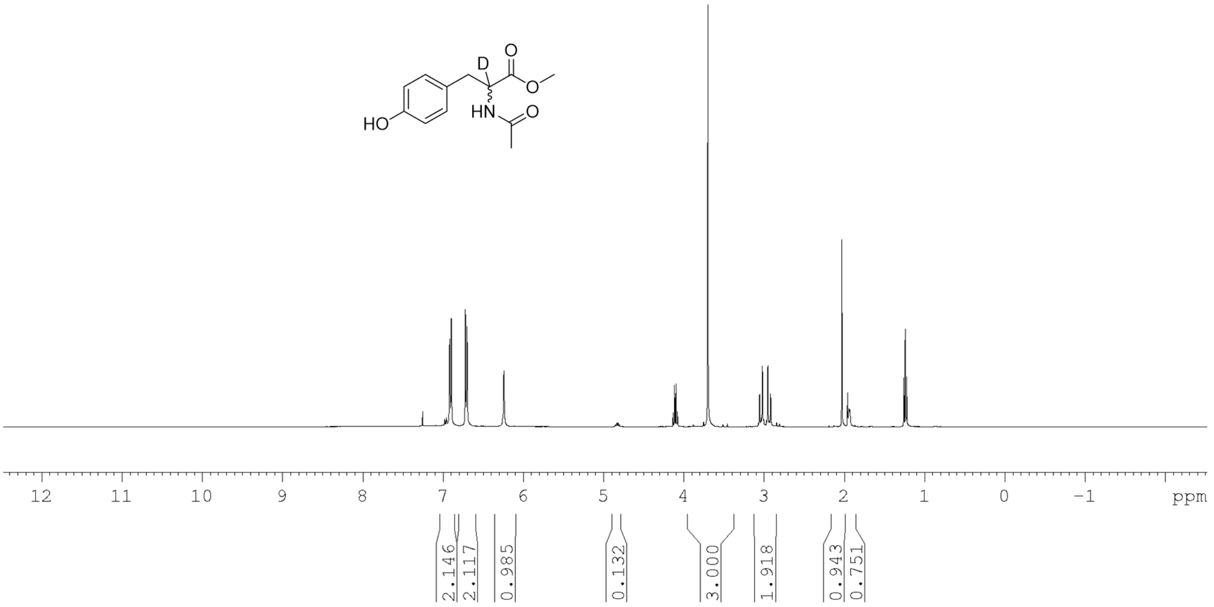

Figure S17. ^{1}H NMR spectrum of **S26** in $CDCl_3$.

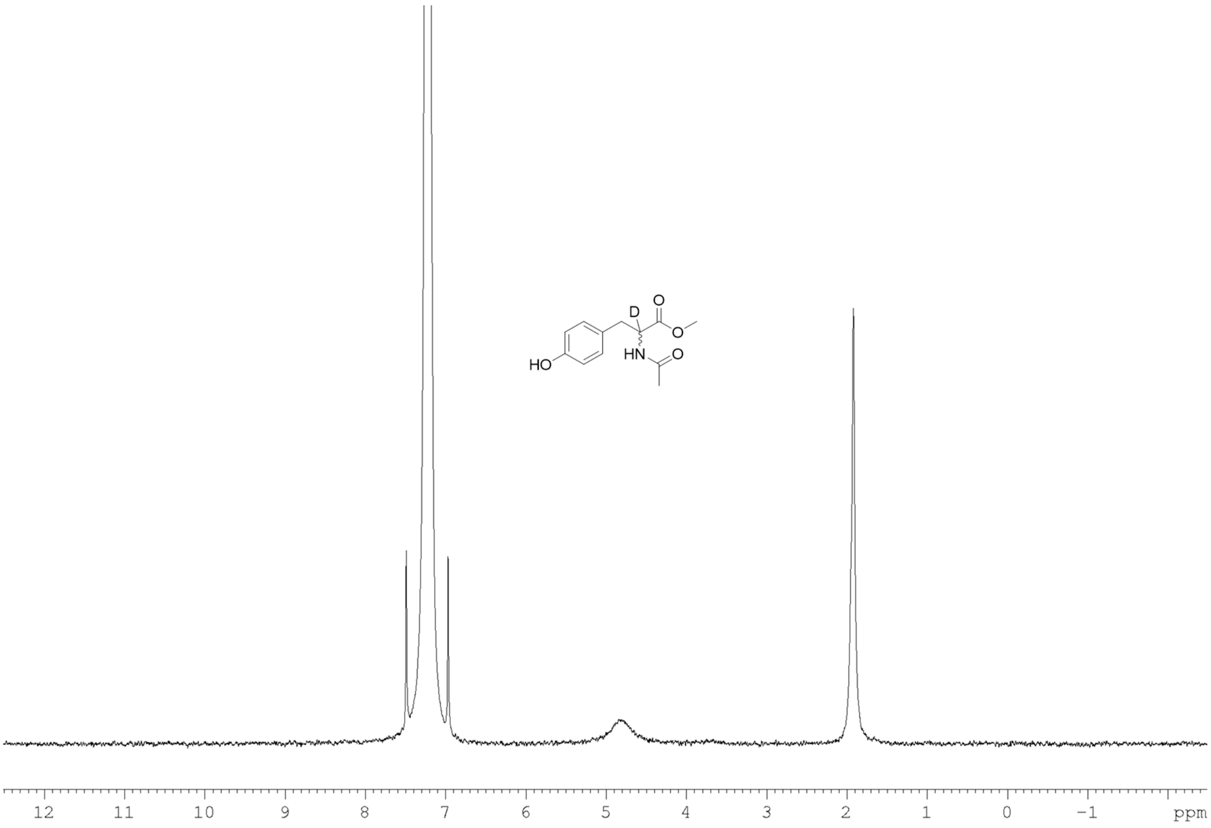

Figure S18. ^{2}H NMR spectrum of **S26** in $CDCl_3$.

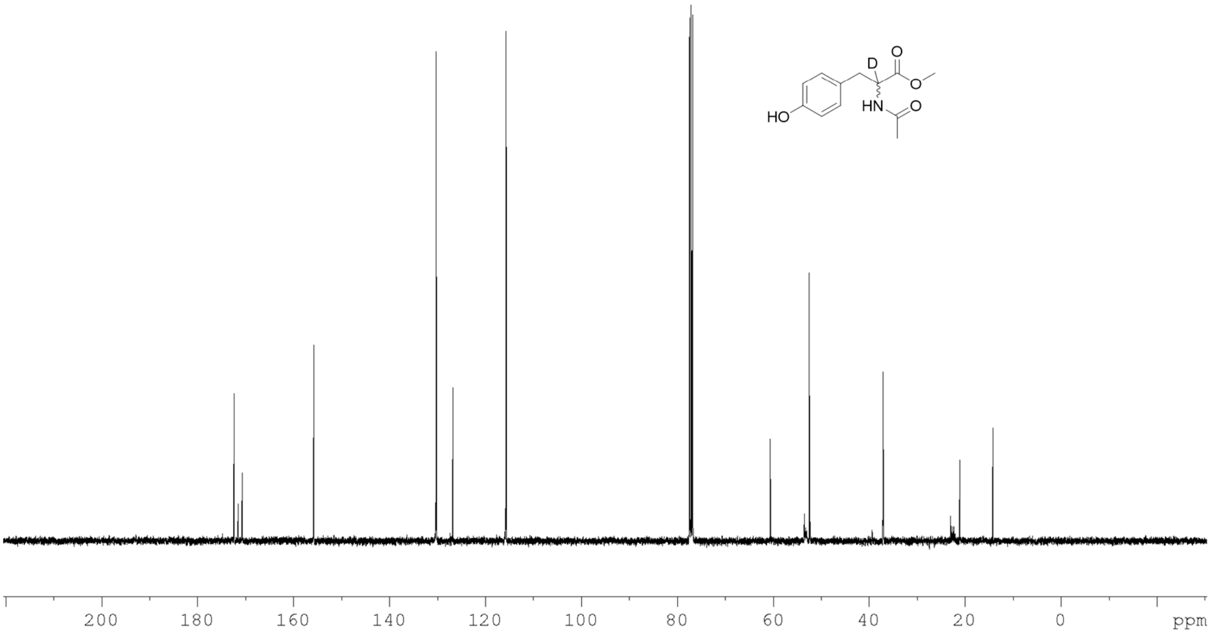

Figure S19. ^{13}C NMR spectrum **S26** in $CDCl_3$.

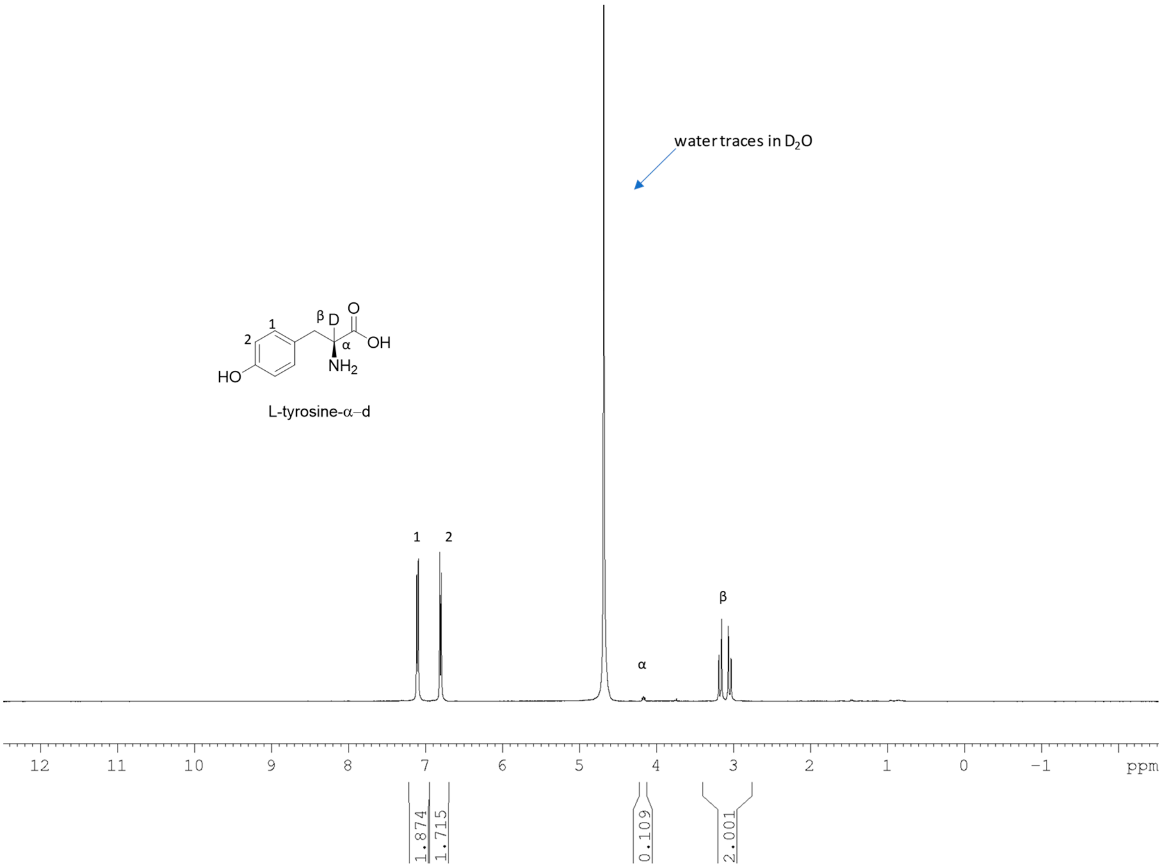

Figure S20. ^{1}H NMR spectrum of L-tyrosine-d_1 **S28** in D_2O.

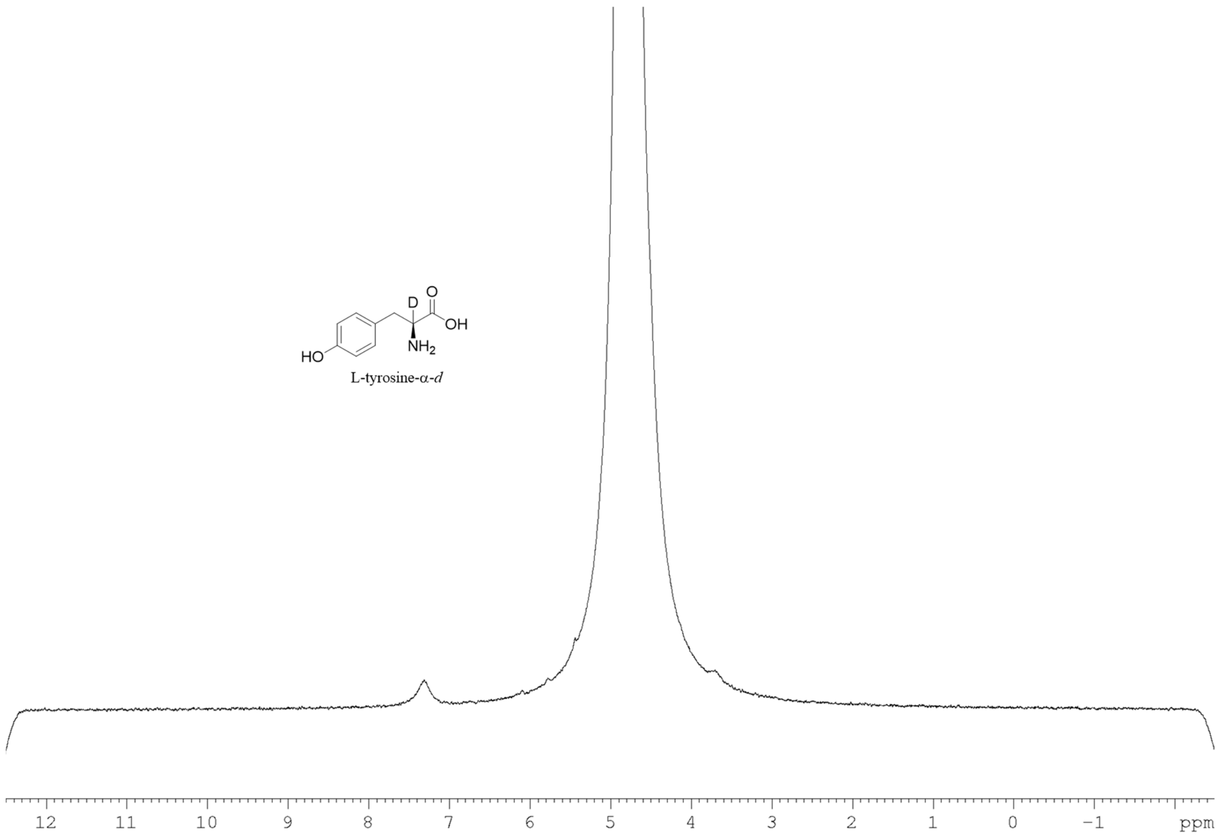

Figure S21. ^2H NMR spectrum of L-tyrosine-d_1 **S28** in D_2O.

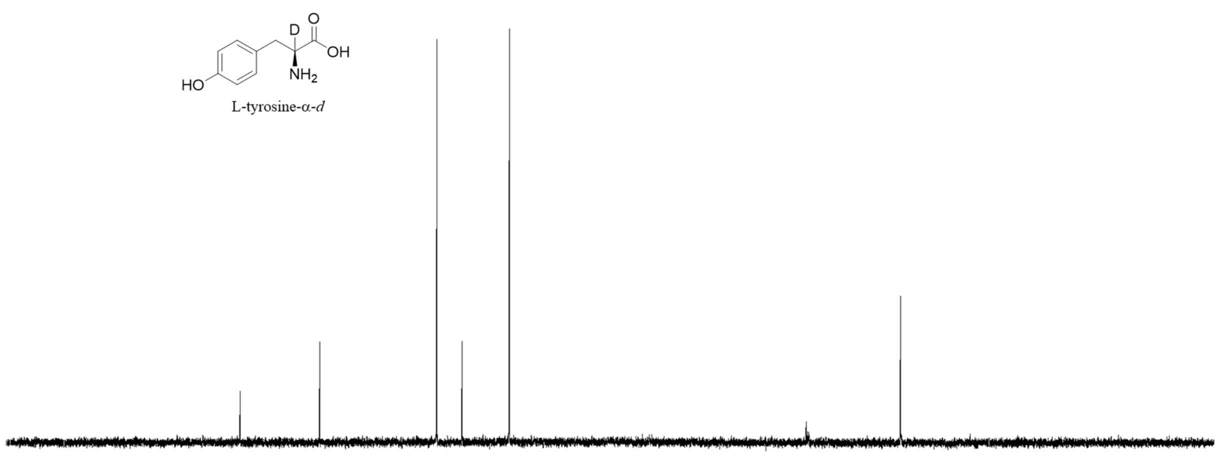

Figure S22. ^{13}C NMR spectrum of L-tyrosine-d_1 **S28** in D_2O.

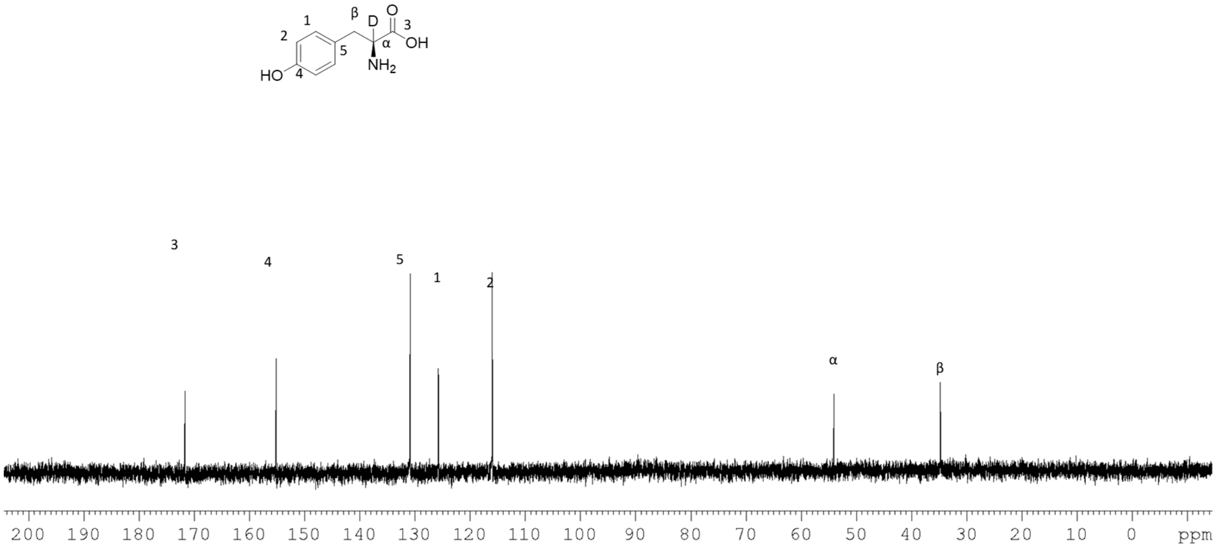

Figure S23. ^{13}C {^{1}H and ^{2}H} spectrum of L-tyrosine-d$_1$ **S28** in D_2O.

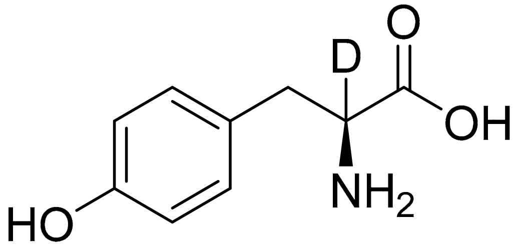

L-tyrosine-α-*d*

Figure S24. DL-Tyrosine-d1 dissolved into D and L tyrosine-d1 using alcalase enzyme under basic conditions. Specific rotation of L-tyrosine-d1 **S28** $[\alpha]_D^{25.8}$ -11.902° (c, 4 in 1M HCl) ref. (L-Tyroisne $[\alpha]_D^{20}$ -11.5° (c, 4 in 1M HCl) in Sigma Aldrich unlabelled version).

Information

Date: 2023-10-12
Notes:

Compound Information

Name / ID:
Formula: C9H10DNO3
m/z: 182.0802
Adduct: [M+H]+
Adduct m/z: 183.0874

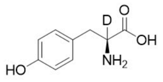

Results

Deuteration: 87.83 %
Deuteration Ratio Spectra
D0: 12.17 %
D1: 87.83 %

Spectra

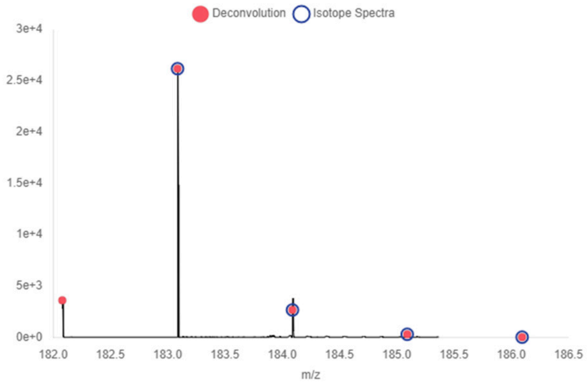

Report generated using the DGet (0.26) web application (0.26.1).

For more information see https://github.com/djdt/dget.

Figure S25. ESMS m/z 183 [M+1] mass spectrum of L-tyrosine-d1; overall deuteration of 87.83%D, https://dget.app/.

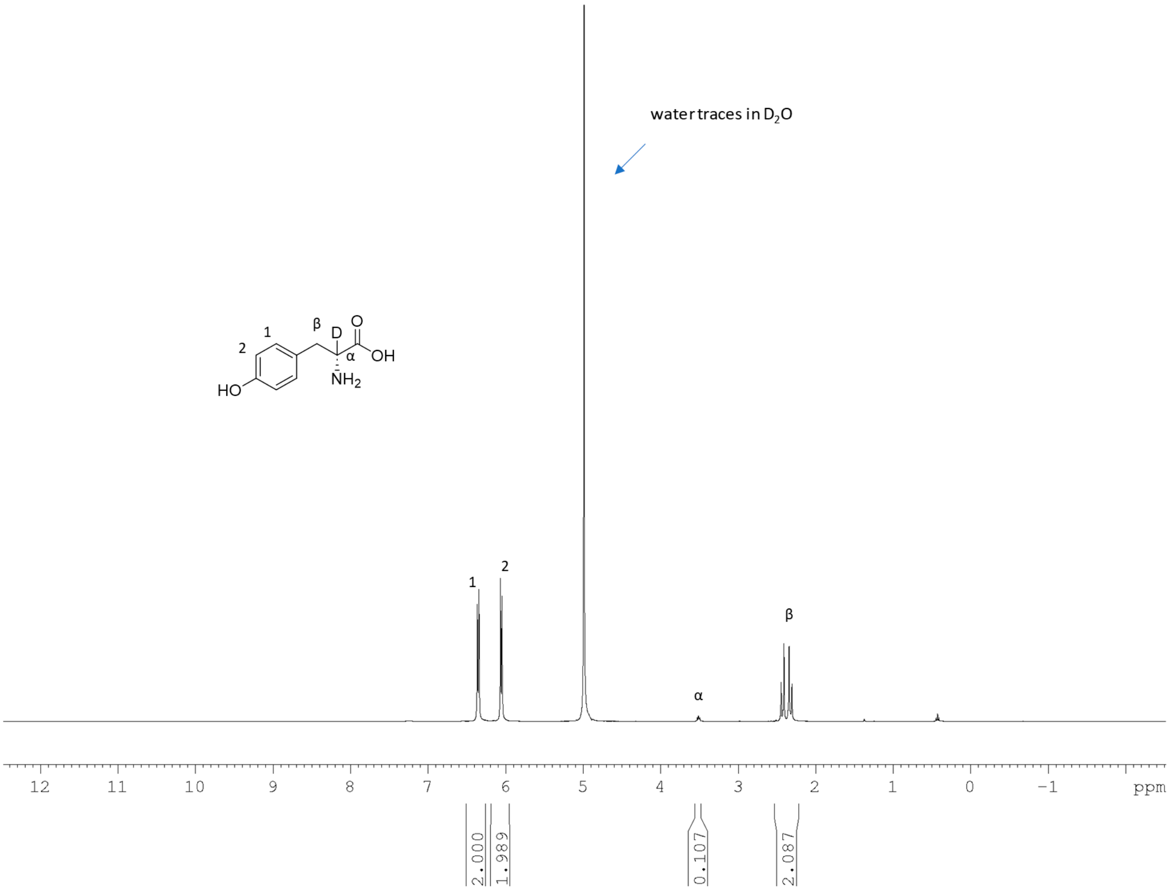

Figure S26. ¹H NMR spectrum of D-tyrosine-d1 **S30** in D_2O.

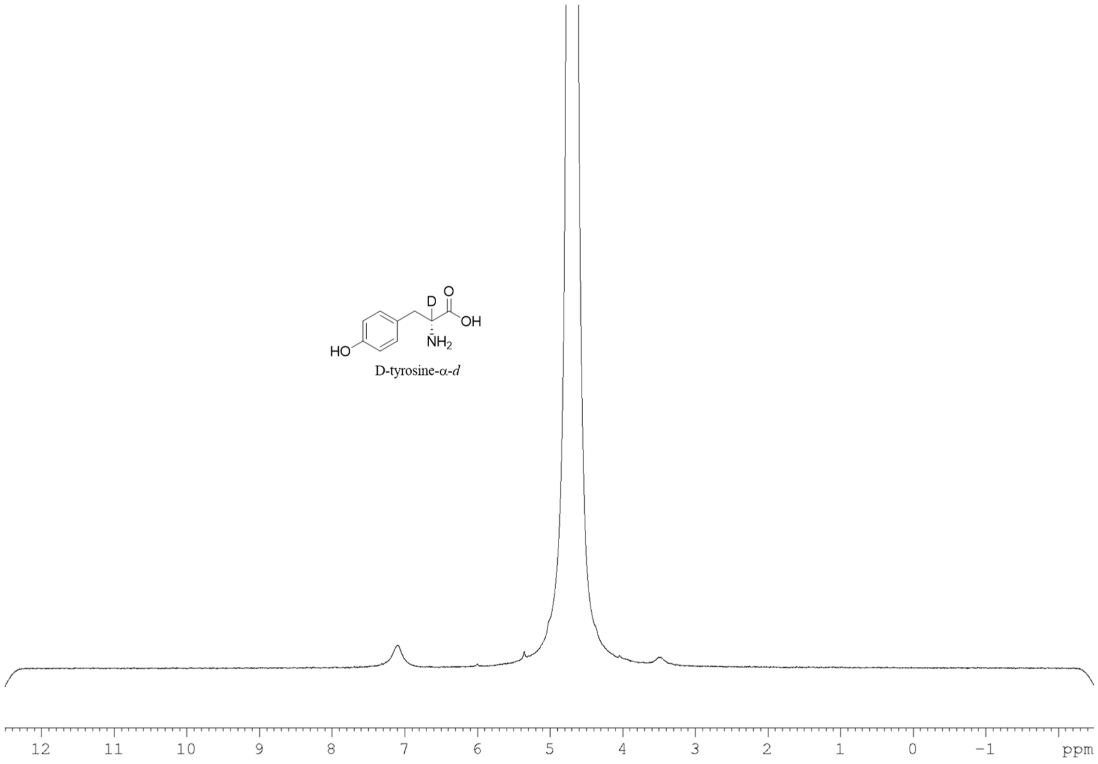

Figure S27. ^2H NMR spectrum of D-tyrosine-d_1 **S30** in D_2O.

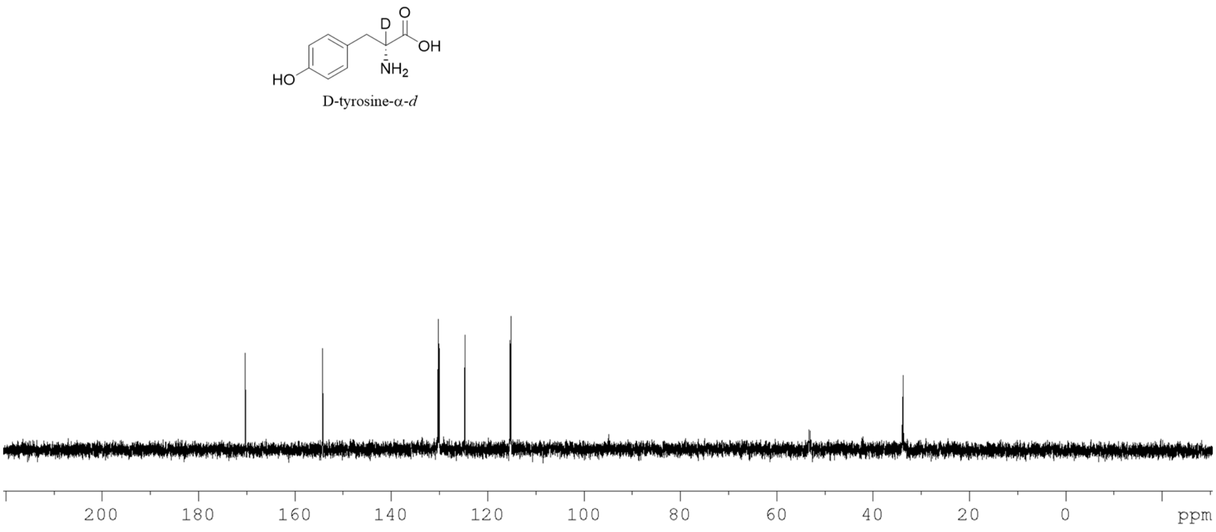

Figure S28. ^{13}C NMR spectrum of D-tyrosine-d_1 **S30** in D_2O.

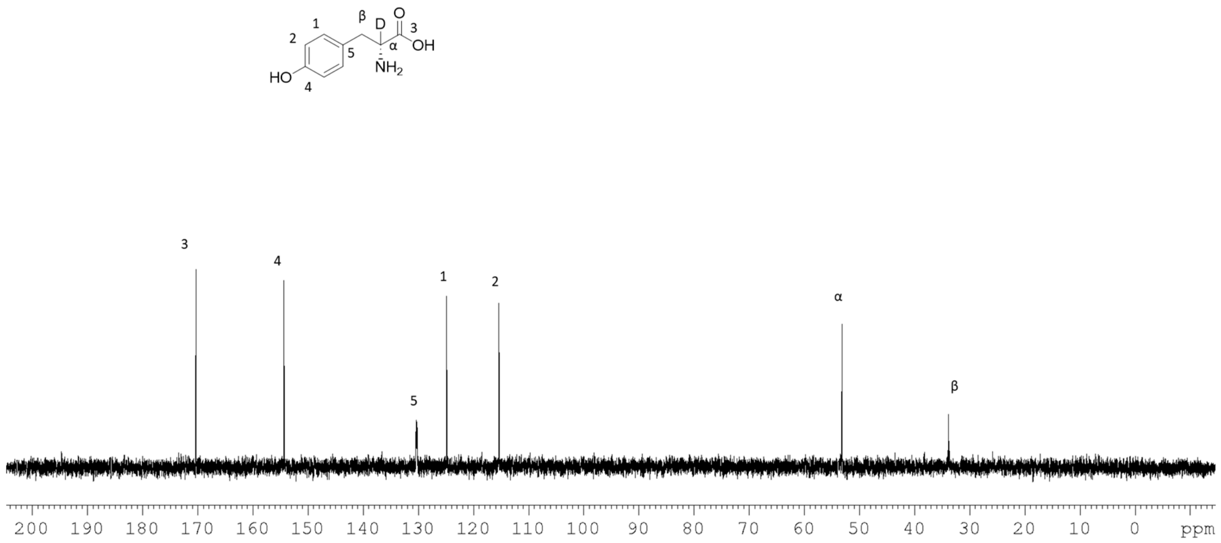

Figure S29. ^{13}C {1H and 2H} spectrum of D-tyrosine-d$_1$ **S30** in D_2O.

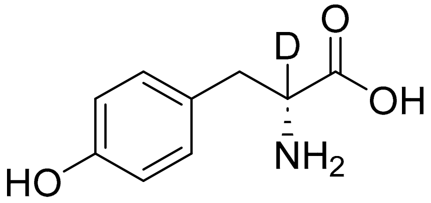

D-tyrosine-α-*d*

Figure S30. DL-tyrosine-d1 dissolved into D and L tyrosine-d$_1$ using alcalase enzyme under basic conditions. Specific rotation of D-Tyrosine-d1 **S30** $[\alpha]_D^{25.8}$ +11.922° (c, 5 in 1M HCl) (D-Tyroisne $[\alpha]_D^{20}$ +11. 5° (c, 5 in 1M HCl) in Sigma Aldrich unlabelled version).

Information

Date: 2023-10-10
Notes:

Compound Information

Name / ID:
Formula: C9H10DNO3
m/z: 182.0802
Adduct: [M+H]+
Adduct m/z: 183.0874

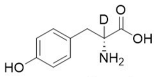

D-tyrosine-α-*d*

Results

Deuteration: **88.64 %**
Deuteration Ratio Spectra
D0: 11.36 %
D1: 88.64 %

Spectra

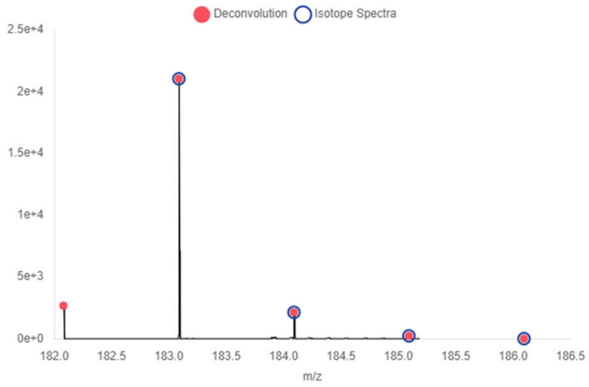

Report generated using the DGet (0.26) web application (0.26.1).
For more information see https://github.com/djdt/dget.

Figure S31. ESMS m/z 183 [M^{+1}] mass spectrum of D-Tyrosine-d_1**S30**; overall deuteration of 88.64%D, https://dget.app/.

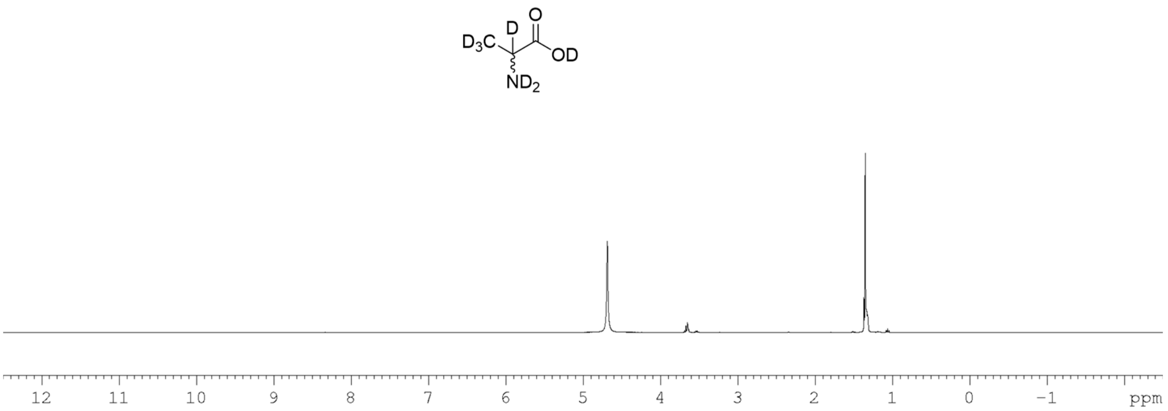

Figure S32. ^{1}H NMR spectrum of DL-alanine-d_7 **S31** in D_2O.

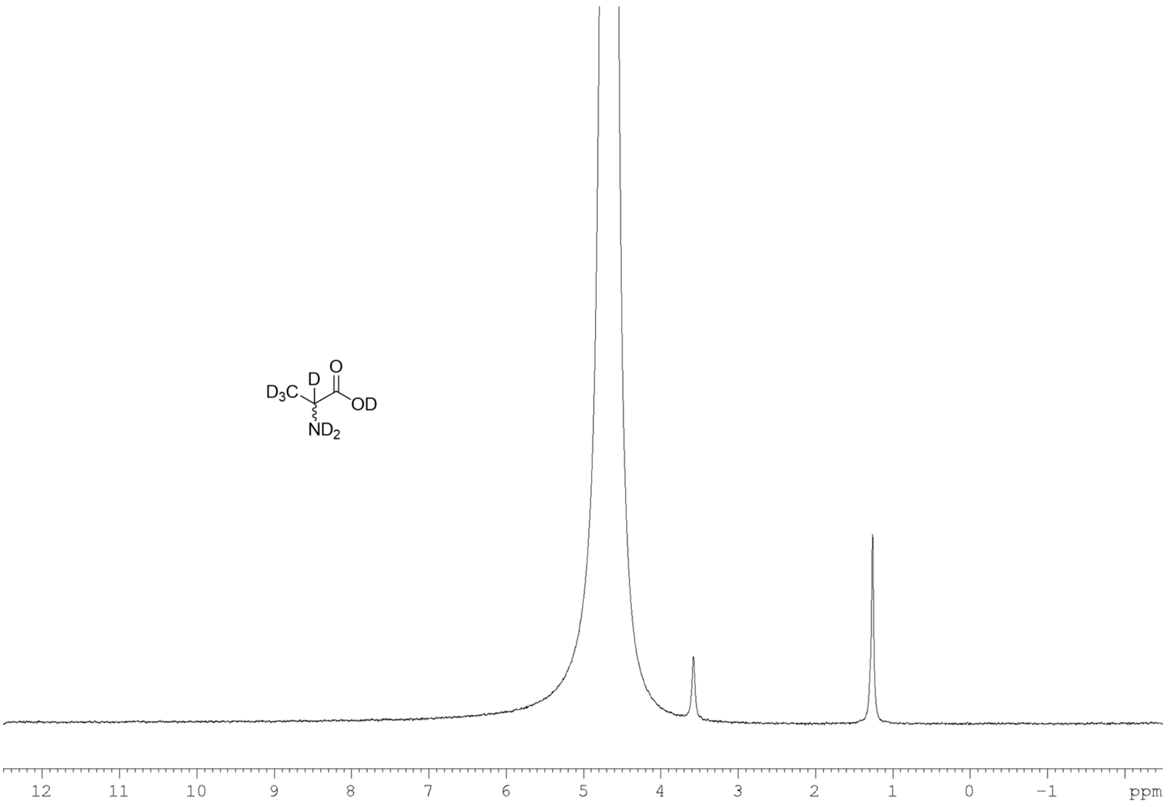

Figure S33. ^{2}H NMR spectrum of DL-alanine-d_7 **S31** in D_2O.

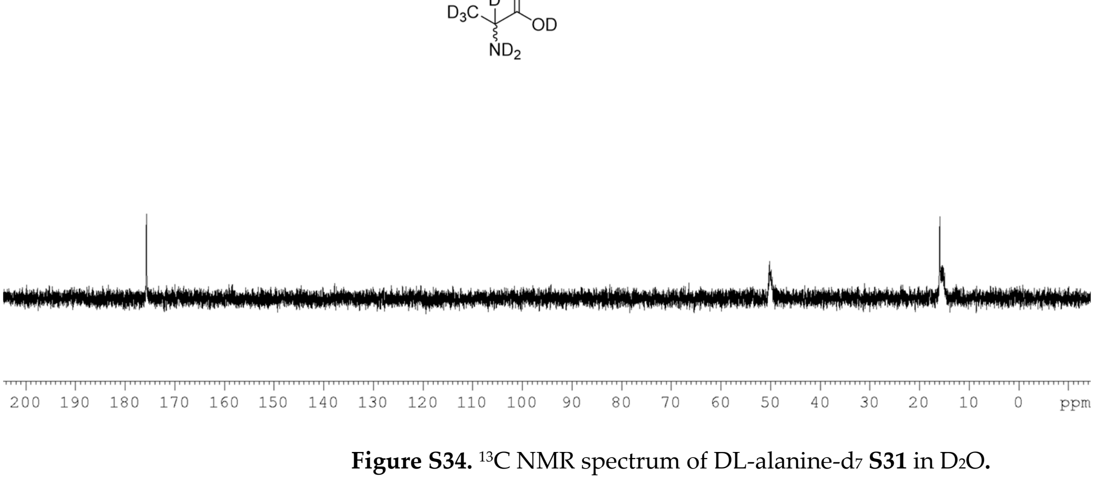

Figure S34. ^{13}C NMR spectrum of DL-alanine-d_7 **S31** in D_2O.

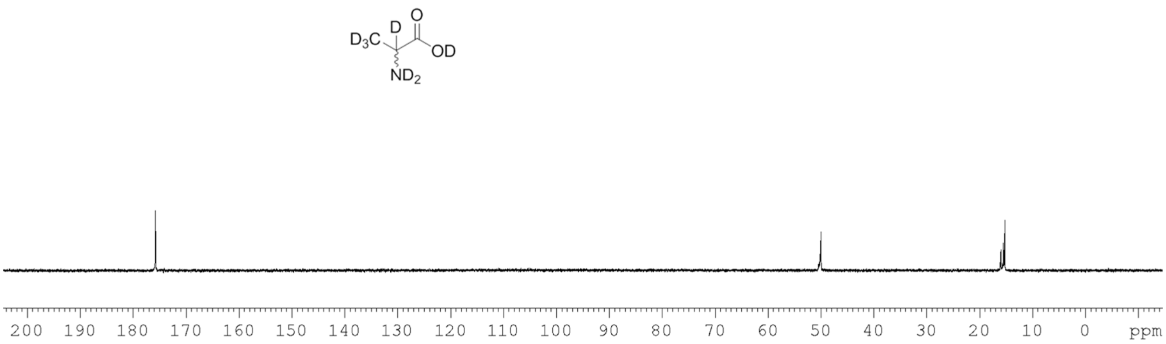

Figure S35. ^{13}C {^{1}H} {^{2}H} spectrum of DL-alanine-d_7 **S31** in D_2O.

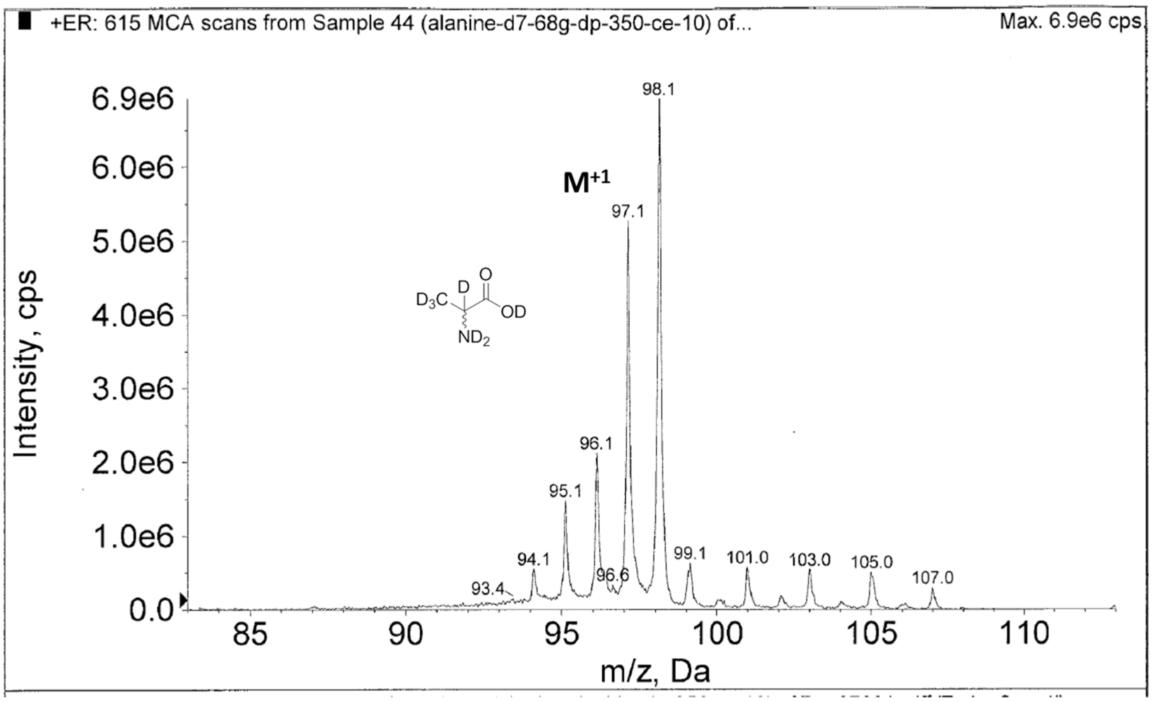

Figure S36. ESI-MS m/z 98 $[M+1]^{+}$ mass spectrum of DL-alanine-d_7 **S31**.

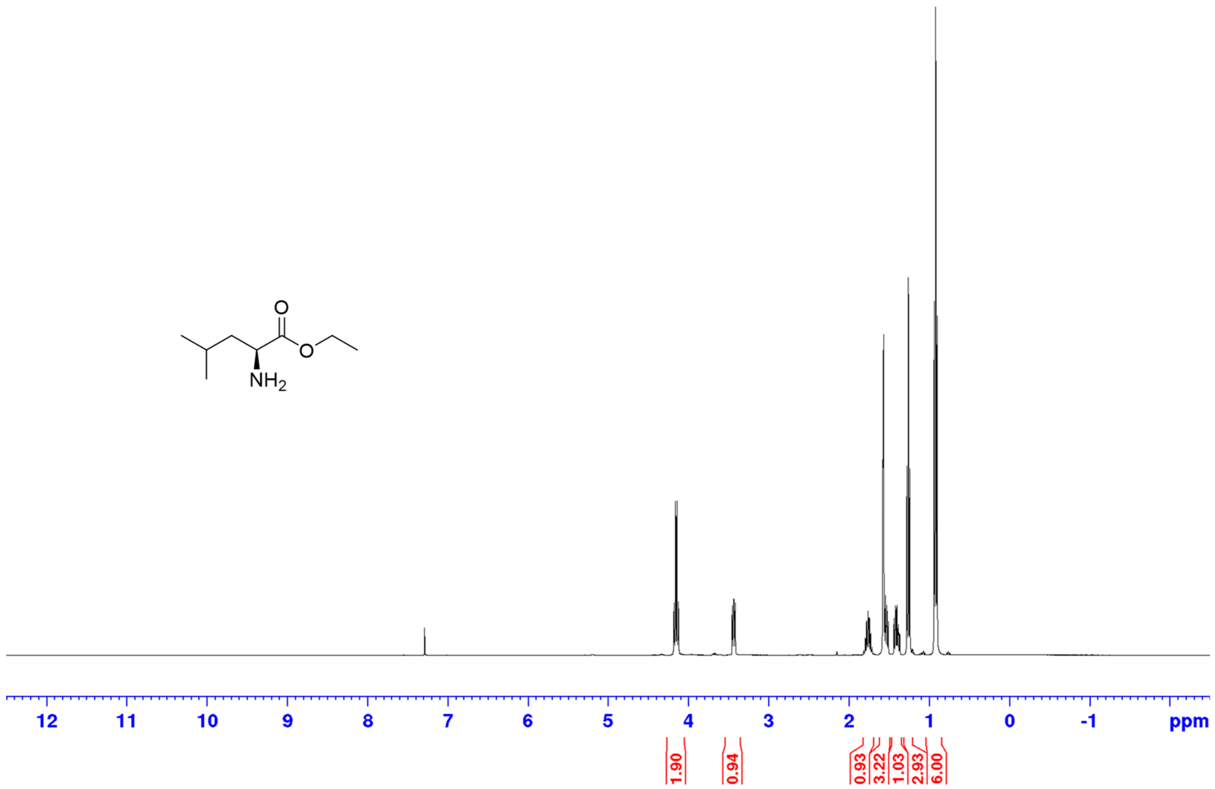

Figure S37. ¹H NMR spectrum of L-leucinate **S34** in CDCl₃.

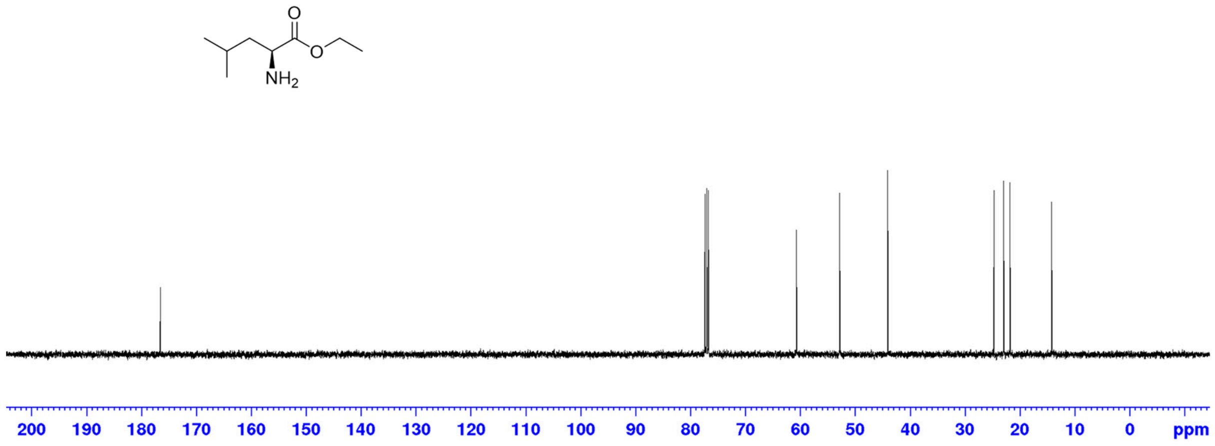

Figure S38. ¹³C NMR spectrum of L-leucinate **S34** in CDCl₃.

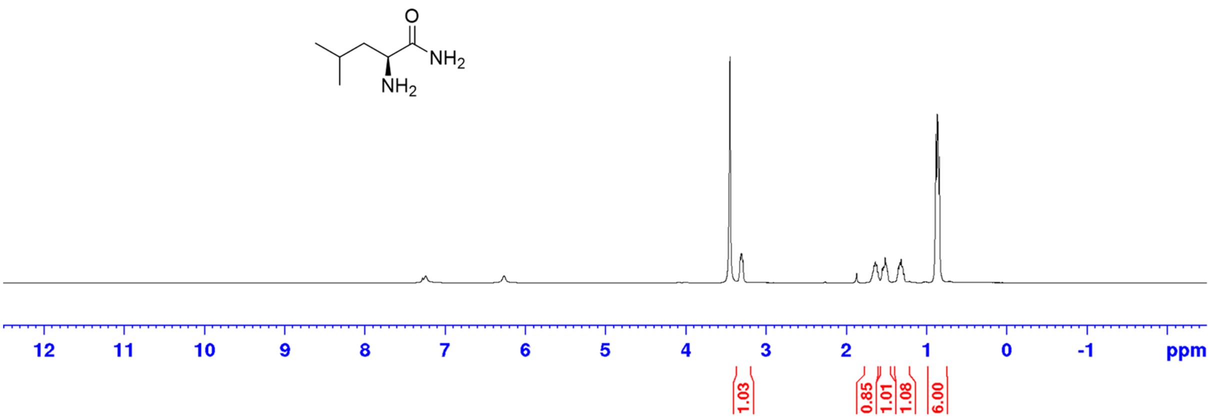

Figure S39. ^{1}H NMR spectrum of L-leucinamide **S35** in $CDCl_3$.

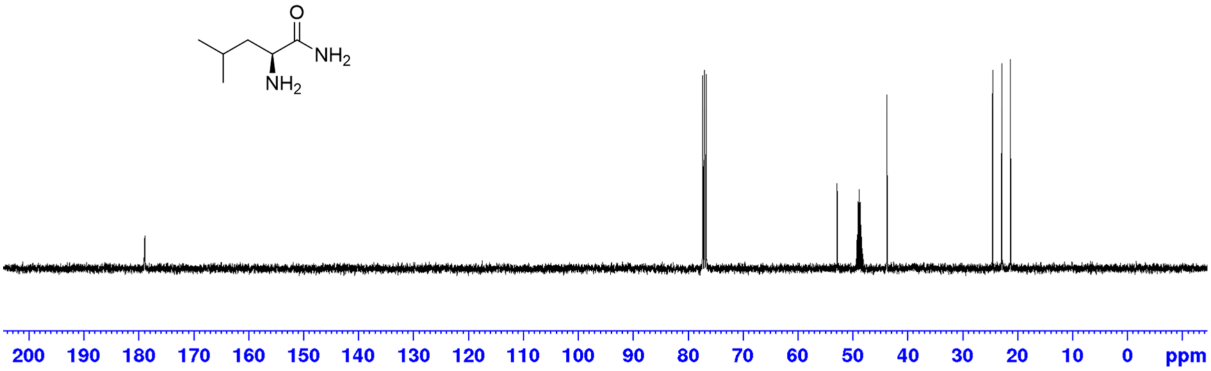

Figure S40. ^{13}C NMR spectrum of L-leucinamide **S35** in $CDCl_3$.

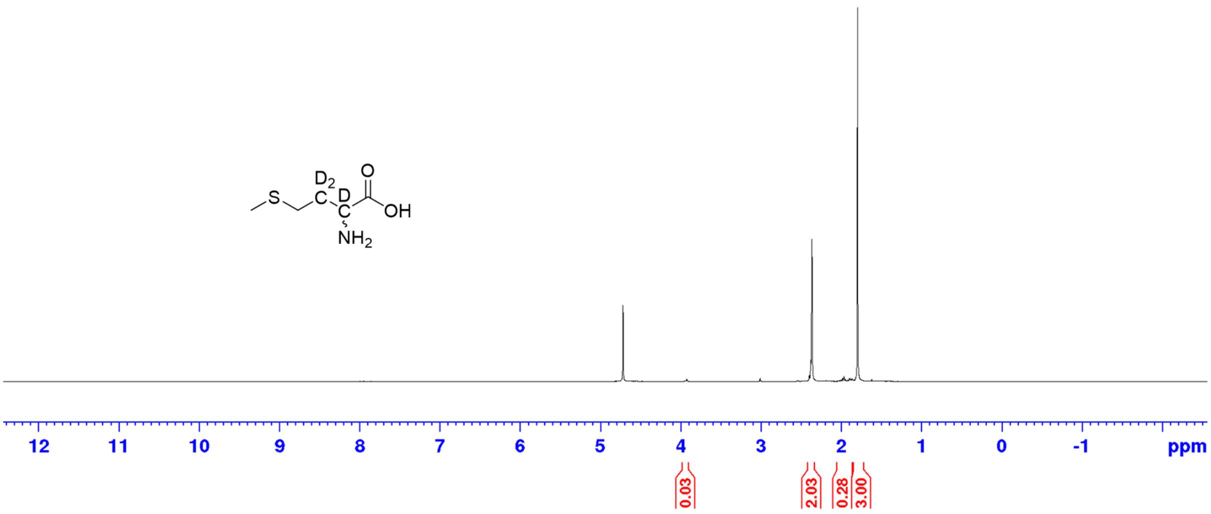

Figure S41. ^{1}H NMR spectrum of DL-methionine-d3 **S38** in D_2O.

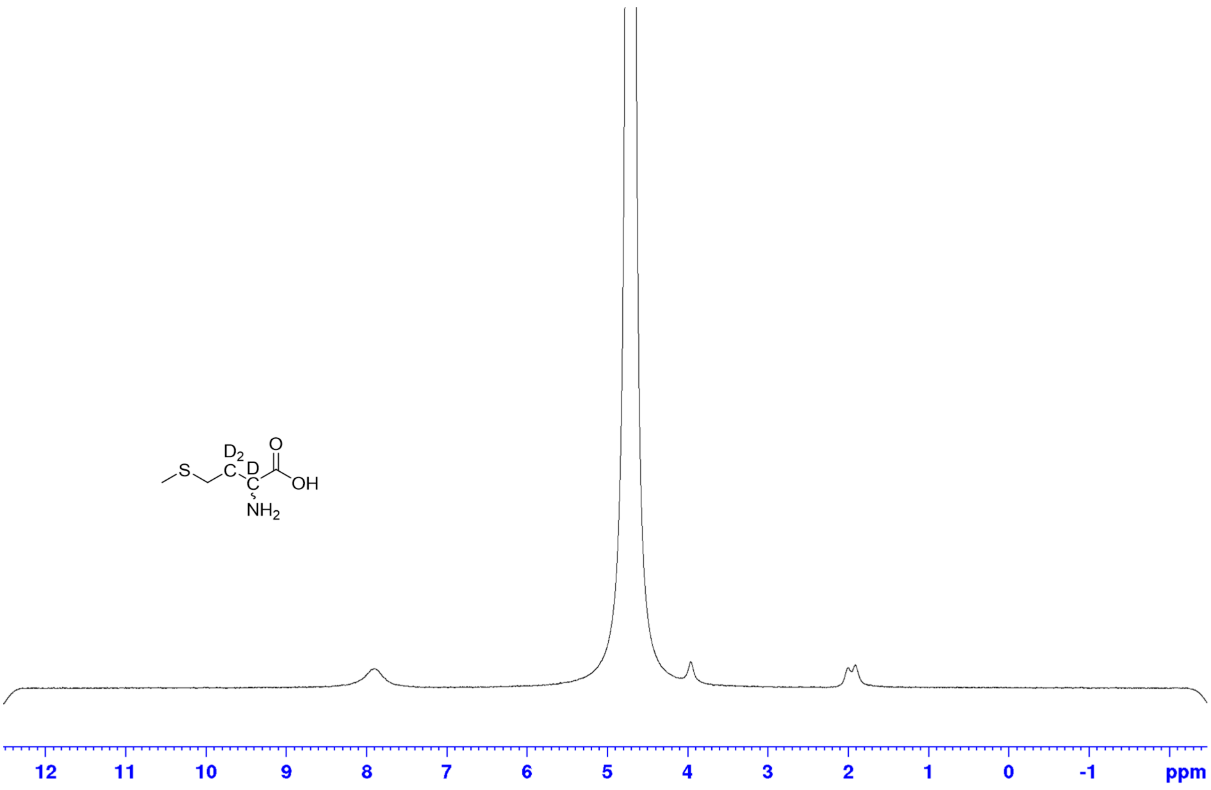

Scheme 42. O.

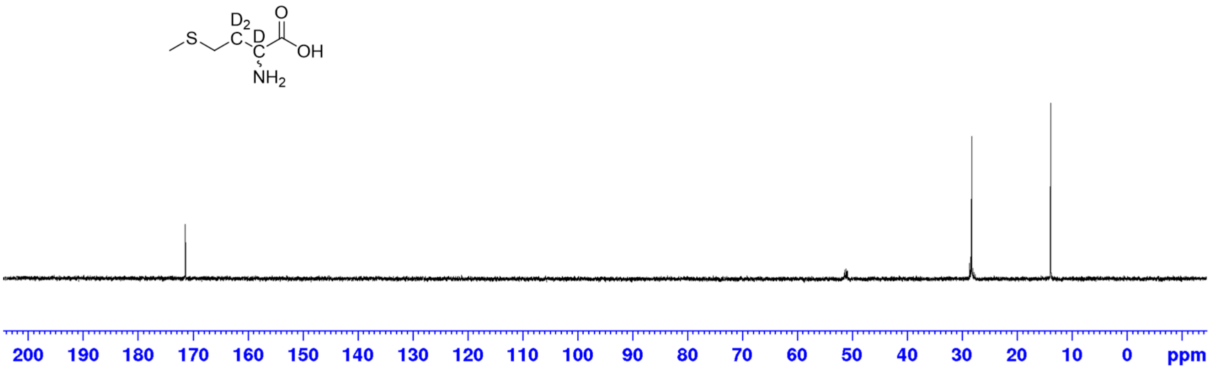

Figure S43. ^{13}C NMR spectrum of DL-methionine-d3 **S38** in D_2O.

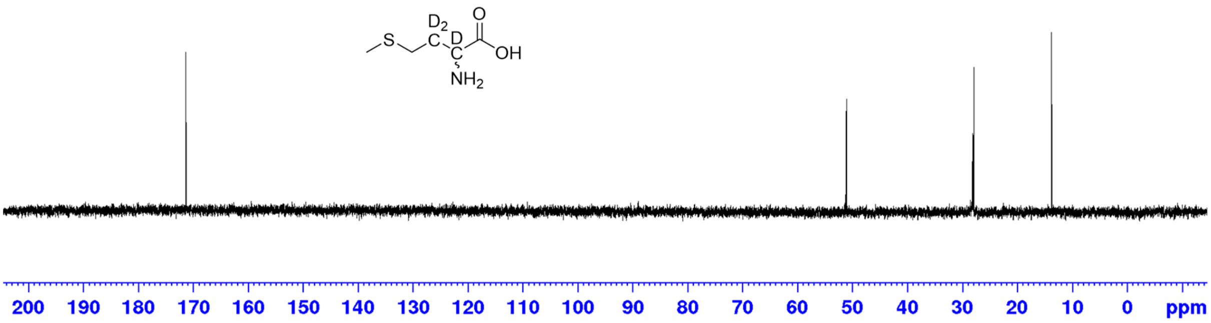

Figure S44. ^{13}C {^1H and ^2H} spectrum of DL-methionine-d3 **S38** in D_2O.

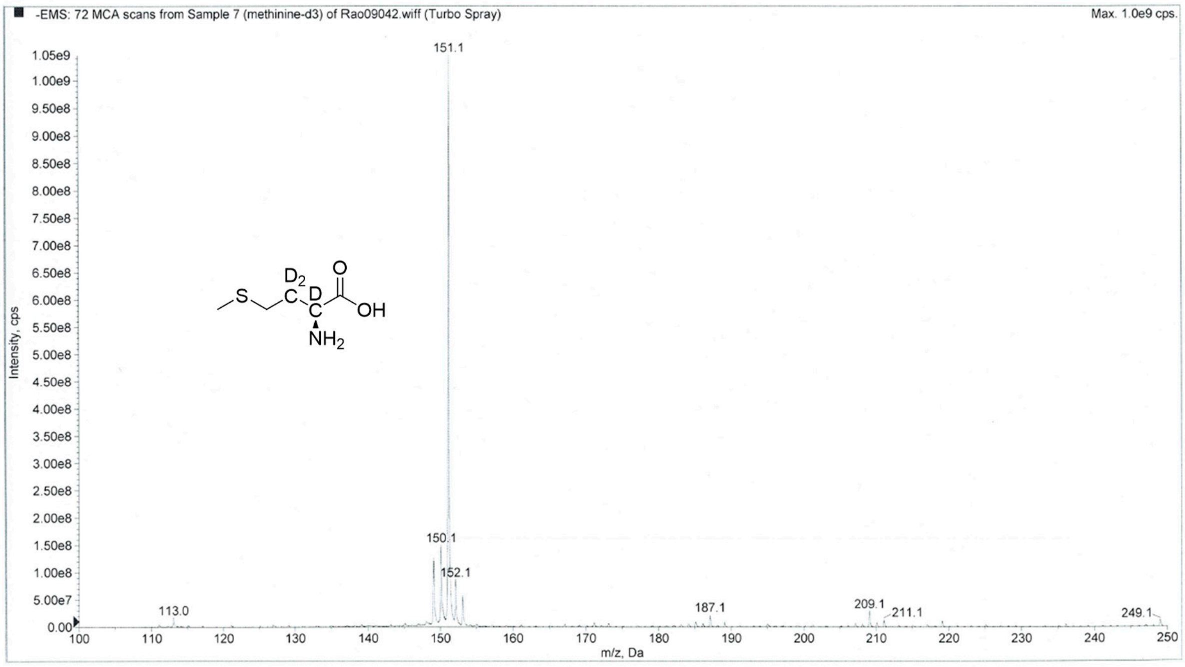

Figure S45. ESMS m/z mass spectrum of **S38** 151 [M-1]⁻.

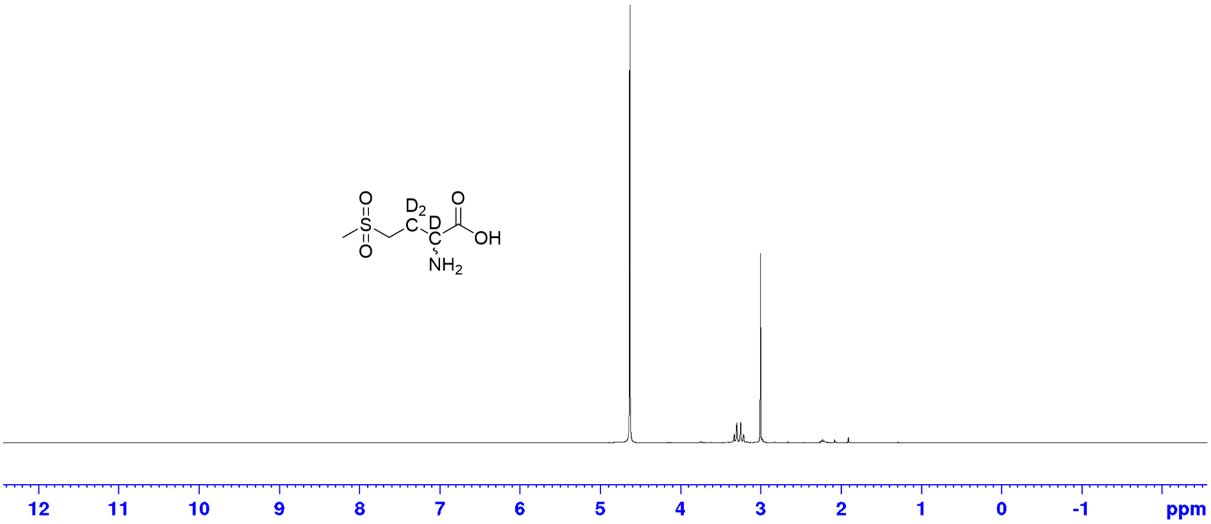

Figure S46. ^{1}H NMR spectrum of DL-methioninedioxide-d$_3$ **S43** in D_2O.

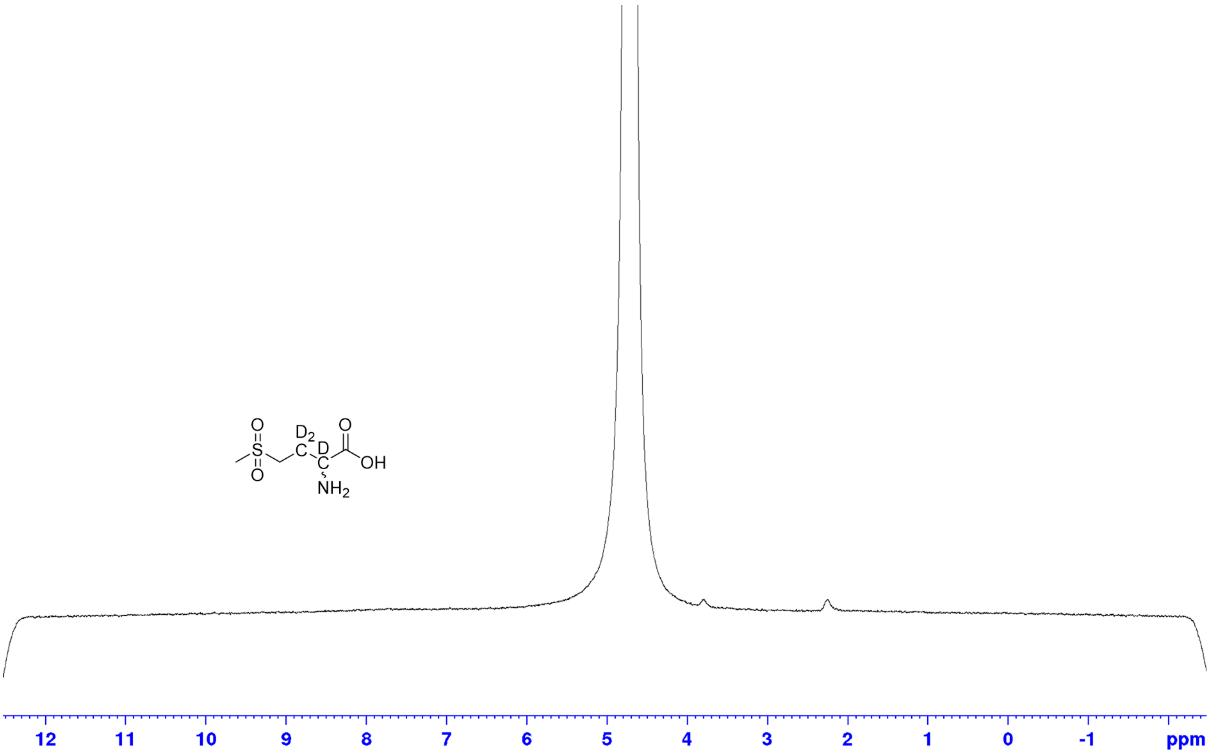

Figure S47. ^{2}H NMR spectrum of DL-methioninedioxide-d$_3$ **S43** in D_2O.

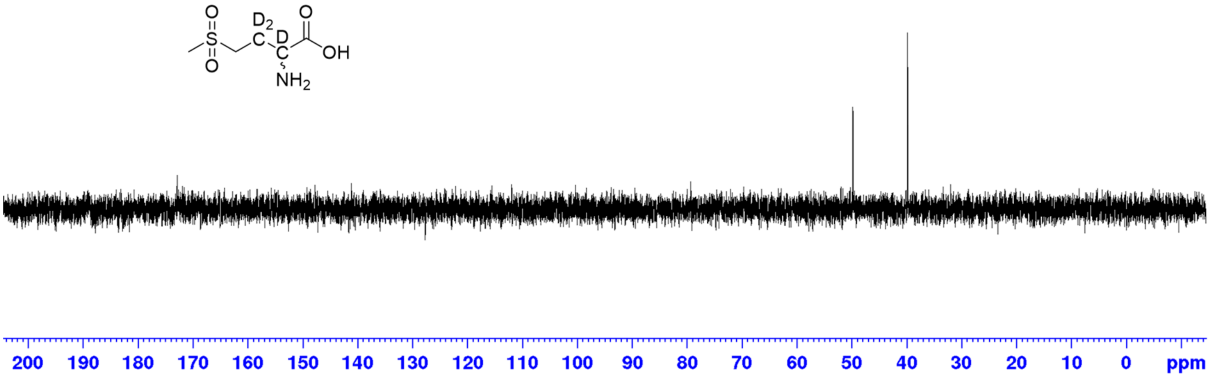

Figure S48. ^{13}C NMR spectrum of DL-methioninedioxide-d$_3$ **S43** in D_2O.

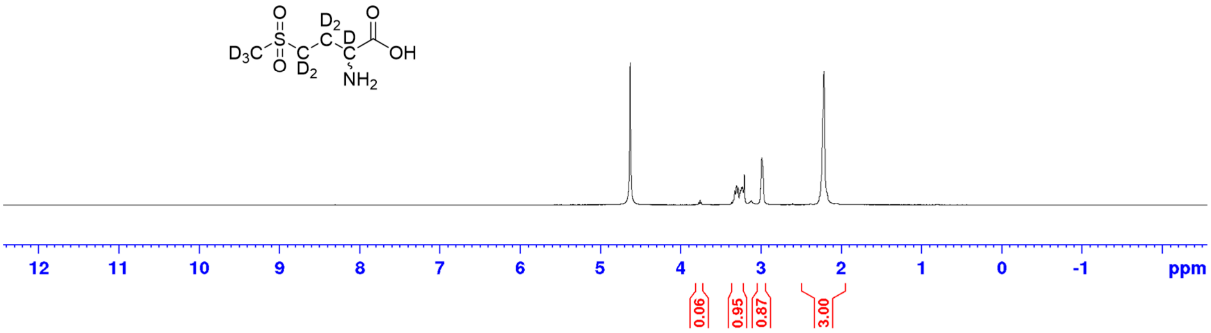

Figure S49. ^{1}H NMR spectrum of DL-methioninedioxide-d3 **S43** in D_2O.

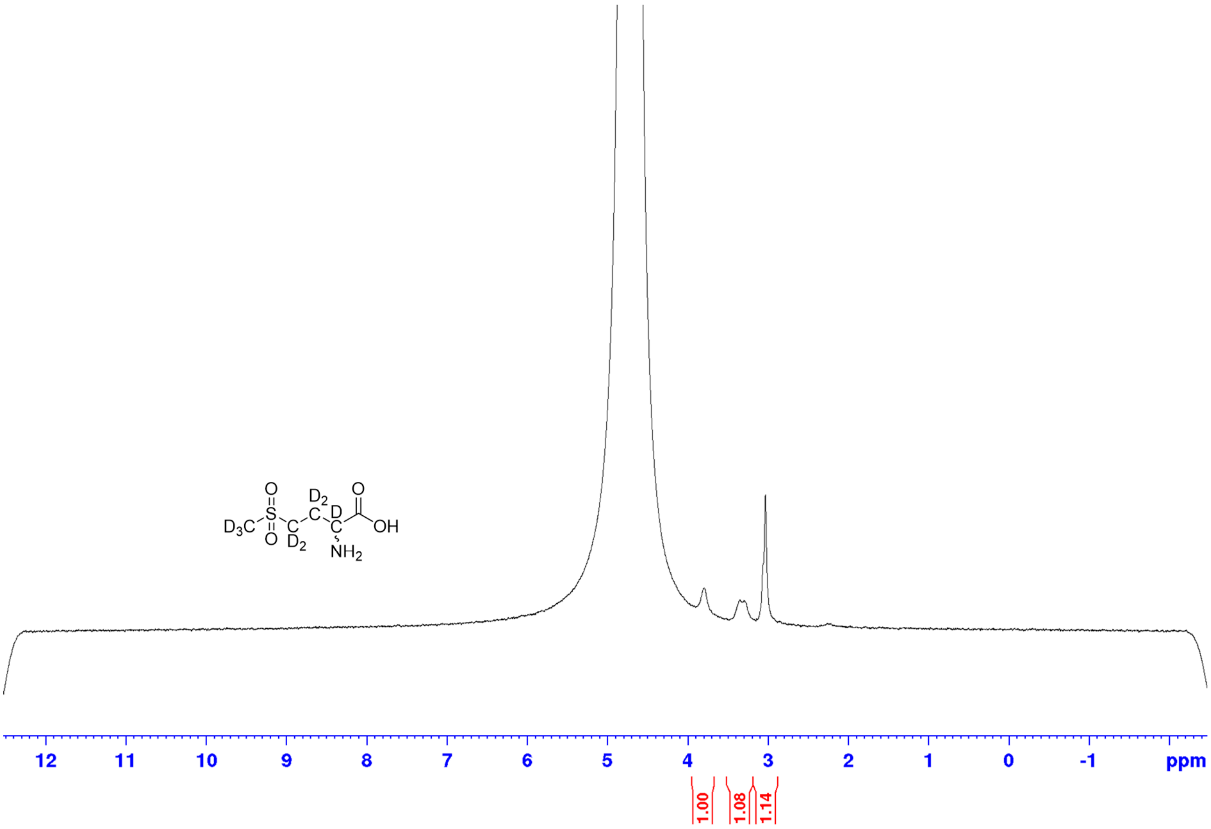

Figure S50. ^{2}H NMR DL-methioninedioxide-d_3 **S43** in D_2O.

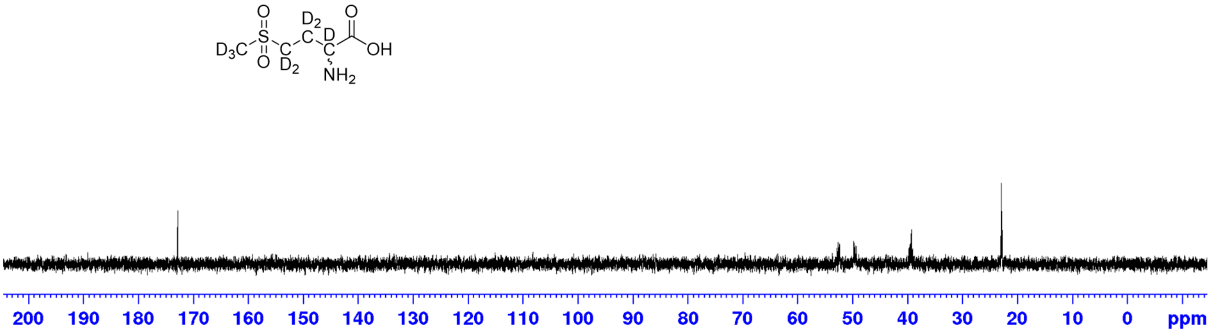

Figure S51. ^{13}C NMR spectrum of DL-methioninedioxide-d$_3$ **S43** in D_2O.

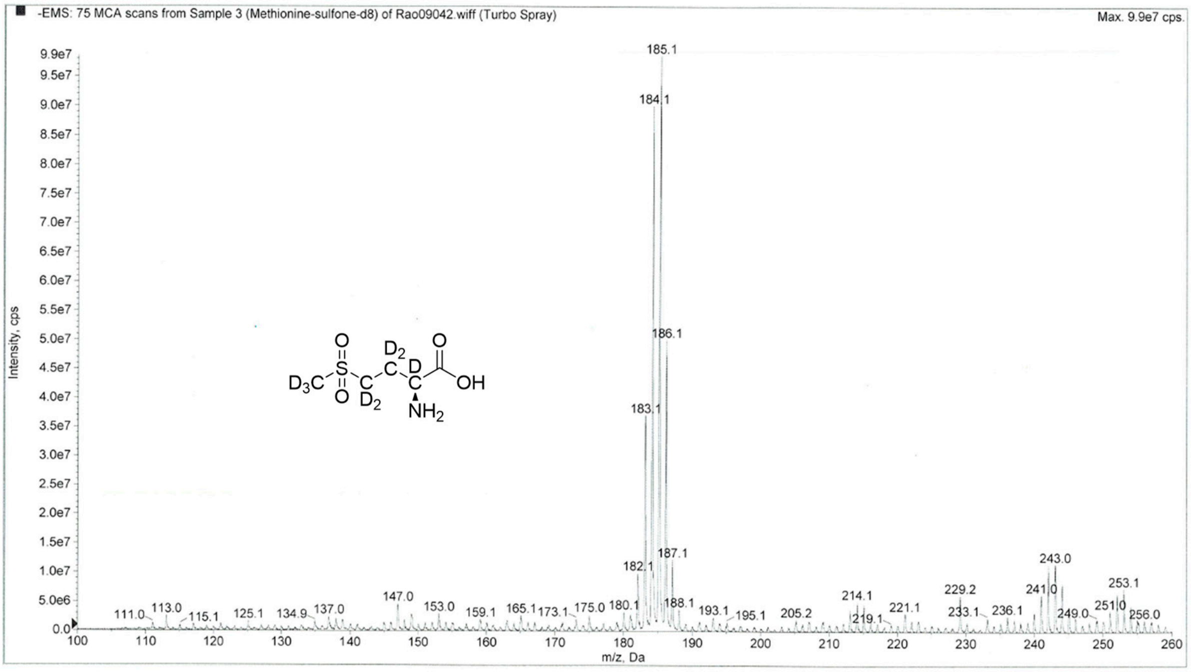

Figure S52. ESMS m/z 188 [M-1]$^-$ mass spectrum of **S43**.

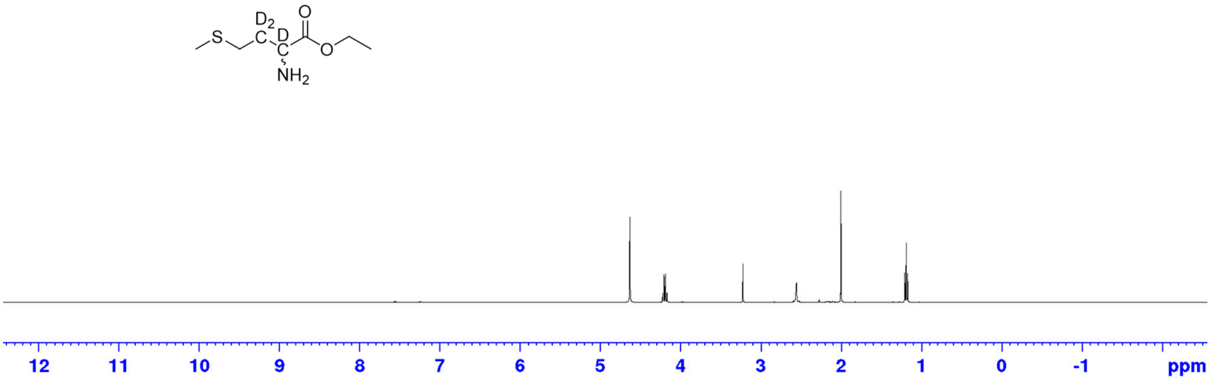

Figure S53. ^{1}H NMR spectrum of DL-methionine-d$_3$ **S39** in D_2O.

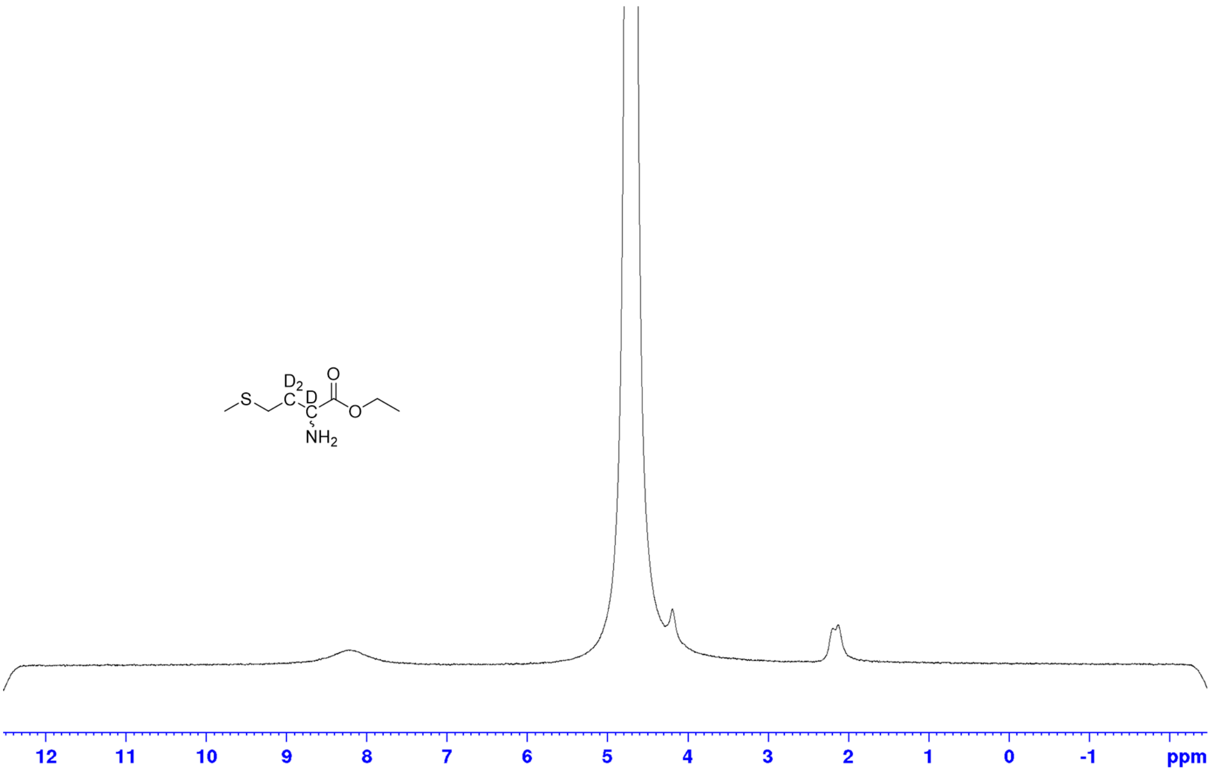

Figure S54. ^{2}H NMR spectrum of DL-methionine-d$_3$ **S39** in D_2O.

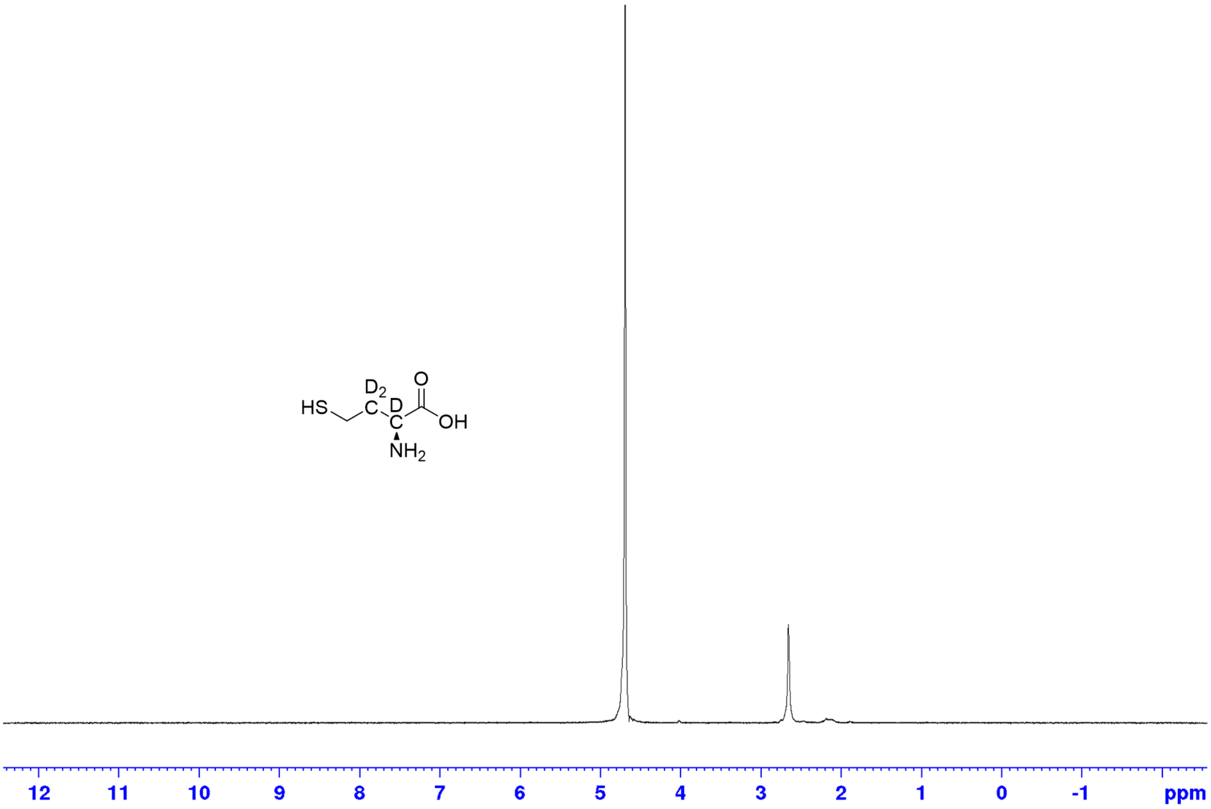

Figure S55. ^1H NMR spectrum of L-homcystein-d3 **S42** in D_2O.

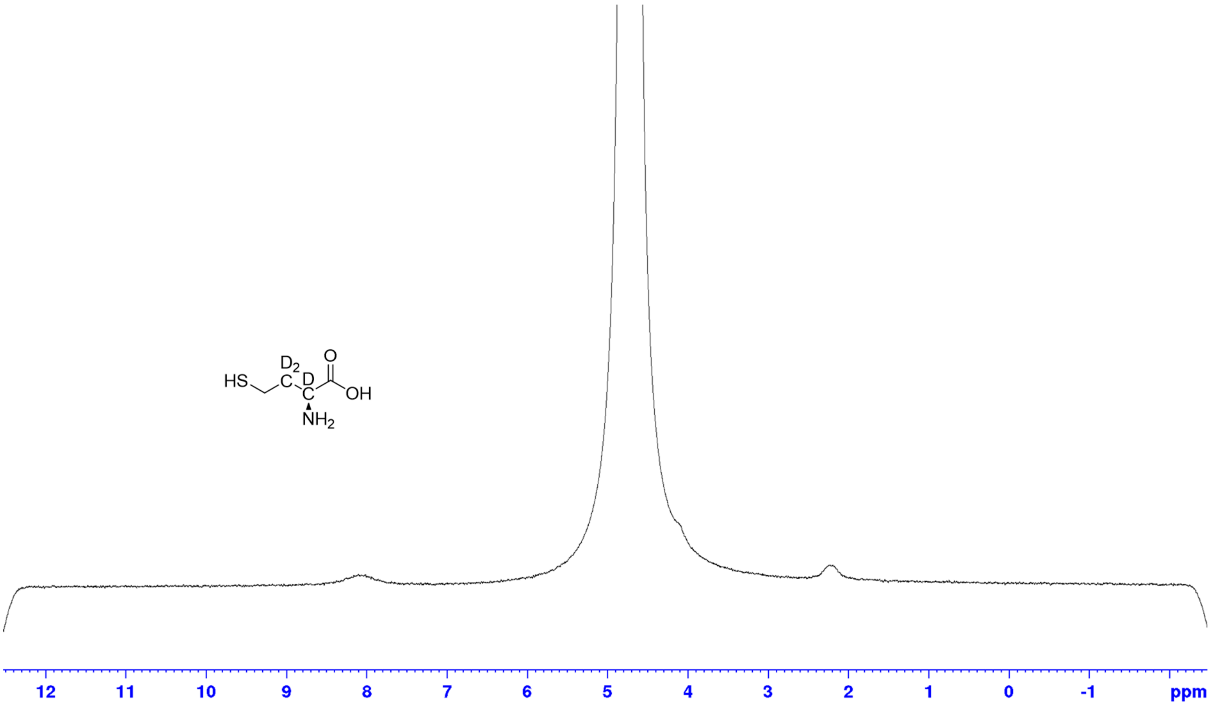

Figure S56. ^2H NMR spectrum of L-homcystein-d3 **S42** D_2O.

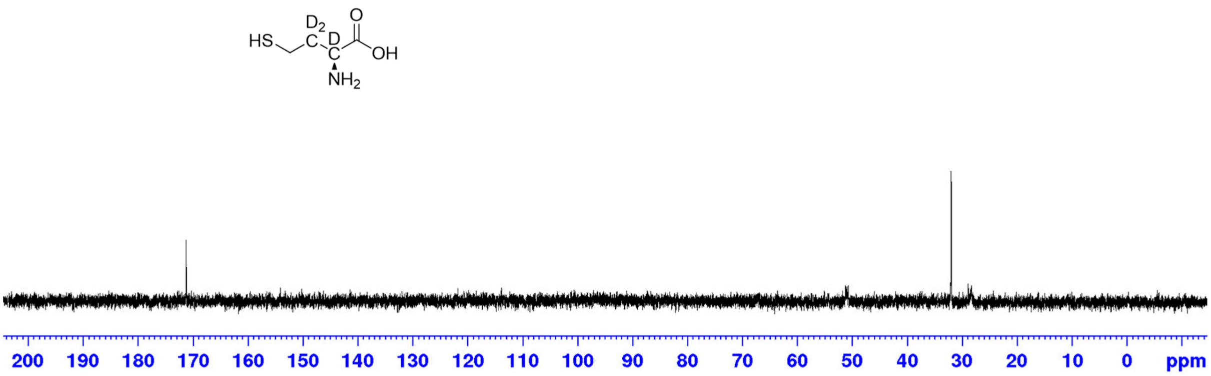

Figure S57. ^{13}C NMR spectrum of L-homcystein-d$_3$ **S42** D_2O.

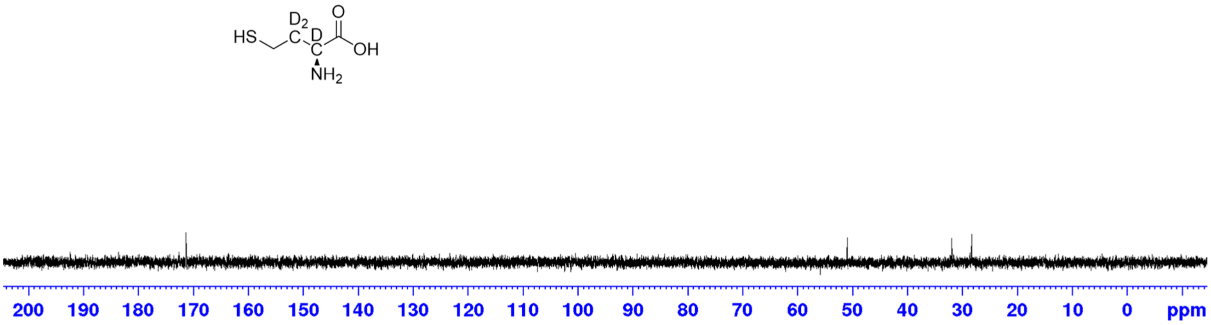

Figure S58. ^{13}C {^1H and ^2H} spectrum of L-homocystein-d$_3$ **S42** in $CDCl_3$.

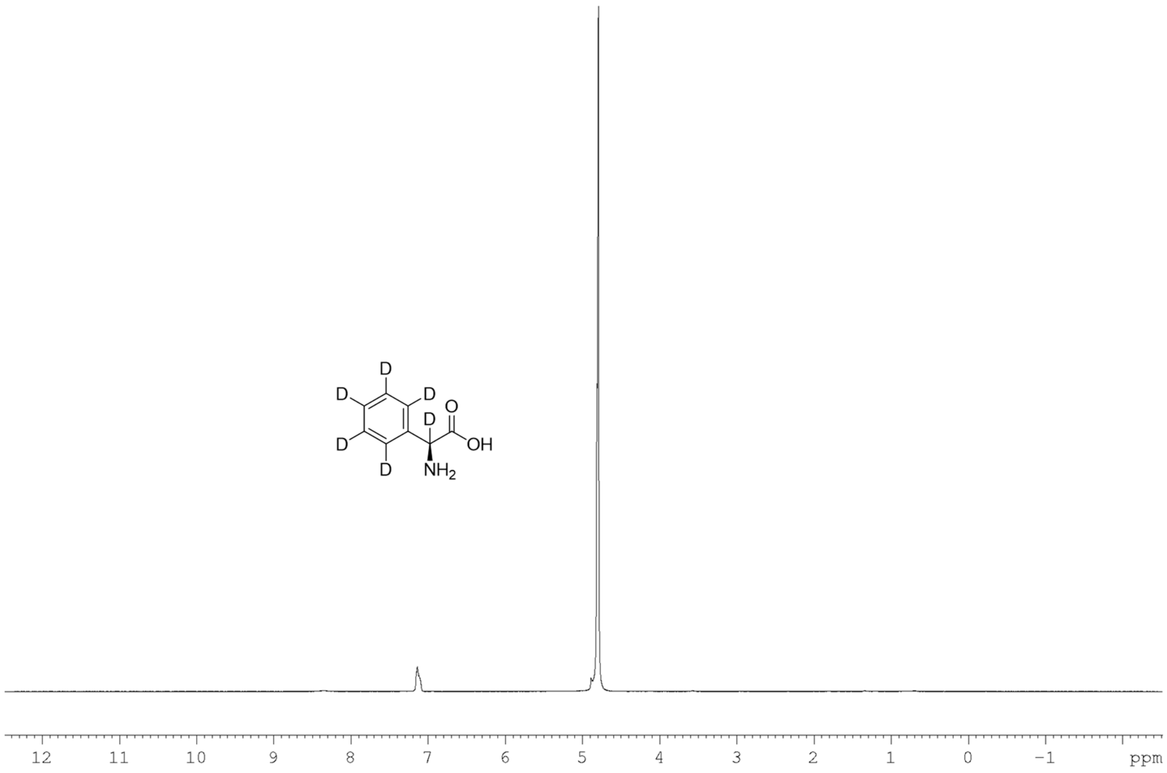

Figure S59. ^{1}H NMR spectrum of DL-phenylglycine-d_6 **51** in D_2O.

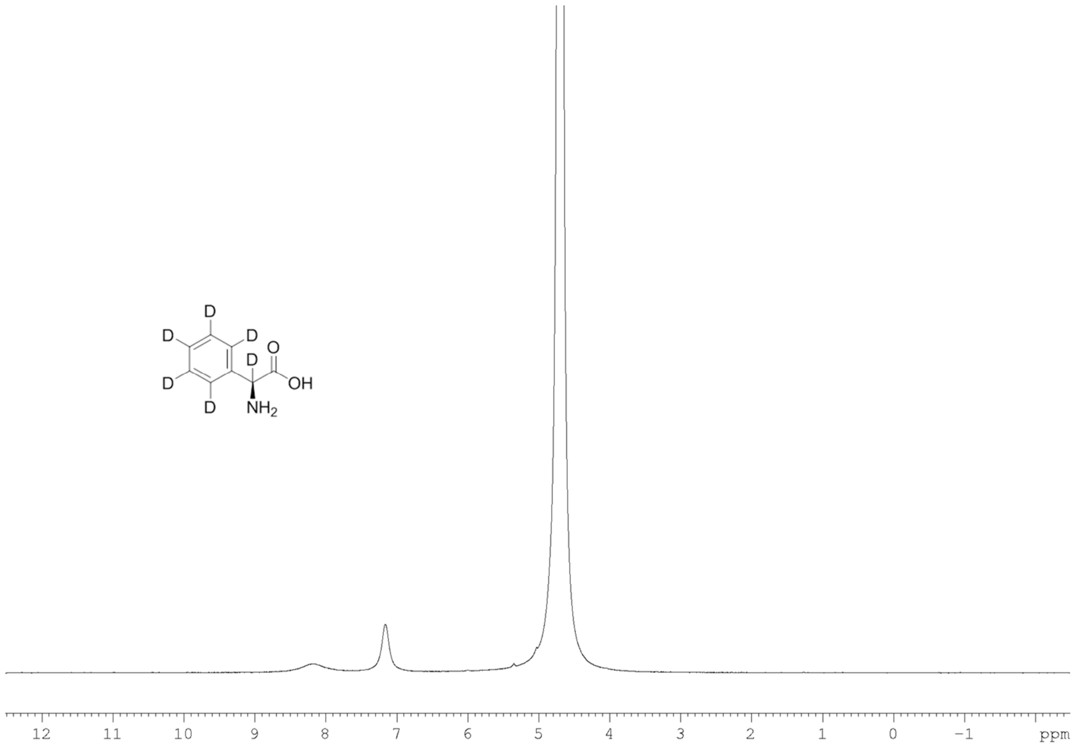

Figure S60. ^{2}H NMR spectrum of DL-phenylglycine-d_6 **S51** in D_2O.

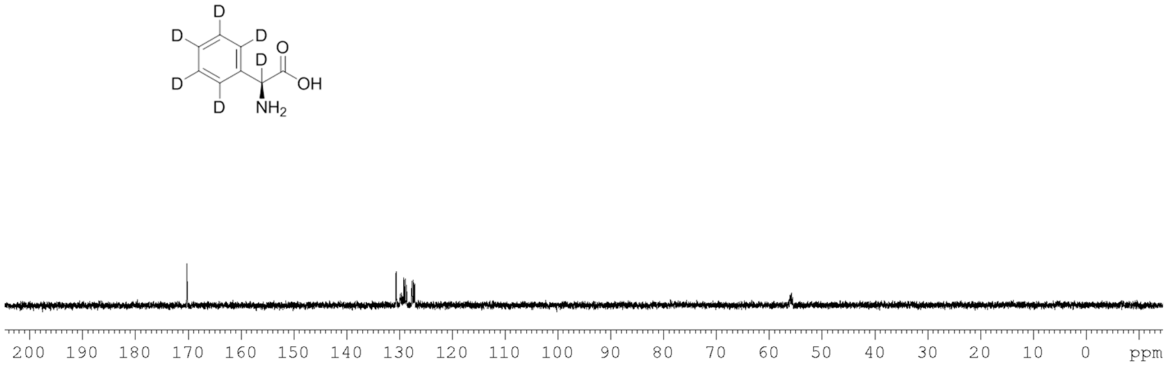

Figure S61. ^{13}C NMR spectrum of DL-phenylglycine-d$_6$ **S51** in D_2O.

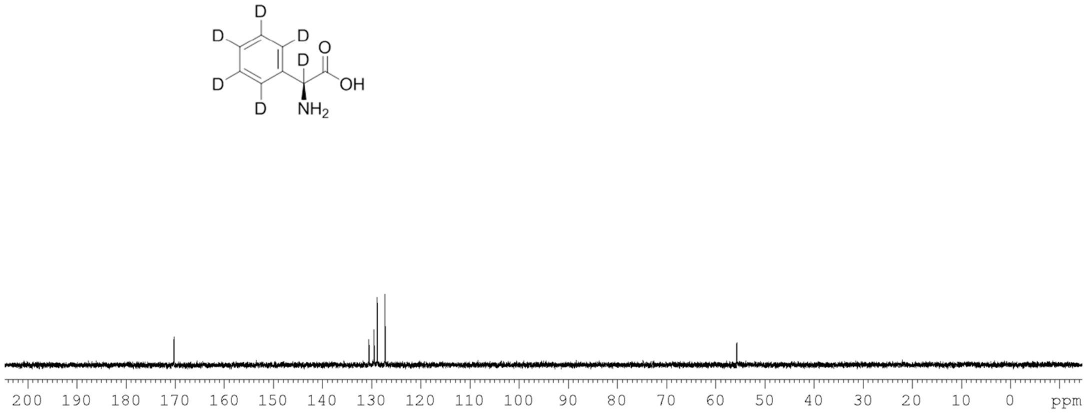

Figure S62. ^{13}C {^1H and ^2H} of DL-phenylglycine-d$_6$ **S51** in D_2O.

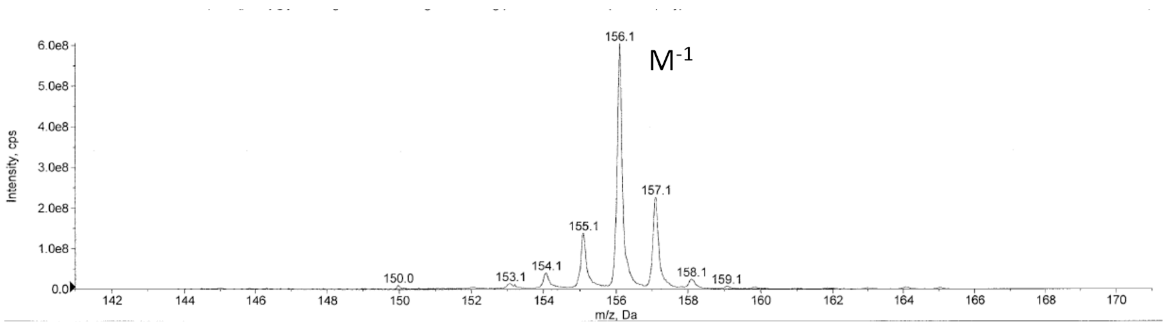

Figure S63. ESI-MS spectrum of DL-phenylglycine-d_6 **S51**, m/z 156 $[M-1]^{-}$.

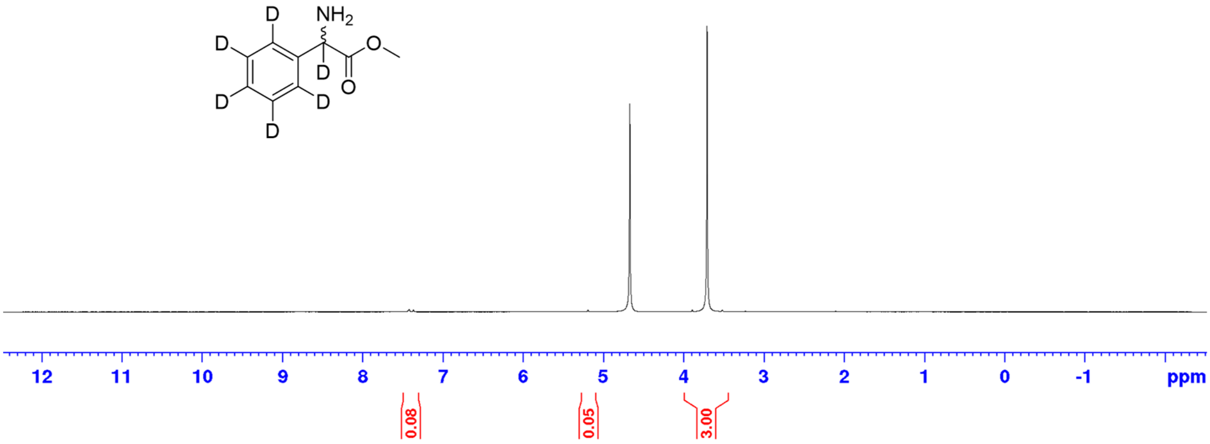

Figure S64. ^1H NMR spectrum of DL-phenylglycine-d_6 methyl ester **S52** in D_2O.

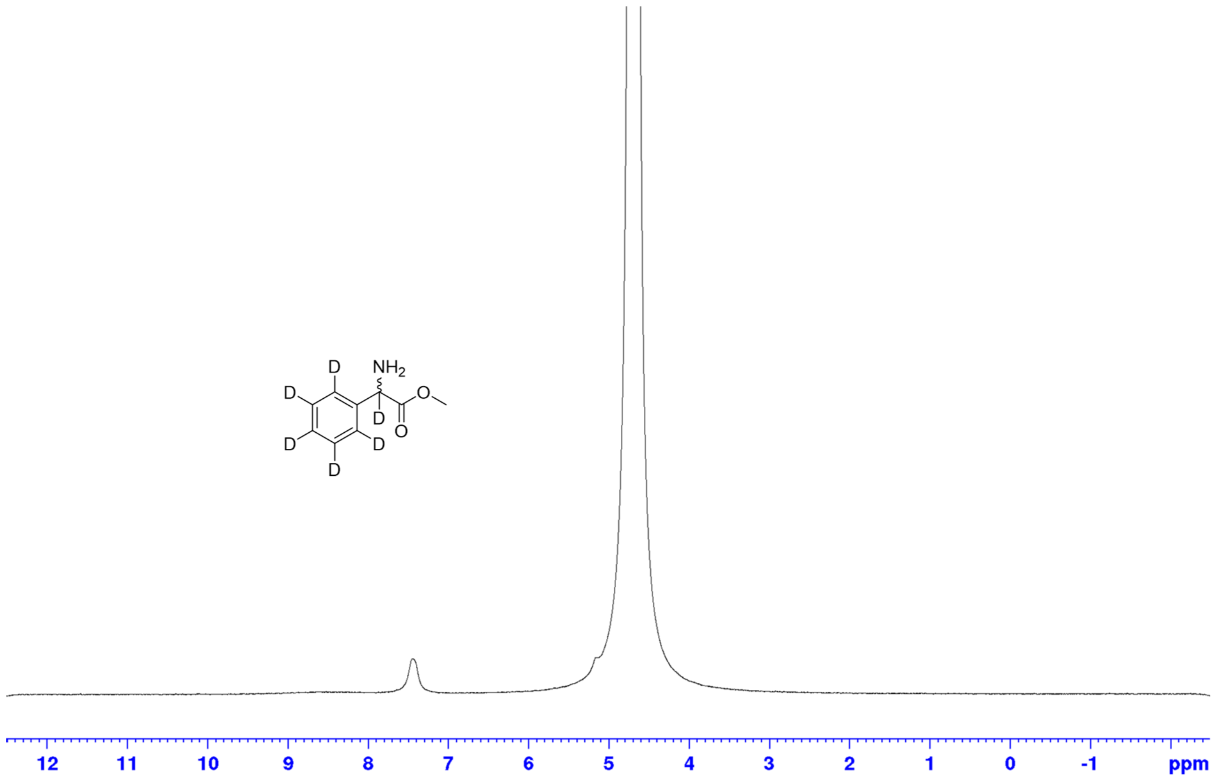

Figure S65. ^{2}H NMR spectrum of DL-phenylglycine-d$_6$ methyl ester **S52** in D_2O.

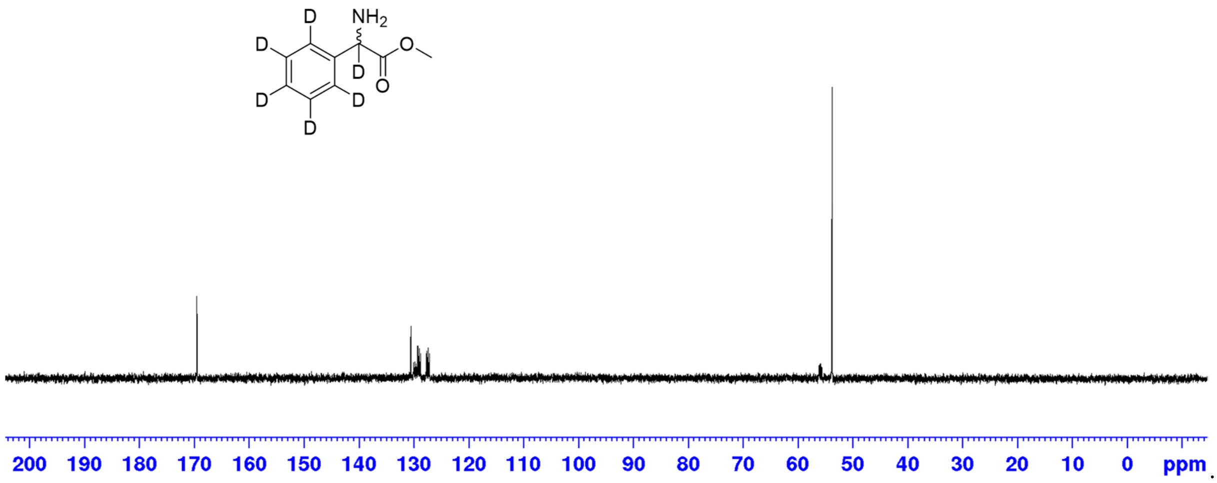

Figure S66. ^{13}C NMR spectrum of DL-phenylglycine-d$_6$ methyl ester **S52** in D_2O.

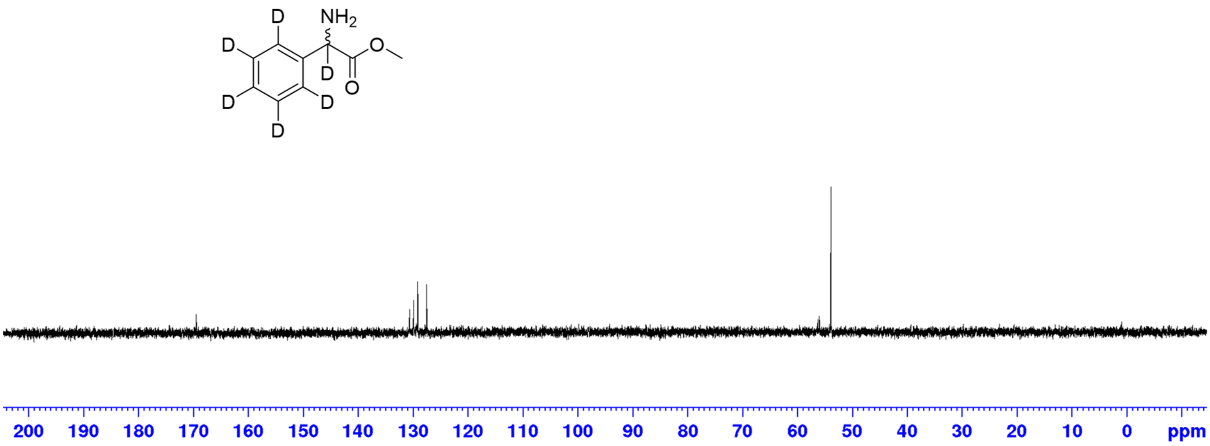

Figure S67. ^{1}H NMR spectrum of DL-phenylglycine-d$_6$ methyl ester **S52** in D_2O.

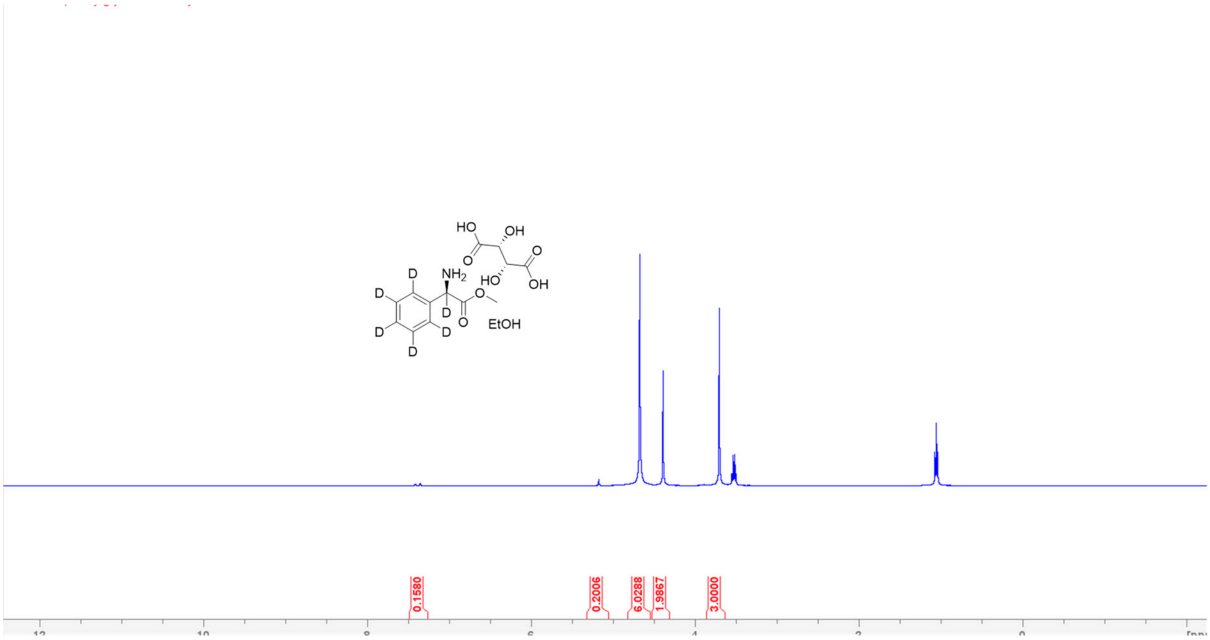

Figure S68. ^{1}H NMR spectrum of DL-phenylglycine-d$_6$ methyl ester tartrate salt **S53** in D_2O.

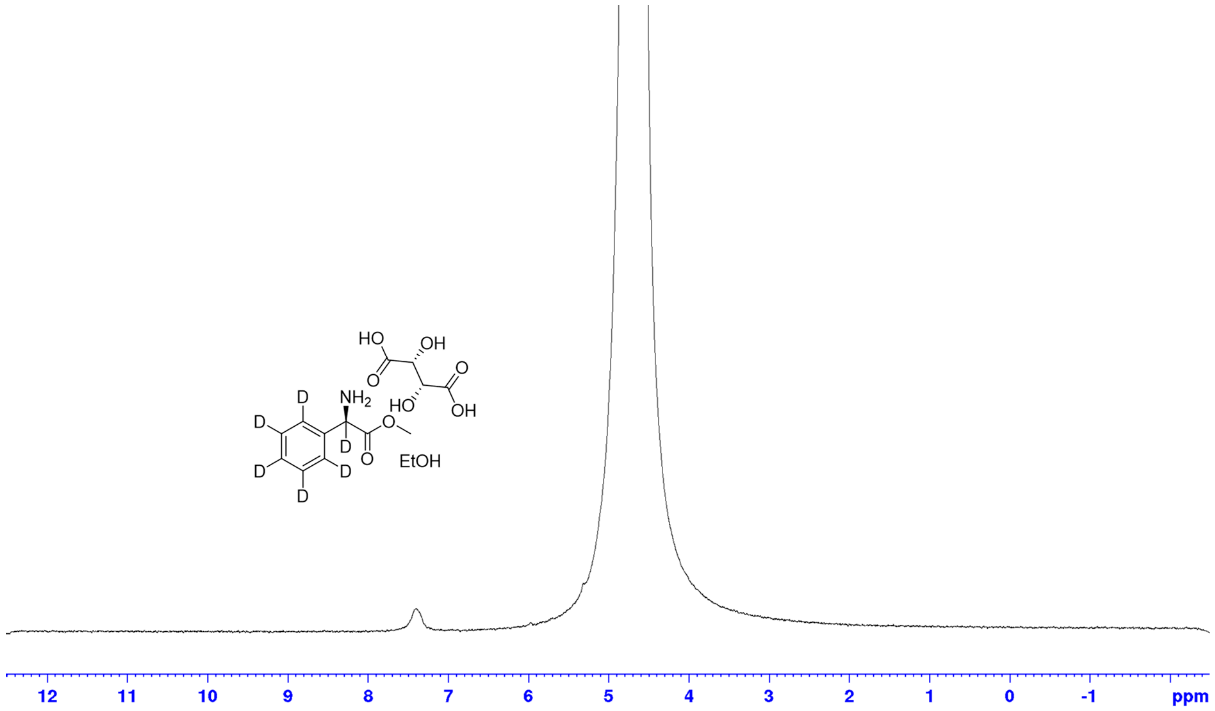

Figure S69. ^{2}H NMR spectrum of DL-phenylglycine-d$_6$ methyl ester tartrate salt **S53** in D_2O.

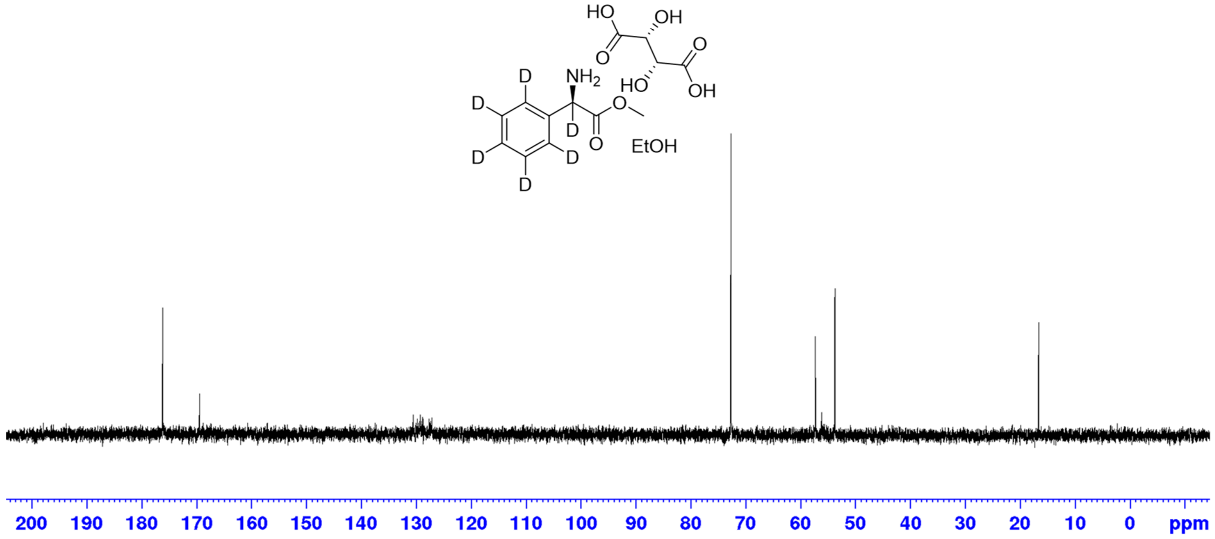

Figure S70. ^{13}C NMR spectrum of DL-phenylglycine-d$_6$ methyl ester tartrate salt **S53** in D_2O.

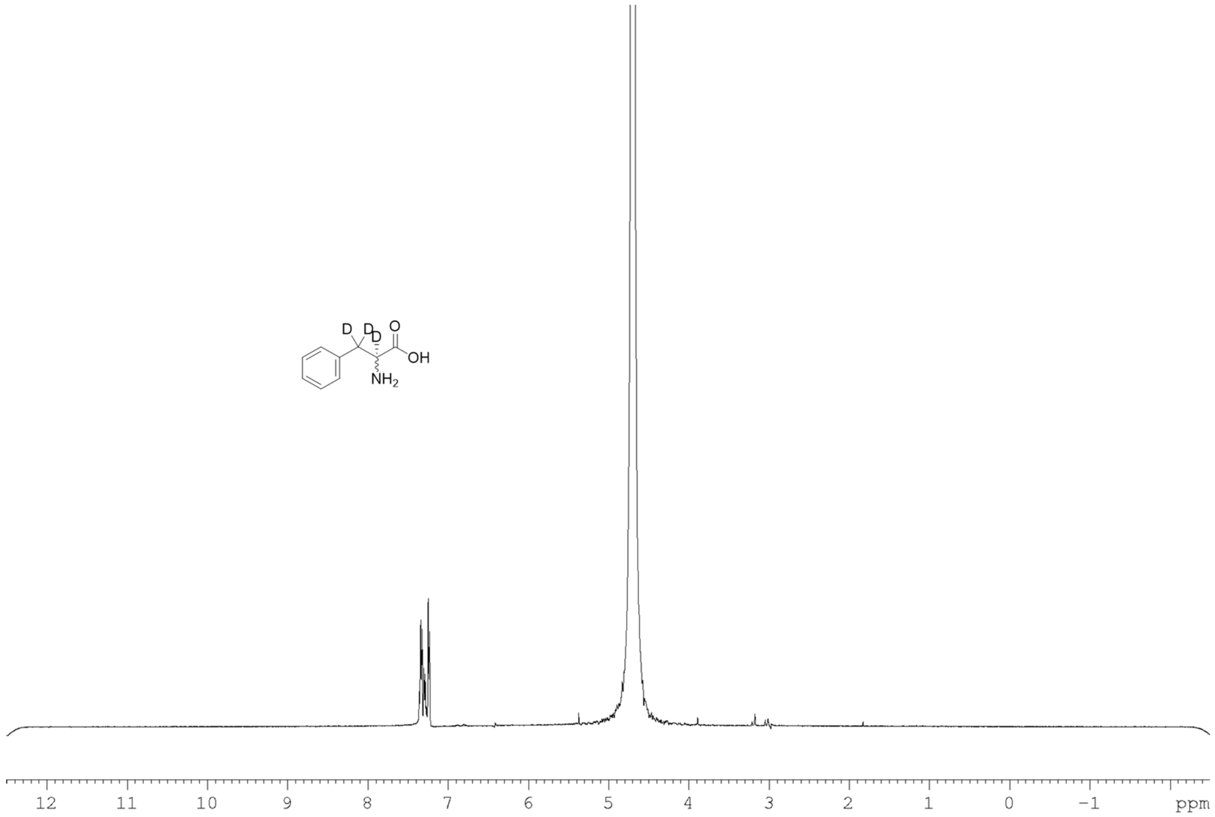

Figure S71. ^{1}H NMR spectrum of DL-phenylalanine-d_3 **S55** in D_2O.

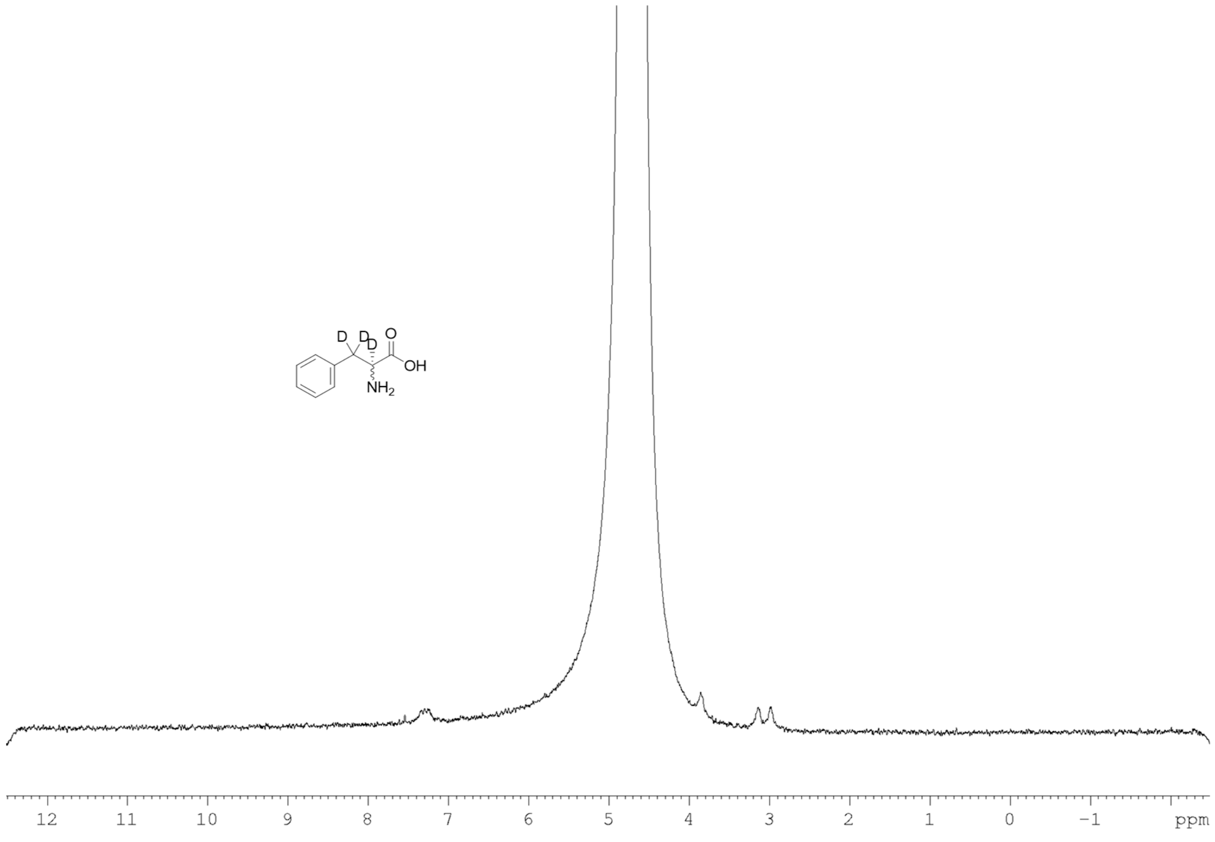

Figure S72. ^{2}H NMR spectrum of DL-phenylalanine-d_3 **S55** in D_2O.

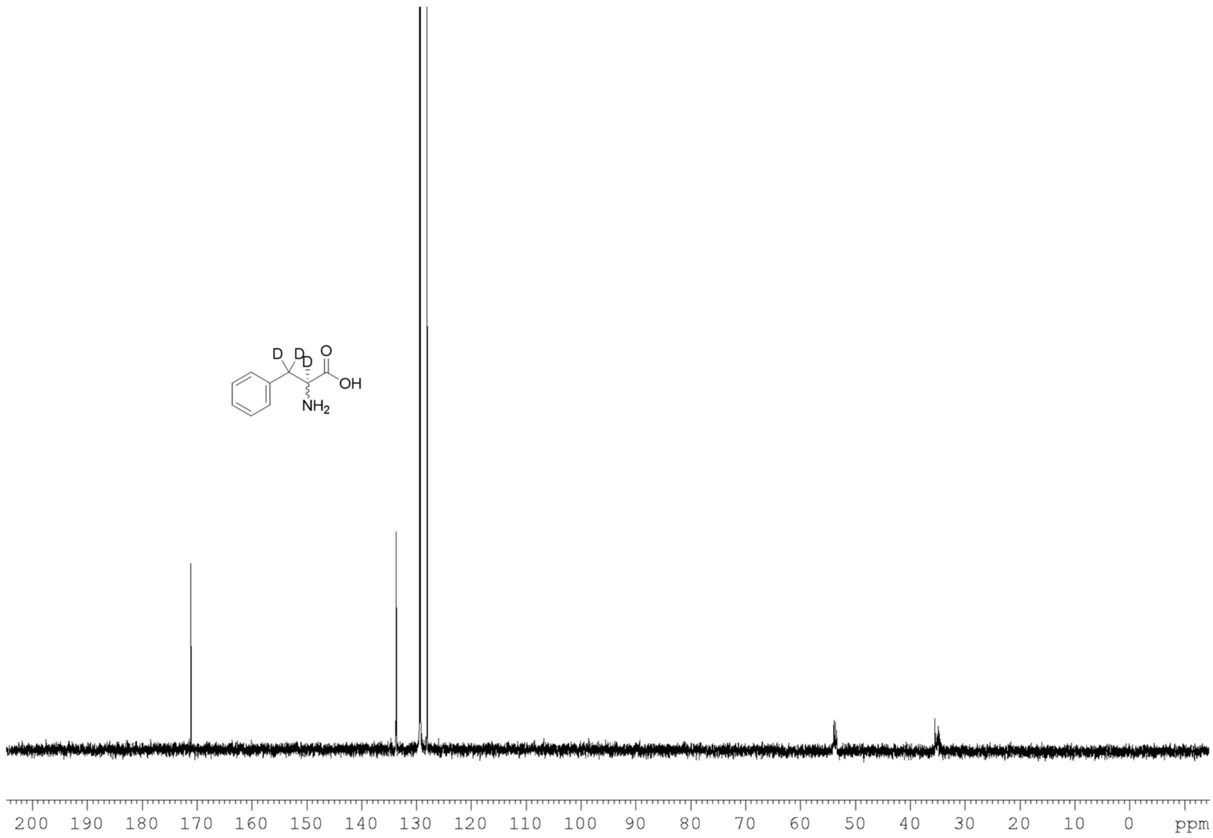

Figure S73. ^{13}C NMR spectrum of DL-phenylalanine-d_3 **S55** in D_2O.

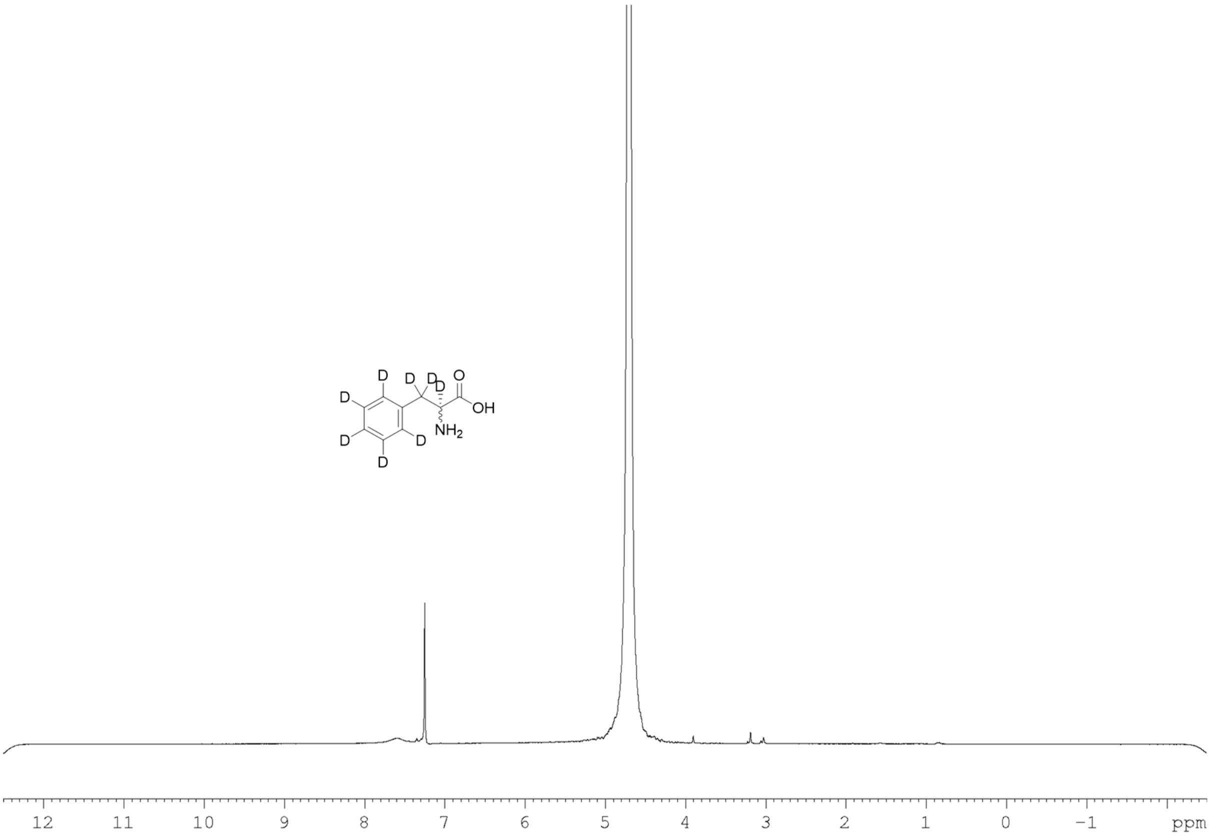

Figure S74. ^{1}H NMR spectrum of DL-phenylalanine-d_8 **S56** in D_2O.

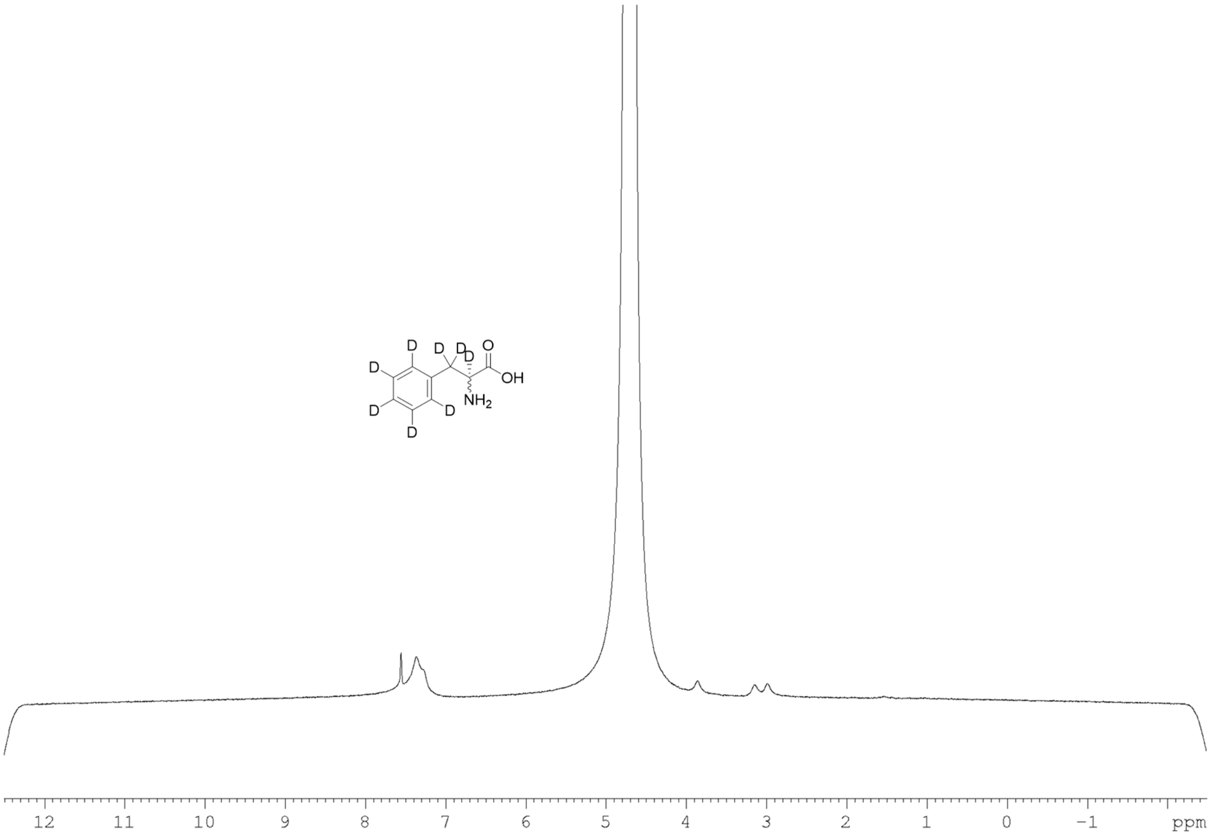

Figure S75. ^{2}H NMR spectrum of DL-phenylalanine-d$_8$ **S56** in D_2O.

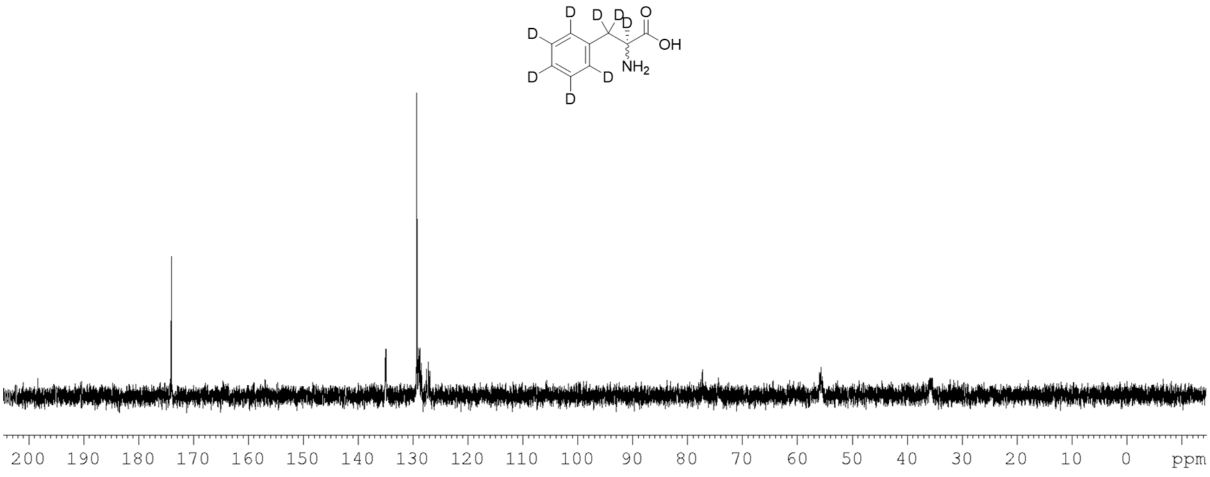

Figure S76. ^{13}C NMR spectrum of DL-phenylalanine-d$_8$ **S56** in D_2O.

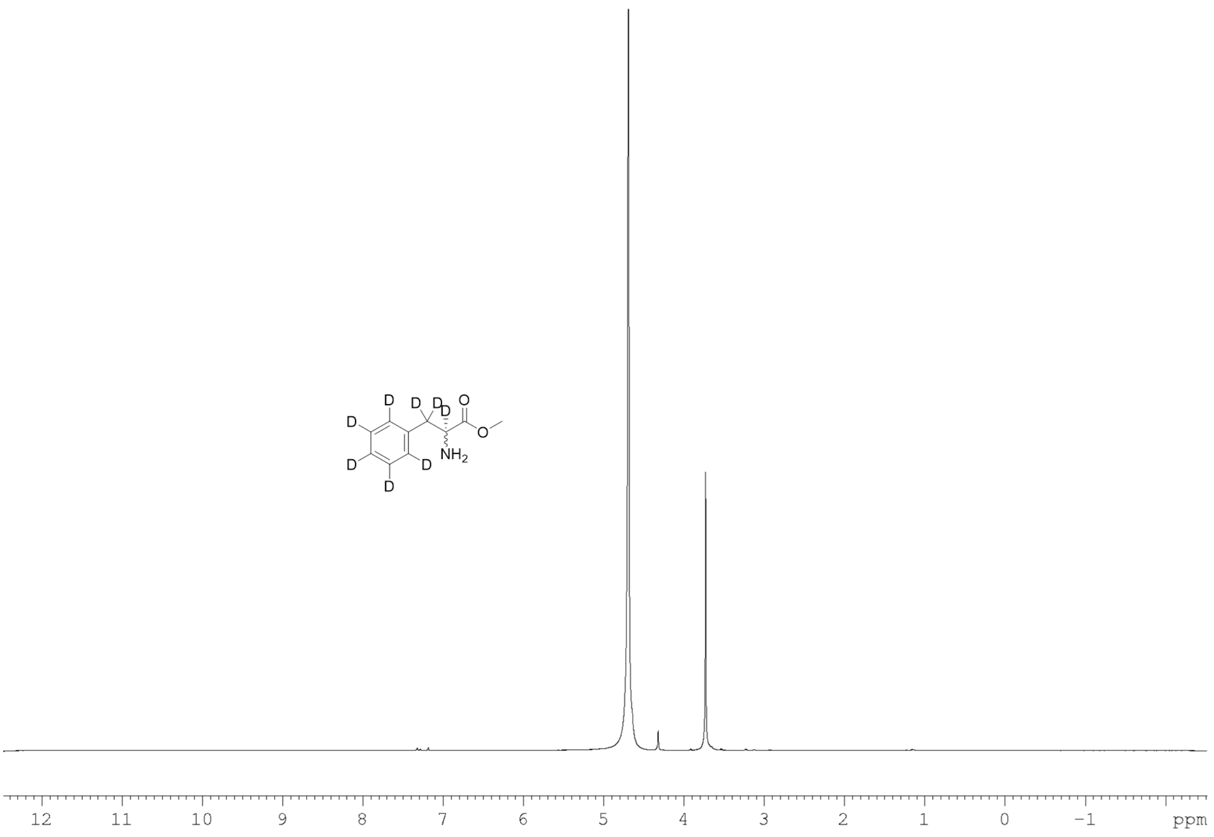

Figure S77. ^{1}H NMR spectrum of DL-phenylalanine-d8 methyl ester **S56a** in D_2O.

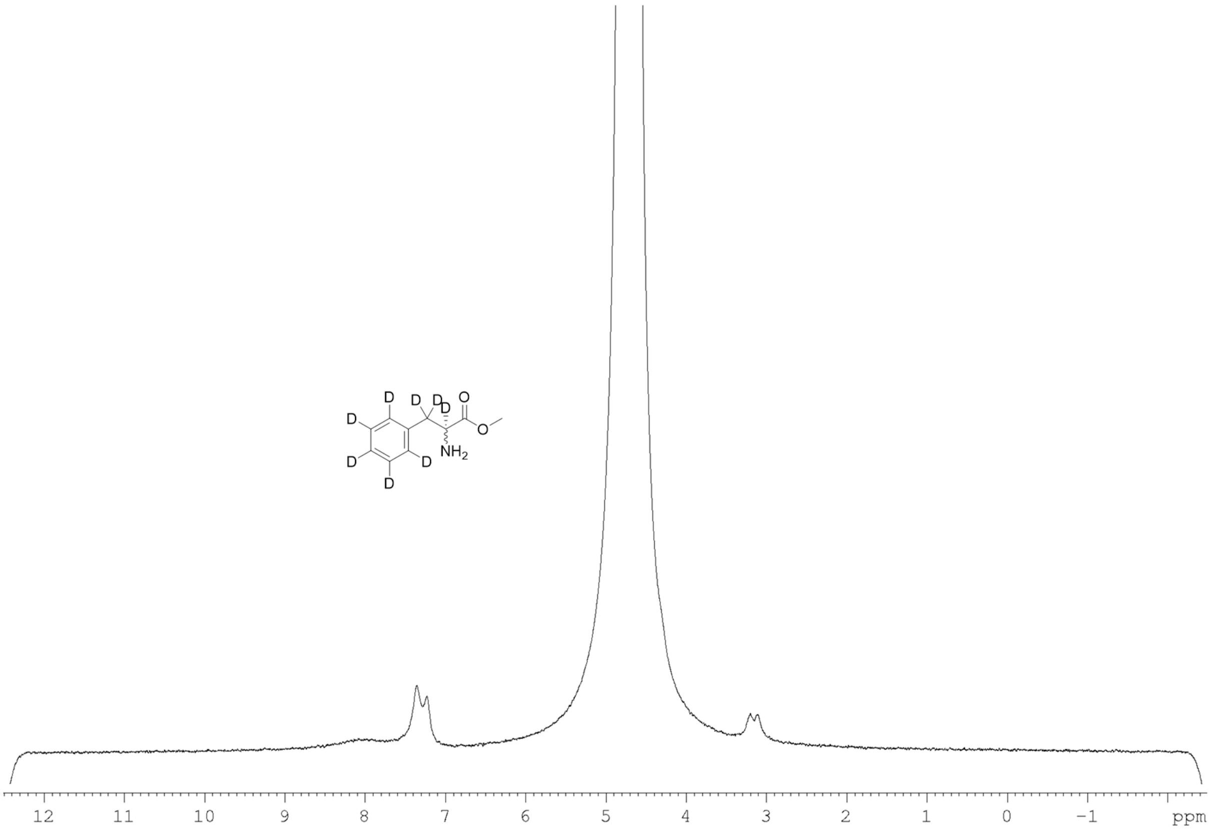

Figure S78. ^{1}H NMR spectrum of DL-phenylalanine-d8 methyl ester **S56a** in D_2O.

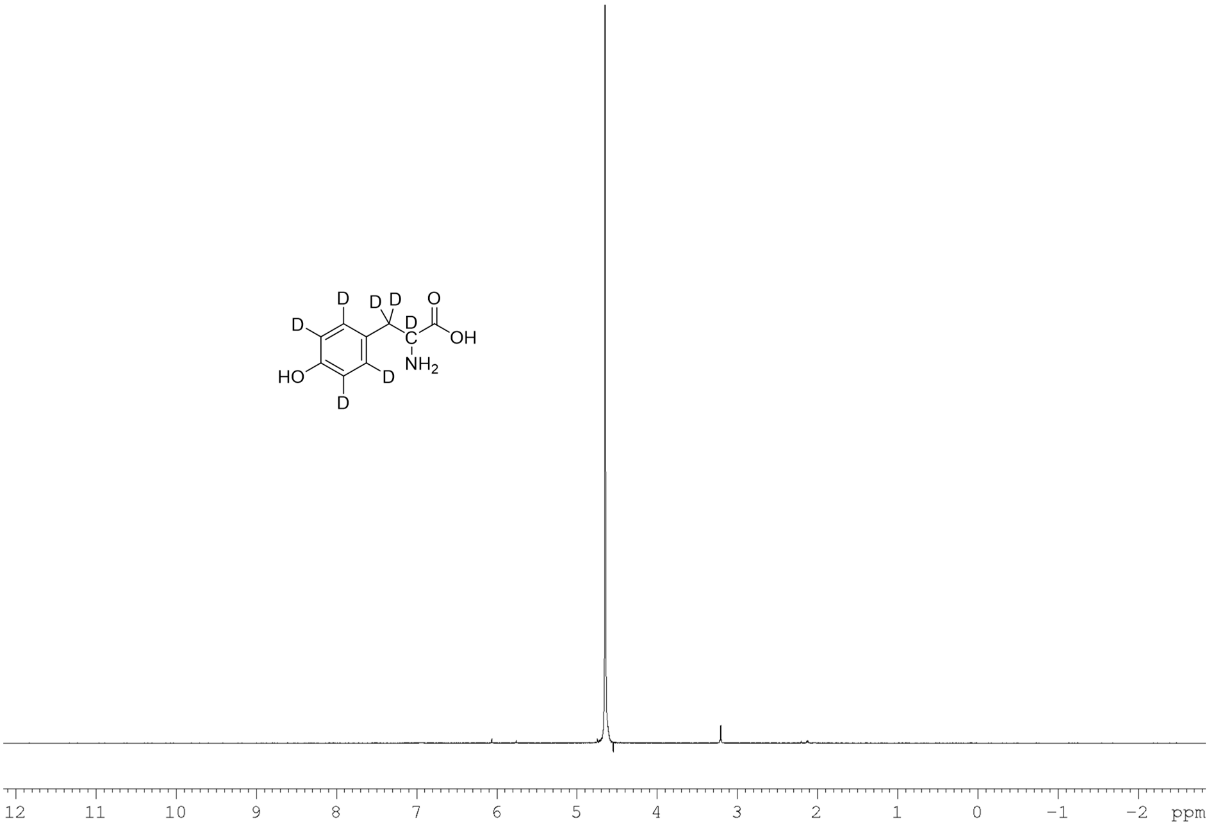

Figure S79. ^{1}H NMR spectrum of DL-tyrosine-d$_7$ **S58** in D_2O.

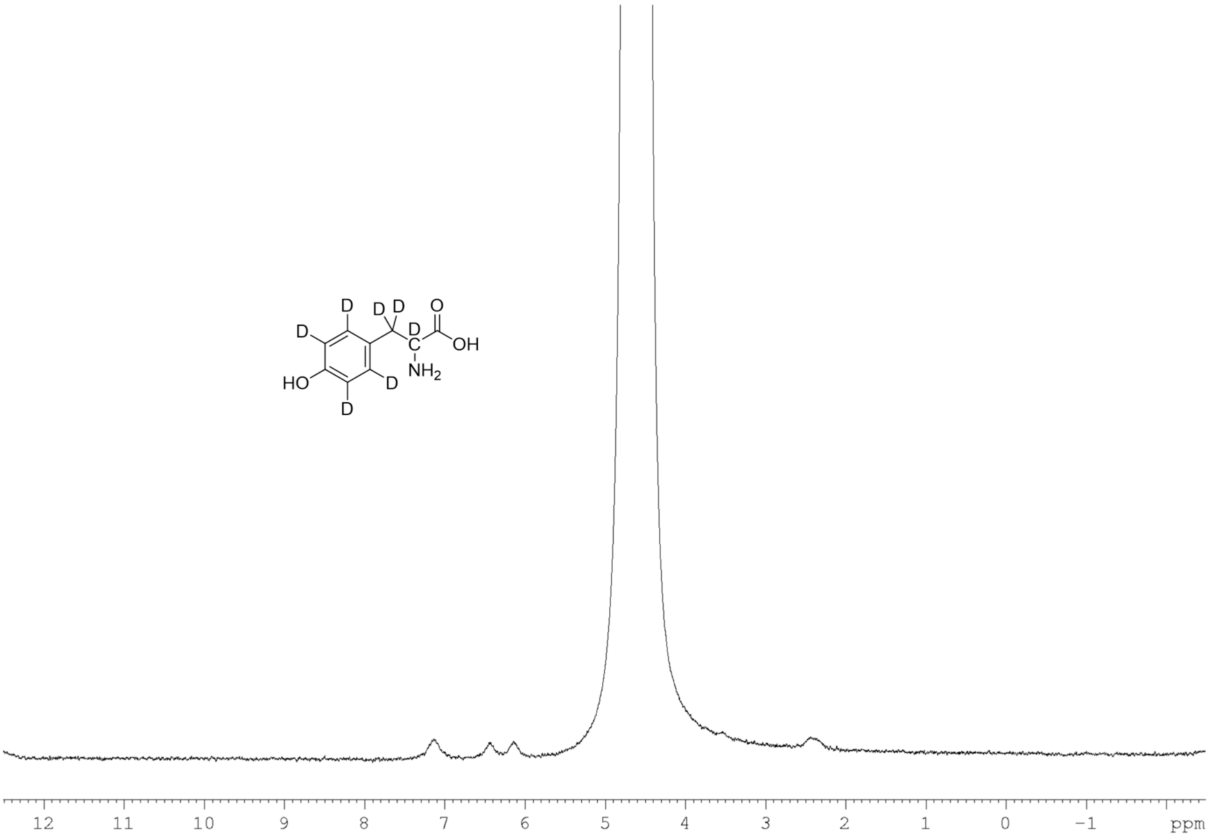

Figure S80. ^{2}H NMR spectrum of DL-tyrosine-d$_7$ **S58** in D_2O.

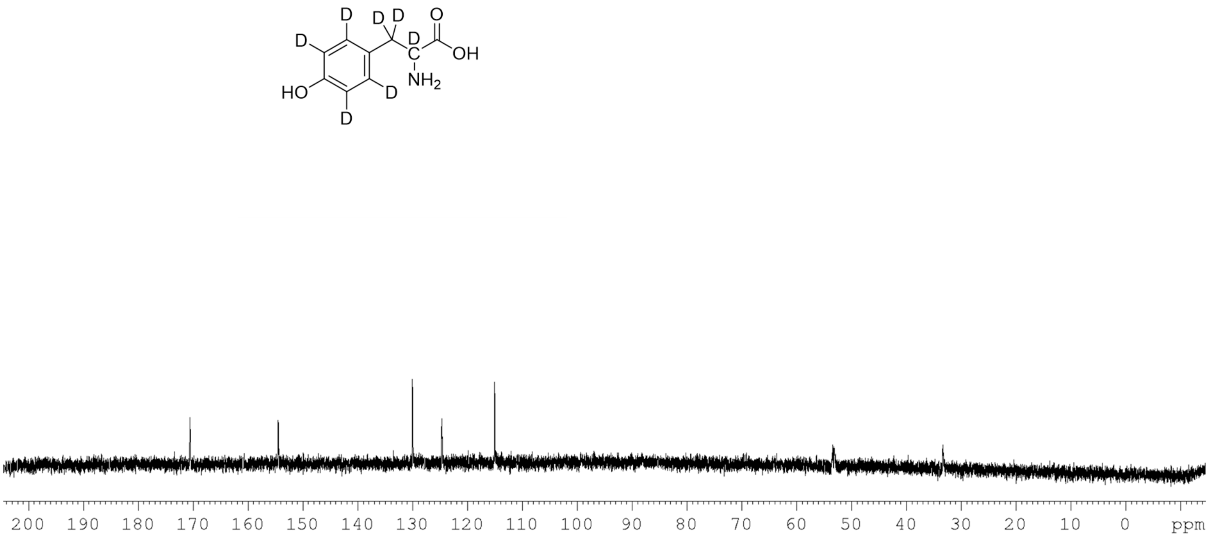

Figure S81. ^{13}C {^{1}H and ^{2}H} spectrum of DL-tyrosine-d_7 **S58** in D_2O.

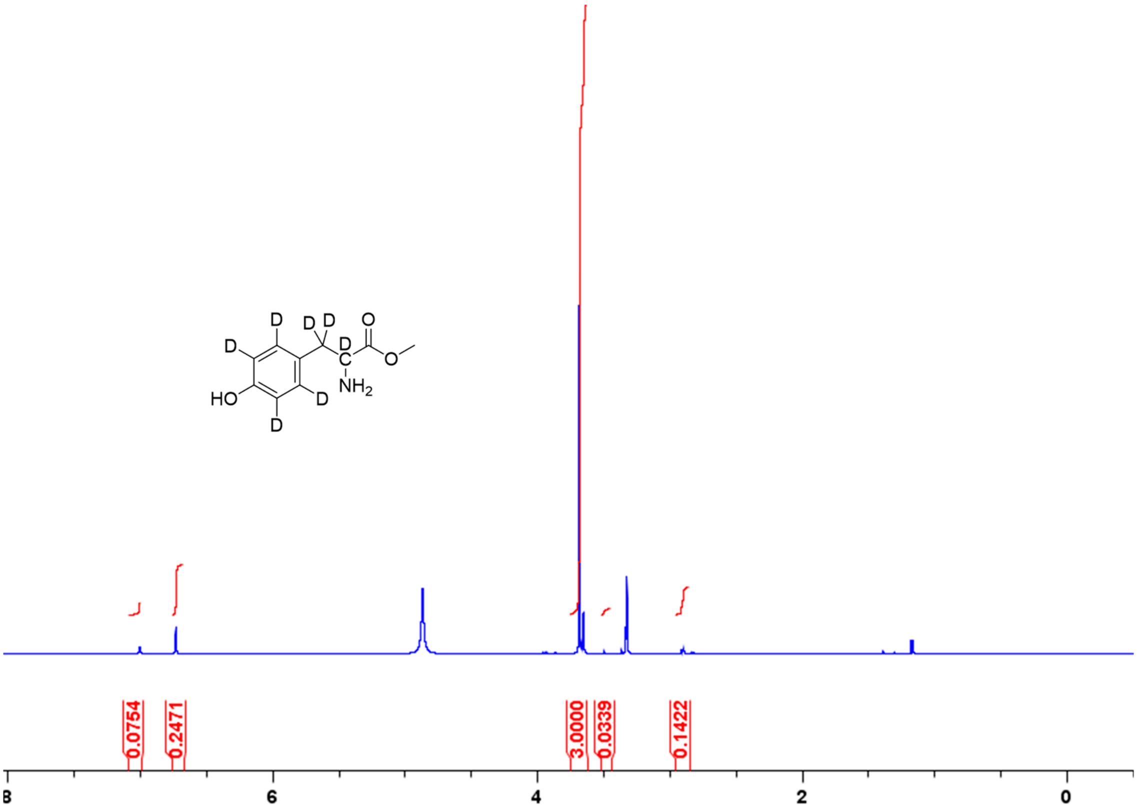

Figure S82. ^{1}H NMR spectrum of DL-tyrosine-d_7 methyl ester **S59** in CD_3OD.

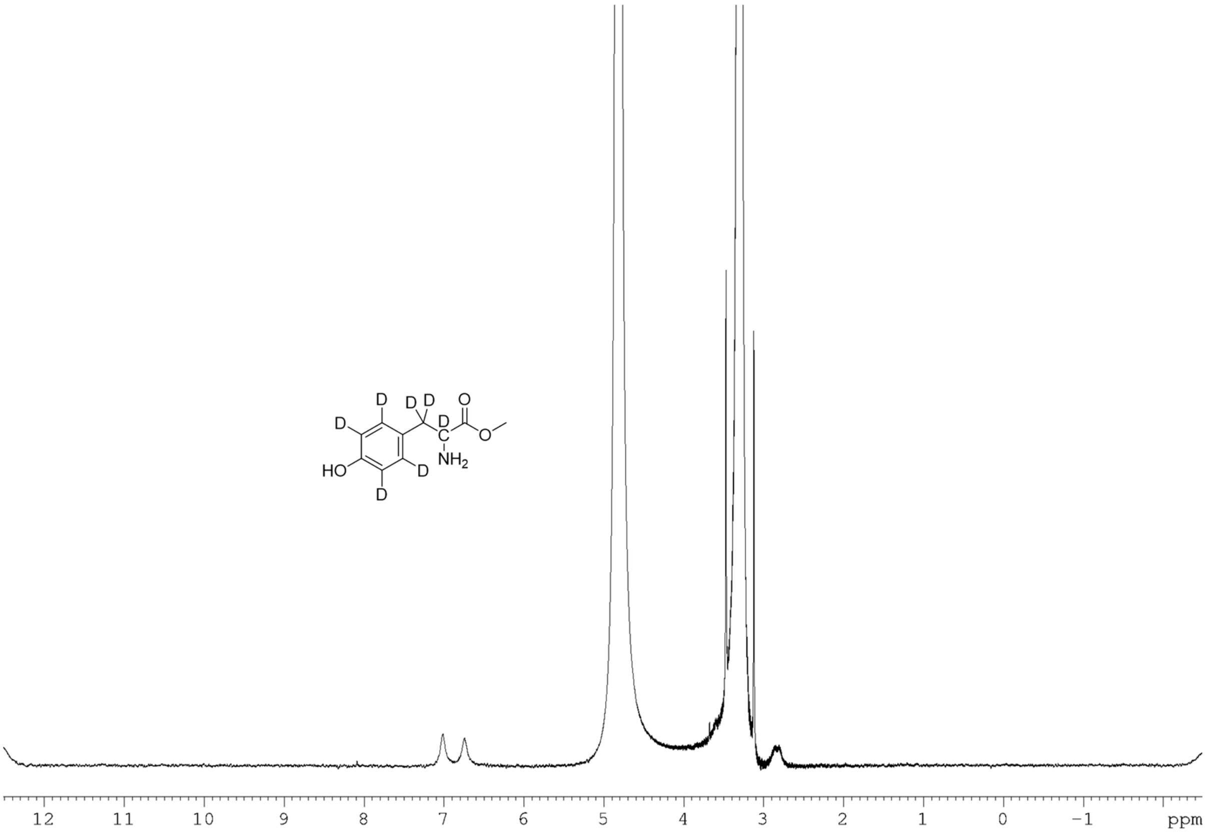

Figure S83. ^{2}H NMR spectrum of DL-tyrosine-d_7 methyl ester **S59** in CD_3OD.

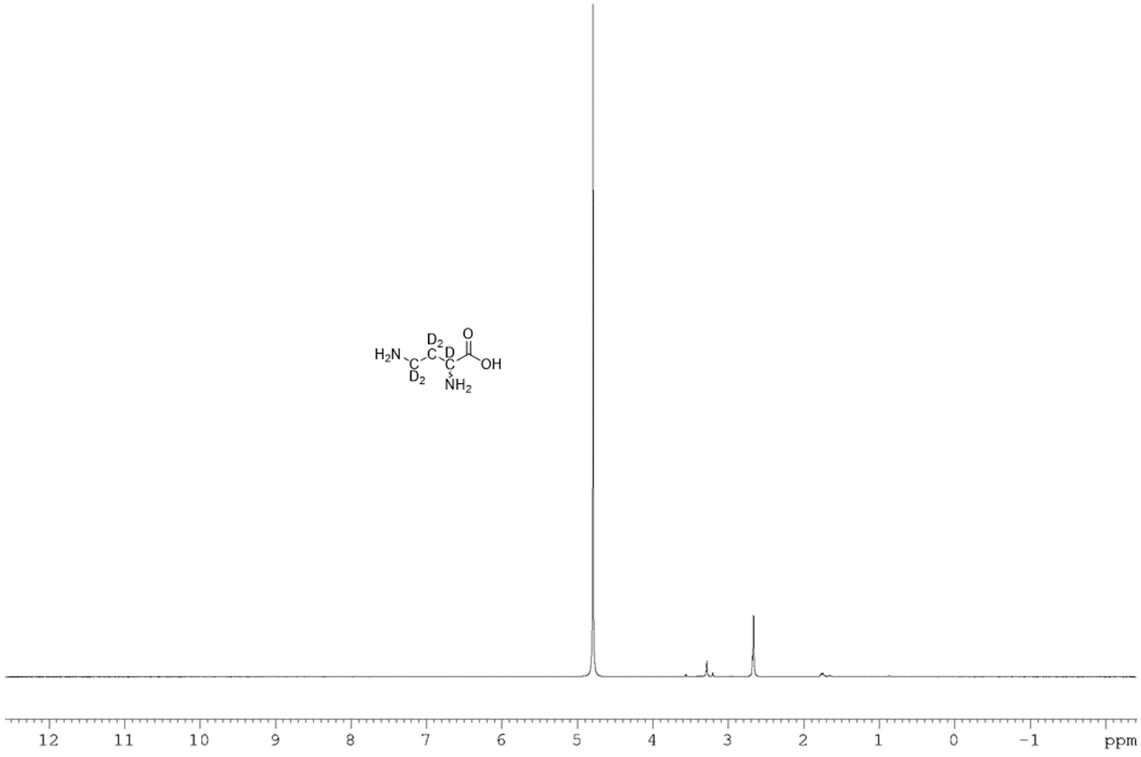

Figure S84. ^{1}H NMR spectrum of DL-2,4-diaminobutyric acid-d_5 **S81** in D_2O.

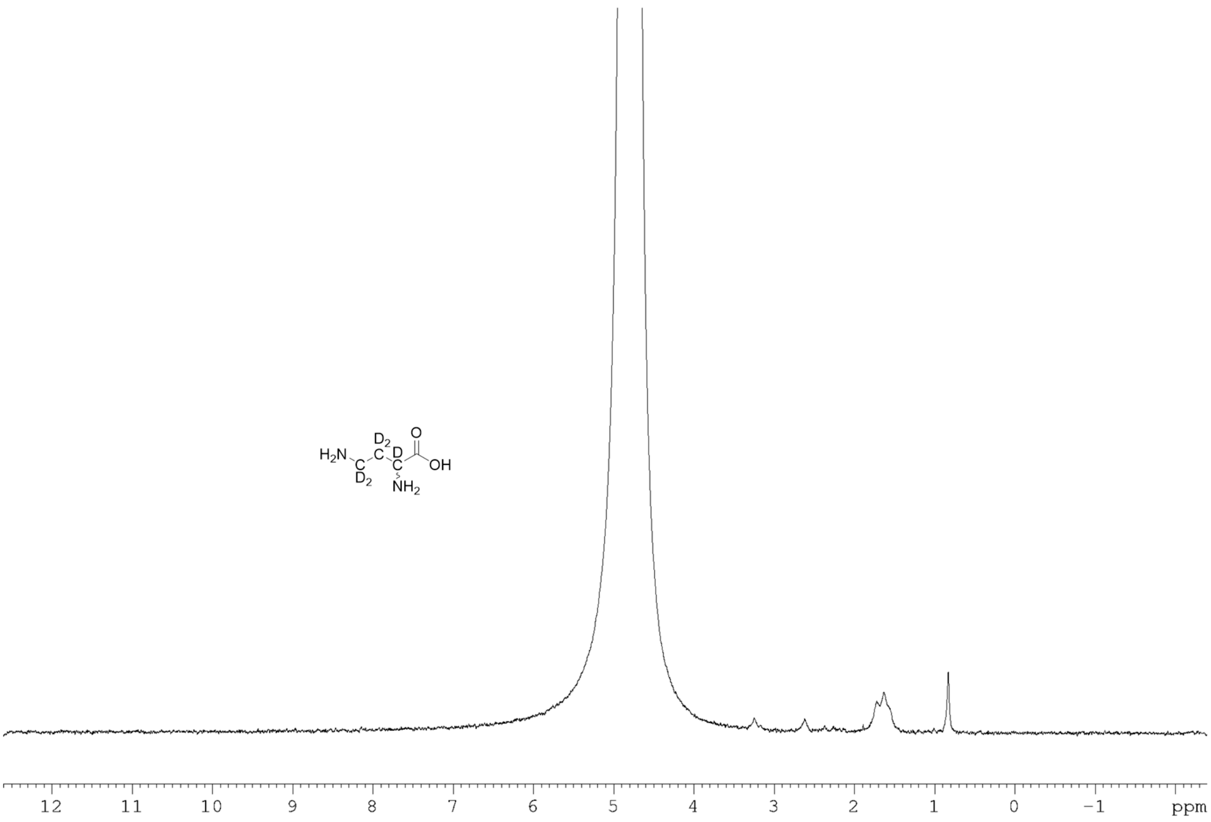

Figure S85. ^{2}H NMR spectrum of DL-2,4-diaminobutyric acid-d_5 **S81** in D_2O.

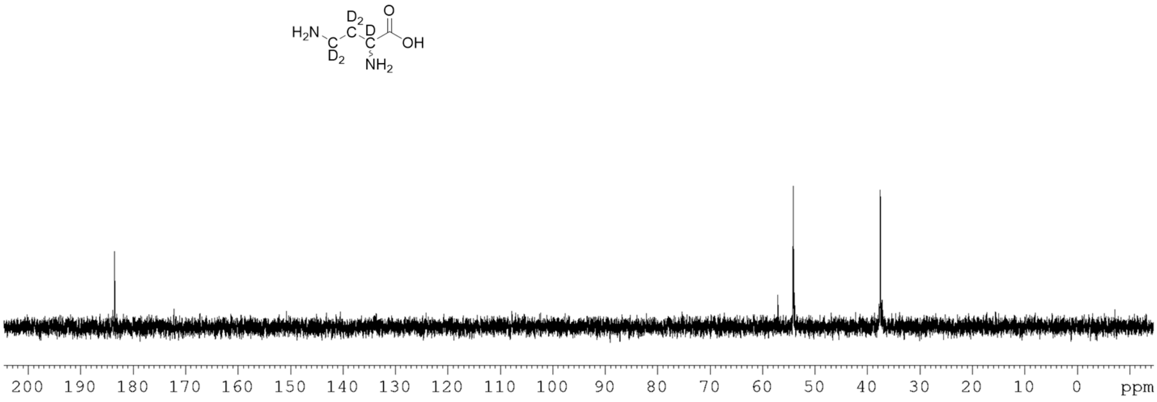

Figure S86. ^{13}C NMR spectrum of DL-2,4-diaminobutyric acid-d_5 **S81** in D_2O.

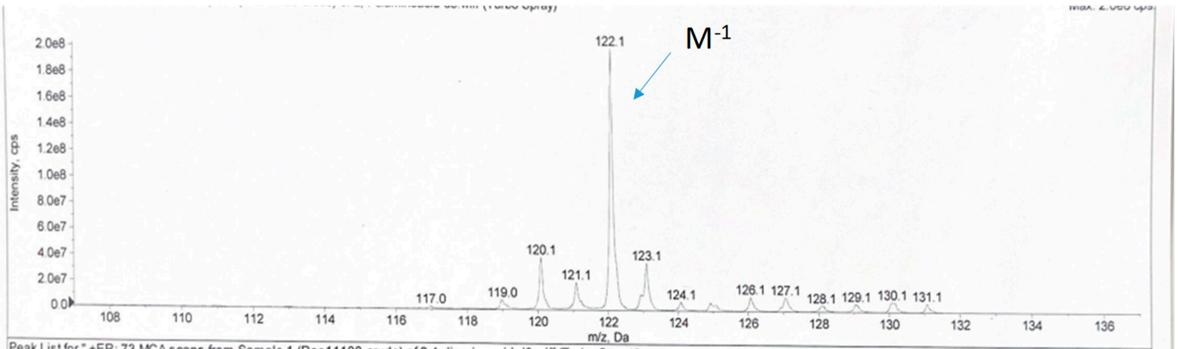

Peak List for " +ER: 73 MCA scans from Sample 1 (Rao11100-crude) of 2,4-diaminoacid-d3.wiff (Turbo Spray)"

	m/z (Da)	Intensity (cps)	Peak area (counts)
1	116.9700	2.2771e6	1.0965e7
2	118.9800	7.6184e6	5.1577e7
3	120.0900	4.1287e7	1.9151e8
4	121.0800	2.1972e7	1.3453e8
5	122.1000	2.0215e8	9.3237e8
6	123.0900	3.7510e7	2.3390e8
7	124.0800	7.0006e6	3.8925e7
8	124.9200	6.5536e6	5.3699e7
9	126.0600	1.1500e7	6.1325e7
10	127.0500	1.0995e7	7.4226e7
11	128.0700	4.8640e6	3.0788e7
12	129.0600	6.2124e6	4.1907e7
13	130.0800	7.9033e6	6.3011e7
14	131.0700	6.8783e6	3.3756e7

Figure S87. ESMS m/z 122 [M-1]$^-$ mass spectrum of **S81**.

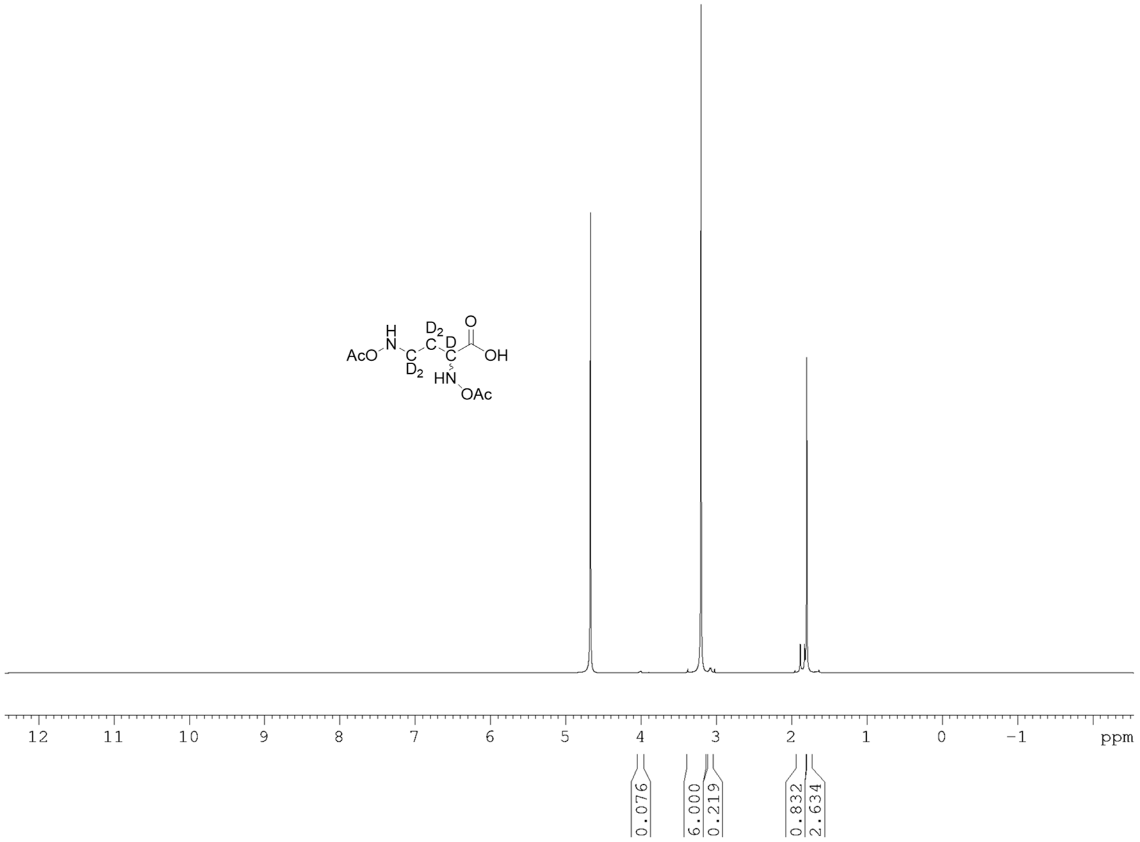

Figure S88. ^{1}H NMR spectrum of DL-2,4-di-N-acetylbutyric acid-d_5 **S82** in D_2O.

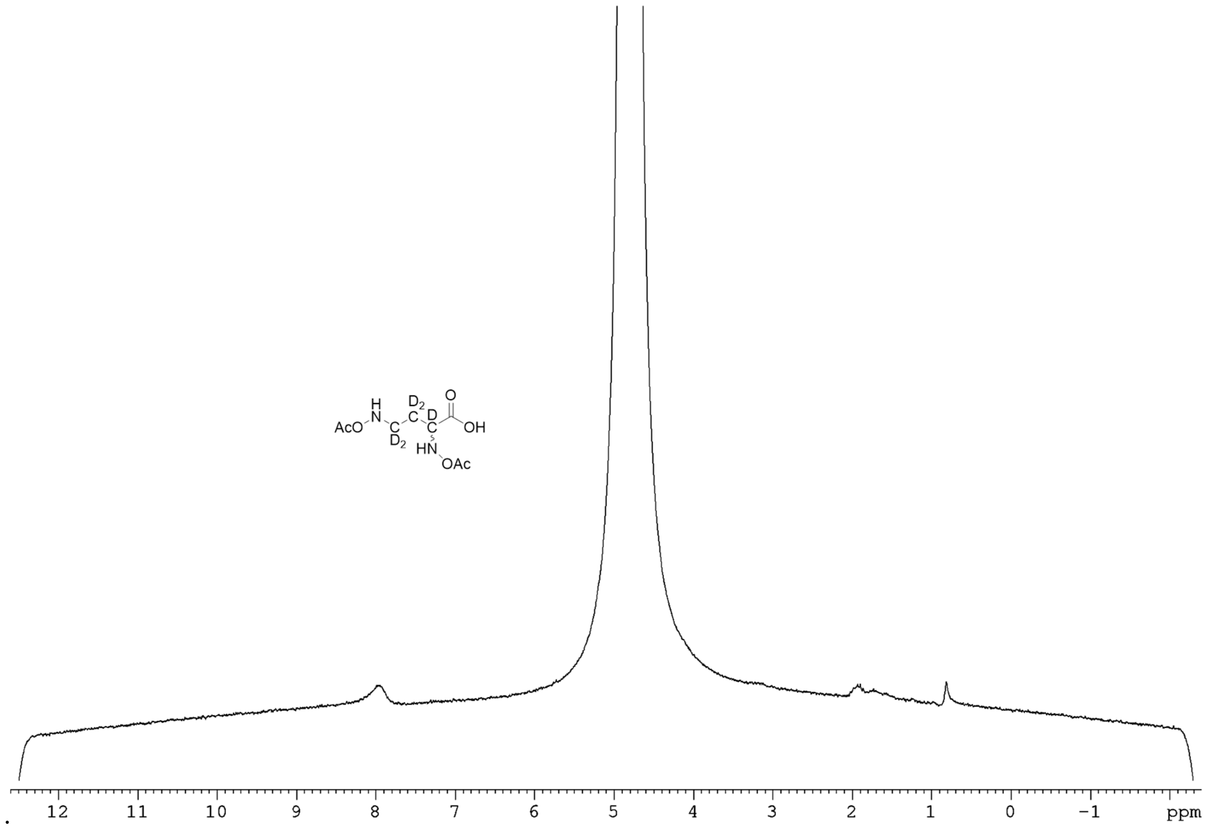

Figure S89. ^{2}H NMR spectrum of DL-2,4-di-N-acetylbutyric acid-d_5 **S82** in D_2O.

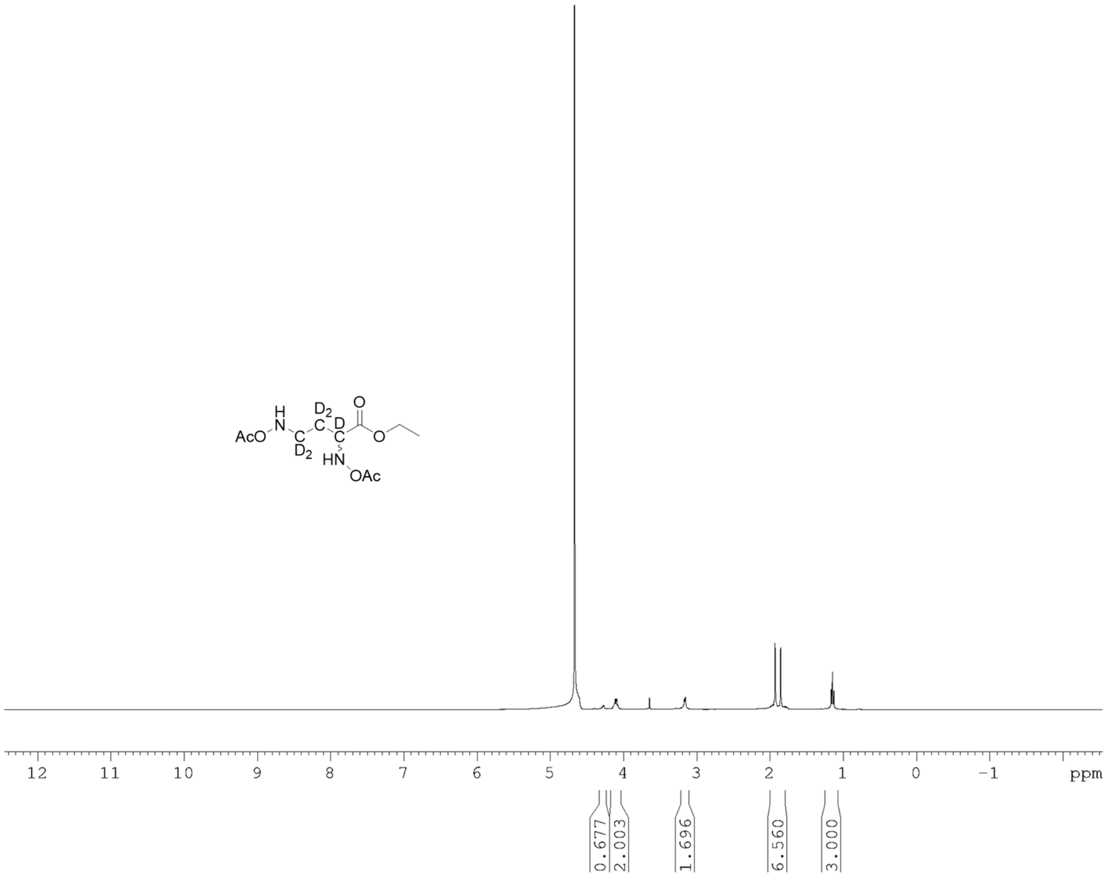

Figure S90. ^{1}H NMR spectrum of DL-2,4- N′N-diacetylbutyric acid-d_5 ethyl ester **S83** in D_2O.

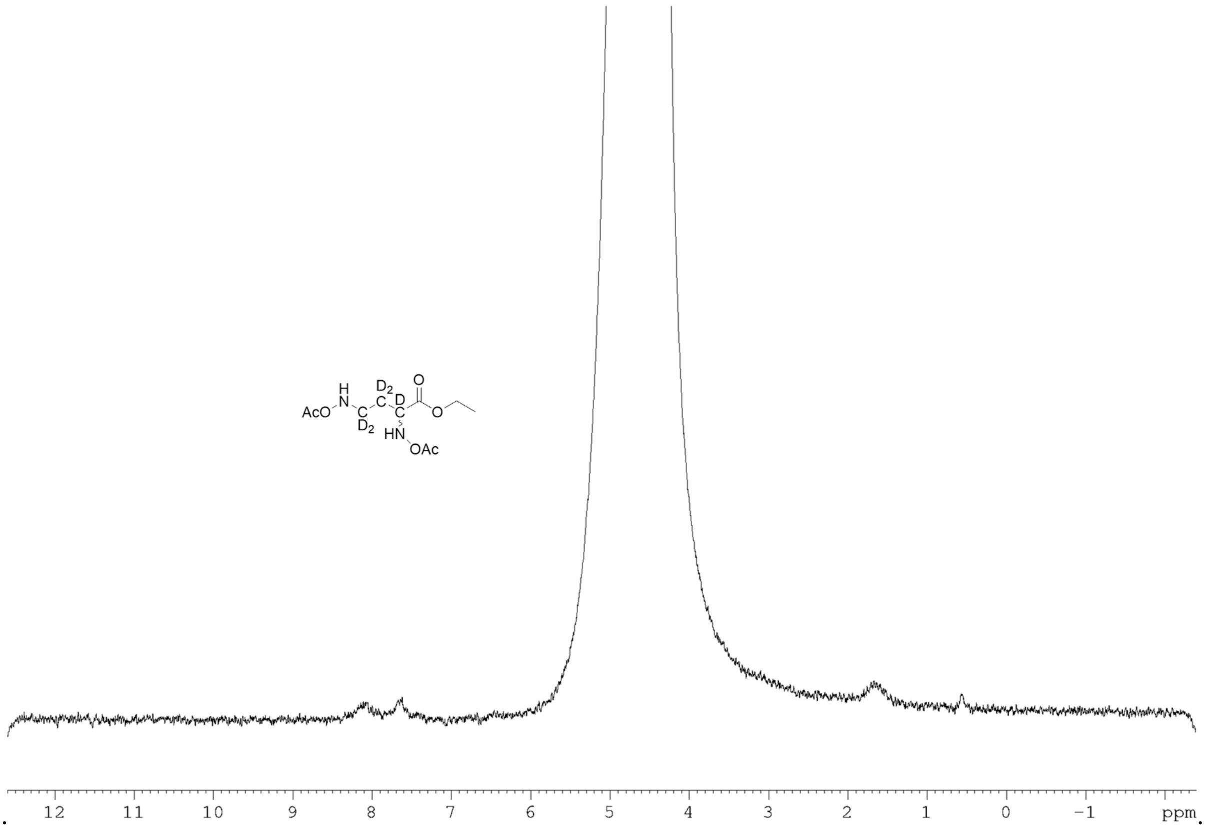

Figure S91. ^{2}H NMR spectrum of DL-2,4- N′N-diacetylbutyric acid-d_5 ethyl ester **S83** in D_2O.

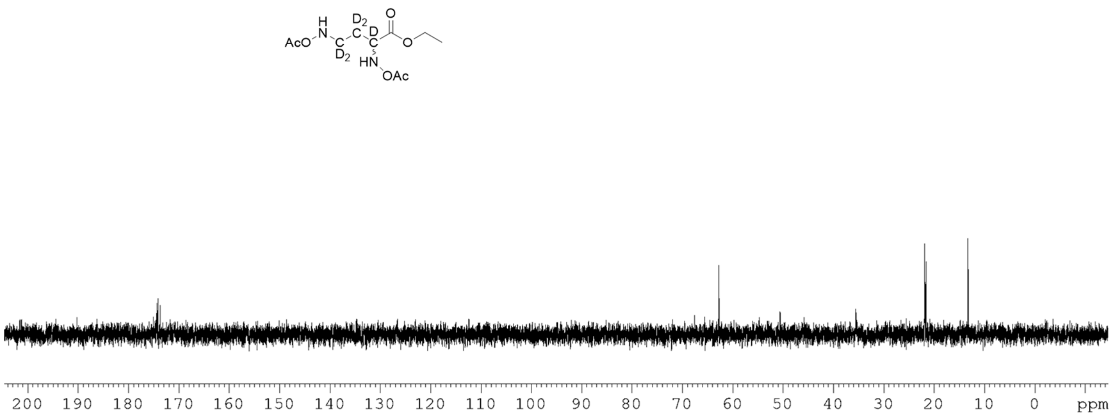

Figure S92. ^{13}C NMR spectrum of DL-2,4- N′N-diacetylbutyric acid-d_5 ethyl ester **S83** in D_2O.

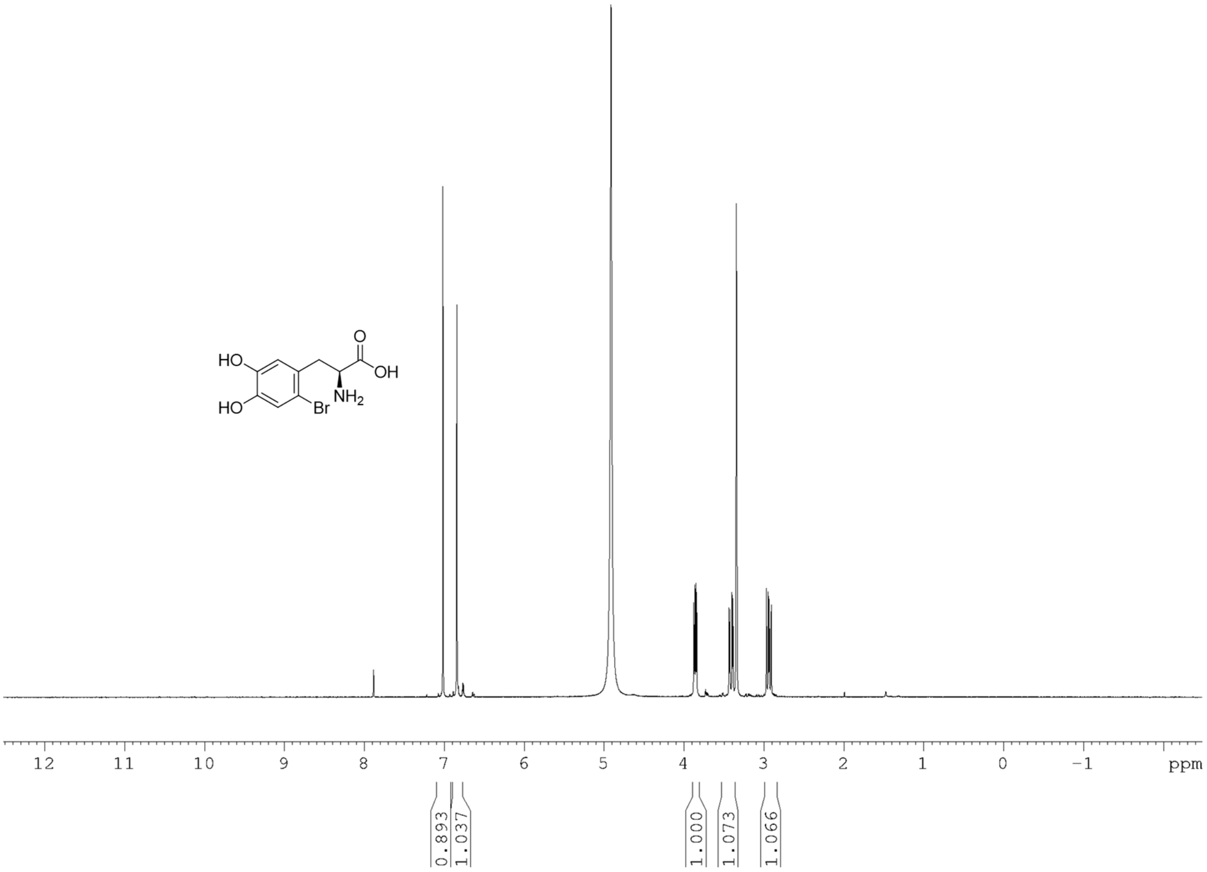

Figure S93. ^{1}H NMR spectrum of Br-L-DOPA **S15** in CD_3OD.

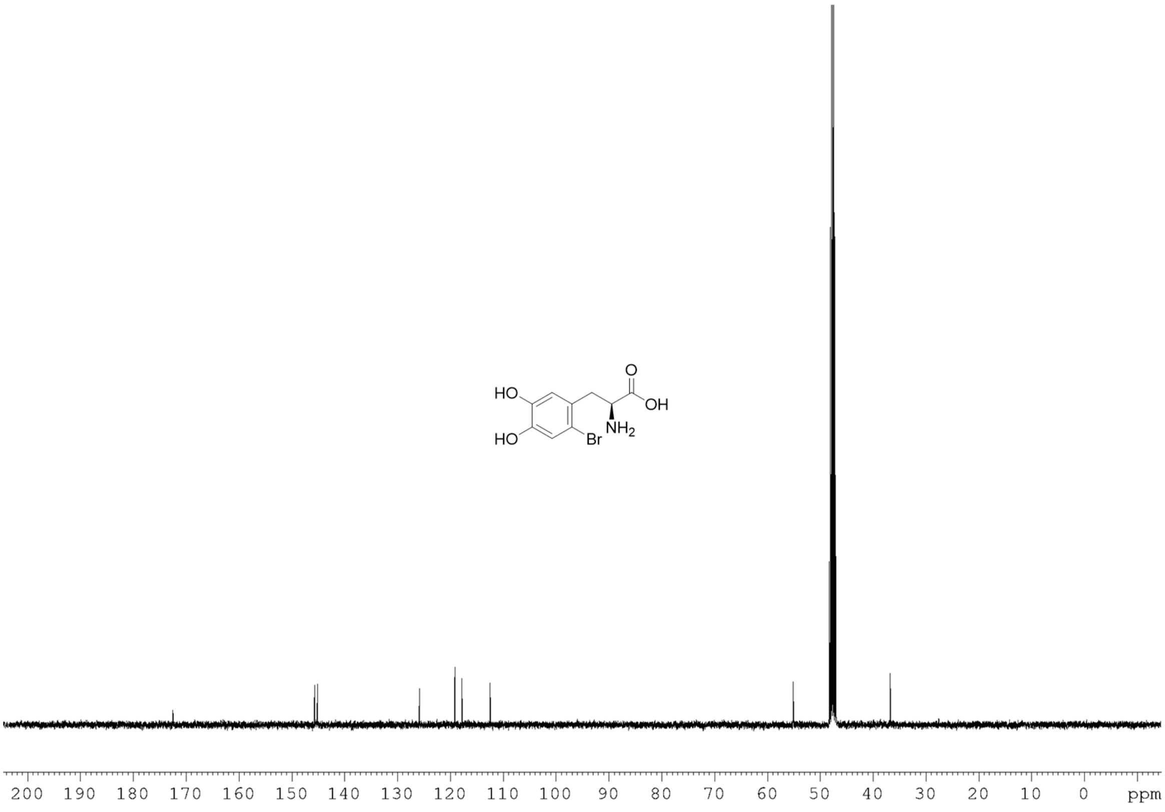

Figure S94. ^{13}C NMR spectrum of Br-L-DOPA **S15** in CD_3OD.

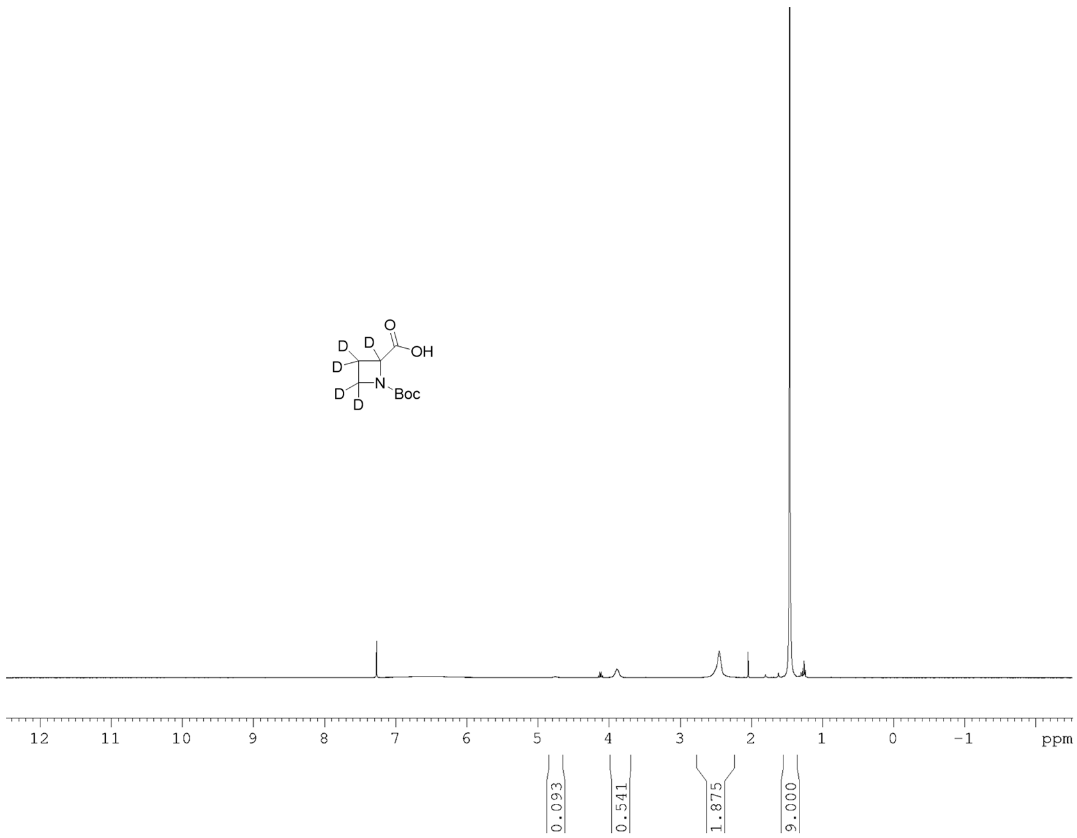

Figure S95. ^{1}H NMR spectrum of N-Boc-azetidine-2-caroxylic acid-d_5 in $CDCl_3$.

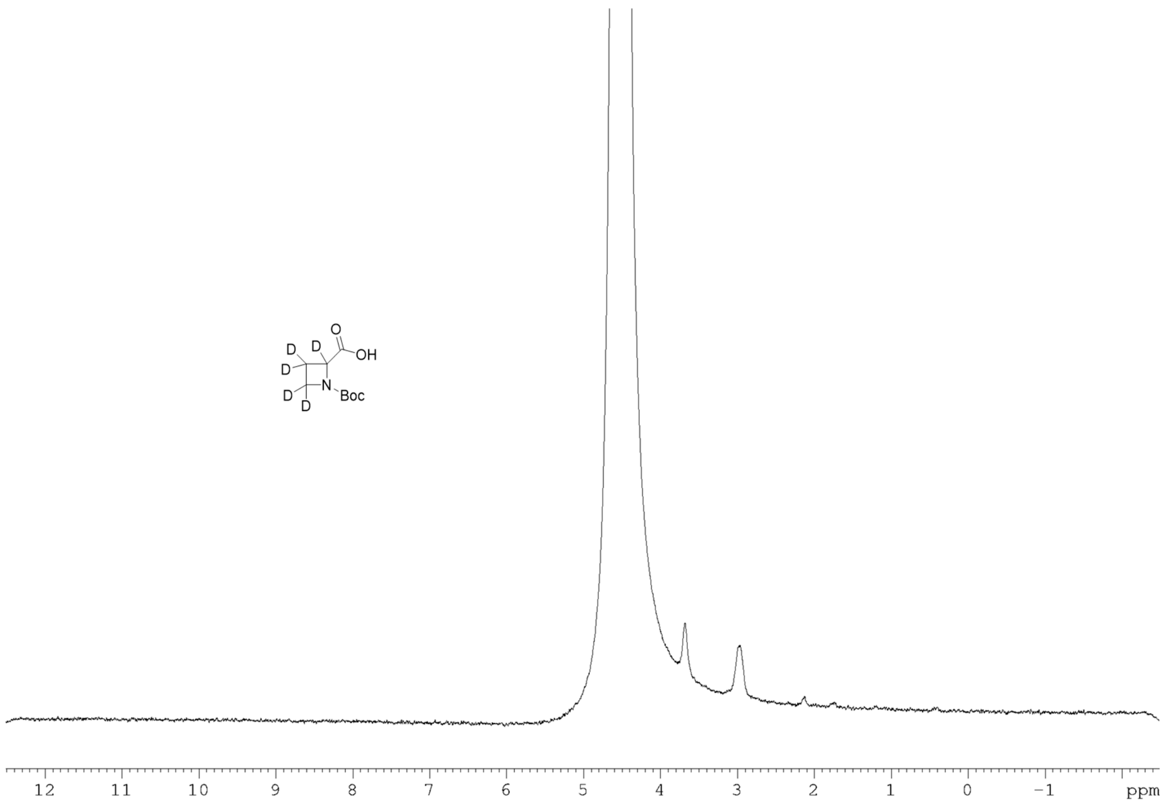

Figure S96. ^{2}H NMR spectrum of N-Boc-azetidine-2-caroxylic acid-d_5 in $CDCl_3$.

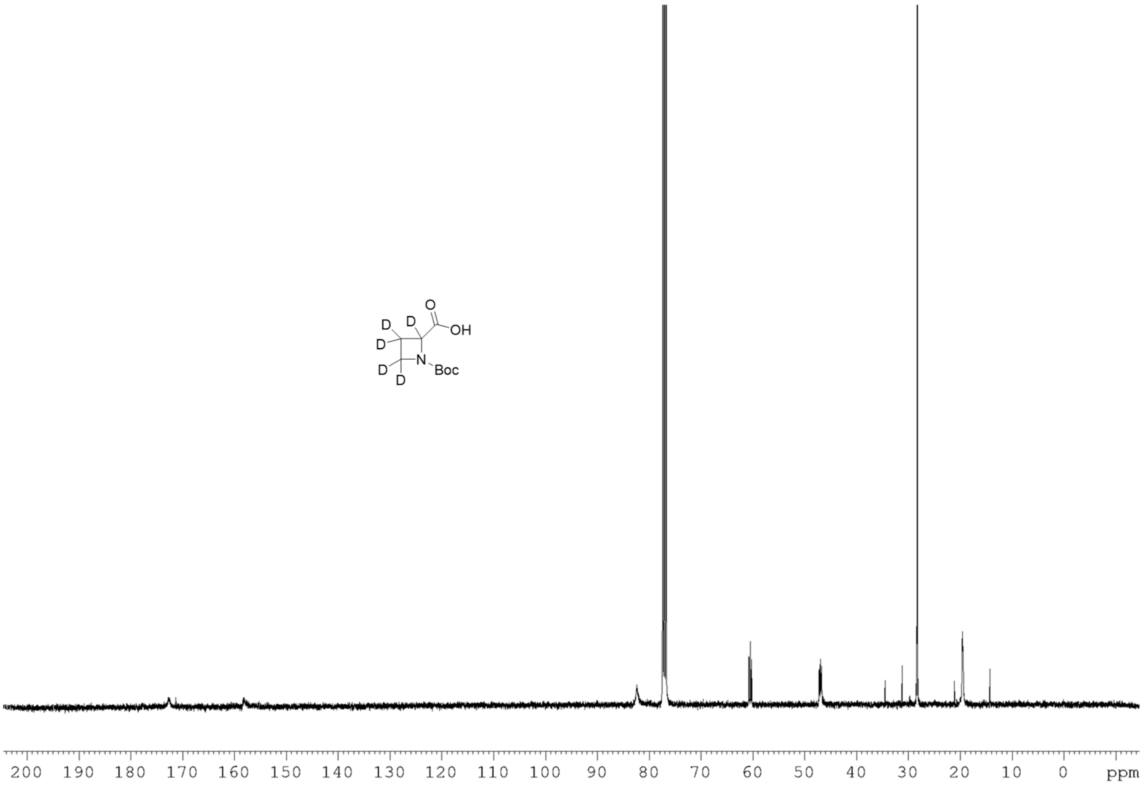

Figure S97. ^{13}C NMR spectrum of N-Boc-azetidine-2-caroxylic acid-d$_5$ in $CDCl_3$.

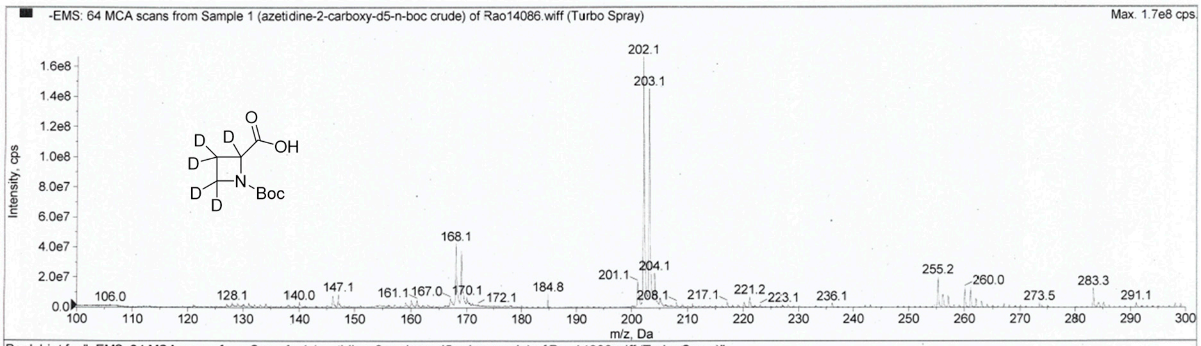

Peak List for " -EMS: 64 MCA scans from Sample 1 (azetidine-2-carboxy-d5-n-boc crude) of Rao14086.wiff (Turbo Spray)"

	m/z (Da)	Intensity (cps)	Peak area (counts)
24	167.0400	6.1923e6	4.4745e7
25	168.0600	4.2517e7	3.7990e8
26	169.0500	3.7637e7	2.9653e8
27	170.1000	6.7743e6	6.3217e7
28	171.0900	3.0229e6	2.8598e7
29	172.1100	1.9949e6	1.5833e7
30	184.8000	8.9139e6	1.1594e7
31	201.0900	1.6982e7	1.7396e8
32	202.1100	1.6664e8	1.4513e9
33	203.1300	1.4568e8	1.2753e9
34	204.1200	2.2925e7	2.0213e8
35	205.1100	6.6270e6	6.5415e7
36	206.1300	1.9800e6	1.7864e7
37	208.0500	3.5365e6	1.9711e7

Figure S98. ESI-MS: m/z 205 [M^{-1}] overall deuteration level 50%D; isotopologue distribution 5.5% *d*1, 46.2% *d*2, 40.2% *d*3, 6.0% *d*4 and 2.0% *d*5.

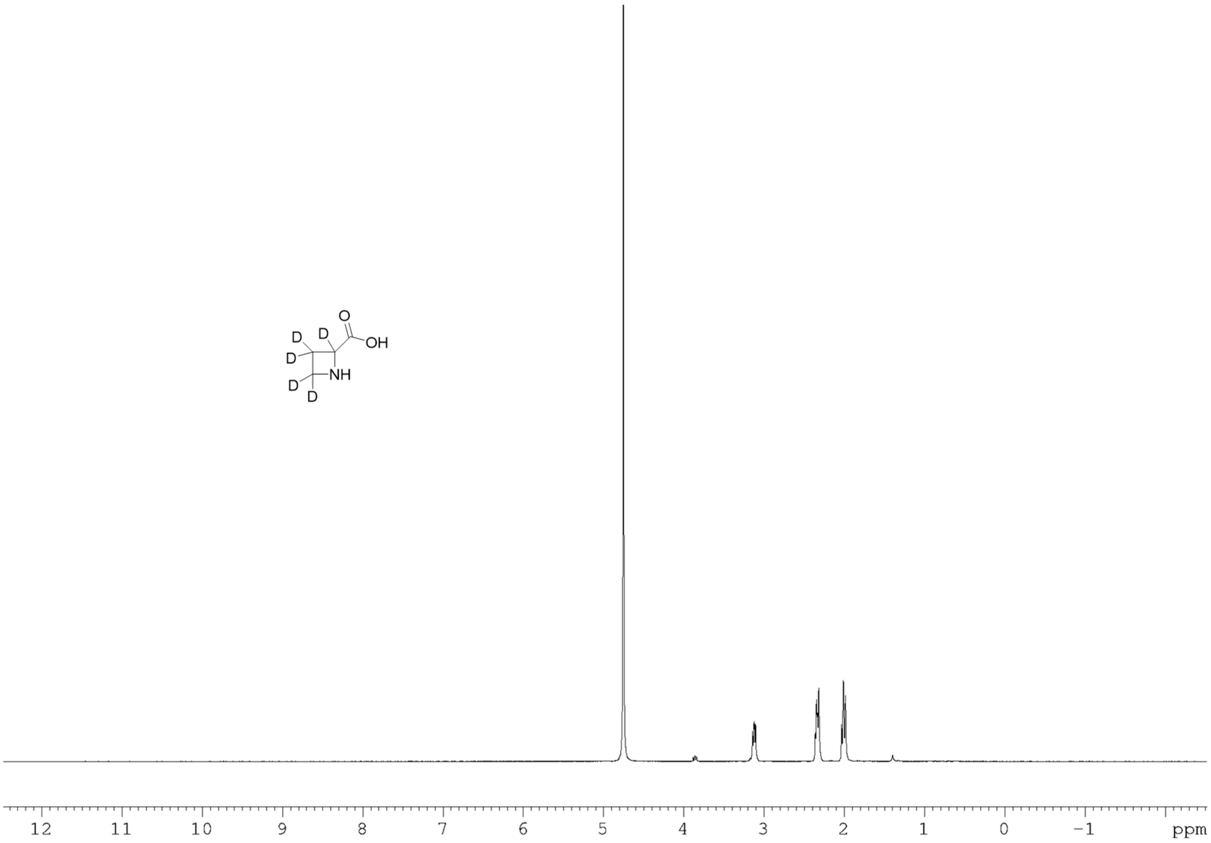

Figure S99. ^1H NMR spectrum of azetidine-2-caroxylic acid-d5 in D_2O.

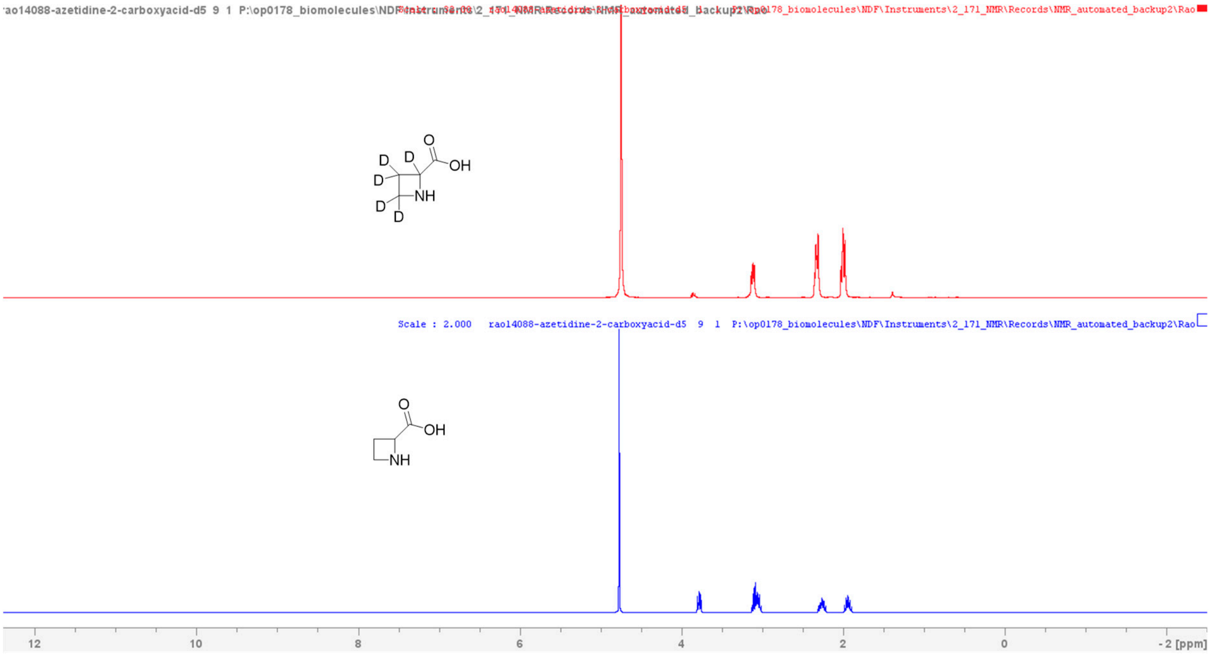

Figure S100. Comparative ^1H NMR spectrum of azetidine-2-caroxylic acid-d5 in D_2O (top red) and protonated of azetidine-2-caroxylic acid (blue bottom).

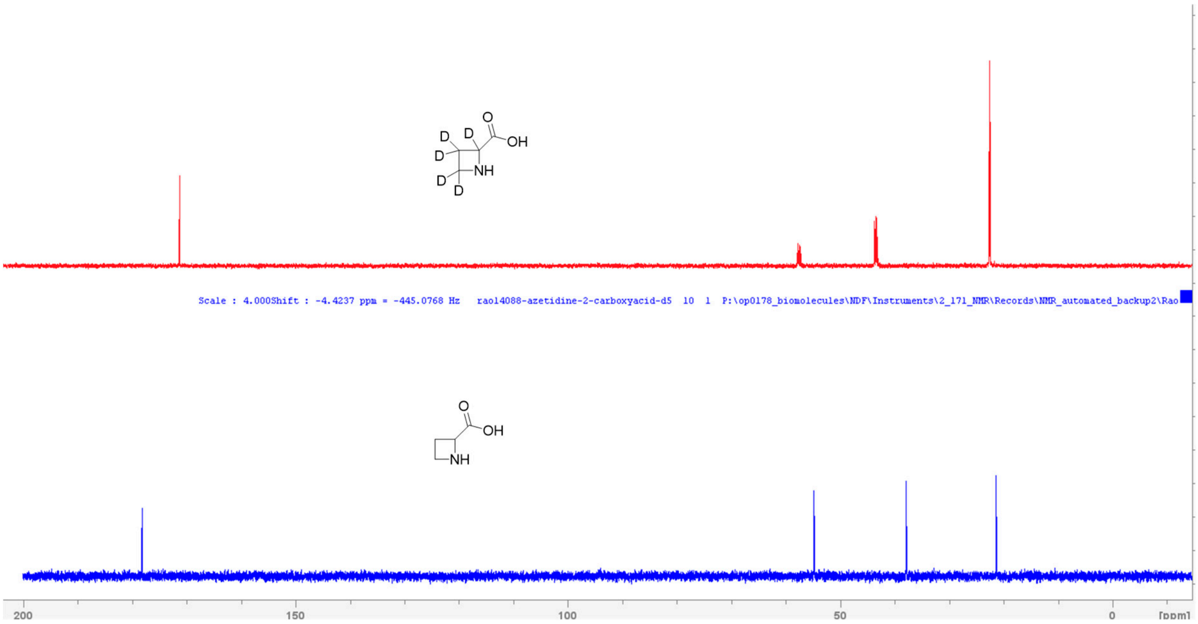

Figure S101. Comparative ^{13}C NMR spectrum of azetidine-2-caroxylic acid-d_5 in D_2O (top red) and protonated of azetidine-2-caroxylic acid (blue bottom).

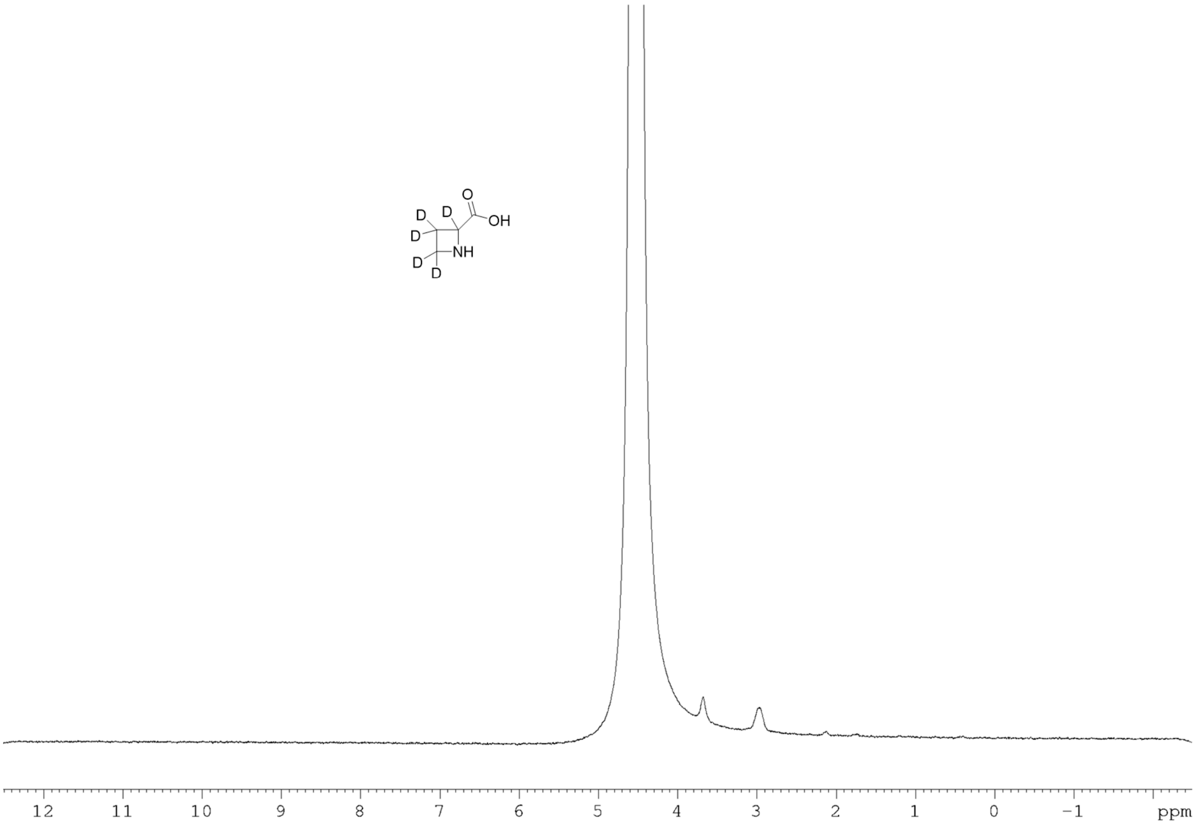

Figure S102. ^{2}H NMR spectrum of azetidine-2-caroxylic acid-d_5 in D_2O.

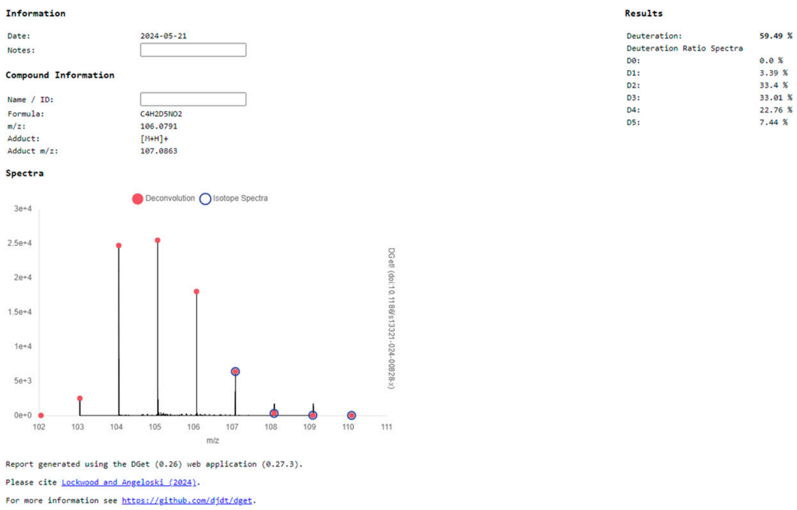

Figure S103. ESI-MS: m/z 107 [M^{-1}] mass spectrum of azetidine-2-caroxylic acid-d_5; overall deuteration level 59.49%; and isotopologue distribution 3.39% *d*1, 33.4% *d*2, 33.01% *d*3, 22.76% *d*4 and 7.44% *d*5. Deuteration levels are also calculated using DGet! Software.
